# A systematic review of the evidence for Canada's Physical Activity Guidelines for Adults

**DOI:** 10.1186/1479-5868-7-39

**Published:** 2010-05-11

**Authors:** Darren ER Warburton, Sarah Charlesworth, Adam Ivey, Lindsay Nettlefold, Shannon SD Bredin

**Affiliations:** 1 Cardiovascular Physiology and Rehabilitation Laboratory, University of British Columbia, Vancouver, Canada; 2 Experimental Medicine Program, Faculty of Medicine, University of British Columbia, Vancouver, Canada; 3 Cognitive and Functional Learning Laboratory, University of British Columbia, Vancouver, Canada

## Abstract

This systematic review examines critically the scientific basis for *Canada's Physical Activity Guide for Healthy Active Living *for adults. Particular reference is given to the dose-response relationship between physical activity and premature all-cause mortality and seven chronic diseases (cardiovascular disease, stroke, hypertension, colon cancer, breast cancer, type 2 diabetes (diabetes mellitus) and osteoporosis). The strength of the relationship between physical activity and specific health outcomes is evaluated critically. Literature was obtained through searching electronic databases (e.g., MEDLINE, EMBASE), cross-referencing, and through the authors' knowledge of the area. For inclusion in our systematic review articles must have at least 3 levels of physical activity and the concomitant risk for each chronic disease. The quality of included studies was appraised using a modified Downs and Black tool. Through this search we identified a total of 254 articles that met the eligibility criteria related to premature all-cause mortality (N = 70), cardiovascular disease (N = 49), stroke (N = 25), hypertension (N = 12), colon cancer (N = 33), breast cancer (N = 43), type 2 diabetes (N = 20), and osteoporosis (N = 2). Overall, the current literature supports clearly the dose-response relationship between physical activity and the seven chronic conditions identified. Moreover, higher levels of physical activity reduce the risk for premature all-cause mortality. The current Canadian guidelines appear to be appropriate to reduce the risk for the seven chronic conditions identified above and all-cause mortality.

## Introduction

There is considerable literature supporting the importance of habitual physical activity in the primary and secondary prevention of varied chronic conditions [1-16]. Routine physical activity is thought to be of benefit for over 25 chronic conditions [17]. Seven chronic diseases in particular have been associated with a physically inactive lifestyle including coronary artery disease, stroke, hypertension, colon cancer, breast cancer, type 2 diabetes (diabetes mellitus) and osteoporosis [18-20].

Canada has played a leading role in the development of physical activity guidelines for individuals across the lifespan. This includes the development (in 1998) of "Canada's Physical Activity Guide to Healthy Active Living" for adults between the ages of 20 and 55 yr [21], which was followed by "Canada's Physical Activity Guide to Healthy Active Living for Older Adults" [22], and "Canada's Physical Activity Guide to Healthy Active Living for Children and Youth" [23]. The adult guidelines (which are now approximately 10 years old) state generally that 20-55 yr adults should accumulate 60 min of daily physical activity or 30 min of moderate to vigorous exercise on at least 4 days a week [18,19].

**Figure 1:**
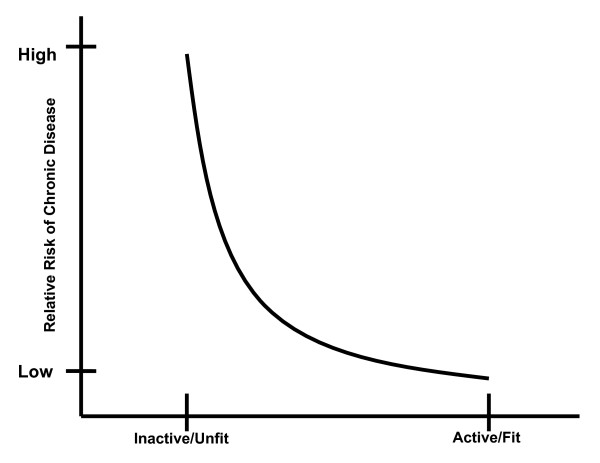
**Theoretical relationship between the risk for chronic disease and physical activity/fitness**.

We reported recently that Canada's adult guidelines were consistent with other international guidelines and were supported by a compelling body of literature [18,19]. We revealed strong evidence that routine physical activity was effective in the primary prevention of cardiovascular disease, stroke, hypertension, breast cancer, colon cancer, type 2 diabetes and osteoporosis. Moreover, physical activity appears to play an important role in the prevention of obesity and obesity-related co-morbidities. However, implicit in the adult guidelines is the belief that there is a dose-response relationship between physical activity and the associated health benefits. Moreover, a central belief in these guidelines and most international physical activity guidelines is that the dose-response relationship is curvilinear with the greatest health benefits seen in physically inactive individuals who become "more physically active." In fact, a consistent pattern (shown in Figure 1) has been hypothesized, wherein there are marked changes in health status with relatively minor increments in physical activity/fitness in individuals that are the least active/fit. Generally, the health benefits have been thought to level off at the upper end of the physical activity/fitness continuum (Figure 1). However, recent work (such as that provided by Gledhill and Jamnik in the Canadian Physical Activity and Lifestyle Approach) has speculated that there are likely multiple dose-response curves for various endpoints [24].

The primary purpose of this systematic review was to examine critically the current literature to determine whether or not a dose-response relationship exists between habitual physical activity and chronic disease. In particular, we sought to determine whether the key messaging "Every little bit counts, but more is even better - everyone can do it!" of the adult physical activity guidelines is supported by a strong body of evidence.

Due to the breadth of literature, we have chosen to focus on the relationship between physical activity and all-cause mortality, and the seven chronic conditions that are thought to be reduced greatly with habitual physical activity (i.e., cardiovascular disease (excluding stroke), stroke, hypertension, colon cancer, breast cancer, type 2 diabetes and osteoporosis) (see Table 1). Owing to the nature of the physical activity guidelines, the emphasis of this paper was on primary prevention, despite the clear evidence that routine physical activity is also an effective secondary preventative strategy against many chronic conditions [16,18,19]. Accordingly, our primary objectives were to examine the evidence for a dose-response relationship between: 1) physical activity and all-cause mortality, and 2) physical activity and incidence of the following chronic conditions (cardiovascular disease (except stroke), stroke, hypertension, type 2 diabetes, colon cancer, breast cancer, and osteoporosis.

**Table 1:** Relative risks (RR) and population attributable risks (PAR%) for physical inactivity in Canada, Australia, and the USA.

	Canada		Australia		USA	
**Disease**	**RR**	**PAR%**	**RR**	**PAR%**	**RR**	**PAR%**

CHD	1.45	19.4	1.5	18	2.0	22

Stroke	1.60	24.3	2.0	16	na	Na

Hypertension	1.30	13.8	na	na	1.5	12

Colon Cancer	1.41	18.0	1.5	19	2.0	22

Breast Cancer	1.31	14.2	1.1	9	1.2	5

Type 2 Diabetes	1.50	21.1	1.3	13	1.5	12

Osteoporosis	1.59	24.0	1.4*	18*	2.0	18*

Source: Canadian Data [20]; Australian Data [161]; US Data: [162]. *Evaluated the incidence of falls/fractures.

## Methods

### Criteria for considering studies for this review

Our research team utilized a rigorous, systematic, and evidence-based approach to examine critically the levels of evidence on physical activity and the risk for premature mortality and chronic disease. Any studies that evaluated the relationship between at least **three** different levels of physical activity and mortality or incidence of chronic disease were eligible for inclusion. Therefore, excluded studies included those that examined only the most active versus least active populations (e.g., sedentary/inactive vs. physically active). Any form of physical activity/exercise measurement (e.g., self-report, pedometer, accelerometer, maximal aerobic power (VO_2 _max)) was eligible for inclusion. The key outcomes were mortality and incidence of chronic disease. Only published, English language studies examining adults (e.g., 19-65 yr) were included. Participants must have previously been healthy (asymptomatic) adults without established chronic disease. There was no restriction according to study design.

To examine the relative risk reductions associated with physical activity, we calculated the mean and median risk reductions across studies focusing on the highest level versus the lowest level of physical activity/fitness. For each study we also determined whether or not a dose-response relationship was present (i.e., reflecting a progressive decrease in the risk with increasing physical activity/fitness levels).

### Search strategy

Literature searches were conducted in the following electronic bibliographical databases:

• MEDLINE (1950-March 2008, OVID Interface);

• EMBASE (1980- March 2008, OVID Interface),

• CINAHL (1982- March 2008, OVID Interface);

• PsycINFO (1840- March 2008, Scholars Portal Interface);

• Cochrane Library (-March 2008),

• SPORTDiscus (-March 2008).

The Medical Subject Headings (MeSH) were kept broad. See tables 2, 3, 4, 5, 6, 7, 8 and 9 for the complete search strategy and keywords used. The electronic search strategies were created and carried out by researchers experienced with systematic reviews of the literature (DW and LN). The citations and applicable electronic versions of the article (where available) were downloaded to an online research management system (RefWorks, Bethesda, Maryland, USA).

**Table 2:** Results of the MEDLINE literature search regarding all-cause mortality.

#	Searches (28 Feb 2008)	Results
1	exp Physical Fitness/	15236
2	Motor Activity/	49721
3	exp Physical Endurance/	15383
4	exp Exercise/	57742
5	exp Exertion/	88903
6	exp Sports/	71887
7	exp exercise therapy/	17231
8	exp exercise tolerance/	4192
9	exp health behaviour/	59409
10	leisure time physical activity.mp	996
11	occupational physical activity.mp	190
12	exp Pliability/	2279
13	exp Muscle Strength/	5717
14	musc$ power.mp	965
15	exp Back/	12821
16	1 or 2 or 3 or 4 or 5 or 6 or 7 or 8 or 9 or 10 or 11 or 12 or 13 or 14 or 15	291635
17	dose-response.mp	321066
18	intensity.mp	142881
19	volume.mp	298471
20	exp Energy Metabolism/	206808
21	exp oxygen consumption/	83352
22	exp time factors/	763712
23	17 or 18 or 19 or 20 or 21 or 22	1651633
24	16 and 23	67698
25	exp Mortality/	190058
26	all cause mortality.mp	4618
27	25 or 26	192720
28	24 and 27	421
29	limit 28 to (english and humans and "all adult (19 plus years)	279

**Table 3:** Results of the MEDLINE literature search regarding cardiovascular disease.

Search #	Searches (3 Mar 2008)	Results
1	exp Physical Fitness/	15244
2	Motor Activity/	49751
3	exp Physical Endurance/	15408
4	exp Exercise/	57806
5	exp Exertion/	88967
6	exp Sports/	71931
7	exp exercise therapy/	17243
8	exp exercise tolerance/	4205
9	exp health behaviour/	59467
10	leisure time physical activity.mp	998
11	occupational physical activity.mp	191
12	exp Pliability/	2289
13	exp Muscle Strength/	5731
14	musc$ power.mp	965
15	exp Back/	12822
16	1 or 2 or 3 or 4 or 5 or 6 or 7 or 8 or 9 or 10 or 11 or 12 or 13 or 14 or 15	291817
17	dose-response.mp	321198
18	intensity.mp	142955
19	volume.mp	298620
20	exp Energy Metabolism/	206886
21	exp oxygen consumption/	83387
22	exp time factors/	764091
23	17 or 18 or 19 or 20 or 21 or 22	1652372
24	16 and 23	67760
25	exp Cardiovascular Diseases/	1411730
26	exp Heart diseases/	675083
27	exp Myocardial infarction/	116070
28	exp Death, Sudden Cardiac/	6772
29	exp Coronary Artery Disease/	18137
30	exp Coronary Disease/	144236
31	exp Vascular Diseases	1018275
32	25 or 26 or 27 or 28 or 29 or 30 or 31	1411730
33	24 and 32	9603
34	limit 33 to (english language and humans and "all adult (19 plus years)")	5544

**Table 4:** Results of the MEDLINE literature search regarding stroke.

Search #	Searches (29 Feb 2008)	Results
1	exp Physical Fitness/	15241
2	Motor Activity/	49744
3	exp Physical Endurance/	15387
4	exp Exercise/	57764
5	exp Exertion/	88921
6	exp Sports/	71907
7	exp exercise therapy/	17237
8	exp exercise tolerance/	4196
9	exp health behaviour/	59430
10	leisure time physical activity.mp	996
11	occupational physical activity.mp	190
12	exp Pliability/	2288
13	exp Muscle Strength/	5720
14	musc$ power.mp	965
15	exp Back/	12821
16	1 or 2 or 3 or 4 or 5 or 6 or 7 or 8 or 9 or 10 or 11 or 12 or 13 or 14 or 15	291718
17	dose-response.mp	321133
18	intensity.mp	142919
19	volume.mp	298526
20	exp Energy Metabolism/	206837
21	exp oxygen consumption/	83359
22	exp time factors/	763871
23	17 or 18 or 19 or 20 or 21 or 22	1651958
24	16 and 23	67720
25	exp Stroke/	45243
26	exp Cerebrovascular Disorders/	196243
27	exp Brain Ischemia/	58943
28	exp Brain Infarction/ or exp Cerebral Infarction	21357
29	exp Infarction, Middle Cerebral Artery/ or exp Intracranial Aneurysm/ or exp Subarachnoid	46725
30	Hemorrhage/ or exp Cerebral Hemorrhage/exp Ischemic Attack, Transient/	14753
31	25 or 26 or 27 or 28 or 29 or 30	196243
32	24 and 31	692
33	limit 32 to (english language and humans and "all adult (19 plus years)")	291

**Table 5:** Results of the MEDLINE literature search regarding hypertension.

Search #	Searches (3 Mar 2008)	Results
1	exp Physical Fitness/	15244
2	Motor Activity/	49751
3	exp Physical Endurance/	15408
4	exp Exercise/	57806
5	exp Exertion/	88967
6	exp Sports/	71931
7	exp exercise therapy/	17243
8	exp exercise tolerance/	4205
9	exp health behaviour/	59467
10	leisure time physical activity.mp	998
11	occupational physical activity.mp	191
12	exp Pliability/	2289
13	exp Muscle Strength/	5731
14	musc$ power.mp	965
15	exp Back/	12822
16	1 or 2 or 3 or 4 or 5 or 6 or 7 or 8 or 9 or 10 or 11 or 12 or 13 or 14 or 15	291817
17	dose-response.mp	3211987
18	intensity.mp	142955
19	volume.mp	298620
20	exp Energy Metabolism/	206886
21	exp oxygen consumption/	83387
22	exp time factors/	764091
23	17 or 18 or 19 or 20 or 21 or 22	1652372
24	exp Hypertension/	168466
25	exp Blood Pressure/	205571
26	exp Blood Pressure Determination/ or exp Blood Pressure Monitoring, Ambulatory/ or exp Blood	18244
27	Pressure Monitors/24 or 25 or 26	336025
28	16 and 23 and 27	5647
29	limit 28 to (english language and humans and "all adult (19 plus years)")	3642

**Table 6:** Results of the MEDLINE literature search regarding colon cancer.

Search #	Searches (3 Mar 2008)	Results
1	exp Physical Fitness/	15244
2	Motor Activity/	49751
3	exp Physical Endurance/	15408
4	exp Exercise/	57806
5	exp Exertion/	88967
6	exp Sports/	71931
7	exp exercise therapy/	17243
8	exp exercise tolerance/	4205
9	exp health behaviour/	59467
10	leisure time physical activity.mp	998
11	occupational physical activity.mp	191
12	exp Pliability/	2289
13	exp Muscle Strength/	5731
14	musc$ power.mp	965
15	exp Back/	12822
16	1 or 2 or 3 or 4 or 5 or 6 or 7 or 8 or 9 or 10 or 11 or 12 or 13 or 14 or 15	291817
17	dose-response.mp	321198
18	intensity.mp	142955
19	volume.mp	298620
20	exp Energy Metabolism/	206886
21	exp oxygen consumption/	83387
22	exp time factors/	764091
23	17 or 18 or 19 or 20 or 21 or 22	1652372
24	exp Colonic Neoplams/	51780
25	exp Rectal Neoplasms/	28011
26	exp Colorectal Neoplasms/	99982
27	exp Colorectal Neoplasms/, Hereditary Nonpolyposis/ or exp Intestinal Neoplasms.	117563
28	24 or 25 or 26 or 27	117563
29	16 and 23 and 28	108
30	limit 29 to (53nglish language and humans and "all adult (19 plus years)")	77

**Table 7:** Results of the MEDLINE literature search regarding breast cancer.

Search #	Searches (28 Feb 2008)	Results
1	exp Physical Fitness/	15236
2	Motor Activity/	49721
3	exp Physical Endurance/	15383
4	exp Exercise/	57742
5	exp Exertion/	88903
6	exp Sports/	71887
7	exp exercise therapy/	17231
8	exp exercise tolerance/	4192
9	exp health behaviour/	59409
10	leisure time physical activity.mp	996
11	occupational physical activity.mp	190
12	exp Pliability/	2279
13	exp Muscle Strength/	5717
14	musc$ power.mp	965
15	exp Back/	12821
16	1 or 2 or 3 or 4 or 5 or 6 or 7 or 8 or 9 or 10 or 11 or 12 or 13 or 14 or 15	291635
17	dose-response.mp	321066
18	intensity.mp	142881
19	volume.mp	298471
20	exp Energy Metabolism/	206808
21	exp oxygen consumption/	83352
22	exp time factors/	763712
23	17 or 18 or 19 or 20 or 21 or 22	1651633
24	exp Breast Neoplasms/	149817
25	16 and 23 and 24	296
26	limit 25 to (54 nglish language and humans and "all adult (19 plus years)"	216

**Table 8:** Results of the MEDLINE literature search regarding type 2 diabetes.

Search #	Searches (29 Feb 2008)	Results
1	exp Physical Fitness/	15241
2	Motor Activity/	49744
3	exp Physical Endurance/	15387
4	exp Exercise/	57764
5	exp Exertion/	88921
6	exp Sports/	71907
7	exp exercise therapy/	17237
8	exp exercise tolerance/	4196
9	exp health behaviour/	59430
10	leisure time physical activity.mp	996
11	occupational physical activity.mp	190
12	exp Pliability/	2288
13	exp Muscle Strength/	5720
14	musc$ power.mp	965
15	exp Back/	12821
16	1 or 2 or 3 or 4 or 5 or 6 or 7 or 8 or 9 or 10 or 11 or 12 or 13 or 14 or 15	291718
17	dose-response.mp	321133
18	intensity.mp	142919
19	volume.mp	298526
20	exp Energy Metabolism/	206837
21	exp oxygen consumption/	83359
22	exp time factors/	763871
23	17 or 18 or 19 or 20 or 21 or 22	1651958
24	16 and 23	67720
25	exp Blood Glucose/or exp Diabetes Mellitus, Type 2/	132583
26	exp Hyperglycemia/	16214
27	exp Glucose Intolerance/ or exp Glucose Tolerance Test/	24986
28	exp Hyperinsulinism/	30490
29	25 or 26 or 27 or 28	165157
30	29 and 24	3006
31	Limit 30 to (english language and humans and "all adult (19 plus years)")	1985

**Table 9:** Results of the MEDLINE literature search regarding osteoporosis.

Search #	Searches (29 feb 2008)	Results
1	exp Physical Fitness/	15241
2	Motor Activity/	49744
3	exp Physical Endurance/	15387
4	exp Exercise/	57764
5	exp Exertion/	88921
6	exp Sports/	71907
7	exp exercise therapy/	17237
8	exp exercise tolerance/	4196
9	exp health behaviour/	59430
10	leisure time physical activity.mp	996
11	occupational physical activity.mp	190
12	exp Pliability/	2288
13	exp Muscle Strength/	5720
14	musc$ power.mp	965
15	exp Back/	12821
16	1 or 2 or 3 or 4 or 5 or 6 or 7 or 8 or 9 or 10 or 11 or 12 or 13 or 14 or 15	291718
17	dose-response.mp	321133
18	intensity.mp	142919
19	volume.mp	298526
20	exp Energy Metabolism/	206837
21	exp oxygen consumption/	83359
22	exp time factors/	763871
23	17 or 18 or 19 or 20 or 21 or 22	1651958
24	exp Osteoporosis, Postmenopausal/ or exp Osteoporosis/	31532
25	exp Fractures, Bone/ or exp Bone Density/	125269
26	exp Bone Diseases/ or exp Bone Diseases, Metabolic/	308084
27	exp "Bone and bones"/	369634
28	exp Tensile Strength/	12050
29	exp Compressive Strength	2838
30	24 or 25 or 26 or 27 or 28 or 29	642158
31	16 and 23 and 30	2138
32	limit 31 to (english language and humans and "all adult (19 plus years)")	1193

### Screening

Two reviewers (LN and SC) screened independently the title and abstract of the citations to identify potential articles for inclusion. Duplicate citations were removed. The reviewers were not blinded to the authors or journals. Biographies of key studies and reviews in the field were also cross-referenced for further articles. For those articles that appeared relevant, the full text was obtained and data was extracted using a common template. In cases of disagreement, discussion with a third reviewer (DW) was used to achieve consensus. Full (100%) consensus was achieved. All studies that were excluded during the citation and full-article screening processes were recorded along with the reasons for exclusion.

### Data Extraction

Two reviewers (LN and SC) completed standardized data extraction forms, which were verified by two other reviewers (DW and SB). We extracted information regarding the study design, the country where the study was conducted, the participant characteristics, the sample size, the objectives of the study, the methodologies employed, the major outcomes (i.e., mortality, incidence of chronic disease, physical activity levels/classifications), and the comments and conclusions made based on the findings of the study. The reviewers were not blinded to the journal or the author names when extracting information from the articles.

### Level of Evidence

The approach used to establish the level and grade of evidence was consistent with that used during creation of the "Canadian clinical practice guidelines on the management and prevention of obesity in adults and children" [25]. The level of evidence provides information regarding the strength of the evidence in favour of physical activity/exercise in the primary prevention of premature mortality and the seven chronic diseases of primary interest. This evaluation process is based on a pre-defined and objective criteria (see Table 10).

**Table 10:** The levels and grade of evidence scaling criteria applied to the articles.

Level of Evidence	Criteria
Level 1	Randomized control trials without important limitations

Level 2	• Randomized control trials with important limitations
	• Observational studies (non-randomized clinical trials or cohort studies) with overwhelming evidence

Level 3	Other observational studies (prospective cohort studies, case-control studies, case series)

Level 4	Inadequate or no data in population of interest
	Anecdotal evidence or clinical experience

	

**Grade of Evidence**	**Criteria**

Grade A	Strong recommendation (action can apply to most individuals in most circumstances)
	• Benefits clearly outweigh risks (or vice-versa)
	• Evidence is at Level 1, 2, or 3

Grade B	Weak recommendation (action may differ depending on individual's characteristics or other circumstances)
	• Unclear if benefits outweigh risks
	• Evidence is at Level 1, 2, or 3

Grade C	Consensus recommendation (alternative actions may be equally reasonable)
	• Unclear if benefits outweigh risks
	• Evidence is at Level 3 or 4

The grade for each article provides information regarding whether physical activity is effective in the primary prevention of the varied conditions evaluated (Table 10). Where applicable this grade informs the reader about the potential risk of the physical activity. A study that receives the highest grading would indicate that the benefits clearly outweigh the risks and receive a strong recommendation.

### Quality Assessment

The quality of each study was also established using the procedures of Gorber et al. [26]. Owing to the fact that only observational study designs were included in our systematic review, we used the Downs and Black [27] scale to assess the quality of non-randomized investigations. Similar to the work of Prince et al. [28] we chose to include the most relevant components of the scoring tool. Therefore, a modified version of the Downs and Black checklist was used with the final checklist consisting of 15 items with a maximum score of 15 points. Higher points reflected a superior quality of investigation.

## Results

### Physical Inactivity and All-Cause Mortality

**Figure 2:**
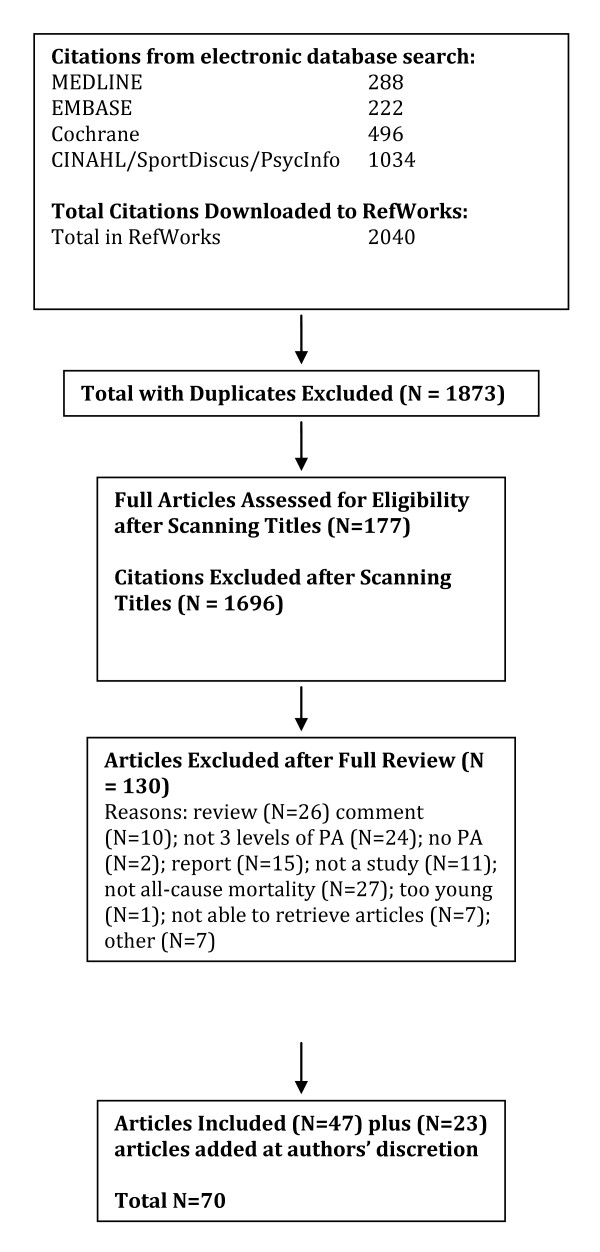
**Results of the Literature Search for All-Cause Mortality**.

A total of 2040 citations were identified during the electronic database search (Figure 2). Of these citations, 288 were identified in MEDLINE, 222 in EMBASE, 496 in Cochrane, and 1034 in the CINAHL/SportDiscus/PsychInfo search. A total of 167 duplicates were found, leaving a total of 1873 unique citations. A total of 1696 articles were excluded after scanning, leaving a total of 177 articles for full review. From these articles 130 were excluded after full review leaving 47 articles for inclusion in the systematic review. An additional 23 articles were added to the review based on the authors' knowledge of the area. The reasons for exclusion included review articles (n = 26), commentary (n = 10), did not report 3 levels of physical activity (n = 24), no objective measure of physical activity (n = 2), report (n = 15), not a formal study (n = 11), not related to all-cause mortality (n = 27), the participants were too young (n = 1), not able to retrieve articles (n = 7), and other (n = 7). Therefore, a total of 70 articles were included in the systematic review of the literature regarding the relationship between physical activity and premature mortality.

The majority of the studies included in our systematic review were prospective cohort investigations (Table 11). These studies involved a total of 1,525,377 participants; averaging 21,791 participants per study (range 302-252,925). There were a total of 111,125 reported cases of premature all-cause mortality (ranging per study from 43-10,952). The total length of study follow-up for the prospective cohort studies averaged 11.1 yr (ranging from 0.5-28 yr). The articles were published over a 22 yr period ranging from 1985 to 2007. These studies involved large samples of men and women from regions throughout the world.

**Table 11:** Studies examining the relationship between physical activity and all-cause mortality.

Publication Country Study Design Quality Score	Objective	Population	Methods	Outcome	Comments and Conclusions
Blair et al 1989 [7]	To study physical fitness (PF) and risk of all-cause mortality in men and women.	• n = 13,344 (10,224 men; 3,120 women)	Baseline and 8 year follow-up	• 283 deaths	Low levels of PF increase the risk for premature mortality.
		• Sex: Men and women		Adjusted risk ratio (RR), 95% confidence interval (CI)	
USA		• Age: 20->60 years (yr)	PF assessment: Maximal treadmill exercise test.		
Prospective cohort		• Characteristics: Participants were given a preventative Medicine examination including maximal treadmill exercise test	Fitness categorized into quintiles:	Men	
D & B score = 12			Q1 = least fit	• Q1 = 3.44 (2.05-5.77)	
			Q2	• Q2 = 1.37 (0.76-2.50)	
			Q3	• Q3 = 1.46 (0.81-2.63)	
			Q4	• Q4 = 1.17 (0.63-2.17)	
			Q5 = most fit	• Q5 = 1.00 (referent)	
				Women	
				• Q1 = 4.65 (2.22-9.75)	
				• Q2 = 2.42 (1.09-5.37)	
				• Q3 = 1.43 (0.60-3.44)	
				• Q4 = 0.76 (0.27-2.11)	
				• Q5 = 1.00 (referent)	

Myers et al 2004 [32]	To determine the effects of PF and physical activity (PA) on all-cause mortality.	• n = 6,213	Baseline and mean 5.5 ± 2.0 year follow-Up	• 1,256 deaths	Being fit or active is associated with >50% reductions in mortality risk.
		• Sex: Men			
USA		• Age: Mean 59.0 ± 11.2 yr		PF Level hazard ratio (HR) (95% CI)	
		• Characteristics: Men referred for exercise testing	PF assessment: Treadmill test to measure VO_2 _peak	• G1 = 1.00 (referent)	PF predicted mortality more strongly than PA.
Prospective cohort				• G2 = 0.59 (0.52-0.68)	
				• G3 = 0.46 (0.39-0.55)	
				• G4 = 0.28 (0.23-0.34)	Increasing PA (by 1000 kcal/wk or 1 MET) confers a mortality benefit of 20%.
D & B score = 12			PA assessment: Self reported PA divided into 4 groups		
				PA Level HR (95% CI)	
			G1 = Lowest level	• G1 = 1.00 (referent)	
			G2	• G2 = 0.63 (0.36-1.10)	
			G3	• G3 = 0.42 (0.23-0.78)	
			G4 = Highest level	• G4 = 0.38 (0.19-0.73)	

Blair et al 1995 [36]	To evaluate the relationship between changes in PF and risk of mortality in men.	• n = 9,777	4.9 year mean follow-up	• 223 deaths	Men who maintained or increased adequate PF had a reduced risk for all-cause mortality than individuals who were consistently unfit.
		• Sex: Men			
		• Age: 20-82 yr		RR (95% CI)	
USA		• Characteristics: Participants were given a preventative medicine examination including maximal treadmill exercise test	PF assessment: Maximal exercise test at baseline and follow-up	• G1 = 1.00 (referent)	
Prospective cohort				• G2 = 0.56 (0.41-0.75)	
				• G3 = 0.52 (0.38-0.70)	
				• G4 = 0.33 (0.23-0.47)	
D & B score = 13			Groups based on changes in PF		
			G1 = unfit to unfit		
			G2 = unfit to fit		
			G3 = fit to unfit		
			G4 = fit to fit		

Bijnen et al 1999 [37]	To examine the association of PA at baseline and 5 years	• n = 472	1985 and 1990	• 118 deaths	Recent levels of PA were more important for mortality risk than PA 5 years previously.
		• Sex: Men			
		• Age: >65 yr	PA assessment: Questionnaire, divided into tertiles: Lowest Middle Highest	Multivariate adjusted RR (95% CI)	
Netherlands	previously with all- cause mortality risk in a cohort of elderly Dutch men.	• Characteristics: Mostly independently living elders (~95%)		PA in 1985: Lowest tertile = 1.00 (referent) Middle tertile	
Retrospective cohort		• Zutphen Elderly Study		• Total activity = 1.25 (0.79- 1.99)	Becoming or remaining sedentary increased the mortality risk.
D & B score = 12				• Walking = 0.97 (0.60-1.57)	
				• Bike = 0.97 (0.59-1.57)	
				• Gardening = 0.66 (0.39-1.10)	
				• Other = 1.08 (0.66-1.78)	
				• Heavy activity = 0.73 (0.45-1.17)	
				• Non heavy activity = 0.89 (0.57-1.40)	
				Highest tertile	
				• Total activity = 1.25 (0.73-2.12)	
				• Walking = 0.94 (0.58-1.55)	
				• Bike = 1.07 (0.61-1.88)	
				• Gardening = 0.77 (0.42-1.39)	
				• Other = 1.24 (0.74-2.07)	
				• Heavy activity = 0.76 (0.44-1.32)	
				• Non heavy activity = 0.94 (0.58-1.53)	
				PA in 1990:	
				Lowest tertile = 1.00 (referent)	
				Middle tertile	
				• Total activity = 0.56 (0.35-0.89)	
				• Walking = 0.82 (0.51-1.32)	
				• Bike = 0.49 (0.29-0.82)	
				• Gardening = 1.67 (1.00-2.79)	
				• Other = 0.93 (0.53-1.65)	
				• Heavy activity = 1.19 (0.73-1.92)	
				• Non heavy activity = 0.61 (0.38-0.99)	
				Highest tertile	
				• Total activity = 0.44 (0.25-0.80)	
				• Walking = 1.17 (0.70-1.96)	
				• Bike = 0.43 (0.23-0.80)	
				• Gardening = 1.03 (0.55-1.94)	
				• Other = 0.74 (0.44-1.23)	
				• Heavy activity = 0.72 (0.40-1.31)	
				• Non heavy activity = 0.65 (0.40-1.05)	

Gregg et al 2003 [39]	To examine the relationship of changes in PA and mortality among older women.	• n = 9,518	Baseline (1986-1988) and median 10.6 year follow-up (1992-1994)	• 2,218 deaths	Increasing and maintaining PA levels could lengthen life for older women but appears to provide less benefit for women aged at least 75 years and those with poor health status.
		• Sex: Women	PA Assessment: Questionnaire, divided into quintiles of PA (kcal/wk)		
		• Age: ≥ 65 yr		Multivariate adjusted HRR	
USA		• Characteristics: White community dwelling participants from 4 US research centres		(95% CI): Quintiles of total	
			Q1= <163	PA	
Prospective cohort			Q2 = 163-503	• Q1 = 1.00 (referent)	
			Q3 = 504-1045	• Q2 = 0.73 (0.64-0.82)	
			Q4 = 1046-1906	• Q3 = 0.77 (0.68-0.87)	
D & B score = 13			Q5 = ≥ 1907	• Q4 = 0.62 (0.54-0.71)	
				• Q5 = 0.68 (0.59-0.78)	
				Walking HRR (95% CI)	
				• Q1 = 1.00 (referent)	
			Quintiles of walking(kcal/wk)	• Q2 = 0.91 (0.81-1.02)	
			Q1 = <70	• Q3 = 0.78 (0.68-0.88)	
			Q2 = 70-186	• Q4 = 0.71 (0.63-0.82)	
			Q3 = 187-419	• Q5 = 0.71 (0.62-0.82)	
			Q4 = 420-897		
			Q5 = 898		
				Multivariate adjusted HRR (95% CI)	
				Change in activity level: Sedentary at baseline	
				• Staying sedentary = 1.00 (referent)	
				• Became active = 0.52 (0.40-0.69)	
				Mod / high active at baseline	
				• Became sedentary = 0.92 (0.77-1.09)	
				• Stayed active = 0.68 (0.56-0.82)	

Wannamethee et al 1998 [40]	To study the relationship between heart rate, PA and all- cause mortality.	• n = 5,934	Baseline (1978-1980) and 12-14 year follow-up	• 219 deaths	Maintaining or taking up light or moderate PA reduces mortality in older men.
		• Sex: Men			
		• Age: Mean 63 yr		Multivariate adjusted RR (95% CI),	
UK		• Characteristics: Healthy, sedentary(4,311 were considered "healthy" in 1992)	PA assessment: Questionnaire, split into groups	PA	
Prospective cohort		• The British Regional Heart Study		• G1 = 1.00 (referent)	
				• G2 = 0.61 (0.43-0.86)	
				• G3 = 0.50 (0.31-0.79)	
D & B score = 12			PA score	• G4 = 0.65 (0.45-0.94)	
			G1 =		
			Inactive/occasional	Regular walking	
			G2 = Light	• G1 = 1.00 (referent)	
			G3 = Moderate	• G2 = 1.15 (0.73-1.79)	
			G4 = Moderately	• G3 = 1.06 (0.75-1.50)	
			vigorous/Vigorous	• G4 = 0.97 (0.65-1.46)	
			Regular walking (min/d)	• G5 = 0.62 (0.37-1.05)	
			G1 = 0	Recreational activity	
			G2 = <20	• G1 = 1.00 (referent)	
			G3 = 21-40	• G2 = 0.95 (0.43-1.07)	
			G4 = 41-60	• G3 = 0.68 (0.43-1.07)	
			G5 = ≥ 60	• G4 = 0.34 (0.35-1.00)	
			Recreational activity, 4 groups	Sporting activity	
			G1 = Inactive/fairly Inactive	• G1 = 1.00 (referent)	
			G2 = Average 4 hr/weekend	• G2 = 0.50 (0.25-1.03)	
			G3 = Fairly active >4 h/weekend	• G3 = 0.88 (0.64-1.23)	
			G4 = Very active		
			Sporting activity, 3 Groups		
			G1 = None		
			G2 = Occasional		
			G3 = >1 time/month		

Paffenbarger et al 1986 [63]	To examine the PA and life-style characteristics of Harvard alumni for the relationship with all-cause mortality.	• n = 16,936	12-16 year follow-up (1962 to 1978)	• 1,413 deaths	The findings suggest a protective effect of exercise against all-cause mortality.
		• Sex: Men		Age adjusted RR (95% CI):	
		• Age: 35-74			
USA		• Characteristics: Harvard alumni	Records of freshman year physical examinations and records of intercollegiate sport	Those who walked	
Prospective cohort				• G1 = 1.00 (referent)	
				• G2 = 0.85	
				• G3 = 0.79	
D & B score = 14				Trend *p *= 0.0009	
			PA assessment: Mailed questionnaires surveying post college	Physical Activity Index (95% CI):	
			PA	• G1 = 1.00 (referent)	
				• G2 = 0.78	
				• G3 = 0.73	
				• G4 = 0.63	
			Exercise reported: Walking (miles/wk) 3	• G5 = 0.62	
			groups	• G6 = 0.52	
			G1 = <3	• G7 = 0.46	
			G2 = 3-8	• G8 = 0.62	
			G3 = ≥ 9		
				Trend *p *= <0.0001	
			PA index (kcal/wk) 3 groups:		
			G1 = <500		
			G2 = 500-999		
			G3 = 1000-1499		
			G4 = 1500-1999		
			G5 = 2000-2499		
			G6 = 2500-2999		
			G7 = 3000-3499		
			G8 = >3500		
			Cox proportional hazard models		

Schnohr et al 2007 [64]	To determine the impact of walking duration and intensity on all-cause mortality.	• n = 7,308 (3,204 male; 4,104 female)	Baseline and an average of 12 year	• 1,391 deaths	The findings indicate that the relative intensity and not duration of walking is the most important in relation to all-cause mortality.
Denmark		• Sex: Male and female	follow-up	Multivariate adjusted HR (95% CI):	
		• Age: 20-93 yr	PA assessment: Questionnaire, 4 durations and 3 intensities		
Prospective cohort		• Characteristics: Participants with no history of CHD, stroke or cancer and who had no difficulty in walking		Men	
D & B score = 12		• The Copenhagen City Heart Study		• G1 = 1.00 (referent)	
				• G2 = 0.38 (0.25-0.58)	
				• G3 = 0.38 (0.18-0.79)	
			Duration (hours/day)	• G4 = 0.69 (0.44-1.07)	
			1 = <0.5	• G5 = 0.37 (0.26-0.54)	
			2 = 0.5-1	• G6 = 0.33 (0.18-0.61)	
			3 = 1-2	• G7 = 0.78 (0.50-1.23)	
			4 = >2	• G8 = 0.41 (0.29-0.59)	
				• G9 = 0.33 (0.20-0.54)	
			Intensity	• G10 = 0.43 (0.22-0.82)	
			Slow intensity (SI)	• G11 = 0.42 (0.29-0.60)	
			Average intensity (AI)	• G12 = 0.28 (0.16-0.48)	
			Fast intensity (FI)		
				Women	
			12 groups	• G1 = 1.00 (referent)	
			G1 = 1 and SI	• G2 = 0.82 (0.52-1.29)	
			G2 = 1 and AI	• G3 = 0.78 (0.27-2.21)	
			G3 = 1 and FI	• G4 = 1.22 (0.82-1.81)	
			G4 = 2 and SI	• G5 = 0.74 (0.52-1.05)	
			G5 = 2 and AI	• G6 = 0.56 (0.33-0.96)	
			G6 = 2 and FI	• G7 = 0.94 (0.60-1.47)	
			G7 = 3 and SI	• G8 = 0.87 (0.61-1.23)	
			G8 = 3 and AI	• G9 = 0.48 (0.28-0.83)	
			G9 = 3 and FI	• G10 = 0.88 (0.40-1.88)	
			G10 = 4 and SI	• G11 = 0.64 (0.44-0.95)	
			G11 = 4 and AI	• G12 = 0.38 (0.21-0.69)	
			G12 = 4 and FI		

Kushi et al 1997 [65]	To evaluate the association between PA and all-cause mortality in postmenopausal women.	• n = 40,417	7 year follow-up	• 2,260 deaths	The results demonstrate a graded inverse association between PA and all-cause mortality in postmenopausal women.
		• Sex: Women			
		• Age: 55-69 yr	PA assessment: Questionnaire for frequency of moderate and vigorous LTPA	Multivariate adjusted Frequency of moderate PA per week RR (95% CI):	
USA		• Characteristics: Postmenopausal Iowa women			
Prospective cohort					
				• G1 = 1.00 (referent)	
				• G2 = 0.71 (0.63-0.79)	
D & B score = 13			Divided by frequency/week	• G3 = 0.63 (0.56-0.71)	
				• G4 = 0.59 (0.51-0.67)	
			G1 = Rarely/never	Trend p = <0.001	
			G2 = 1 time/week to a few times/month		
				Frequency of vigorous PA per week	
			G3 = 2-4 times/week		
			G4 = >4 times/week	• G1 = 1.00 (referent)	
				• G2 = 0.83 (0.69-0.99)	
				• G3 = 0.74 (0.59-0.93)	
			Activity index	• G4 = 0.62 (0.42-0.90)	
			G1 = Low	Trend p = 0.009	
			G2 = Medium		
			G3 = High		
				• G1 = 1.00 (referent)	
				• G2 = 0.77 (0.69-0.86)	
				• G3 = 0.68 (0.60-0.77)	
				Trend p = <0.001	

Paffenbarger et al 1993 [67]	To analyze changes in the lifestyles of Harvard College alumni and the association of these changes with mortality.	• n = 10,269	Baseline (1977) and 8 year follow-up (1985)	• 476 deaths	Beginning moderately vigorous sports activity was associated with lower rates of death from all causes among middle aged and older men.
		• Sex: Men			
		• Age: 45-84 yr (in 1977)		Beginning moderate sports activity was associated with 23% lower risk of death (95% CI 4%-42%, *p *= 0.015) than those not taking up moderate activity	
USA		• Characteristics: Participants with no reported life- threatening disease	PA Assessment: Questionnaire -- blocks walked daily, stairs climbed daily and type, frequency and duration of weekly sports and recreational activities		
Prospective cohort					
D & B score = 13					
			Physical activity index (kcal/wk)		
			Sports and recreational activities		
			Light <4.5 METs		
			Moderate >4.5 METs		
			Weekly lists of deaths were obtained from the Harvard college alumni office		
			Proportional hazard models with Poisson regression methods		

Katzmarzyk and Craig 2002 [154]	To quantify the relationship between musculoskeletal fitness and all-cause mortality.	• n = 8,116 (3,933 male; 4,183 female)	Baseline (1981) and	• 238 deaths	Some components of musculoskeletal fitness are predictive of mortality.
			13 year follow-up		
		• Sex: Men and women		RR (95% CI) adjusted for age, smoking status, body mass and VO_2max_	
Canada			Musculoskeletal fitness (sit ups, push ups, grip strength, sit and reach) measures divided into quartiles		
		• Age: 20-69 yr	Q1 = lowest	Sit ups	
Prospective cohort		• Characteristics: Participants who had musculoskeletal fitness measurements taken	Q2	Men	
			Q3	• Q1 = 2.72 (1.56-4.64)	
			Q4 = highest	• Q2 = 1.32 (0.73-2.41)	
D & B score = 11				• Q3 = 1.61 (0.90-2.87)	
				• Q4 = 1.00 (referent)	
		• Canadian Fitness Survey			
			Cox proportional hazard ratio model	Women	
				• Q1 = 2.26 (1.15-4.43)	
				• Q2 = 2.24 (1.07-4.67)	
				• Q3 = 1.27 (0.59-2.72)	
				• Q4 = 1.00 (referent)	
				Push-ups	
				Men	
				• Q1 = 1.25 (0.77-2.05)	
				• Q2 = 1.17 (0.71-1.90)	
				• Q3 = 0.94 (0.55-1.62)	
				• Q4 = 1.00 (referent)	
				Women	
				• Q1 = 0.61 (0.32-1.17)	
				• Q2 = 0.81 (0.45-1.47)	
				• Q3 = 0.87 (0.48-1.58)	
				• Q4 = 1.00 (referent)	
				Grip strength (kg)	
				Men	
				• Q1 = 1.49 (0.86-2.59)	
				• Q2 = 1.42 (0.82-2.45)	
				• Q3 = 1.59 (0.95-2.68)	
				• Q4 = 1.00 (referent)	
				Women	
				• Q1 = 1.08 (0.58-1.99)	
				• Q2 = 0.62 (0.44-1.56)	
				• Q3 = 1.25 (0.70-2.23)	
				• Q4 = 1.00 (referent)	
				Sit and reach (cm)	
				Men	
				• Q1 = 1.06 (0.64-1.74)	
				• Q2 = 1.01 (0.61-1.66)	
				• Q3 = 1.20 (0.74-1.95)	
				• Q4 = 1.00 (referent)	
				Women	
				• Q1 = 1.18 (0.66-2.10)	
				• Q2 = 1.07 (0.60-1.91)	
				• Q3 = 0.77 (0.44-1.46)	
				• Q4 = 1.00 (referent)	

Andersen et al 2000 [163]	To evaluate the relationship between levels of OPA, LTPA, cycling to work and sports participation and all-cause mortality.	• n = 30,640 (17,265 men; 13,375 women)	14.5 year follow-up	• 8,549 deaths	LTPA was inversely associated with all-cause mortality in both men and women in all age groups.
			PA assessment: Questionnaire for LTPA, divided into:	Incidence of all-cause mortality and PA	
Denmark		• Sex: Men and women			
Prospective cohort		• Age: 20-93 years (yr)		Multivariate adjusted RR (95% CI)	
			G1 = Low		
		• Characteristics: Participants of the Copenhagen City Heart Study, Glostrup Population Study and Copenhagen Male Study	G2 = Moderate		
D & B score = 13			G3 = High	Age 20-44 yr	
				Men	
				• G1 = 1.00 (referent)	
				• G2 = 0.73 (0.56-0.96)	
				• G3 = 0.74 (0.55-1.01)	
				Women	
				• G1 = 1.00 (referent)	
				• G2 = 0.75 (0.54-1.04)	
				• G3 = 0.66 (0.42-1.05)	
				Age 45-64 yr	
				Men	
				• G1 = 1.00 (referent)	
				• G2 = 0.75 (0.67-0.84)	
				• G3 = 0.75 (0.67-0.85)	
				Women	
				• G1 = 1.00 (referent)	
				• G2 = 0.73 (0.65-0.83)	
				• G3 = 0.66 (0.56-0.77)	
				Age >65 yr	
				Men	
				• G1 = 1.00 (referent)	
				• G2 = 0.62 (0.53-0.73)	
				• G3 = 0.60 (0.50-0.72)	
				Women	
				• G1 = 1.00 (referent)	
				• G2 = 0.52 (0.45-0.61)	
				• G3 = 0.49 (0.39-0.61)	
				All age groups	
				Men	
				• G1 = 1.00 (referent)	
				• G2 = 0.72 (0.66-0.78)	
				• G3 = 0.71 (0.65-0.78)	
				Women	
				• G1 = 1.00 (referent)	
				• G2 = 0.65 (0.60-0.71)	
				• G3 = 0.59 (0.52-0.67)	

Barengo et al 2004 [164]	To investigate whether moderate or high LTPA are associated with reduced CVD and all-cause mortality, independent of CVD risk factors and other forms of PA in men and women.	• n = 31,677 (15,853 men; 16,824 women)	20 year follow-up	HRR (95% CI)	Moderate and high levels of LTPA and OPA are associated with reduced premature all-cause mortality.
		• Sex: Men and women	PA assessment: Questionnaire self administered to measure OPA, LTPA and commuting activity	LTPA	
Finland		• Age: 30-59 yr		• 1.00 (referent) = low	
		• Characteristics: Participants from eastern and south-western Finland		• 0.91 (0.84-0.98) = mod, Men	
Prospective cohort					
				• 0.79 (0.70-0.90) = high, Men	
D & B score = 14				• 0.89 (0.81-0.98) = mod, women	
				• 0.98 (0.83-1.16) = high, women	
				OPA	
				• 1.00 (referent) = low	
				• 0.75 (0.68-0.83) = mod, men	
				• 0.77 (0.71-0.84) = active, men	
				• 0.79 (0.70-0.89) = mod, women	
				• 0.78 (0.70-0.87) = active, women	

Bath 2003 [165]	To examine differences between older men and women on the self-rated health mortality relationship.	• n = 1,042 (406 men; 636 women at baseline)	Baseline, 4 and 12 years post	Number of deaths: At 4 years 242 (106 men; 136 women)	The self-rated health-mortality relationship can be explained by health and related factors among older men and women.
UK		• Sex: Men and women		• At 12 years 665 (287 men; 378 women)	
Prospective cohort		• Age: >65 yr			
		• Characteristics: Community-dwelling Elderly	General physical health 14-item health index (Ebrahin et al 1987) scoring from 0-14 (no health problems -- multiple health problems)	Multivariate adjusted HR (95% CI)	
D & B score = 11					
		• The Nottingham Longitudinal Study of Activity and Ageing			
				Men after 4 years	
				• High = 1.00 (referent)	
				• Med = 1.19 (0.61-2.33)	
			PA assessment: Self-rated health surveys, divided into 3 levels of PA:	• Low = 1.51 (0.75-3.03)	
			High	Women after 4 years	
			Medium	• High = 1.00 (referent)	
			Low	• Med = 1.03 (0.58-1.82)	
				• Low = 1.51 (0.86-2.67)	
				Men after 12 years	
			Cox proportional hazards regression Models	• High = 1.00 (referent)	
				• Med = 1.28 (0.94-1.74)	
				• Low = 1.13 (0.82-1.55)	
				Women after 12 years	
				• High = 1.00 (referent)	
				• Med = 1.20 (0.90-1.61)	
				• Low = 1.23 (0.93-1.62)	

Bijnen et al 1998 [166]	To describe the association between PA and mortality (CVD, stroke, all-cause) in elderly men.	• n = 802	10 year follow-up	• 373 deaths	PA may protect against all- cause mortality in elderly men
		• Sex: Men			
		• Age: 64-84 yr	PA assessment: Questionnaire, divided into groups:	Multivariate adjusted RR (95% CI)	
Netherlands		• Characteristics: Retired Dutch men			
				• G1 = 1.00 (referent)	
Prospective cohort			G1 = Lowest	• G2 = 0.80 (0.63-1.02)	
			G2 = Middle	• G3 = 0.77 (0.59-1.00)	
			G3 = Highest	*p *= 0.04	
D & B score = 12					

Blair et al 1993 [167]	To evaluate the relationship of sedentary living habits to all-cause mortality in women.	• n = 3,120	Baseline and 8 year follow-up	• 43 deaths	There is a graded inverse relationship between PF and all-cause mortality in women.
		• Sex: Women			
		• Age: Not available		Age adjusted death rates (per 10,000 person years) by fitness	
USA		• Characteristics: Participants were given a preventative medicine examination	PF assessment: PF measured via maximal treadmill exercise test;		
Prospective				• Low Fitness = 40	The lack of relationship between PA and death rate was believed to be due to an inadequate assessment of PA.
				• Mod Fitness = 16	
D & B score = 14				• High Fitness = 7	
			PA assessment: Questionnaire		
				No difference between levels of PA	

Blair et al 1996 [168]	To review the association of PF to all-cause and CVD mortality.	• n = 32,421 (25,341 men; 7,080 women)	Baseline and average 8 year follow-up (range 0.1-19.1 years)	• 601 deaths in men	The study observed a steep inverse gradient of death rates across low, moderate and high PF levels. The association was strong and remained after adjustment for potential confounding factors.
				• 89 deaths in women	
		• Sex: Men and women			
USA		• Age: 20-80 yr (mean 43 yr)		RR (95% CI) in low PF vs.	
			PF assessment: Treadmill test; duration was used to assign participants to sex specific groups:	high PF	
Prospective cohort		• Characteristics: Participants were excluded if they did not reach 85% of their age predicted maximal heart rate on the maximal exercise treadmill test		Men	
				• 1.52 (1.28-1.82)	
				Women	
D & B score = 14				• 2.10 (1.36-3.26)	
			Low (least fit 20%)	Adjusted deaths per 10,000 person years according to PF	
			Moderate (next 40%)		
			High (most fit 40%)	Men	
		• Aerobics Center Longitudinal Study	Proportional hazard modeling	• Low = 49	
				• Med = 27	
				• High = 23	
				Women	
				• Low = 29	
				• Med = 13	
				• High = 14	

Boyle et al 2007 [169]	To examine the association between PA and the risk of incident disability, including impairment in activities of daily living and instrumental activities of daily living in community based older persons free from dementia.	• n = 1,020	2.6 year follow-up	• 156 deaths	The risk of death decreased 11% with each hour of PA/wk.
		• Sex: Men and women			
		• Age: 54-100 yr	PA assessment: Questionnaire, hr/wk of PA Incidence of all-cause mortality	HR for all-cause mortality	
USA		• Characteristics: Participants from 40 retirement communities across Chicago		The risk of death was 11% lower for each hr/wk of PA	
Prospective cohort					
D & B score = 13		• Rush Memory and Aging Project			

Bucksch et al 2005 [170]	To examine the effect of moderately intense PA on all-cause mortality.	• n = 7,187 (3,742 men; 3,445 women)	Baseline (1984-1986) and 12-14 yr follow-up (1998)	• 943 deaths	Participants who achieved recommended amounts of MPA or VPA were at a significantly lower risk of death than their sedentary counterparts.
		• Sex: Men and women		RR (95% CI) for achieving recommended PA vs. not achieving recommendation	
Germany		• Age: 30-69 yr			
Prospective cohort		• Characteristics: Participants were healthy and physically active during leisure time	PA assessment: Questionnaire (Minnesota Leisure Time Physical Activity questionnaire) divided into groups based on: Achieving recommended amount of MPA (30 min, 5 d/wk (≥2.5 h/wk))		
				Women	
				• MPA = 0.65 (0.51-0.82)	
D & B score = 13				• VPA = 0.78 (0.57-1.08)	
				• MPA or VPA = 0.60 (0.47-0.75)	
				Men	
				• MPA = 0.90 (0.77-1.01)	
				• VPA = 0.74 (0.61-0.90)	
				• MPA or VPA = 0.80 (0.68-0.94)	
			Achieving recommended amount of VPA (20 min, 3 d/wk (≥ 1 h/wk))		
				RR (95% CI) for volume of lifestyle activities (kcal/kg/wk)	
			Volume of lifestyle activities (kcal/kg/wk)	Women	
			G1 = 0	• G1 = 1.00 (referent)	
			G2 = <14	• G2 = 0.79 (0.57-1.08)	
			G3 = 14-33.5	• G3 = 0.68 (0.50-0.94)	
			G4 = ≥ 33.5	• G4 = 0.57 (0.41-0.79)	
				*p *< 0.001	
				Men	
				• G1 = 1.00 (referent)	
				• G2 = 0.98 (0.76-1.17)	
				• G3 = 0.80 (0.63-1.00)	
				• G4 = 0.91 (0.74-1.13)	
				*p *= 0.20	
				Adjusted for age, other recommendation, social class, smoking, BMI, cardio risk factor index, alcohol intake, chronic disease index and dietary factors	

Bucksch and Helmert 2004 [171]	To examine LTPA and premature death in the general population of former West Germany.	• n = 7,187 (3,742 men; 3,445 women)	Baseline (1984-1986) and 12-14 year follow-up (1998)	• 943 deaths	LTPA is inversely associated with all-cause mortality in men and women.
		• Sex: Men and women		RR (95% CI)	
		• Age: 30-69 yr		Men, LTPA	
Germany		• Characteristics: Participants were selected on the basis of the German Cardiovascular Prevention Study	PA assessment: Questionnaire (Minnesota Leisure Time Physical Activity questionnaire) divided into groups based on: LTSA (h/wk)	• G1 = 1.00 (referent)	
				• G2 = 0.85 (0.78-0.93)	
Prospective cohort				• G3 = 0.64 (0.50-0.82)	
				• G4 = 0.70 (0.54-0.91)	
				*p *< 0.001	
D & B score = 14		• The National Health Survey of the German Federal Institute of Population Research (1984-1998)		Men, LTPA index	
			G1 = 0	• G1 = 1.00 (referent)	
			G2 = <1	• G2 = 0.92 (0.70-1.23)	
			G3 = 1-2	• G3 = 0.89 (0.69-1.17)	
			G4 = >2	• G4 = 0.61 (0.44-0.84)	
				*p *<0.01	
			The LTSA-index (kcal/kg/wk)		
			G1 = 0	Women, LTPA	
			G2 = 1-10	• G1 = 1.00 (referent)	
			G3 = 10-25	• G2 = 0.93 (0.82-1.04)	
			G4 = >25	• G3 = 0.69 (0.48-0.98)	
				• G4 = 0.57 (0.35-0.94)	
			Mortality -- Records from the mandatory population registries	*p *< 0.01	
				Women, LTPA index	
				• G1 = 1.00 (referent)	
			Cox proportional hazard regression model	• G2 = 0.68 (0.45-1.01)	
				• G3 = 0.79 (0.51-1.21)	
				• G4 = 0.46 (0.25-0.85)	
				*p *< 0.01	
				Adjusted for age, social class, smoking, BMI, cardio risk factor index, alcohol intake, chronic disease index and dietary factors	

Carlsson et al 2006 [172]	To investigate the association between PA and mortality in post-menopausal women.	• n = 27,734	Baseline (1997) and 2-7 year follow-up (1999-2004)	• 1,232 deaths	The study indicates that even fairly small amounts of activity will reduce mortality in older women.
		• Sex: Women			
		• Age: 51-83 yr		RR (95% CI) adjusted for lifestyle and medical problems	
Sweden		• Characteristics: Women who participated in a population based Screening programme in 1987			
Prospective cohort			PA assessment: Questionnaires for: METs/day, different PA (walking/biking), LTPA, OPA, household PA, TV watching and reading		
				PA (METs/day)	
				• >50 = 1.00 (referent)	
D & B score = 12				• 45-50 = 1.05 (0.77-1.42)	
		• The Swedish Mammography Cohort		• 40-45 s = 1.09 (0.81-1.46)	
				• 45-40 = 1.26 (0.94-1.70)	
				• <35 = 2.56 (1.85-3.53)	
			Mortality -- Records from the National Population Register		
				Different PA	
				Walking/biking (min/d)	
				• > 90 = 1.00 (referent)	
				• 60-90 = 1.01 (0.76-1.34)	
				• 40-60 = 0.92 (0.70-1.20)	
				• 20-40 = 0.96 (0.75-1.23)	
				• <20 = 1.16 (0.90-1.50)	
				• Almost never = 1.94 (1.51-2.50)	
				LTPA (hr/wk)	
				• >5 = 1.00 (referent)	
				• 4-5 = 0.95 (0.74-1.22)	
				• 2-3 = 1.02 (0.83-1.26)	
				• 1 = 1.09 (0.88-1.36)	
				• <1 = 1.91 (1.56-2.35)	
				OPA	
				• Heavy manual labour = 1.00 (referent)	
				• Walking/lifting/ a lot carrying = 0.96 (0.55-1.70)	
				• Walking/lifting/ not a lot carrying = 1.00 (0.60-1.68)	
				• Mostly standing = 0.91 (0.52-1.61)	
				• Seated 50% of time = 0.97 (0.58-1.62)	
				• Mostly sedentary = 1.93 (1.15-3.25)	
				Household work (hr/d)	
				• >8 h/d = 1.00 (referent)	
				• 7-8 = 0.68 (0.49-0.93)	
				• 5-6 = 0.66 (0.51-0.87)	
				• 3-4 = 0.83 (0.64-1.06)	
				• 1-2 = 0.89 (0.69-1.15)	
				• <1 = 1.73 (1.30-2.32)	
				Adjusted for age	

Crespo et al 2002 [173]	To study the relationship between PA and obesity with all- cause mortality in Puerto Rican men.	• n = 9,136 (1962-1965)	Baseline and 12 year follow-up	• 1,445 deaths	Some PA is better than none in protecting against all-cause mortality. The benefits are independent of body weight.
Puerto Rico		• Sex: Men	PA assessment: Questionnaire, divided into 4 groups based on METs	Multivariate OR (95% CI) adjusted for age	
			G1 = low		
			G2		
			G3		
			G4 = high		
Prospective cohort		• Age: 35-79 yr	Multivariate logistic function model	• C1 = 1.00 (referent)	
D & B score = 12		• Characteristics: Participants with no known coronary heart disease		• C2 = 0.67 (0.57-0.78)	
		• The Puerto Rico Heart Health Program		• C3 = 0.63 (0.54-0.74)	
				• C4 = 0.54 (0.46-0.64)	
				*p *< 0.0001	
				Multivariate adjusted OR (95% CI)	
				• C1 = 1.00 (referent)	
				• C2 = 0.68 (0.58-0.79)	
				• C3 = 0.63 (0.54-0.75)	
				• C4 = 0.55 (0.46-0.65)	
				*p *< 0.0001	

Davey Smith et al 2000 [174]	To examine the relationship of PA and various causes of death.	• n = 6,702 (at baseline)	Baseline (1969-1970) and 25 year follow-up	• 926 deaths	In the study, an inverse association of both LTPA and walking pace with mortality from all-causes was seen.
UK		• Sex: Men	PA assessment: Questionnaire with 3 groups for walking pace (Slower, same, faster) and 3 groups for LTPA (inactive, moderately active, active)	Age adjusted RR (95% CI) for walking pace	
Prospective cohort		• Age: 40-64 yr		• Slower = 2.47 (2.2-2.8)	
D & B score = 13		• Characteristics: Participants from rural northern Japan		• Same = 1.35 (1.2-1.5)	
		• Whitehall study		• Faster = 1.00 (referent) *p *< 0.001	
				Fully adjusted RR (95% CI) for walking pace	
				• Slower = 1.87 (1.6-2.1)	
				• Same = 1.21 (1.1-1.3)	
				• Faster = 1.00 (referent) *p *< 0.001	
				Age adjusted RR (95% CI) for LTPA	
				• Inactive = 1.44 (1.3-1.6)	
				• Mod = 1.13 (1.0-1.2)	
				• Active = 1.00 (referent) *p *< 0.001	
				Fully adjusted RR (95% CI) for LTPA	
				• Inactive = 1.20 (1.1-1.3)	
				• Mod = 1.07 (1.0-1.2)	
				• Active = 1.00 (referent) *p *< 0.001	

Eaton et al 1995 [175]	To determine whether self-reported PA predicts a decreased rate of CHD and all- cause mortality in middle aged men.	• n = 8,463	21 year follow-up	• 2,593 deaths	Baseline levels of self- reported LTPA predicted a decreased rate of CHD and all-cause mortality.
Europe, Israel, mid eastern Asia, Northern Africa		• Sex: Men	PA assessment: Questionnaire for LTPA	Age adjusted RR (95% CI) LTPA	
Prospective cohort		• Age: ≥40 yr	G1 = Sedentary	• G1 = 1.00 (referent)	
D & B score = 12		• Characteristics: Government employees without known CVD	G2 = Light	• G2 = 0.84 (0.74-0.94)	
			G3 = Light daily	• G3 = 0.81 (0.73-0.90)	
			G4 = Heavy	• G4 = 0.84 (0.72-0.98)	
				OPA	
			Questionnaire for OPA	• G1 = 1.00 (referent)	
			G1 = Sitting	• G2 = 0.99 (0.88-1.12)	
			G2 = Standing	• G3 = 1.09 (0.99-1.20)	
			G3 = Walking	• G4 = 1.16 (1.03-1.30)	
			G4 = Physical labour		

Fang et al 2005 [176]	To assess the association of exercise and CVD outcome among persons with different blood pressure status.	• n = 9,791 (3,819 men; 5,972 women)	17 year follow-up	Incidence of all-cause mortality and PA	A significant effect of exercise on mortality in normotensive subjects was not found.
USA		• Sex: Men and women	PA assessment: Questionnaire with 3 groups	Multivariate adjusted HR (95% CI)	
Prospective cohort		• Age:25-74 yr	G1 = Least exercise	• G1 = 1.00 (referent)	
D & B score = 12		• Characteristics: Non- institutionalized participants	G2 = Moderate exercise	• G2 = 0.75 (0.53-1.05)	
			G3 = Most exercise	• G3 = 0.71 (0.45-1.12)	

Fried et al 1998 [177]	To determine the disease, functional and personal characteristics that jointly predict mortality.	• n = 5,886	5 year follow-up	• 646 deaths	PA was a predictor of 5-year mortality.
USA		• Sex: Men and women	PA assessment: Self reported exercise (5 groups)	Incidence of all-cause mortality and PA	
Prospective cohort		• Age: ≥65 yr	MPA or VPA (kJ/wk)	Multivariate adjusted RR (95% CI)	
D & B score = 11		• Characteristics: Community dwelling elders	G1 = ≤282	• G1 = 1.00 (referent)	
			G2 = 283-1789	• G2 = 0.78 (0.60-1.00)	
			G3 = 1790-4100	• G3 = 0.81 (0.63-1.05)	
			G4 = 4101-7908	• G4 = 0.72 (0.55-0.93)	
			G5 = >7908	• G5 = 0.56 (0.43-0.74) *p *< 0.005	

Fujita et al 2004 [178]	To examine the relationship between walking duration and all-cause mortality in a Japanese cohort.	• n = 41,163 (20,004 men; 21,159 women)	Baseline (1990) and 11 year follow-up (2001)	• 1,879 deaths	Time spent walking was associated with a reduced risk for all-cause mortality.
Japan		• Sex: Men and women	PA assessment: Questionnaire Walking, 3 levels:	Age and sex adjusted RR (95% CI) for time spent walking (hr/d)	
			G1 = ≤30 min		
			G2 = 30 min to 1 hr		
			G3 = ≥1 hr		
Prospective cohort		• Age: 40-64 yr	Cox proportional hazard model	Whole group	
D & B score = 13		• Characteristics: Healthy, sedentary		• G1 = 1.22 (1.09-1.35)	
				• G2 = 1.09 (0.95-1.22)	
				• G3 = 1.00 (referent) *p *< 0.001	
				Men only	
				• G1 = 1.14 (1.00-1.30)	
				• G2 = 1.03 (0.90-1.19)	
				• G3 = 1.00 (referent *p *= 0.061	
				Women only	
				• G1 = 1.40 (1.16-1.68)	
				• G2 = 1.23 (1.01-1.49)	
				• G3 = 1.00 (referent) *p *< 0.001	
				RR (95% CI) for time spent walking (hr/d) (adjusted for age, education, marital status, past history of diseases, smoking, drinking, BMI and dietary variables)	
				Whole group	
				• G1 = 1.17 (1.04-1.31)	
				• G2 = 1.06 (0.93-1.20)	
				• G3 = 1.00 (referent) *p *= 0.011	
				Men	
				• G1 = 1.08 (0.94-1.25)	
				• G2 = 0.98 (0.84-1.14)	
				• G3 = 1.00 (referent) *p *= 0.318	
				Women	
				• G1 = 1.38 (1.12-1.70)	
				• G2 = 1.24 (1.00-1.54)	
				• G3 = 1.00 (referent) *p *< 0.001	

Glass et al 1999 [179]	To examine any association between social activity, productive activity and PA and mortality in older people.	• n = 2,761 (1,169 men; 1,143 women)	13 year follow-up	Incidence of all-cause mortality by fitness activity quartile	More active elderly people were less likely to die than those who were less active.
USA		• Sex: Men and women	PA assessment: Interview, Amount of activity	13 yr mortality by amount of activity	
Prospective cohort		• Age: ≥ 65 yr	G1 = Low	• G1 = 74.0	
D & B score = 12		• Characteristics: Healthy elders	G2 = Low-medium	• G2 = 69.8	
			G3 = Medium-high	• G3 = 62.4	
			G4 = High	• G4 = 55.2	

Gulati et al 2003 [180]	To determine whether exercise capacity is a predictor for all-cause mortality in asymptomatic women.	• n = 5,721	Baseline (1992) and 8 year follow-up (2000)	• 180 deaths	This study confirmed that exercise capacity is an independent predictor of death in asymptomatic women, greater than what has been previously established among men.
USA		• Sex: Women	PF Assessment: Treadmill stress test Exercise capacity (METs) G1 = <5 G2 = 5-8 G3 = >8	For every 1 MET increase there was a reduced death risk of 17% (*p *< 0.001)	
Prospective cohort		• Age: Mean 52 ± 11 yr		Age-adjusted RR	
D & B score = 11		• Characteristics: Asymptomatic women		• G1 = 2.0 (1.3-3.2)	
		• St James Women Take Heart Project		• G2 = 1.6 (1.1-2.4)	
				• G3 = 1.00 (referent)	
				Adjusted for Framingham Risk Score	
				• G1 = 3.1 (2.1-4.8)	
				• G2 = 1.9 (1.3-2.9)	
				• G3 = 1.00 (referent)	

Haapanen et al 1996 [181]	To examine the association between LTPA and all-cause mortality.	• n = 1,072	Baseline and a 10 yr 10 month follow-up	• 168 deaths	Low PA is a risk factor for all-cause mortality.
Finland		• Sex: Men	PA assessment: Self-reported LTPA, divided into 4 groups by EE (kJ/wk) G1 = 0-3349 G2 = 3350-6279 G3 = 6280-8791 G4 = >8791	RR (95% CI) according to EE group	
Prospective cohort		• Age: 35-63 yr	Mortality--National Death Index search	• G1 = 2.74 (1.46-5.14)	
D & B score = 14		• Characteristics: Healthy, sedentary	Cox proportional HR	• G2 = 1.10 (0.55-2.21)	
				• G3 = 1.74 (0.87-3.50)	
				• G4 = 1.00 (referent)	

Hakim et al 1998 [182]	To examine the association between walking and mortality in retired men.	• n = 707	Baseline and 12 yr follow-up	• 208 deaths	The findings in older physically capable men indicate that regular walking is associated with a lower overall mortality rate.
USA		• Sex: Men		RR (95% CI) according to distance walked	
Prospective cohort		• Age: 61-81 yr		Adjusted for age	
D & B score = 12		• Characteristics: Retired non-smoking men who were physically capable of participating in low intensity activities on a daily basis	PA assessment: Questionnaire Distance walked (miles/day)	• G1 vs. G3 = 1.9 (1.3-2.9)	
			G1 = 0.0-0.9	• G1 vs. G3 = 1.6 (1.2-2.2)	
			G2 = 1.0-2.0	• G2 vs. G3 = 1.2 (0.8-1.7)	
			G3 = 2.1-8.0	Trend *p *= 0.002	
		• Honolulu Heart Program			
				Adjusted for risk factors	
				• G1 vs. G3 = 1.8 (1.2-2.7)	
				• G1 vs. G2 = 1.5 (1.1-2.1)	
				• G2 vs. G3 = 1.1 (0.8-1.7)	
				Trend *p *= 0.01	

Hillsdon et al 2004 [183]	To examine whether VPA is associated with all-cause mortality.	• n = 10,522 (4,929 men; 5,593 women)	>10 year follow-up	• 825 deaths	Questionnaire respondents who reported engaging in VPA less than twice a week experienced a 37% reduced risk of all-cause mortality compared with respondents who reported a lower frequency of VPA.
		• Sex: Men and women	PA assessment: Questionnaire for frequency of VPA	Age and sex adjusted RR (95% CI)	
UK		• Age: 35-64 yr	G1 = Never, <1 time/month		
		• Characteristics: Healthy, sedentary	G2 = <2 times/wk	• G1 = 1.00 (referent)	
Prospective Cohort		• OXCHECK study	G3 = >2 times/wk	• G2 = 0.57 (0.42-0.79)	
				• G3 = 0.72 (0.54-0.95)	
D & B score = 11				Fully adjusted RR (95% CI)	
				• G1 = 1.00 (referent)	
			Mortality -- Recorded from the Office of National Statistics	• G2 = 0.63 (0.45-0.89)	
				• G3 = 0.81 (0.60-1.09)	
			Cox proportional HR		

Hu et al 2005 [184]	To examine the association of PA and BMI and their combined effect with the risk of total, CVD and cancer mortality.	• n = 47,212 (22,528 men; 24,684 women)	17.7 year follow-up	• 7,394 deaths	Regular PA is an important indicator for decreased risk of all-cause mortality. PA has a strong independent effect on mortality.
		• Sex: Men and women			
Finland		• Age:25-64 yr	PA assessment: Questionnaire for PA level, divided into 3 groups	Adjusted HR (95% CI)	
		• Characteristics: Participants from eastern Finland		Men	
Prospective cohort				• G1 = 1.00 (referent)	
				• G2 = 0.74 (0.68-0.81)	
			G1 = Low	• G3 = 0.63 (0.58-0.70)	
D & B score = 12			G2 = Moderate	Trend *p *= <0.001	
			G3 = High		
				Women	
				• G1 = 1.00 (referent)	
				• G2 = 0.64 (0.58-0.70)	
				• G3 = 0.58 (0.52-0.64)	
				Trend *p *= <0.001	

Hu et al 2004 [185]	To examine the association of BMI and PA with death.	• n = 116,564	Baseline (1976) and	• 10,282 deaths	Reduced PA is a strong and independent predictor of death.
		• Sex: Women	24 year follow-up		
		• Age: 30-55 yr		Multivariate RR (95% CI) by PA (hr/wk)	
USA		• Characteristics: Females free of known CVD and cancer	PA assessment: Questionnaire for PA level, divided into 3 groups (hr/week)	• G1 = 1.00 (referent)	
			G1 = ≥ 3.5	• G2 = 1.18 (1.10-1.26)	
Prospective cohort			G2 = 1.0-3.4	• G3 = 1.52 (1.41-1.63)	
D & B score = 11			G3 = <1.0	Multivariate RR (95% CI) by PA adjusted for BMI	
				• G1 = 1.00 (referent)	
			BMI (kg/m^2^)	• G2 = 1.14 (1.06-1.22)	
			G1 = <25	• G3 = 1.44 (1.34-1.55)	
			G2 = 25-29		
			G3 = 30		
			Cox proportional HR		

Kampert et al 1996 [186]	To examine PF and PA in relation to all-cause and cancer mortality.	• n = 32,421 (25,341 men; 7,080 women)	Baseline (1970) and ~8 year follow-up (1989)	• 690 deaths	The data support the hypothesis that an active and fit way of life delays death.
		• Sex: Men and women		Adjusted RR (95% CI) by quintiles of activity	
USA		• Age: 20-88 yr (mean ~43)			
Prospective cohort		• Characteristics: Predominantly white and from the middle and upper socioeconomic strata	PA assessment: Questionnaire, divided into quintiles of activity (min/wk)	Men	
				• Sedentary = 1.00 (referent)	
				• C1-2 = 0.71 (0.58-0.97)	
D & B score = 13				• C3 = 0.83 (0.59-1.16)	
			Male activity categories	• C4 = 0.57 (0.30-1.08)	
				• C5 = 0.92 (0.29-2.88)	
			Sedentary = 855	Trend *p *= 0.011	
			C1-2 = 1,072		
			C3 = 1,292	Women	
			C4 = 1,453	• Sedentary = 1.00 (referent)	
			C5 = 1,601	• C1-2 = 0.68 (0.39-1.17)	
				• C3 = 0.39 (0.09-1.65)	
			Females activity categories	• C4-5 = 1.14 (0.27-4.80)	
			Sedentary = 605	Trend *p *= 0.217	
			C1-2 = 792		
			C3 = 979		
			C4-5 = 1,158		
			Cox proportional HR		

Kaplan et al 1996 [187]	To assess LTPA and its association with all cause mortality.	• n = 6,131 (3298 men; 2833 women)	28 year follow-up	• 1,226 deaths	The data provide further support for the importance of PA and indicate that the protective effect of PA is a robust one.
		• Sex: Men and women	PA assessment: Three questions about PA, with scores 0 (never), 2 (sometimes) or 4 (often).	Incidence of all-cause mortality and PA	
USA		• Age: 16-94 yr			
		• Characteristics: Northern Californian adults			
Prospective cohort				Death rates/1000 person years	
				Men	
D & B score = 13				• T1 = 24.68	
			Tertiles of PA score	• T2 = 11.37	
			T1 = 0-2	• T3 = 7.59	
			T2 = 4-6	Women	
			T3 = 8-12	• T1 = 18.03	
				• T2 = 7.66	
				• T3 = 3.88	

Khaw et al 2006 [188]	To examine the relationship between PA patterns over 1 year and total mortality.	• n = 22,191 (9,984 men; 12,207 women)	8 year follow-up	• 1,553 deaths	Even very moderate levels of usual PA are associated with reductions in mortality.
		• Sex: Men and women	PA assessment: Questionnaire, divided into 4 groups of PA	Incidence of all-cause mortality and PA	
UK		• Age: 45-79 yr		Adjusted RR (95% CI)	
		• Characteristics: Community living participants		All	
Prospective cohort			G1 = Inactive	• G1 = 1.00 (referent)	
			G2 = Moderately inactive	• G2 = 0.83 (0.73-0.95)	
D & B score = 13				• G3 = 0.68 (0.58-0.80)	
			G3 = Moderately active	• G4 = 0.68 (0.57-0.81)	
			G4 = Active	Age <65	
				• G1 = 1.00 (referent)	
				• G2 = 1.01 (0.78-1.31)	
				• G3 = 0.81 (0.62-1.07)	
				• G4 = 0.82 (0.62-1.09)	
				Age >65	
				• G1 = 1.00 (referent)	
				• G2 = 0.77 (0.66-0.91)	
				• G3 = 0.65 (0.53-0.79)	
				• G4 = 0.64 (0.50-0.80)	

Kohl et al 1996 [189]	To determine the association of maximal exercise hemodynamic responses with risk of all-cause mortality.	• n = 26,621 (20,387 men; 6,234 women)	Average 8.1 year follow-up	• 348 deaths in men and 66 in women	The results suggest an exaggerated SBP or an attenuated heart rate response to maximal exercise may indicate an elevated risk for mortality.
		• Sex: Men and women			
USA		• Age: Male mean 42.2 yr; female mean 41.9 Yr		Adjusted RH (95% CI) by maximal exercise test HR	
Prospective cohort				Men	
		• Characteristics: Apparently healthy patients of a preventive medicine centre	PF assessment: Maximal exercise test HR (bpm), divided into 4 Groups:	• Q1 = 1.00 (referent)	
			G1 = <171	• Q2 = 0.61 (0.44-0.85)	
D & B score = 12			G2 = 171-178	• Q3 = 0.69 (0.51-0.93)	
			G3 = 179-188	• Q4 = 0.60 (0.41-0.87)	
			G4 = >188	Trend *p*<0.05	
				Women	
				• Q1 = 1.00 (referent)	
				• Q2 = 1.23 (0.65-2.32)	
				• Q3 = 0.69 (0.30-1.63)	
				• Q4 = 0.71 (0.22-2.24)	
				Trend *p*>0.05	

Kujala et al 1998 [190]	To investigate LTPA and mortality in a cohort of twins.	• n = 15,902 (7,925 men; 7,977 women)	Baseline 1975 and death outcome from 1977-1994	• 1,253 deaths	LTPA is associated with reduced mortality, even after genetic and other familial factors are taken into account.
		• Sex: Men and women		HR (95% CI)	
Finland		• Age: 25-64 yr			
		• Characteristics: Healthy, Finnish same sex twins	PA assessment: Questionnaire, quintiles of fitness in MET hours/day	Adjusted for age and sex	
Prospective cohort				• Sedentary = 1.00 (referent)	
				• OE = 0.71 (0.62-0.81)	
		• The Finnish Twin Cohort		• CE = 0.57 (0.45-0.74)	
D & B score = 13			Q1 = <58	Trend *p *= 0.001	
			Q2 = 59-1.29		
			Q3 = 1.30-2.49	Adjusted for age, sex, smoking	
			Q4 = 2.50-4.49		
			Q5 = >4.50	• Sedentary = 1.00 (referent)	
				• OE = 0.76 (0.67-0.87)	
			Categorized into:	• CE = 0.68 (0.53-0.88)	
			-Sedentary		
			-Occasional exerciser (OE)	Trend *p *= 0.001	
			-Conditioning exerciser (CE)	Adjusted for age, sex, smoking, occupational group, alcohol	
				• Sedentary = 1.00 (referent)	
				• OE = 0.80 (0.69-0.91)	
				• CE = 0.76 (0.59-0.98)	
				Trend *p *= 0.002	
				HR (95% CI) among 434 same sex twin pairs compared with sedentary category in 1975	
					
				• Sedentary = 1.00 (referent)	
				• OE = 0.66 (0.46-0.94)	
				• CE = 0.44 (0.23-0.83)	
				Trend *p *= 0.005	
				Adjusted for smoking	
				• Sedentary = 1.00 (referent)	
				• OE = 0.70 (0.48-1.01)	
				• CE = 0.56 (0.29-1.09)	
				Trend *p *= 0.04	
				Adjusted for smoking, occupational group, alcohol	
				• Sedentary = 1.00 (referent)	
				• OE = 0.73 (0.50-1.07)	
				• CE = 0.56 (0.29-1.11)	
				Trend *p *= 0.06	
				OR (95% CI) in quintiles among 434 same sex twin pairs compared with sedentary category in 1975	
				• Q1 = 1.00 (referent)	
				• Q2 = 0.85	
				• Q3 = 0.72	
				• Q4 = 0.68	
				• Q5 = 0.60	

LaCroix et al 1996 [191]	To determine whether walking is associated with a reduced risk of CVD hospitalization and death in older adults.	• n = 1,645 (615 men; 1030 women)	4.2 year follow-up	RR (95% CI) by category of walking	Walking more than 4 hr/wk was associated with a reduced risk of mortality from all-causes.
		• Sex: Men and women	PA assessment: Questionnaire for walking h/wk, divided into 3 groups		
USA		• Age: ≥65 yr	G1 = <1 hr/week	Men	
		Characteristics: Participants from a group health co-operative	G2 = 1-4 hr/week	• G1 = 1.00 (referent)	
Prospective cohort			G3 = >4 hr/week	• G2 = 0.78 (0.43-1.45)	
				• G3 = 0.89 (0.49-1.62)	
D & B score = 12				Women	
				• G1 = 1.00 (referent)	
				• G2 = 0.50 (0.28-0.90)	
				• G3 = 0.48 (0.25-0.83)	
				Age 65-74 yr	
				• G1 = 1.00 (referent)	
				• G2 = 0.81 (0.40-1.61)	
				• G3 = 1.13 (0.60-2.15)	
				Age ≥75 yr	
				• G1 = 1.00 (referent)	
				• G2 = 0.63 (0.37-1.08)	
				• G3 = 0.46 (0.25-0.84)	
				High functioning	
				• G1 = 1.00 (referent)	
				• G2 = 0.73 (0.38-1.41)	
				• G3 = 0.89 (0.48-1.65)	
				Limited functioning	
				• G1 = 1.00 (referent)	
				• G2 = 0.60 (0.34-1.05)	
				• G3 = 0.51 (0.28-0.92)	

Lam et al 2004 [192]	To investigate the relationship LTPA and mortality in Hong Kong.	• n = 24,079 cases (13,778 men; 10,301 women);	10 years prior	Multivariate adjusted OR (95% CI) by LTPA	The data confirm and extend previous findings in Caucasian populations on the association between LTPA and longevity.
			PA assessment:	Men	
Hong Kong		• n = 13,054 controls (3,918 men; 9,136 women)	Questionnaire for LTPA, divided into 3 groups	• G1 = 1.00 (referent)	
				• G2 = 0.60 (0.54-0.67)	
Case-Control				• G3 = 0.66 (0.60-0.73)	
		• Sex: Men and women	G1 = <1 times per month		
D & B score = 12		• Age: ≥35 yr		Women	
		• Characteristics: All ethnic Chinese	G2 = 1-3 times per month	• G1 = 1.00 (referent)	
				• G2 = 0.81 (0.74-0.88)	
			G3 = ≥4 times per month	• G3 = 0.71 (0.66-.077)	

Lan et al 2006 [193]	To investigate the relationship between exercise and all-cause mortality.	• n = 2,113 (1,081 men; 1,032 women)	Baseline and 2 year follow-up	• 197 deaths	Older persons are recommended to expend at least 1000 kcal/wk through regular exercise for mortality reduction.
		• Sex: Men and women		HR (95% CI) by LTPA frequency	
Taiwan		• Age: ≥65 yr	PA assessment: Questionnaire for LTPA (frequency/wk)		
Prospective cohort		• Characteristics: Non-institutionalized elders		Adjusted for age and sex	Protection of exercise against death also increases with the number of activities.
			G1 = Sedentary	• G1 = 1.00 (referent)	
		• Taiwan National Health Interview Survey	G2 = 1 time/wk	• G2 = 0.49 (0.36-0.67)	
D & B score = 13			G3 = ≥2 times/wk	• G3 = 0.20 (0.09-0.46)	
				Trend *p *= <0.001	
			Questionnaire for EE (kcal/wk), divided into 5 groups:	Multivariate adjusted	
				• G1 = 1.00 (referent)	
			G1 = Sedentary	• G2 = 0.70 (0.50-0.98)	
			G2 = <500	• G3 = 0.35 (0.15-0.82)	
			G3 = 500-999	Trend *p *= 0.014	
			G4 = 1000-1999		
			G5 = ≥2000		
				HR (95% CI) by EE	
				Adjusted for age and sex	
				• G1 = 1.00 (referent)	
				• G2 = 0.64 (0.41-1.01)	
				• G3 = 0.55 (0.35-0.85)	
				• G4 = 0.30 (0.17-0.53)	
				• G5 = 0.24 (0.12-0.48)	
				Trend *p *<0.001	
				Multivariate adjusted	
				• G1 = 1.00 (referent)	
				• G2 = 0.80 (0.49-1.30)	
				• G3 = 0.74 (0.46-1.17)	
				• G4 = 0.50 (0.27-0.90)	
				• G5 = 0.43 (0.21-0.87)	
				Trend *p *= 0.043	

Laukkanen et al 2001 [194]	To examine the relationship between maximal oxygen uptake and overall mortality.	• n = 1,294	Baseline and 10.7 year follow-up	• 124 deaths	PF has a strong, graded, inverse association with overall mortality.
		• Sex: Men		Adjusted RR (95% CI) by quartile	
Finland		• Age: 42.0-61.3 yr (mean 52.1)			
		• Characteristics: Men free from CVD, COPD, and cancer at baseline	PF assessment: Exercise tolerance test, 4 groups by maximal oxygen uptake (ml/kg/min)		
Prospective cohort				Maximal oxygen uptake	
				• G1 = 1.00 (referent)	
				• G2 = 1.47 (0.71-3.01)	
D & B score = 14				• G3 = 2.79 (1.44-5.39)	
			G1 = >37.1	• G4 = 3.85 (2.02-7.32)	
			G2 = 32.3-37.1	Linear trend *p *= <0.001	
			G3 = 27.6-32.2		
			G4 = <27.6	Test duration	
				• G1 = 1.00 (referent)	
			Test duration (min)	• G2 = 2.22 (1.08-4.55)	
			G1 = >11.2	• G3 = 2.23 (1.11-4.49)	
			G2 = 9.6-11.2	• G4 = 3.94 (2.01-7.74)	
			G3 = 8.2-9.5	Linear trend *p*<0.001	
			G4 = <8.2		

Lee and Paffenbarger 2000 [195]	To compare various levels of PA with mortality.	• n = 13,485	Baseline and 15 year follow-up	• 2,539 deaths	The study provides some support for recommendations that emphasize MPA. A benefit of VPA is also evident.
		• Sex: Men			
		• Age: Mean 57.5 yr		RR (95% CI)	
		• Characteristics: Men who matriculated as undergraduates in 1916-1950	PA assessment:	• G1 = 1.00 (referent)	
USA			Questionnaires for LTPA index (including walking, stair climbing, sports and recreational activity),	• G2 = 0.80 (0.72-0.88)	
				• G3 = 0.74 (0.65-0.83)	
Prospective cohort				• G4 = 0.80 (0.69-0.93)	
		• The Harvard Alumni Health Study		• G5 = 0.73 (0.64-0.84)	
				Trend *p *= <0.001	
D & B score = 12			5 groups (kJ/wk)		
			G1 = <4200		
			G2 = 4200-8399		
			G3 = 8400-12599		
			G4 = 12600-16799		
			G5 = ≥ 16800		

Lee et al 1995 [196]	To examine the independent association of vigorous and non-vigorous PA with longevity.	• n = 17,321	Follow-up 22-26 years	• 3,728 deaths	There is a graded inverse relationship between PA and mortality. Vigorous, but not non-vigorous activities are associated with longevity.
		• Sex: Men			
		• Age: Mean 46 yr	PA assessment: Questionnaires for EE (kJ/wk), quintiles	RR (95% CI) by EE (kJ/wk)	
USA		• Characteristics: Harvard University alumni, without self-reported physician diagnosed cardiovascular disease, cancer or chronic obstructive pulmonary disease		Q1= 1.00 (referent)	
				• Q2 = 0.94 (0.86--1.04)	
Prospective cohort			Q1 = ≤ 630	• Q3 = 0.95 (0.86--1.05)	
			Q2 = 630-1680	• Q4 = 0.91 (0.83 - 1.01)	
			Q3 = 1680-3150	• Q5 = 0.91 (0.82-1.00)	
D & B score = 12			Q4 = 3150-6300		
			Q5 = >6300	RR (95% CI) by EE (Vigorous activity, kJ/wk)	
				• Q1 = 1.00 (referent)	
		• The Harvard Alumni Health Study		• Q2 = 0.88 (0.82-0.96)	
				• Q3 = 0.92 (0.82-1.02)	
				• Q4 = 0.87 (0.77-0.99)	
				• Q5 = 0.87 (0.78-0.97)	

Lee et al 2004 [197]	To investigate the effect of various PA patterns on all-cause mortality.	• n = 8,421	Baseline 1988 and follow-up 1993	• 1,234 deaths	The results suggest that regular PA generating 1000 kcal/wk or more should be recommended for lowering mortality rates. Among those with no major risk factors, even 1-2 episodes per week generating 1000 kcal or more can postpone mortality.
		• Sex: Men			
		• Age: Mean 66 yr		Age adjusted RR (95% CI) by PA pattern	
USA		• Characteristics: Participants free of major chronic disease	PA assessment: Questionnaire for PA (kcal/wk), 4 groups		
				• G1 = 1.00 (referent)	
Prospective cohort				• G2 = 0.75 (0.63-0.90)	
			G1 = <500	• G3 = 0.82 (0.63-1.07)	
		• The Harvard Alumni Health Study	(Sedentary)	• G4 = 0.61 (0.53-0.69)	
D & B score = 11			G2 = 500-999		
			(Insufficiently active)	Multivariate adjusted	
			G3 = ≥ 1000		
			(Weekend warrior)	• G1 = 1.00 (referent)	
			G4 = Regularly active	• G2 = 0.75 (0.62-0.91)	
				• G3 = 0.85 (0.65-1.11)	
				• G4 = 0.64 (0.55-0.73)	

Leitzmann et al 2007 [198]	To examine PA guidelines in relation to mortality.	• n = 252,925 (142,828 male; 110,097 women)	Baseline and 6 month follow-up	• 7,900 deaths	Following PA guidelines is associated with lower risk of death. Mortality benefit may also be achieved by engaging in less than recommended activity levels.
USA		• Sex: Men and women	PA assessment: Questionnaire for MPA and VPA, 5 groups each MPA (h/wk)	Multivariate adjusted RR (95% CI) according to activity	
		• Age: 50-71 yr		MPA	
Prospective cohort		• Characteristics: Participants free of CVD, cancer or emphysema		• G1 = 1.00 (referent)	
		• The National Institute of Health-American Association of Retired Persons		• G2 = 0.85 (0.79-0.93)	
				• G3 = 0.79 (0.74-0.85)	
D & B score = 13			G1 = sedentary	• G4 = 0.76 (0.71-0.82)	
			G2 = <1	• G5 = 0.68 (0.63-0.74)	
			G3 = 1-3	Trend *p *= <0.001	
			G4 = 4-7	VPA	
			G5 = >7		
			VPA (frequency/wk)	• G1 = 1.00 (referent)	
			G1 = inactive	• G2 = 0.77(0.71-0.83)	
			G2 = <1	• G3 = 0.77 (0.72-0.82)	
			G3 = 1-2	• G4 = 0.68 (0.63-0.73)	
			G4 = 3-4	• G5 = 0.71 (0.66-0.77)	
			G5 = ≥ 5	Trend *p *= <0.001	
			Cox proportional HR		

Leon et al 1997 [199]	To examine the long-term association of LTPA and risk of death from coronary heart disease and all-causes.	• n = 12,138	16 year follow-up	• 1,904 deaths	The data suggest that a relatively small amount of daily moderate intensity LTPA can reduce premature mortality in middle-aged and older men at high risk for CHD.
		• Sex: Men			
		• Age: 35-57 yr	PA assessment: Minnesota LTPA questionnaire, categorized by frequency/month and average duration, deciles (min/d)	Multivariate adjusted RR (95% CI) by deciles of LTPA	
USA		• Characteristics: Men who at entry to the study were free of clinical evidence of CHD or other serious medical problems but were at the upper 10%-15% of a CHD probability score distribution derived from the FHS data			
Prospective cohort				• D1 = 1.00 (referent)	
				• D2-4 = 0.85 (0.73-0.99)	
				• D5-7 = 0.87 (0.75-1.02)	
D & B score = 12				• D8-10 = 0.83 (0.71-0.97)	
			D1 = 4.9		
			D2-4 = 22.7		
			D5-7 = 53.9		
			D8-10 = 140.4		
		• Multiple Risk Factor Intervention Trial	Cox proportional HR		

Lissner et al 1996 [200]	To examine the relationship of OPA and LTPA on all-cause mortality in women.	• n = 1,405	Baseline and 20 year follow-up	• 277 deaths	Decreases in PA as well as low initial levels are strong risk factors for mortality.
		• Sex: Women			
		• Age: 38-60 yr		RR (95% CI) by LTPA	
Sweden		• Characteristics: Free from major disease at baseline	PA assessment: Questionnaire for OPA and LTPA, 3 groups		
				20 year follow-up	
Prospective cohort				LTPA during age 20-38 years	
		• The Gothenburg Prospective Study of Women		• Low = 1.00 (referent)	
			G1 = Low	• Med = 0.66 (0.34-1.26)	
D & B score = 10			G2 = Medium	• High = 0.46 (0.21-1.01)	
			G3 = High		
				LTPA during age 39-60 years	
			Proportional hazard regression	• Low = 1.00 (referent)	
				• Med = 0.56 (0.35-0.90)	
				• High = 0.44 (0.22-0.91)	
				LTPA during the past 12 months	
				• Low = 1.00 (referent)	
				• Med = 0.56 (0.39-0.82)	
				• High = 0.45 (0.24-0.86)	
				20 year follow-up	
				OPA during age 20-38 years	
				• Low = 1.00 (referent)	
				• Med = 0.59 (0.18-1.87)	
				• High = 0.50 (0.16-1.58)	
				OPA during age 39-60 years	
				• Low = 1.00 (referent)	
				• Med = 0.66 (0.21-2.08)	
				• High = 0.47 (0.14-1.52)	
				OPA during the past 12 months	
				• Low = 1.00 (referent)	
				• Med = 0.28 (0.17-0.46)	
				• High = 0.24 (0.14-0.43)	

Manini et al 2006 [201]	To determine whether energy expenditure is associated with all-cause mortality in older adults.	• n = 302 (150 men; 152 women)	Mean follow-up of 6.15 years	• 55 deaths	Free-living activity EE was strongly associated with lower risk of mortality.
		• Sex: Men and women		HR (95% CI) by tertiles of PA EE	
USA		• Age: 70-82 yr	PA assessment: Questionnaire, divided into tertiles of PA EE (kcal/d)		
Prospective cohort		• Characteristics: High-functioning community dwelling elders		Adjusted for age, sex, race and study site	
			T1 = <521	• T1 = 1.00 (referent)	
D & B score = 13			T2 = 521-770	• T2 = 0.63 (0.29-1.18)	
			T3 = >770	• T3 = 0.37 (0.15-0.76)	
				Trend *p *= 0.009	
				Adjusted for age, sex, race, study site, weight, height, percent body fat and sleep duration	
				• T1 = 1.00 (referent)	
				• T2 = 0.57 (0.30-1.09)	
				• T3 = 0.31 (0.14-0.69)	
				Trend *p *= 0.004	
				Adjusted for age, sex, race, study site, self rated health, education, smoking, CVD, lung disease, diabetes, hip or knee osteoarthritis, osteoporosis, cancer and depression	
				• T1 = 1.00 (referent)	
				• T2 = 0.65 (0.33-1.28)	
				• T3 = 0.33 (0.15-0.74)	
				Trend *p *= 0.007	

Matthews et al 2007 [202]	To determine the effects of exercise and non-exercise PA on mortality.	• n = 67,143	Baseline and an average of 5.7 year follow-up	• 1,091 deaths	Overall PA levels are an important determinant of longevity.
		• Sex: Women			
		• Age: 40-70 yr		RR (95% CI)	
China		• Characteristics: Women without heart disease, stroke or cancer			
			PA assessment: Interview to report (MET h/d), 4 groups Overall activity	Multivariate adjustment	
Prospective cohort				Overall activity (MET hr/d)	
				• G1 = 1.00 (referent)	
		• The Shanghai Women's Health Study		• G2 = 0.81 (0.69-0.96)	
D & B score = 12			G1 = ≤ 9.9	• G3 = 0.67 (0.57-0.80)	
			G2 = 10.0-13.6	• G4 = 0.61 (0.51-0.73)	
			G3 = 13.7-18.0	Trend *p *= 0.000	
			G4 = ≥ 18.1		
				Adult exercise (MET hr/d)	
			Adult exercise	• G1 = 1.00 (referent)	
			G1 = 0	• G2 = 0.84 (0.74-0.96)	
			G2 = 0.1-3.4	• G3 = 0.77 (0.59-0.99)	
			G3 = 3.5-7.0	• G4 = 0.64 (0.36-1.14)	
			G4 = ≥ 7.1	Trend *p *= 0.008	
			Cox proportional hazard models		

Menotti and Seccareccia 1985 [203]	To investigate the relationship between OPA and all-cause mortality.	• n = 99,029	Baseline and 5 year follow-up	• 2,661 deaths	The results suggest that PA may play a role in the prediction of fatal events.
		• Sex: Men			
		• Age: 40-59 yr			
		• Characteristics: Men employed on the Italian railway system	PA assessment: Questionnaire Men at risk classified by 3 levels of PA and 3 levels of job responsibility, combined to create 8 groups of PA-job responsibility	Age adjusted death rates per 1000 over 5 years classified by PA only	
Italy				• Sedentary = 26.20	
Prospective cohort				• Moderate = 27.05	
				• Heavy = 27.35	
D & B score = 12				Age adjusted death rates per 1,000 over 5 years classified by PA and job responsibility	
			G1 = sedentary -- low	• G1 = 30.00	
			G2 = sedentary -- med	• G2 = 25.20	
			G3 = sedentary -- high	• G3 = 25.80	
			G4 = moderate -- low	• G4 = 26.30	
			G5 = moderate -- med	• G5 = 28.50	
			G6 = moderate -- high	• G6 = 25.80	
			G7 = heavy -- low	• G7 = 26.90	
			G8 = heavy -- med	• G8 = 30.80	

Mensink et al 1996 [204]	To compare various indices for PA and their association with cardiovascular risk factors as well as total and CVD mortality.	• n = 15,436 (7,689 men; 7797 women)	5-8 year follow-up	Incidence of all-cause mortality and PA	An inverse relation of PA and total mortality.
Germany		• Sex: Men and women	PA assessment: Questionnaire Total activity, 3 groups	Adjusted RR (95% CI)	
		• Age: 25-69 yr			
Prospective cohort		• Characteristics: Participants from communities in Western Germany		Total activity, men	
			G1 = Low	• G1 = 1.00 (referent)	
			G2 = Moderate	• G2 = 0.56 (0.30-1.04)	
D & B score = 12			G3 = High	• G3 = 0.78 (0.42-1.44)	
				Total activity, women	
			LTPA, 3 groups	• G1 = 1.00 (referent)	
			G1 = Low	• G2 = 1.24 (0.60-2.58)	
			G2 = Moderate	• G3 = 1.29 (0.58-2.85)	
			G3 = High		
			Conditioning activity, 3 groups	LTPA, men	
				• G1 = 1.00 (referent)	
			G1 = No activity	• G2 = 0.61 (0.35-1.05)	
			G2 = Moderate	• G3 = 0.79 (0.48-1.31)	
			G3 = High	LTPA, women	
				• G1 = 1.00 (referent)	
			Sports activity, 4 groups	• G2 = 0.94 (0.51-1.75)	
				• G3 = 0.81 (0.44-1.49)	
			G1 = no sports		
			G2 = <1 hour	Conditioning activity, men	
			G3 = 1-2 hours	• G1 = 1.00 (referent)	
			G4 = >2 hours	• G2 = 0.76 (0.44-1.34)	
				• G3 = 0.67 (0.36-1.25)	
				Conditioning activity, women	
				• G1 = 1.00 (referent)	
				• G2 = 0.38 (0.13-1.06)	
				• G3 = 0.80 (0.42-1.54)	
				Sports Activity, men	
				• G1 = 1.00 (referent)	
				• G2 = 0.49 (0.26-0.95)	
				• G3 = 0.57 (0.30-1.09)	
				• G4 = 0.36 (0.16-0.79)	
				Sports activity, women	
				• G1 = 1.00 (referent)	
				• G2 = 0.38 (0.12-1.23)	
				• G3 = 0.52 (0.23-1.17)	
				• G4 = 0.28 (0.07-1.17)	

Morgan and Clarke 1997 [205]	To assess the value of broadly based customary PA scores in predicting 10-year mortality in elderly people.	• n = 1,042 (407 men; 635 women)	10 year follow-up	Incidence of all-cause mortality and PA	A wide range of customary or habitual PA, can provide indices showing both cross sectional and predictive validity for 10 year mortality.
		• Sex: Men and women	PA assessment: Questionnaire for PA, 3 groups		
UK		• Age: ≥65 yr		HR (95% CI)	
		• Characteristics: British elders		Men	
Prospective cohort			G1 = Low	• G1 = 1.59 (1.12-2.25)	
		• Nottingham Longitudinal Study of Activity and Aging	G2 = Intermediate	• G2 = 1.35 (0.96-1.89)	
			G3 = High	• G3 = 1.00 (referent)	
D & B score = 12				Women	
				• G1 = 2.07 (1.53-2.79)	
				• G2 = 1.53 (1.12-2.09)	
				• G3 = 1.00 (referent)	

Myers et al 2002 [206]	To compare PF and PA levels with all-cause mortality.	• n = 6,213	Baseline and mean 6.2 ± 3.7 year follow-up	• 1,256 deaths	Exercise capacity is a more powerful predictor of mortality among men than other established risk factors for CVD.
		• Sex: Men			
		• Age: Mean 59 ± 11 yr		Age adjusted RR (95% CI) by quintile	
USA		• Characteristics: Participants with a normal exercise test result (n = 2,534) and participants with an abnormal exercise test or CVD or both (n = 3,679)			
			PF assessment: Treadmill test for VO_2 _peak, divided into quintiles (METs)	• Q1 = 4.5 (3.0-6.8)	
Prospective cohort				• Q2 = 2.4 (1.5-3.8)	
				• Q3 = 1.7 (1.1-2.8)	
				• Q4 = 1.3 (0.7-2.2)	
D & B score = 12			Q1 = Lowest level	• Q5 = 1.00 (referent)	
			1.0-5.9		
			Q2		
			Q3		
			Q4		
			Q5 = Highest level		
			≥13.0		

Ostbye et al 2002 [207]	To analyze the effect of smoking and other modifiable risk factors on ill health, defined in a multidimensional fashion.	• n = 12,956	6 year follow-up	• 782 deaths	Quitting smoking and increasing exercise levels are the lifestyle interventions most likely to improve overall health.
		• Sex: Men and women			
		• Age: 50-60 yr	PA assessment: Questionnaire for PA, 4 groups	Incidence of all-cause mortality and PA	
USA		• Characteristics: Participants from the Health and Retirement Study (HRS) only			
Prospective cohort			G1 = Sedentary	Death rates (95% CI) per 1000 population/yr	
			G2 = Light		
			G3 = Moderate	• G1 = 20.6 (17.8-24.0)	
D & B score = 13			G4 = Heavy	• G2 = 9.1 (8.1-9.5)	
				• G3 = 8.3 (7.5-9.2)	
				• G4 = 4.4 (3.5-5.6)	

Paffenbarger et al 1994 [208]	To study the adoption or maintenance of PA and other optional lifestyle patterns for their influence on mortality rates of Harvard College alumni.	• n = 14,786	Follow-up between	• 2,343 deaths	Adopting a physically active lifeway delays mortality and extends longevity.
		• Sex: Men	1977 and 1988		
		• Age: 45-84 yr (in 1977)		RR (95% CI) of mortality according to PA	
USA			PA assessment: Questionnaire for blocks walked daily, stairs climbed daily and type, frequency and duration of weekly sports and recreational activities		
		Characteristics: Harvard College alumni			
Prospective cohort				Physical activity index (kcal/wk)	
				• G1 = 1.00 (referent)	
D & B score = 14				• G2 = 1.13 (1.01-1.26)	
				• G3 = 0.72 (0.64-0.82)	
				• G4 = 0.77 (0.69-0.85)	
			Physical activity index (kcal/wk) Sports and recreational activities were scored according to intensity and duration	Walking (km/wk)	
				• G1 = 1.00 (referent)	
				• G2 = 1.21 (1.08-1.35)	
				• G3 = 0.94 (0.83-1.07)	
				• G4 = 0.89 (0.78-1.01)	
				Moderately vigorous sports play (METs)	
			Light < 4.5 METs		
			Moderate ≥ 4.5 METs		
				• G1 = 1.00 (referent)	
				• G2 = 1.11 (0.93-1.33)	
				• G3 = 0.73 (0.65-0.81)	
				• G4 = 0.72 (0.64-0.80)	
				Adjusted for potential confounding influences	

Richardson et al 2004 [209]	To investigate the impact of a sedentary lifestyle on all-cause mortality.	• n = 9,611 (4,642 men; 4,969 women)	Baseline (1992) and 8 year follow-up	• 810 deaths	A sedentary lifestyle is associated with a higher risk of death in pre- retirement aged adults.
		• Sex: Men and women		OR (95% CI)	
USA		• Age: 51-61 yr	PA assessment: Questionnaire for PA, 3 groups:	• G1 = 1.00 (referent)	
Prospective cohort		• Characteristics: Participants born between 1931-1941 and who not institutionalized in 1992		• G2 = 0.64 (0.52-0.81)	
			G1 = Sedentary	• G3 = 0.62 (0.44-0.85)	
			G2 = occasional or light	*p *= 0.01	
D & B score = 13			G3 = Regular MVPA		
		• Health and Retirement Study			

Rockhill et al 2001 [210]	To determine the association between recreational PA and mortality in women.	• n = 80,348	Baseline (1980) and follow-up between 1982-1996	• 4,871 deaths	People who are more physically active are at reduced mortality risk relative to those who are less active.
		• Sex: Women			
		• Age: 30-55 yr		Multivariate adjusted RR (95% CI) by (hr/wk)	
USA		• Characteristics: Free from CVD or cancer at baseline			
		• Nurses Health Study	PA assessment: Questionnaire in 1980 and up-dated every 2- 4 years, 5 groups of PA (hr/wk)	• G1 = 1.00 (referent)	
Prospective cohort				• G2 = 0.82 (0.76-0.89)	
				• G3 = 0.75 (0.69-0.81)	
				• G4 = 0.74 (0.68-0.81)	
D & B score = 11				• G5 = 0.71 (0.61-0.82)	
				*p*<0.001	
			G1 = <1		
			G2 = 1-1.9		
			G3 = 2-3.9		
			G4 = 4-6.9		
			G5 = ≥7		

Rosengren and Wilhelmsen 1997 [211]	To investigate the effect of OPA and LTPA on risk of death.	• n = 7,142	Baseline (1970-1973) and 20 year follow-up	• 2,182 deaths	The study demonstrates the protective effect of LTPA on mortality.
		• Sex: Men			
		• Age: 47-55 yr		Unadjusted RR (95% CI)	
		• Characteristics: Without symptomatic CHD	PA assessment: Postal questionnaires, 3 groups:	• G1 = 1.00 (referent)	
Sweden				• G2 = 0.74 (0.68-0.82)	
				• G3 = 0.73 (0.68-0.79)	
Prospective cohort			G1 = Sedentary		
			G2 = Moderately active	Multivariate adjustment	
			G3 = Regular exercise	• G1 = 1.00 (referent)	
D & B score = 13				• G2 = 0.84 (0.77-0.93)	
				• G3 = 0.83 (0.77-0.90)	

Schnohr et al 2003 [212]	To assess the associations of regular LTPA and changes in LTPA with risk of death.	• n = 7,023 (4,471 men; 5,676 women)	18 year follow-up	• 2,725 deaths	Maintaining or adopting a moderate or high degree of PA was associated with lower risk of death.
		• Sex: Men and women	PA assessment: Questionnaire, 9 groups	Incidence of all-cause mortality and PA and changes in PA	
Denmark		• Age: 20-79 yr			
		• Characteristics: Participants from the Copenhagen City Heart Registered Population			
Prospective cohort			G1 = Low--low		
			G2 = Low--moderate	Adjusted RR (95% CI)	
			G3 = Low--high	Men	
D & B score = 12			G4 = Moderate- low	• G1 = 1.00 (referent)	
			G5 = Moderate-Moderate	• G2 = 0.64 (0.49-0.83)	
				• G3 = 0.64 (0.47-0.87)	
			G6 = Moderate-high	• G4 = 0.73 (0.56-0.96)	
			G7 = High-low	• G5 = 0.71 (0.57-0.88)	
			G8 = High-moderate	• G6 = 0.64 (0.51-0.81)	
			G9 = High-high	• G7 = 1.11 (0.76-1.62)	
				• G8 = 0.66 (0.51-0.85)	
				• G9 = 0.61 (0.48-0.76)	
				Women	
				• G1 = 1.00 (referent)	
				• G2 = 0.75 (0.57-0.97)	
				• G3 = 0.72 (0.50-1.05)	
				• G4 = 0.70 (0.54-0.91)	
				• G5 = 0.64 (0.52-0.79)	
				• G6 = 0.58 (0.45-0.73)	
				• G7 = 0.72 (0.48-1.07)	
				• G8 = 0.61 (0.47-0.80)	
				• G9 = 0.66 (0.51-0.85)	

Schnohr et al 2004 [213]	To examine whether the relationship between established risk factors and mortality differs with socioeconomic status as measured by level of education.	• n = 30,635 (16,236 men; 14,399 women)	16 year follow-up	• 10,952 deaths	The study shows the strong predictive effect of PA on mortality is independent of education level.
		• Sex: Men and women	Socioeconomic status assessment: level of education	Incidence of all-cause mortality and PA stratified by years of education	
Denmark		• Age: 20-93 yr			
		• Characteristics: Participants from the Copenhagen City Heart Registered Population			
Prospective cohort			PA assessment: Questionnaire	Deaths <8 years of education	
D & B score = 12				Men	
			4 groups of PA	G1 = 916	
			G1 = none or very little	G2 = 1693	
			G2 = 2-4 h/wk of LPA	G3 = 1012	
			G3 = >4 h/wk of LPA or 2-4 h/wk of high level activity	G4 = 67	
			G4 = Competition level or >4 h/wk of hard level activity	Women	
				• G1 = 872	
				• G2 = 1298	
				• G3 = 346	
				• G4 = 10	
				8-11 years of education	
				Men	
				• G1 = 432	
				• G2 = 1040	
				• G3 = 616	
				• G4 = 33	
				Women	
				• G1 = 363	
				• G2 = 852	
				• G3 = 268	
				• G4 = 10	
				>11 years of education	
				Men	
				• G1 = 104	
				• G2 = 302	
				• G3 = 182	
				• G4 = 11	
				Women	
				• G1 = 48	
				• G2 = 129	
				• G3 = 61	
				• G4 = 3	

Schnohr et al 2006 [214]	To investigate the association between LTPA and mortality.	• n = 4,894 (2,136 men; 2,758 women)	Baseline (1976) and start of follow-up in 1981-1983 (to 2000)	• 1,787 deaths	Long-term moderate or high PA was associated with significantly lower mortality in men and women.
		• Sex: Men and women		RR (95% CI)	
Denmark		• Age: 20-79 yr			
		• Characteristics: Healthy males and women		Unadjusted	
Prospective cohort			PA assessment: Survey for LTPA, 3 groups:	• G1 = 1.00 (referent)	
				• G2 = 0.64 (0.56-0.73)	
		• The Copenhagen City Heart Study		• G3 = 0.56 (0.48-0.65)	
D & B score = 13			G1 = Low	Trend *p *< 0.001	
			G2 = Mod		
			G3 = High	Multivariate adjustment	
				• G1 = 1.00 (referent)	
				• G2 = 0.78 (0.68-0.89)	
				• G3 = 0.75 (0.64-0.87)	
				Trend *p *= 0.001	

Schooling et al 2006 [215]	To examine how a Comprehensive assessment of baseline health status affects the relationship between obesity or PA and mortality.	• n = 54,088 (17,849 men; 36,239 women)	4.1 year follow-up	• 3,819 deaths	PA, which normally has a negative relationship with adiposity, had the largest impact on survival for the health states, with the strongest inverse relationship between BMI and mortality.
		• Sex: Men and women	PA assessment: Interview for PA min/d, 3 groups	Incidence of all-cause mortality and PA	
Hong Kong		• Age: ≥ 65 yr			
Prospective cohort		• Characteristics: Chinese elders	G1 = None	Adjusted HR (95% CI)	
			G2 = ≤ 30 min/d	• G1 = 1.00 (referent)	
			G3 = ≥ 30 min/d	• G2 = 0.83 (0.76-0.91)	
D & B score = 13				• G3 = 0.73 (0.67-0.80)	
				Trend *p*<0.001	

Sundquist et al 2004 [216]	To study the association between varying levels of PA and all-cause mortality in the elderly.	• n = 3,206 (1,414 men; 1,792 women)	Baseline (1988-1989) and follow-up in 2000	• 1,806 deaths	Even occasional PA decreases the risk of mortality among elderly people.
		• Sex: Men and women	PA assessment: Questionnaire for PA, 5 groups	Age-adjusted HR (95% CI)	
Sweden		• Age: ≥65 yr		Men	
		• Characteristics: Non-institutionalized elders		• G1 = 1.00 (referent)	
Prospective cohort				• G2 = 0.74 (0.62-0.87)	
			G1 = none	• G3 = 0.57 (0.44-0.73)	
		The Swedish Annual Level-of-Living Survey (Statistics Sweden)	G2 = occasionally	• G4 = 0.51 (0.41-0.64)	
D & B score = 12			G3 = once per week	• G5 = 0.60 (0.44-0.82)	
			G4 = twice per week	Women	
			G5 = vigorously at least twice per week	• G1 = 1.00 (referent)	
				• G2 = 0.70 (0.59-0.82)	
				• G3 = 0.59 (0.46-0.77)	
			Cox proportional HR	• G4 = 0.47 (0.35-0.62)	
				• G5 = 0.54 (0.31-0.94)	
				Men and women	
				Multivariate adjustment	
				• G1 = 1.00 (referent)	
				• G2 = 0.72 (0.64-0.81)	
				• G3 = 0.60 (0.50-0.71)	
				• G4 = 0.50 (0.42-0.59)	
				• G5 = 0.60 (0.46-0.79)	

Talbot et al 2007 [217]	To investigate how changes in LTPA affect all-cause mortality.	• n = 2,092 (1,316 men; 776 women)	Baseline in 1958 for males and in 1978 for females and an average follow-up of 21.2 ± 9.4 years for men and 10.2 ± 5.6 years for women	• 628 deaths (538 male; 90 female)	Greater declines in total and high-intensity LTPA are independent predictors of all-cause mortality.
		• Sex: Men and women			
USA		• Age: 19-<90 yr		RR (95% CI) for standard deviation of rate of change in LTPA	
Prospective cohort		• Characteristics: Community residents, generally with above average income, high education and with good or excellent self related health		(*If RR is <1 then a SD increase is associated with decrease mortality. If RR is >1, then a SD increase is associated with increase in mortality*)	
D & B score = 13			PA assessment: Questionnaire for LTPA (METs min/24 h), 3 groups		
		The Baltimore Longitudinal Study of Aging	G1 = low		
			G2 = medium	Multivariate adjustment	
			G3 = high	Men <70 years	
			Rate of change (ROC)	• G1 = 0.96 (0.84-1.08)	
				• G2 = 0.91 (0.79-1.04)	
				• G3 = 0.42 (0.33-0.53)	
				• ROC low = 0.90 (0.80-1.01)	
				• ROC med = 1.01 (0.90-1.14)	
				• ROC high = 0.78 (0.65-0.94)	
				Men >70 years	
				• G1 = 0.95 (0.82-1.10)	
				• G2 = 0.89 (0.76-1.05)	
				• G3 = 0.78 (0.62-0.97)	
				• ROC low = 1.07 (0.93-1.24)	
				• ROC med = 1.13 (1.00-1.27)	
				• ROC high = 0.91 (0.75-1.12)	
				Women <70 years	
				• G1 = 0.75 (0.53-1.07)	
				• G2 = 0.61 (0.36-1.03)	
				• G3 = 0.80 (0.50-1.30)	
				• ROC low = 1.02 (0.74-1.40)	
				• ROC med = 1.38 (0.86-2.28)	
				• ROC high = 0.90 (0.63-1.27)	
				Women >70 years	
				• G1 = 0.85 (0.63-1.15)	
				• G2 = 0.78 (0.39-1.59)	
				• G3 = 0.62 (0.32-1.22)	
				• ROC low = 1.10 (0.85-1.42)	
				• ROC med = 0.96 (0.46-2.03)	
				• ROC high = 0.70 (0.40-1.22)	

Trolle-Lagerros et al 2005 [218]	To quantify the effect of PA on overall mortality in younger women and to assess the effect of past versus current activity.	• n = 99,099	11.4 year follow-up	• 1,313 deaths	Current PA substantially reduces mortality among women. The association is observed even with low levels of PA and is accentuated with increased PA.
		• Sex: Women			
		• Age: 30-49 yr	PA assessment: Questionnaire using a 5 point scale, 5 groups	Incidence of all-cause mortality and PA past and current	
Sweden and Norway		• Characteristics: Participants from Norway and one region of Sweden			
Retrospective cohort			G1 = Sedentary	Adjusted HR (95% CI)	
			G2 = Low	PA at enrolment	
			G3 = Moderate	• G1 = 1.00 (referent)	
D & B score = 13			G4 = High	• G2 = 0.78 (0.61-1.00)	
			G5 = Vigorous	• G3 = 0.62 (0.49-0.78)	
				• G4 = 0.58 (0.44-0.75)	
				• G5 = 0.46 (0.33-0.65)	
				Trend *p*<0.0001	
				PA at age 30 yr	
				• G1 = 1.00 (referent)	
				• G2 = 0.79 (0.55-1.15)	
				• G3 = 0.90 (0.64-1.28)	
				• G4 = 0.98 (0.68-1.42)	
				• G5 = 0.96 (0.65-1.44)	
				Trend *p *= 0.22	
				PA at age 14 yr	
				• G1 = 1.00 (referent)	
				• G2 = 0.95 (0.66-1.38)	
				• G3 = 0.96 (0.69-1.34)	
				• G4 = 0.88 (0.62-1.25)	
				• G5 = 1.06 (0.75-1.51)	
				Trend *p *= 0.62	

Villeneuve et al 1998 [219]	To examine the relationship between PF, PA and all-cause mortality.	• n = 14,442 (6,246 men; 8,196 women)	Baseline (1981) and 7 year follow-up	RR (95% CI) by EE, multivariate adjustment	There was a reduction in mortality risk associated with even modest participation in activities of low intensity.
		• Sex: Men and women			
Canada		• Age: 20-69 yr	PA assessment: Questionnaire for EE (kcal/kg/day), 5 groups	LTPA, men	
		• Characteristics: Asymptomatic for CVD		• G1 = 1.00 (referent)	
Prospective cohort				• G2 = 0.81 (0.59-1.11)	
				• G3 = 0.79 (0.54-1.13)	
		Canadian Fitness Survey	G1 = 0-<0.5	• G4 = 0.86 (0.61-1.22)	
D & B score = 11			G2 = 0.5-<1.5	• G5 = 0.82 (0.65-1.04)*	
			G3 = 1.5-<3.0		
			G4 = ≥ 3.0	Non vigorous LTPA, men	
			G5 = ≥ 0.5	• G1 = 1.00 (referent)	
			PF levels:	• G2 = 0.81 (0.56-1.17)	
			Recommended	• G3 = 0.70 (0.44-1.13)	
			Minimum	• G4 = 0.82 (0.53-1.27)	
				• G5 = 0.78 (0.59-1.04)*	
			Undesirable Refusal		
				LTPA, women	
			Multivariate Poisson regression analysis	• G1 = 1.00 (referent)	
				• G2 = 0.94 (0.69-1.30)	
				• G3 = 0.92 (0.64-1.34)	
				• G4 = 0.71 (0.45-1.11)	
				• G5 = 0.88 (0.68-1.04)*	
				Non vigorous LTPA, women	
				• G1 = 1.00 (referent)	
				• G2 = 0.97 (0.69-1.36)	
				• G3 = 0.87 (0.57-1.33)	
				• G4 = 0.72 (0.43-1.21)	
				• G5 = 0.89 (0.67-1.17)*	
				RR (95% CI) by fitness levels, adjusted for age, sex and smoking Recommended = 1.00 (referent)	
				• Minimum = 1.02 (0.69-1.51)	
				• Undesirable = 1.52 (0.72-3.18)	
				• Refusal = 1.04 (0.45-2.39)	

Weller and Corey 1998 [220]	To study the relationship between PA and mortality in women.	• n = 6,620	Baseline and 7 year follow-up	• 449 deaths	PA is inversely associated with risk of death in women.
		• Sex: Women			
		• Age: ≥;30 yr		OR (95% CI)	
Canada		• Characteristics: Without known heart disease	PA assessment: Questionnaires for: EE (kcal/kg/d), quartiles		
		• Canadian Fitness Survey		EE (kcal/kg/d)	
Prospective cohort				• Q1 = 1.00 (referent)	
				• Q2 = 0.91 (0.66-1.25)	
			Q1 = lowest	• Q3 = 0.94 (0.72-1.23)	
D & B score = 11			Q2 =	• Q4 = 0.89 (0.67-1.17)	
			Q3 =		
			Q4 = highest	LTPA levels	
			LTPA, 3 groups	• G1 = 1.00 (referent)	
			G1 = Sedentary	• G2 = 0.63 (0.46-0.86)	
			G2 = Mod	• G3 = 0.76 (0.59-0.98)	
			G3 = High		
				Walking	
			Walking, 3 groups	• G1 = 1.00 (referent)	
			G1 = < half the time	• G2 = 0.64 (0.49-0.82)	
			G2 = half the time	• G3 = 0.64 (0.47-0.86)	
			G3 = > half the time		

Yu et al 2003 [221]	To examine the relationship between LTPA and all-cause mortality.	• n = 1,975	Baseline and 10 year follow-up	• 252 deaths	The study found a strong inverse association between heavy LTPA and all-cause mortality.
UK		• Sex: Men			
		• Age: 49-64 yr		Age adjusted HR (95% CI)	
		• Characteristics: Without a history of CHD at baseline	PA assessment: Questionnaire (Minnesota LTPA index, kcal/d), 3 group	• G1 = 1.00 (referent)	
				• G2 = 0.73 (0.54-0.99)	
Prospective cohort				• G3 = 0.74 (0.55-1.04)	
				Trend *p *= 0.046	
D & B score = 11			G1 = Light to no activity	Multivariate adjusted	
			G2 = Moderate activity	• G1 = 1.00 (referent)	
			G3 = Heavy activity	• G2 = 0.79 (0.58-1.08)	
				• G3 = 0.76 (0.56-1.04)	
				Trend *p *= 0.083	

D & B score, Downs and Black quality score; PF, physical fitness; YR, years; RR, risk ratio; 95% CI, 95% confidence interval; PA, physical activity; VO2 peak, peak oxygen consumption; HR, hazard ratio; min/d, minutes per day; kcal/wk, kilocalories per week; LTPA, leisure-time physical activity; MET, metabolic equivalent; VO2 max, maximal oxygen consumption; OPA, occupational physical activity; CVD, cardiovascular disease; hr/wk, hours per week; MPA, moderate physical activity; kcal/kg/wk, kilocalories per kilogram per week; kJ/wk, kilojoules per week; EE, energy expenditure; G, groups; EE, energy expenditure; BMI, body mass index; C, class; kg/m^2^, kilogram by meters squared; HR, heart rate; BPM, beats per minute; MVPA, moderate to vigorous physical activity; OR, odds ratio; Q, quartile or quintile; RCT, randomized clinical trial; T, tertiles; TPA, total physical activity; VPA, vigorous physical activity; mL/kg/min, milliliters per kilogram per minute.

We observed a mean 31% lower risk for all-cause mortality in the most active individuals. The median risk reduction was 32%. It is important to highlight that many of these studies included women, with sub-analyses that revealed similar risk reductions between sexes. Our findings are consistent with previous reports [15,16,29-31]. The majority (90%) of the studies supported the health benefits of physical activity demonstrating a significant risk reduction in physically active individuals. *The level of evidence would be considered to be a Level 2A based on the presence of overwhelming evidence from observational trials*. The studies examined were generally of a good quality with a mean (and median) score of 12 out of 15 (range 10-14).

**Figure 3:**
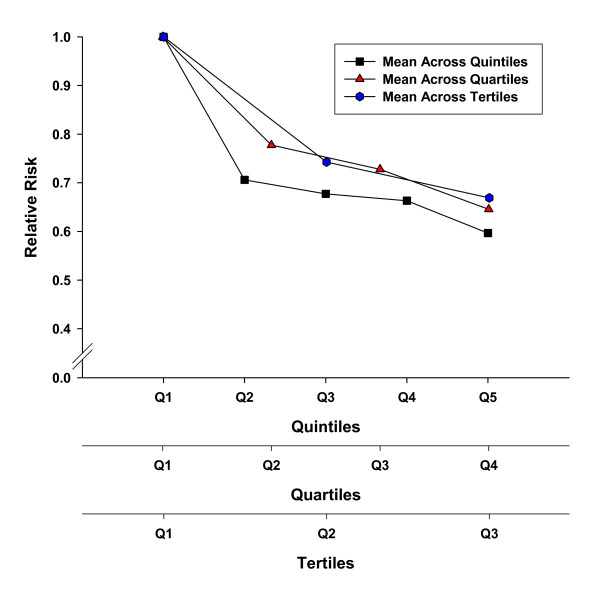
**Mean relative risk reduction in all-cause mortality across physical activity/fitness categories**.

A clear dose-response relationship was also observed with marked reductions in the risk for all-cause mortality occurring with relatively small increments in physical activity (Figure 3). To examine more closely the temporal relationship between physical activity and all-cause mortality we calculated the (unadjusted) relative risks associated with incremental levels of physical activity/fitness using the reported cases of all-cause mortality and the number of participants (per group) in each investigation. In some instances, we were required to calculate the number of participants based on the reported incidence rates and person years, or based on data obtained directly from the authors (2 investigations). We were not able to obtain this information in 18 investigations, and as such this analysis was restricted to the remaining 52 investigations. There was considerable variability in the methods of classifying the physical activity/fitness levels of the participants. Accordingly, Figure 3 illustrates the mean relative risk reduction according to three separate study types including those that subdivided participants into tertiles, quartiles and quintiles, respectively. This figure demonstrates clearly the dose-response relationship between physical activity and all-cause mortality. Collectively, the literature is consistent indicating that the current Canadian guidelines (approximately 4.2 MJ/wk, 1000 kcal/wk) are associated with a 20-30% lower risk for premature all-cause mortality, with greater health benefits with high volumes and/or intensities of activity. In our analyses it was apparent that the greatest differences in risk occurred between the lowest adjacent activity/fitness categories, suggesting that sedentary individuals can markedly reduce their risk for all-cause mortality with relatively minor increments in physical activity. This is consistent with the current messaging of Canada's physical activity guidelines.

The strength of the relationship between physical fitness and premature mortality has been well-established [6,32,33]. In our analyses there were greater risk reductions in studies that took objective measures of physical fitness. We observed an average risk reduction of approximately 45%, which was consistent between men and women. A risk reduction of greater than 50% was not uncommon in these studies. For instance, Myers et al. (2004) reported that being fit or physically active was associated with greater than 50% lower mortality risk in men. They also noted that a 4.2 MJ/wk (1000 kcal/wk) increase in physical activity, or a 1 metabolic equivalent (MET) higher physical fitness level was associated with a mortality benefit of around 20%. It is also important to highlight that longitudinal studies evaluating changes in physical activity or fitness have revealed a lower premature mortality risk [16,34-41]. As we previously reported, routine physical activity or elevated physical fitness also appears to reduce the risk for premature mortality in individuals with risk factors for chronic disease [42,43].

#### Implications

Since the seminal work of Morris and colleagues (in the 1950s [44,45]) and the early work of Paffenbarger (in the 1970s [46,47]) there has been considerable research (especially epidemiological evidence) documenting the health benefits of engaging in routine physical activity and/or being physically fit [17,48]. Both physical activity (a behaviour) and physical fitness (an attained state) appear to be related to health status in a dose-dependent fashion, with physical fitness demonstrating the strongest relationship [18,19]. Numerous reports indicate that physical inactivity and/or low physical fitness are associated with an increased risk for chronic disease and premature all-cause and disease-specific mortality [2,43,49-51]. Some of the most compelling research includes the relationship between physical activity/fitness and all-cause mortality. As demonstrated below and in Table 11 and Figure 1, this literature is extensive.

The assessment of the relationship between all-cause mortality is complicated by the inclusion of deaths related to suicides, homicide, and accidents [18,19,52]. Nonetheless, the available evidence is incontrovertible; individuals who are habitually physically active and/or physically fit are at a markedly reduced risk for premature all-cause mortality [15,16,18,19]. In Canada, physical inactivity is a major cause of premature mortality from diseases of the cardiovascular system (33.3%), cancers (29.1%), and type 2 diabetes (3.5%) [53]. Globally, physical inactivity has been linked with 2 million premature deaths per year, including 22% of cases of coronary heart disease, and 10-16% of cases of breast cancer, colon cancer, rectal cancer and type 2 diabetes [54]. As such, the promotion of the health benefits of physical activity is of paramount importance for the effective prevention of chronic disease and premature mortality on a national and international scale.

In summary, there is a clear dose-response relationship between physical activity and premature all-cause mortality. Physically active individuals have an approximate risk reduction of 31% in comparison to physically inactive individuals. When objective measures of aerobic fitness are taken the risk reductions are even greater approximating 45%.

*Recommendation #1*

*For a reduced risk for premature mortality, it is recommended that individuals should participate in 30 min or more of moderate to vigorous exercise on most days of the week. Greater health benefits appear to occur with higher volumes and/or intensities of activity. [Level 2, Grade A]*

### Primary Prevention of Cardiovascular Disease

**Figure 4:**
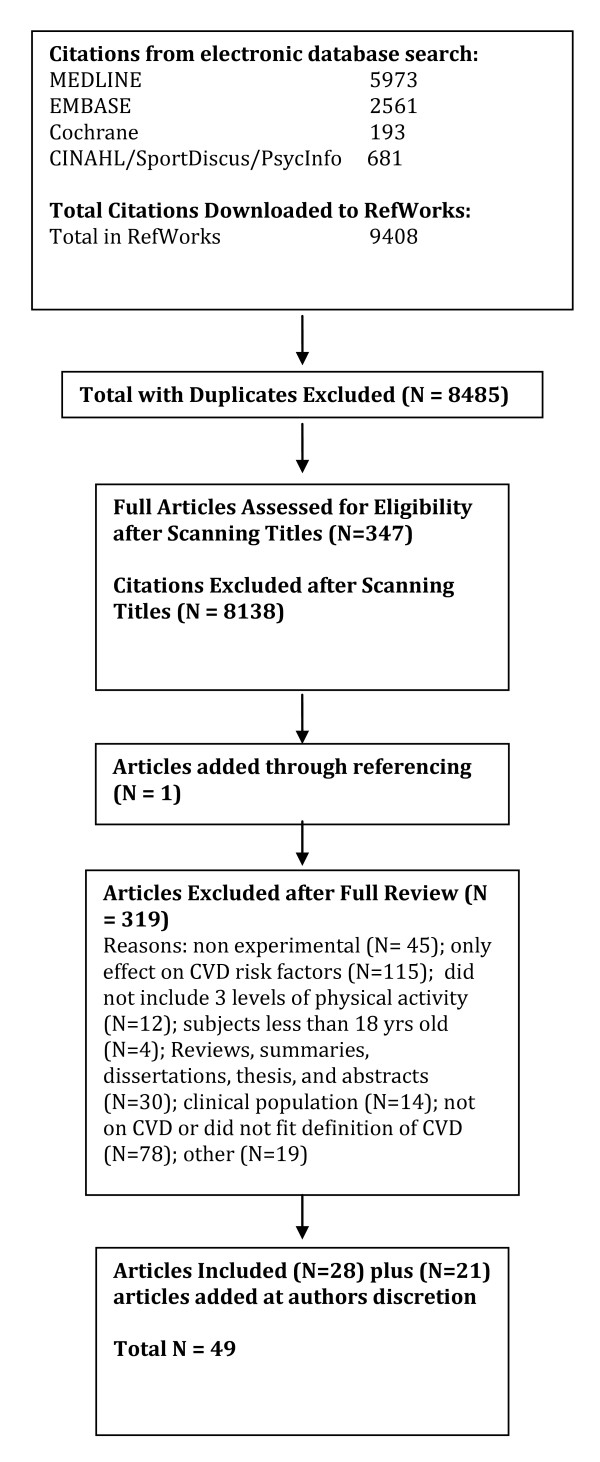
**Results of the Literature Search for Cardiovascular Disease**.

In our systematic search of the literature, a total of 9408 citations were identified during the electronic database search (Figure 4). Of these citations, 5973 were identified in MEDLINE, 2561 in EMBASE, 193 in Cochrane, and 681 in the CINAHL/SportDiscus/PsychInfo search. A total of 923 duplicates were found, leaving a total of 8485 unique citations. A total of 8138 articles were excluded after scanning, leaving a total of 347 articles for full review. An additional 20 articles were added through cross-referencing. From these articles 319 were excluded after full review leaving 33 articles for inclusion in the systematic review. The reasons for exclusion included non-experimental studies (n = 45), only effect on cardiovascular disease risk factors (n = 115), did not report 3 levels of physical activity (n = 12), subjects less than 18 yr of age (n = 4), reviews, summaries, dissertations, thesis, and abstracts (n = 30), clinical population (n = 14), not on cardiovascular disease or did not fit definition of cardiovascular disease (n = 78), and other (n = 19). Therefore, a total of 49 articles were included in the systematic review of the literature regarding the relationship between physical activity and the incidence of cardiovascular disease.

The majority of the studies included in our systematic review were prospective cohort investigations (Table 12). These studies involved a total of 726,474 participants; averaging 12,313 participants per study (range 680-88,393). There were a total of 34,815 reported cases of cardiovascular disease (ranging per study from 42-2,596). The total length of study follow-up for the prospective cohort studies averaged 14.1 yr (ranging from 2-29 yr). The articles were published over a 32 yr period ranging from 1975 to 2007. These studies involved large samples of men and women from regions throughout the world.

**Table 12:** Studies examining the relationship between physical activity and cardiovascular disease.

Publication Country Study Design Quality Score	Objective	Population	Methods	Outcome	Comments and Conclusions
Paffenbarger and Hale 1975 [47]	To evaluate the role of PA in reducing coronary mortality among longshoreman	• n = 6,351	22 years of follow up, or until reached the age of 75 yr	RR (95% CI) Sudden death	VPA is associated with reduced risk of coronary mortality, particularly sudden cardiac death.
USA		• Sex: Men		• G1 = 1.00 (referent)	
		• Age: 35-74 yr		• G2 = 3.5	
		• Characteristics: Longshoreman	PA assessment: Energy and oxygen cost requirements of longshoring jobs	• G3 = 2.8	
Prospective cohort				Delayed death	
				• G1 = 1.00 (referent)	
D & B score = 12				• G2 = 1.4	
			Activity level	• G3 = 1.5	
			G1 = Heavy (5.2-7.5 kcal/min)	Unspecified death	
			G2 = Moderate (2.4-5.0 kcal/min)	• G1 = 1.00 (referent)	
			G3 = Light (1.5-2.0 kcal/min)	• G2 = 1.1	
				• G3 = 1.6	
			Outcome measure: Death from CHD		

Manson et al 2002 [56]	To compare the roles of walking and vigorous exercise in the prevention of CV events in a large, ethnically diverse cohort of postmenopausal women.	• n = 73,743	Enrolment from 1994-98 Clinic visit for baseline screening,	• Number of New Cases: 345	Both walking and VPA are associated with substantial reductions in the incidence of CHD events.
USA		• Sex: Women		• Total Number of CVD events: 1551	
		• Age: 50-79 yr			
		• Characteristics: Healthy, Post Menopausal		Age adjusted RR (95% CI) Total exercise (MET-hr/wk)	
			PA assessment: Questionnaire for: Total exercise (MET- hr/wk)		
Prospective cohort			G1 = 0-2.4	• G1 = 1.00 (referent)	
		• Women's Health Initiative Observational Study	G2 = 2.5-7.2	• G2 = 0.73 (0.53-0.99)	
			G3 = 7.3-13.4	• G3 = 0.69 (0.51-0.95)	
D & B score = 12			G4 = 13.5-23.3	• G4 = 0.68 (0.50-0.93)	
			G5 = ≥ 23.4	• G5 = 0.47 (0.33-0.67)	
				*p *= <0.001	
				Walking (MET-hr/wk)	
			Walking (MET-hr/wk)	• G1 = 1.00 (referent)	
			G1 = None	• G2 = 0.71 (0.53-0.96)	
			G2 = 0.1-2.5	• G3 = 0.60 (0.44-0.83)	
			G3 = 2.6-5.0	• G4 = 0.54 (0.39-0.76)	
			G4 = 5.1-10.0	• G5 = 0.61 (0.44-0.84)	
			G5 > 10	*p *= 0.004	
			Time for VPA (min)	Vigorous exercise	
			G1 = None	• G1 = 1.00 (referent)	
			G2 = 1-60	• G2 = 1.12 (0.79-1.60)	
			G3 = 61-100	• G3 = 0.56 (0.32-0.98)	
			G4 = 101-150	• G4 = 0.73 (0.43-1.25)	
			G5 = >150	• G5 = 0.58 (0.34-0.99)	
				*p *= 0.008	
			Outcome Measure: Incidence of CVD and CHD		

Wisloff et al 2006 [58]	To study the association between the amount and intensity of exercise and CVD mortality.	• n = 56,072 (27,143 men; 28,929 women)	Length of follow-up: 16 ± 4 yr	• Number of Cases: 1,603 male, 993 female	Men and women who exercise to a moderate degree and spend less than the recommended energy (< 1000 kcal/wk) are at lower risk of dying from heart disease than those who never exercise.
Norway		• Sex: Men and women	PA assessment: Questionnaire for LTPA, 4 groups	Multivariate RR (95% CI)	
Prospective cohort		• Age: ≥ 20 yr		Men	
		• Characteristics: Free form CVD	Men	• Q1 = 1.00 (referent)	
			Q1 = None	• Q2 = 0.66 (0.50-0.87)	
D & B score = 12		• HUNT study	Q2 = 1/wk >30 min high	• Q3 = 0.83 (0.65-1.06)	
			Q3 = 2-3/wk > 30 min high	• Q4 = 0.77 (0.59-1.01)	
			Q4 = ≥ 4/wk > 30 min high	Women	
				• Q1 = 1.00 (referent)	
			Women	• Q2 = 0.63 (0.31-1.29)	
			Q1 = None	• Q3 = 0.66 (0.32-1.34)	
			Q2 = 1/wk ≤ 30 min low	• Q4 = 0.86 (0.45-1.62)	
			Q3 = 1/wk ≤ 30 min high		
			Q4 = 2-3/wk ≤ 30 min low		
			Outcome Measure: Ischaemic heart disease mortality		
			Cox proportional HR		

Lee et al 2001 [59]	To examine the relationship between PA (specifically walking) and CHD among women, including those at high risk for CHD.	• n = 39,372	Recruitment of Participants: Sept 1992-May 1995	• Number of Cases: 244	Even light to moderate activity is associated with lower CHD rates in women.
USA and Puerto Rico		• Sex: Women			
		• Age: ≥ 45 yr		Multivariate RR (95% CI) Time spent walking	
		• Characteristics: Healthy	PA assessment: Questionnaires Divided into 4 or 5 groups:	• G1 = 1.00 (referent)	
		• Women's Health Study		• G2 = 0.86 (0.57-1.29)	As little as 1 hour of walking per week predicted lower risk.
Prospective cohort				• G3 = 0.49 (0.28-0.86)	
				• G4 = 0.48 (0.29-0.78)	
				*p *= <0.001	
D & B score = 12			Time spent walking		
			G1 = No regular walking	Walking pace	
			G2 = 1-59 min/wk	• G1 = 1.00 (referent)	
			G3 = 1.0-1.5 h/wk	• G2 = 0.56 (0.32-0.97)	
			G4 = ≥ 2.0 h/wk	• G3 = 0.71 (0.47-1.05)	
			Walking pace (km/h)	• G4 = 0.52 (0.30-0.90)	
			G1 = No regular walking	*p *= 0.02	
			G2 = 3.2		
			G3 = 3.2-4.7		
			G4 = ≥ 4.8	EE (kcal/wk)	
				• Q1 = 1.00 (referent)	
			EE (kcal/wk)	• Q2 = 0.79 (0.56-1.12)	
			G1 = 200	• Q3 = 0.55 (0.37-0.82)	
			G2 = 200-599	• Q4 = 0.75 (0.50-1.12)	
			G3 = 600-1499 and	*p *= 0.03	
			G4 = 1500 or more		
				Energy expended VPA (kcal/wk)	
			Energy expenditure for VPA (kcal/wk)	• G1 = 1.00 (referent)	
			G1 = No vigorous, <200 kcal/wk	• G2 = 0.65 (0.46-0.91)	
			G2 = No vigorous, ≥ 200 kcal/wk	• G3 = 1.18 (0.79-1.78)	
				• G4 = 0.96 (0.60-1.55)	
				• G5 = 0.63 (0.38-1.04)	
			G3 = Vigorous, 1-199 kcal/wk		
			G4 = Vigorous, 200-499 kcal/wk		
			G5 = Vigorous, ≥ 500 kcal/wk		

Paffenbarger et al 1993 [67]	To analyze changes in the lifestyle of Harvard Alumni and the associations of these changes to mortality.	• n = 10,269	Baseline measure in 1962 or 1967 with a follow up in 1977	Alumni who increased their PA index to 2000 kcal or more per week had a 17% lower risk of death from CHD then those who were sedentary (*p *= 0.507)	Moderately vigorous sports activity was associated with lower rates of death from CHD among middle aged and older men
		• Sex: Men			
		• Age: 45-84 yr			
USA		• Characteristics: Health, Harvard College Alumni			
Prospective cohort			PA assessment: Mailed questionnaires included questions on type, duration, intensity, frequency of PA.	Men who took up moderate took up moderately vigorous activity had a 41% lower risk than those who continued not to engage in such activity (*p *= 0.044)	
D & B score = 13			Outcome Measure: CHD deaths between 1977 and 1985		
			Cox proportional hazards model		
			Poisson regression methods		
			The Mantel extension of the Mantel-Haenszel test		

Haapanen et al 1997 [77]	To examine the association between duration and intensity of LTPA and the risk of CHD.	• n = 2,840 (1,500 men; 1,340 women)	Length of Follow-up: 10 yrs	• Incident Rates (per 1000 person-years) for CHD = 108 for men and 75 for women.	Total EE had an inverse and independent association with risk of CHD in middle aged Finnish men but not among women.
Finland		• Sex: Men and women	PA assessment: Questionnaire for LTPA EE (kcal/wk)	Multivariate RR (95% CI) LTPA and CHD mortality	
Prospective cohort		• Age: 35-63 yr		Men	
		• Characteristics: Healthy	Men	• G1 = 1.98	
			G1 = 0-1100	• G2 = 1.33	
D & B score = 13			G2 = 1101-1900	• G3 = 1.00 (referent)	
			G3 = >1900		
				Women	
			Women	• G1 = 1.25	
			G1 = 0-900	• G2 = 0.73	
			G2 = 901-1500	• G3 = 1.00 (referent)	
			G3 = >1500		
			Outcome Measure: CHD mortality		
			Cox proportional HR		

Barengo et al 2004 [164]	To investigate whether moderate or high LTPA are associated with a reduced CVD and all-cause mortality, independent of CVD risk factors and other forms of PA in men and women.	• n = 31,677 (15,853 men; 16,824 women)	20 year follow-up	• Number of Cases (Men): 1,661	Moderate and high levels of LTPA and OPA are associated with reduced CVD mortality.
			PA assessment: Questionnaire for LTPA and OPA, 3 groups	• Number of Cases (Women): 778	
Finland		• Sex: Men and women		HR (95% CI) LTPA, men	
Prospective cohort		• Age: 30-59	G1 = Low activity	• G1 = 1.00 (referent)	
		• Characteristics: Participant from eastern and south-western Finland	G2 = Moderate activity	• G2 = 0.91 (0.82-1.00)	
D & B score = 14			G3 = High activity	• G3 = 0.83 (0.69-0.99)	
				LTPA, women (referent)	
				• G1 = 1.00	
				• G2 = 0.83 (0.71-0.96)	
				• G3 = 0.89 (0.68-1.18)	
				OPA, men	
				• G1 = 1.00 (referent)	
				• G2 = 0.75 (0.64-0.87)	
				• G3 = 0.77 (0.69-0.87)	
				OPA, women	
				• G1 = 1.00 (referent)	
				• G2 = 0.73 (0.60-0.88)	
				• G3 = 0.77 (0.65-0.91)	

Bijnen et al 1998 [166]	To describe the association between the PA pattern of elderly men and CHD mortality.	• n = 802	Length of Follow-up: 10	• Number of Cases: 90	PA did not show a protective effect on death from CHD.
		• Sex: Men			
		• Age: 64-84 yr	PA assessment: Questionnaire, divided into 3 groups	RR (95% CI)	
Netherlands		• Characteristics: Free from Serious Illness		• G1 = 1.00 (referent)	
				• G2 = 0.63 (0.38-1.05)	
Prospective cohort			G1 = Lowest	• G3 = 0.85 (0.51-1.44)	
		• Ethnicity: Dutch	G2 = Middle		
		• Zutphen Elderly Study	G3 = Highest		
D & B score = 13			Outcome Measure: CHD Mortality		
			Cox Proportional HR		

Davey-Smith et al 2000 [174]	To examine the association between two measures of physical activity (LTPA and usual walking pace) with cause specific mortality (CHD).	• n = 6,702	Length of Follow-up: 25 yrs	• Number of Cases: 955	Inverse associations of both LTPA and walking pace with mortality from CHD were seen.
		• Sex: Men			
		• Age: 40-64 yr		RR (95% CI) by walking pace	
England		• Whitehall Study	PA assessment: Questionnaire during examination for walking pace and LTPA	• G1 = 1.45 (0.9-2.2)	
				• G2 = 1.30 (1.1-1.6)	
Prospective cohort				• G3 = 1.00 (referent)	
				*p *< 0.01	
D & B score = 11			Walking pace	Multivariate RR (95% CI) by LTPA level	
			G1 = Slower	• G1 = 1.24 (1.0-1.5)	
			G2 = Same	• G2 = 0.94 (0.8-1.2)	
			G3 = Faster	• G3 = 1.00	
				*p *< 0.05	
			LTPA		
			G1 = Inactive		
			G2 = Moderate		
			G3 = Active		
			Outcome Measure: CHD Mortality		
			Cox Proportional HR		

Eaton et al 1995 [175]	To determine whether self reported PA predicts a decreased risk of CHD.	• n = 8,463 (LTPA), 8,418 (OPA)	Length of Follow-up: 21 yrs	• Number of Cases: 709	Baseline levels of self reported LTPA predicted a decreased rate of CHD.
				Age adjusted RR (95% CI) by LTPA level	
USA		• Sex: Men	PA assessment: Interview	• G1 = 1.00 (referent)	
		• Age: 40 yr		• G2 = 0.79 (0.63-0.99)	
Prospective cohort		• Characteristics: Healthy, free of CHD	LTPA	• G3 = 0.73 (0.59-0.89)	
			G1 = Sedentary	• G4 = 0.71 (0.52-0.98)	
			G2 = Light		
D & B score = 11		Ethnicity: Israeli	G3 = Light Daily	Age adjusted RR (95% CI) by OPA level	
			G4 = Heavy	• G1 = 1.00 (referent)	
				• G2 = 0.99 (0.75-1.18)	
			OPA	• G3 = 0.94 (0.78-1.12)	
			G1 = Sitting	• G4 = 0.87 (0.67-1.10)	
			G3 = Walking		
			G4 = Physical Labour		
			Outcome Measure: CHD Death		
			Cox Proportional HR		

Hillsdon et al 2004 [183]	To examine whether a short, easily administered measure of PA is associated with the risk of death from all causes and specific causes.	• n = 10,522 (4,929 men; 5,593 women)	Length of Follow-up: > 10 yrs	• Number of Cases: 155	Self reported VPA is associated with the risk of future mortality.
				Multivariate RR (95% CI) by PA level	
UK		• Sex: Men and women	PA assessment: Questionnaire, 3 groups:	• G1 = 1.00 (referent)	
				• G2 = 0.46 (0.19-1.12)	
Prospective cohort		• Age: 35-64 yr	G1 = Never / <1 time/month	• G3 = 0.96 (0.53-1.75)	
		• Characteristics: no history of chest pain			
			G2 = <2 times/wk		
D & B score = 11			G3 = ≥ 2 times/wk		
			Outcome Measure: IHD mortality		
			Cox proportional HR		

Leon et al 1997 [199]	To study the relationship of PA to CHD in a well defined population at above average risk for CHD over a 16 yr observation period.	• n = 12,138	Follow up for 16 years	Age Adjusted RR (95% CI)	A relatively small amount (10-36 min/d) of daily moderate intensity LTPA can significantly reduce premature mortality from CHD in middle aged men at high risk for CHD.
USA		• Sex: Men		• G1 = 1.00 (referent)	
		• Age: 35-57 yr	PA assessment: Questionnaire at baseline (Minnesota LTPA questionnaire), divided/grouped into deciles of LTPA (min/d)	• G2 = 0.71 (0.56-0.91)	
		• Characteristics: Free of CHD but in the upper 10-15% of a CHD probability risk score		• G3 = 0.75 (0.59-0.96)	
				• G4 = 0.69 (0.54-0.96)	
Prospective cohort				Multivariate adjusted RR (95% CI)	
				• G1 = 1.00 (referent)	
D & B score = 11			G1 = D1: (0-9 min/d)	• G2 = 0.75 (0.54-0.96)	
		Multiple risk factor intervention trial	G2 = D2-4: (10-36 min/d)	• G3 = 0.81 (0.64-1.04)	
			G3 = D5-7: (37-75 min/d)	• G4 = 0.75 (0.59-0.96)	
			G4 = D8-10: (76-359 min/d)		
			Outcome Measure: CHD Mortality		

Rosengren et al 1997 [211]	To examine the long term effect of OPA and LTPA on the risk of death from CHD.	• n = 7,142	Length of Follow-up: 20 yrs	Number of Cases: 684	There appears to be a protective effect of LTPA on CHD-related death.
		• Sex: Men			
		• Age: 47-55 yr		Multivariate RR (95% CI) for LTPA	
Sweden		• Characteristics: Swedish men	PA assessment: Questionnaire for LTPA, 3 groups	• G1 = 1.00 (referent)	
				• G2 = 0.84 (0.71-1.00)	
Prospective cohort				• G3 = 0.84 (0.73-0.96)	
			G1 = Sedentary		
			G2 = Moderately active		
D & B score = 13			G3 = Regular exercise		
			Outcome Measure: CHD death		
			Proportional HR		

Schnohr et al 2006 [214]	To describe the associations between different levels of LTPA and subsequent causes of death.	• n = 4,894 (2,136 men; 2,758 women)	Participants included in the study were only those whose PA levels did not change over 5 years	• Number of Cases: 292	There was an inverse and significant dose- response association between LTPA and CHD-related mortality.
				Adjusted RR (95% CI) Whole group	
Denmark		• Sex: Men and women		• G1 = 1.00 (referent)	
				• G2 = 0.71 (0.51-0.99)	
Prospective cohort		Age: 20--79 yr	PA assessment:	• G3 = 0.56 (0.38-0.82)	
		• Characteristics: Healthy	Questionnaire LTPA		
D & B score = 12		• Copenhagen City Heart Study	G1 = <4 METS	Men	
			G2 = 4-6 METS	• G1 = referent	
			G3 = >6 METS	• G2 = survived 4.9 yrs longer	
				• G3 = survived 6.8 yrs longer	
			Cox proportional HR		
				Women	
				• G1 = referent	
				• G2 = survived 5.5 yrs longer	
				• G3 = survived 6.4 yrs longer	

Weller et al 1998 [220]	To examine the relationship between PA and mortality.	• n = 6,620	Length of Follow-up: 7 yrs	• Number of Cases: 109	LTPA is inversely associated with risk of fatal MI.
		• Sex: Women			
		• Age: ≥ 30 yr	PA assessment:	OR (95% CI) by LTPA	
Canada		• Characteristics: Canadian Women	Questionnaire, 4 groups for LTPA (kcal/kg/day) and non-LTPA (kcal/kg/day)	• Q1 = 1.00 (referent)	
				• Q2 = 0.61 (0.07-1.19)	
Prospective cohort				• Q3 = 0.84 (0.52-1.37)	
				• Q4 = 0.63 (0.36-1.09)	
D & B score = 9			LTPA (kcal/kg/day)	OR (95% CI) by non-LTPA	
			Q1 = ≥ 0	• Q1 = 1.00 (referent)	
			Q2 = ≥ 0.1	• Q2 = 0.71 (0.44-1.16)	
			Q3 = ≥ 0.5	• Q3 = 0.57 (0.33-0.97)	
			Q4 = ≥ 1.6	• Q4 = 0.49 (0.26-0.92)	
			Non-LTPA (kcal/kg/day)		
			Q1 = ≥ 0		
			Q2 = ≥ 2.8		
			Q3 = ≥ 5.9		
			Q4 = ≥ 9.9		
			Outcome Measure: Fatal MI		
			Logistic regression analysis		

Yu et al 2003 [221]	To examine the optimal intensity of LTPA to decrease the risk of CHD mortality in middle aged British men.	• n = 1,975	10 year follow-up	• Number of Cases: 82	Strong significant inverse relationship between heavy LTPA and CHD mortality.
		• Sex: Men	PA assessment: Questionnaire (Minnesota LTPA questionnaire), 3 groups	Multivariate adjusted HR (95% CI)	
		• Age: 49-64 yr		• G1 = 1.00 (referent)	
UK		• Characteristics: Healthy, no previous history of CHD		• G2 = 0.74 (0.44-1.25)	
				• G3 = 0.55 (0.31-0.98)	
Prospective cohort				*p *= 0.039	Relationship was not significant for low- moderate intensity LTPA and OPA.
		• Caerphilly collaborative heart study	Total activity level (kcal/day)		
D & B score = 11			G1 = 0.0 - 161.6		
			G2 = 161.8 - 395.3		
			G3 = 395.5 - 2747.2		
			Cox proportional HR		

Altieri et al 2004 [222]	To assess the possible protective role of PA on CHD.	• n = 985 (507 men; 478 women)	PA assessment: Questionnaire for OPA, divided into quartiles	Number of Cases: 507	LTPA from 15-19 yrs as well as OPA from 30 - 39 yrs both have a significant inverse relationship with risk of non fatal acute MI.
				OR (95% CI) for CHD and OPA	
Italy		• Sex: Men and women	Q1 = lowest	• Q1 = 1.00 (referent)	
			Q2	• Q2 = 0.63 (0.39-1.03)	
Case Control		• Age: < 79 yr	Q3	• Q3 = 0.56 (0.35-0.90)	
		• Characteristics: Case: Patients admitted to Hospital with non-fatal Acute MI. Controls: Patients admitted to hospital for acute condition unrelated to known or potential risk factors for acute MI	Q4 = highest	• Q4 = 0.57 (0.34-0.95)	
D & B score = 11				*p *= 0.045	
			Outcome Measure: Non Fatal acute MI		
			Unconditional logistic regression		

Batty et al 2003 [223]	To examine the relationship between physical activity and three mortality endpoints in healthy persons.	• n = 6,474	Length of Follow-up: 25 yr	• Number of Cases: 837	A suggestion that the symptomatic nature of ischemia appeared to modify the affects of
		• Sex: Men		• Number of Dropouts: 158	
		• Age: 40-64 yr	PA assessment: Questionnaire for LTPA, divided into 3 groups:		
UK		• Characteristics: British civil servants who underwent a resting ECG		HR (95% CI) for CHD and LTPA	
				• G1 = 1.14 (0.9-1.4)	PA on total and CHD mortality.
Prospective cohort			G1 = Inactive	• G2 = 0.94 (0.8-1.1)	
			G2 = Moderate	• G3 = 1.00 (referent)	
			G3 = Active		
D & B score = 13					
			Outcome Measure: CHD mortality		
			Cox Proportional HR		

Chen and Millar [224]	To examine the potential protective effect of LTPA on the incidence of heart disease and depression.	• n = 15,670	Length of Follow-up: 2 yrs	• 100 cases	Regular and at least MPA can be beneficial to heart health.
		• Sex: Men and women			
		• Age: ≥ 20 yr	PA assessment: EE from self administered questionnaire, 4 groups (kcal/kg/day)	Adjusted OR (95% CI)	
Canada		• Characteristics: Healthy and free from heart disease		• G1 = 5.0 (1.84-13.59)	
				• G2 = 3.7 (1.26-10.67)	
Prospective cohort				• G3 = 1.00 (referent)	
			G1 = Sedentary	• G4 = 1.3 (0.41-3.89)	
			G2 = Light (<1.5)		
D & B score = 11		National Population Health Survey	G3 = Moderate (1.5-2.9)		
			G4 = Active (≥ 3)		
			Outcome Measure: CHD incidence		
			Multiple logistic regression		

Conroy et al 2005 [225]	To examine the relationship between 1) PA during young adulthood and middle age, and 2) PA during each time period and CHD during middle age and older women.	• n = 37,169	Length of Follow-up: 9 yrs	• Number of Cases: 477	PA during middle age predicts lower risk of CHD
		• Sex: Women			
		• Age: ≥ 45 yr		Multivariate RR (95% CI) Baseline PA and incidence of CHD	
US		• Characteristics: Healthy women health professionals	PA assessment: Questionnaire for EE (kcal/wk) and months/yr		
		• Women's Health Study		• G1 = 1.00 (referent)	
Cohort study				• G2 = 0.62 (0.48-0.80)	
				• G3 = 0.61 (0.48-0.79)	
D & B score = 11			Baseline PA (kcal/wk)	• G4 = 0.61 (0.46-0.81)	
			G1 = <200	*p *= <0.001	
			G2 = 200-599		
			G3 = 600-1499	Past PA and incidence of CHD	
			G4 = ≥ 1500	• G1 = 1.00 (referent)	
				• G2 = 0.76 (0.57-1.02)	
			Past PA	• G3 = 0.95 (0.72-1.24)	
			Months per year	• G4 = 1.04 (0.78-1.39)	
			G1 = 0	• G5 = 0.81 (0.58-1.14)	
			G2 = 1-3		
			G3 = 4-6		
			G4 = 7-9		
			G5 = 10-12		
			Outcome Measure: Incidence of CHD		
			Cox proportional hazard regression		

Dorn et al 1999 [226]	To examine the long-term relationships between total PA and mortality from all causes and CHD in the general population.	• n = 1,461 (698 men; 763 women)	Length of Follow-up: 29 years	• Number of Cases: 109 men, 81 women	PA favorably influences mortality risks in non- obese men and younger women.
USA		• Sex: Men and women	PA assessment: Questionnaire	Multivariate RR (95% CI) for PAI in non- obese men	
Prospective cohort		• Age: 15-96 yr		• 0.40 (0.19-0.88) for 1 kcal/kg/h	
		• Characteristics:	Outcome Measure: CHD	Multivariate RR (95% CI) for PAI in obese men	
		Healthy, free from CHD, diabetes, and Stroke.	Mortality	• 1.86 (0.86-4.03) for 1 kcal/kg/h	
D & B score = 11					
			Cox Proportional Hazard		
			Ratio	Multivariate RR (95% CI) for PAI in women < 60 yrs	
		• Ethnicity: White.		• 0.42 (0.11-1.52) for 1 kcal/kg/h	
				Multivariate RR (95% CI) for PAI in women > 60 yrs	
				• 1.78 (0.77-4.09) for 1 kcal/kg/h	

Folsom et al 1997 [227]	To examine the association of PA at baseline with CHD incidence.	• n = 13,999 (6,166 men; 7833 women)	Length of Follow-up: 4-7 yrs	• Number of Cases: 223 men, 97 women,	No significant relationships.
				Multivariate RR (95% CI) LTPA, men	
USA		• Sex: Men and women	PA assessment: Questionnaire during home interview, divided into quartiles of LTPA and sports activity		
				• Q1 = 1.00 (referent)	
Prospective cohort		• Age: 45-64 yr		• Q2 = 1.08 (0.75-1.55)	
		• Characteristics: no CHD at baseline		• Q3 = 0.83 (0.51-1.36)	
				• Q4 = 0.89 (0.59-1.35)	
D & B score = 9			Q1 = Low		
		• Ethnicity: Black and non Black	Q2	LTPA, women	
			Q3	• Q1 = 1.00 (referent)	
		• Atherosclerosis Risk in Communities Study	Q4 = High	• Q2 = 0.74 (0.42-1.31)	
				• Q3 = 1.07 (0.55-2.09)	
			Outcome Measure: CHD incidence Poisson Regression	• Q4 = 0.64 (0.34-1.24)	
				Multivariate RR (95% CI) Sports, men	
				• Q1 = 1.00 (referent)	
				• Q2 = 1.15 (0.79-1.68)	
				• Q3 = 1.03 (0.68-1.54)	
				• Q4 = 0.83 (0.56-1.23)	
				Sports, women	
				• Q1 = 1.00 (referent)	
				• Q2 = 0.99 (0.58-1.67)	
				• Q3 = 0.64 (0.32-1.27)	
				• Q4 = 0.72 (0.37-1.38)	

Fransson et al 2004 [228]	To estimate the influence of LTPA and OPA on acute MI.	• n = 4069 (2,742 men; 1,327 women)	PA assessment: Questionnaire for LTPA, 5 groups	• Number of Cases: 1,204 men, 550 women	Exercise seems to reduce the risk of MI.
Sweden		• Sex: Men and Women	G1 = Seldom	OR (95% CI)	
			G2 = Sometimes		
Case Control		• Age: 45-70 yr	G3 = 1×/wk	LTPA, men	
		• Characteristics: Cases: Diagnosed with acute MI	G4 = 2-3×/wk	• G1 = 1.00 (referent)	
D & B score = 12			G5 = >3×/wk	• G2 = 0.76 (0.61-0.95)	
				• G3 = 0.67 (0.51-0.88)	
				• G4 = 0.63 (0.49-0.83)	
		• Stockholm Heart Epidemiology	Questionnaire for total physical activity, 3 groups	• G5 = 0.53 (0.38-0.73)	
			G1 = Passive		
			G2 = Somewhat active	LTPA, women	
			G3 = Active	• G1 = 1.00 (referent)	
			Questionnaire for sitting at work, 3 groups	• G2 = 0.69 (0.49-0.98)	
				• G3 = 0.38 (0.25-0.58)	
			G1 = Less than half the time	• G4 = 0.62 (0.38-1.01)	
			G2 = About half the time	• G5 = 0.31 (0.15-0.66)	
			G3 = More than half the time	Total physical activity, men	
				• G1 = 1.00 (referent)	
				• G2 = 0.66 (0.47-0.94)	
			Outcome Measure: Acute MI	• G3 = 0.46 (0.31-0.69)	
				Total physical activity, women	
			Conditional and unconditional logistics regression	• G1 = 1.00 (referent)	
				• G2 = 0.34 (0.22-0.53)	
				• G3 = 0.16 (0.07-0.37)	
				Sitting at work, men	
				• G1 = 1.00 (referent)	
				• G2 = 0.91 (0.73-1.15)	
				• G3 = 0.90 (0.72-1.12)	
				Sitting at work, women	
				• G1 = 1.00 (referent)	
				• G2 = 0.77 (0.51-1.17)	
				• G3 = 0.47 (0.31-0.69)	

Fransson et al 2006 [229]	To evaluate whether LTPA compensates for the increased risk of acute MI associated with overweight and obesity.	• n = 4069 (2,742 men; 1,327 women)	PA Assessment: Questionnaire for LTPA, 3 groups	Number of Cases: 1204 men, 550 women	Regular LTPA seems to provide protection against MI and non- fatal MI.
				Multivariate OR (95% CI) for acute MI	
Sweden		• Sex: Men and women	G1 = Very little /occasional walks	LTPA, men	
				• G1 = 1.00 (referent)	
Case Control		• Age: 45-70 yr	G2 = Occasional / once per week	• G2 = 0.70 (0.58-0.84)	
		• Characteristics: Cases: had acute MI		• G3 = 0.57 (0.46-0.71)	
D & B score = 12			G3 = Twice per week or more	LTPA, women	
				• G1 = 1.00 (referent)	
			Outcome measure: Acute MI	• G2 = 0.52 (0.40-0.68)	
				• G3 = 0.44 (0.30-0.65)	
				Multivariate OR (95% CI) for non-fatal MI	
			Conditional and unconditional logistics regression	LTPA, men	
				• G1 = 1.00 (referent)	
				• G2 = 0.79 (0.65-0.96)	
				• G3 = 0.63 (0.50-0.79)	
				LTPA, women	
				• G1 = 1.00 (referent)	
				• G2 = 0.64 (0.48-0.86)	
				• G3 = 0.58 (0.39-0.87)	

Haapanen-Niemi 2000 [230]	To investigate the independent associations and the possible interaction of BMI LTPA and perceived physical performance and functional capacity with the risk of mortality.	• n = 2,212 (1,090 men; 1,122 women)	Length of Follow-up: 16 yrs	• Number of Cases: 208 all cause deaths, 54% of those CVD. 73% of CVD deaths due to CHD	Increase perceived PF is associated with a reduced risk of CHD mortality in men.
Finland		• Sex: Men and women	PA assessment: Postal Survey		
				Multivariate RR (95% CI)	
Prospective cohort		• Age: 35-63 yr	Total LTPA energy expenditure (kcal/wk)	Total LTPA EE index and CHD mortality, men	
		• Characteristics: Healthy		• G1 = 1.00 (referent)	
			G1 = High	• G2 = 0.88 (0.44-1.76)	
D & B score = 13		• Ethnicity: Finnish	G2 = Moderate	• G3 = 1.70 (0.90-3.21)	
			G3 = Low	*p *= 0.056	
			Perceived physical fitness compared to age-mates	Multivariate RR (95% CI) Perceived physical fitness, men	
			G1 = Better	• G1 = 1.00 (referent)	
			G2 = Similar	• G2 = 2.82 (1.06-7.46)	
			G3 = Worse	• G3 = 4.64 (1.56-13.84)	
			Outcome Measure: CHD mortality	*p *= 0.011	
				Total LTPA EE index and CHD mortality, women	
			Cox proportional HR		
				• G1 = 1.00 (referent)	
				• G2 = 0.43 (0.16-1.16)	
				• G3 = 1.17 (0.51-2.68)	
				*p *= 0.046	
				Multivariate RR (95% CI) Perceived physical fitness, women	
				• G1 = 1.00 (referent)	
				• G2 = 0.82 (0.32-2.16)	
				• G3 = 1.89 (0.57-6.27)	
				*p *= 0.154	

Kannel et al 1986 [231]	To examine the role of low levels of OPA and LTPA in the development of CV morbidity and mortality over the short and long term.	• n = 1,166	Length of Follow-up: 24 yrs	• Number of Cases: 220 mortality, 371 morbidity	Rate of CHD Mortality and Morbidity decreases with increased level of PA but no association was found with physical demand of work
		• Sex: Men			
		• Age: 45-65 yr			
USA		• Characteristics:	PA assessment: Questionnaire during examination	Cumulative 24 year age adjusted rate per 1000 people	
Prospective cohort				24 hr PA index for LTPA CHD mortality	
			PA index:	• G1 = 255	
D & B score = 11			G1 = <29	• G2 = 184	
			G2 = 30-34	• G3 = 152	
			G3 = >34	*p *< 0.01	
			Physical demand of work	24 hr PA index for LTPA CHD incidence	
			G1 = Sedentary	• G1 = 414	
			G2 = Light	• G2 = 353	
			G3 = Medium	• G3 = 311	
			G4 = Heavy		
			Outcome Measure: CHD mortality and Morbidity	Physical demand of work and CHD mortality	
				• G1 = 216	
			Cox proportional HR	• G2 = 209	
				• G3 = 169	
				• G4 = 170	
				Physical demand of work and CHD incidence:	
				• G1 = 355	
				• G2 = 405	
				• G3 = 307	
				• G4 = 325	

Kaprio et al 2000 [232]	To examine the contribution of genetic and other familial factors to the relationship between LTPA and CHD.	• n = 8,205	Length of Follow-up: 18 yrs	• Number of Cases: 723	LTPA compared to being sedentary helps prevent CHD in men.
		• Sex: Men			
		• Age: 25-69 yr		Multivariate RR (95% CI)	
Finland		• Characteristics: Same sex twin pairs, free of CVD	PA assessment: Questionnaire for LTPA, 3 groups:	• G1 = 1.00 (referent)	
				• G2 = 0.84 (0.70-1.01)	
Prospective cohort				• G3 = 0.68 (0.50-0.92)	
			G1 = Sedentary	*p *= 0.010	
			G2 = Occasional		
D & B score = 12			Exercisers		
			G3 = Conditioning		
			Exercisers		
			Outcome Measure: Hospitalization or death from CHD		
			Poisson regression		

Lakka et al 1994 [233]	To investigate the independent associations of LTPA and maximal oxygen uptake with the risk of acute MI.	• n = 1,166	Baseline examination: 1984-1989		Conditioning LTPA and VO_2 _max had an inverse, graded and independent association with the risk
		• Sex: Men			
		• Age: 42-61 yr		Adjusted RH (95% CI) by conditioning PA level	
Finland		• Characteristics: Healthy with normal ECG	PA assessment: Questionnaire for conditioning PA (h/wk), 3 groups (h/wk)		
			G1 = <0.7	• G1 = 1.00 (referent)	
Prospective cohort			G2 = 0.7	• G2 = 1.11 (0.58-2.12)	
		• Kuopio Ischaemic Heart Disease Risk Factor Study	G3 = >2.2	• G3 = 0.31(0.12-0.85)	
D & B score = 13				Adjusted RG (95% CI) by VO_2 _max	
				• G1 = 1.00	
			PF assessment: VO_2 _max (ml/kg/min)	• G2 = 0.76 (0.38-1.50)	
				• G3 = 0.26 (0.10-0.68)	
			G1 = <28.0		
			G2 = 28.0-33.6		
			G3 = >33.6		
			Outcome event: acute MI		
			Cox proportional HR		

Laukkanen at al 2004 [234]	To determine whether VO_2peak _predicts CVD morbidity and mortality in a sample of men as related to conventional risk factors, medications or underlying chronic disease.	• 1,294 healthy; 1,057 unhealthy	PF Assessment: VO_2 _peak (ml/kg/min) measured by exercise test with an electrically braked cycle ergometer, divided into quartiles	• Number of Cases: 204 CV deaths, 323 non-fatal coronary events	Dose-response relationship between directly measured PF and CVD death among healthy men at baseline.
Finland		• Sex: Men		Healthy men with low VO_2 _peak (lowest quartile) had an increased risk	
		• Age: 42-60 yr			
Prospective cohort		• Characteristics: Healthy and not healthy participants			
			Q1 = <27.6	Adjusted RR (95% CI) by PF quartile Fatal MI	
			Q2 = 27.6-32.2		Unfit men with unfavorable risk profiles are the risk group that would benefit the most from preventative measures.
D & B score = 11			Q3 = 32.3-37.1	• 3.29 (0.86-12.90)	
		• Kuopio Ischaemic Heart Disease Risk Factor Study	Q4 = >37.2		
				Non-Fatal MI	
			Outcome Measure: Incidence of fatal and non fatal CVD during 13 year follow-up	• 2.16 (1.12-4.18)	
			Cox proportional HR		

Lee at al 2000 [235]	To investigate whether different durations of exercise episode are associated with different risk of CHD.	• n = 7,307	Baseline survey in 1988	• Number of Cases: 482	Longer durations of PA bouts are not associated with decreased CHD risk compared with shorter bouts, once total EE is taken into account.
		• Sex: Men			
USA		• Age: Mean 66.1 ± 7.5	PA assessment: Survey for EE (kJ/wk), divided into 5 groups and episodes of PA (min), divided into 6 groups	Multivariate adjusted RR (95% CI) by EE	
				• G1 = 1.00 (referent)	
		• Characteristics: Healthy		• G2 = 0.80 (0.57-1.12)	
				• G3 = 0.80 (0.55-1.16)	
Prospective cohort		• Harvard Alumni Study		• G4 = 0.74 (0.47-1.17)	
				• G5 = 0.62 (0.41-0.94)	
D & B score = 12			Energy expenditure (kJ/wk)		As long as the total EE is similar, more frequent shorter bouts or longer less frequent bouts have an equivalent reduction in CHD risk.
			G1 = <4,200	Multivariate adjusted RR (95% CI) by duration of PA episode	
			G2 = 4,200-8,399		
			G3 = 8,400-12,599		
			G4 = 12,600-16,799	• G1 = 1.00 (referent)	
			G5 = ≥ 16,800	• G2 = 1.15 (0.70-1.87)	
				• G3 = 1.01 (0.68-1.51)	
				• G4 = 1.11 (0.67-1.84)	
			Duration of PA episode (min)	• G5 = 1.18 (0.77-1.80)	
			G1 = None	• G6 = 1.25 (0.83-1.87)	
			G2 = 1-15		
			G3 = 16-30		
			G4 = 31-45		
			G5 = 46-60		
			G6 = >60		
			Outcome Measure: Fatal and Non Fatal CHD		
			Proportional hazards regression		

Lee et al 2003 [236]	To investigate whether moderate- intensity exercise is associated with reduced CHD.	• n = 7,337	PA assessment: Survey rating usual level of exertion when exercising, divided into tertiles	• Number of Cases: 551	Inverse association between relative intensity of PA and the risk of CHD.
USA		• Sex: Male		Multivariate adjustment RR (95% CI)	
		• Age: Mean 66.1 yr		• T1 = 1.00 (referent)	
				• T2 = 0.87 (0.70-1.09)	
		• Characteristics: Healthy		• T3 = 0.92 (0.75-1.14)	
Prospective cohort			Energy expenditure (kcal/wk)		
		Harvard Alumni Study			
			T1 = <1000		
D & B score = 13			T2 = 1000-2499		
			T3 = ≥ 2500		
			Cox proportional HR		

Lemaitre et al 1999 [237]	To investigate whether regular participation in moderate intensity activity confers overall protection from sudden primary cardiac arrest.	• n = 355 cases, 503 controls	PA assessment: Interview (with spouses) for LTPA, 7 groups	• 355 cases	Participation in moderate intensity LTPA was associated with a decreased risk of primary cardiac arrest.
		• Sex: Men and women		RR (95% CI)	
USA			G1 = No activity	• G1 = 1.00 (referent)	
		• Age: 25-74 yr	G2 = Gardening only≤ 60 min/wk	• G2 = 0.52 (0.21-1.28)	
Case control		• Characteristics: Previously healthy prior to primary cardiac arrest. Control Subjects: Individually matched to case patients on age (within 7 years) and sex at a ratio of about 2:1 were randomly selected from community by random-digit dialing	G3 = Gardening only > 60 min/wk	• G3 = 0.34 (0.13 0.89)	
			G4 = Walking ≤ 60 min/wk	• G4 = 0.45 (0.17-1.19)	
D & B score = 11			G5 = Walking > 60 min/wk	• G5 = 0.27 (0.11-0.67)	
			G6 = Moderate intensity	• G6 = 0.31 (0.13-0.74)	
			LTPA (not walking or gardening)	G7 = 0.34 (0.16-0.75)	
			G7 = High intensity LTPA		
			Logistic regression analysis		

Lemaitre et al 1995 [238]	To examine whether LTPA decreases the risk of MI in postmenopausal women.	• n = 1,193	PA assessment: Phone interview for LTPA, divided into quartiles of EE (mean kcal/wk)	• Number of Cases: 268	Risk of MI among postmenopausal women is decreased by 50% with modest LT energy expenditures, equivalent to 30-45 min of walking for exercise three times per week
		• Sex: Women			
		• Age: Mean 67 yr		Multivariate RR (95% CI)	
USA				• Q1 = 1.00 (referent)	
		• Characteristics: Postmenopaus al Cases: Diagnosed with non-fatal MI Controls: free from MI	Q1 = 71	• Q2 = 0.52 (0.34-0.80)	
Case control			Q2 = 472	• Q3 = 0.40 (0.26-0.63)	
			Q3 = 1183	• Q4 = 0.40 (0.25-0.63)	
D & B score = 11			Q4 = 3576	*p *= <0.001	
			Outcome Measure: Diagnosed with non-fatal MI		
			Logistic regression analysis		

Li et al 2006 [239]	To examine independent and joint associations of PA and adiposity with CHD incidence.	• n = 88,393	Length of Follow-up: 20 yrs	• Number of Cases: 2,358	Physical inactivity independently contributes to the development of CHD in women.
		• Sex: Women		• Number of Dropouts: <2% lost to follow-contributes to the development of CHD in women.	
USA		• Age: 34-59 yr		up	
		• Characteristics: Nurses	PA assessment: Questionnaire for LTPA (hr/wk), 3 groups		
Prospective cohort				Multivariate HR (95% CI)	
		• Nurses' Health Study		• G1 = 1.00 (referent)	
			G1 = ≥3.5	• G2 = 1.34 (1.18-1.51)	
D & B score = 12			G2 = 1-3.49	• G3 = 1.43 (1.26-1.63)	
			G3 = <1		
			Outcome Measure: CHD incidence		
			Cox proportional HR		

Lemaitre et al 1995 [240]	To evaluate the effect of PA on MI occurrence.	• n = 1,107 (726 controls, 381 cases)	PA assessment: Questionnaire, 3-5 groups depending on variable	OR (95% CI),	PA level was inversely associated with occurrence of MI in both sexes, although the association presented a significant linear trend only for women; in men it suggested a u-shaped relation.
				Total PA, men	
Portugal		• Sex: Men and women		• G1 = 1.00 (referent)	
			Total PA (MET hr/day), men	• G2 = 0.54 (0.33-0.88)	
Case control		• Age: ≥ 40 yr		• G3 = 0.34 (0.20-0.59)	
		• Characteristics: Case: Admitted to Hospital and diagnosed with first episode of MI Control: Healthy, no history of CHD	G1 = 28.3-32.1	• G4 = 0.59 (0.36-0.98)	
D & B score = 12			G2 = 32.2-33.3	• G5 = 0.90 (0.56-1.45)	
			G3 = 33.4-36.5	Trend *p *= 0.827	
			G4 = 36.6-40.3	Total PA, women	
			G5 = 40.4-83.1	• Q1 = 1.00 (referent)	
			Total PA (MET hr/day), women	• Q2 = 0.39 (0.21-0.73)	
			Q1 = 28.9-32.7	• Q3 = 0.33 (0.17-0.64)	
			Q2 = 32.8-34.1	• Q4 = 0.22 (0.11-0.47)	
			Q3 = 34.2-37.8	*p *= <0.001	
			Q4 = 37.8-70.6		
				Sport participation, men	
			Sport participation (MET hr/day), men	• G1 = 1.00 (referent)	
			G1 = 0.0	• G2 = 0.36 (0.19-0.69),	
			G2 = 0.1-1.0	• G3 = 0.72 (0.41-1.26),	
			G3 = 1.1-2.0	• G4 = 0.42 (0.23-0.76),	
			G4 = 2.1-3.6	• G5 = 0.31 (0.16-0.62)	
			G5 = 3.7-15.4	*p *= <0.001	

Lovasi et al 2007 [241]	To investigate the shape of the relationship between LTPA and MI risk.	• n = 4,094	PA assessment: Telephone interview (Minnesota LTPA Questionnaire)	• Number of Cases: 697	Time engaged in LTPA, even non strenuous LTPA was associated with a lower risk of MI, and the shape of this relationship was non- linear
		• Sex: Men and women		Adjusted OR (95% CI)	
USA		• Age: 64 ± 9 yr		LTPA and non fatal CHD	
		• Characteristics: Group Health Cooperative Members		• G1 = 1.00 (referent)	
Case control			LTPA	• G2 = 0.88 (0.66-1.17)	
			G1 = None	• G3 = 0.62 (0.46-0.83)	
D & B score = 11			G2 = <2	• G4 = 0.61 (0.45-0.82)	
			G3 = 2-5	• G5 = 0.59 (0.44-0.80)	
			G4 = 5-9		
			G5 = >9 h/wk	Adjusted RR (95% CI) Strenuous LTPA and non Fatal CHD	
			Strenuous LTPA	• G1 = 1.00 (referent)	
			G1 = None	• G2 = 0.76 (0.59-0.99)	
			G2 = non strenuous LTPA	• G3 = 0.53 (0.40-0.70)	
			G3 = Any Strenuous		
			LTPA		
			Outcome measure: non fatal CHD		
			Logistic regression		

Manson et al 1999 [242]	To assess the comparative roles of walking and vigorous exercise in the prevention of coronary events in women.	• n = 72,488	PA assessment:	• Number of Cases: 645 coronary events	Both walking and VPA are associated with a substantial reductions in incidence of CHD. Risk reductions for each were similar hen total PAy was similar. Walking 3 or more hours per week could reduce the risk of CHD by 30-40%.
		• Sex: Women	Questionnaire with detailed information on PA.		
		• Age: 40-65 yr		Multivariate RR (95% CI) by total PA score	
USA		• Characteristics: Healthy, no Previous history of CHD		• G1 = 1.00 (referent)	
				• G2 = 0.88 (0.71-1.10)	
Prospective cohort			Total PA score	• G3 = 0.81(0.64-1.02)	
			G1 = 1-2.0	• G4 = 0.74 (0.58-0.95)	
		Nurses' Health Study	G2 = 2.1-4.6	• G5 = 0.66 (0.51-0.86)	
D & B score = 12			G3 = 4.7-10.4	*p *= 0.002	
			G4 = 10.5-21.7		
			G5 = >21.7		
				Multivariate RR (95% CI) by walking activity	
				• G1 = 1.00 (referent)	
			Walking, in those who did not participate in VPA: (MET hr/wk)	• G2 = 0.78 (0.57-1.06)	
			G1 = 0.5	• G3 = 0.88 (0.65-1.21)	
			G2 = 0.6-2.0	• G4 = 0.70 (0.51-0.95)	
			G3 = 2.1-3.8	• G5 = 0.65 (0.47-0.91)	
			G4 = 3.9-9.9	*p *= 0.02	
			G5 = ≥ 10		
				Multivariate RR (95% CI) by walking pace	
				• 1.00 (referent)	
			Walking pace (mph)	• 0.75 (0.59-0.96)	
			G1 = <2.0	• 0.64 (0.47-0.88)	
			G2 = 2.0-2.9		
			G3 = ≥ 3.0		

Mora et al 2007 [243]	To investigate whether differences in several CV risk factors mediate the effect of PA on reduced risk of CVD.	• n = 27,055	10.9 ± 1.6 yr of follow up	• Number of Cases: 640	There remained a borderline significant inverse association between PA and risk of CHD after adjustment for all sets of risk factors.
		• Sex: Women			
		• Age: ≥ 45 yr	PA assessment: Questionnaires at study entry for categories of EE from PA (kcal/wk), 4 groups	HR (95% CI), basic model	
USA		• Characteristics: Healthy		• G1 = 1.00 (referent)	
				• G2 = 0.84 (0.67-1.06)	
Prospective cohort		• Women's health study		• G3 = 0.76 (0.61-0.96)	
				• G4 = 0.62 (0.48-0.82)	
			G1 = <200	*p *= 0.001	
D & B score = 13			G2 = 200-599		While all sets of risk factors should some mediation on the effect of PA on CHD none made the relationship insignificant
			G3 = 600-1499	Multivariate adjusted HR (95% CI)	
			G4 = ≥ 1500	• G1 = 1.00 (referent)	
				• G2= 0.71 (0.58-0.87)	
			Outcome measure:	• G3 = 0.64 (0.52-0.78)	
			Incidence of CVD and	• G4 = 0.48 (0.38-0.62)	
				*p *= <0.001	
			Cox proportional HR		

O'Connor et al 1995 [244]	To examine the association between intensity of exercise and CHD risk.	• n = 680 (532 men and 148 women)	PA assessment: Home interview for PA, divided into quartiles	• Number of Cases: 340	Significant inverse association between PA level and the risk of non fatal MI in men, which persisted after adjustment for other risk factors.
				Adjusted OR (95% CI) by PA level, men	
				• Q1 = 1.00 (referent)	
USA		• Sex: Men and women	Q1 = Lowest	• Q2 = 0.60 (0.32-1.13)	
			Q2	• Q3 = 0.41 (0.21-0.78)	
Case control		• Age: < 76 yr	Q3	• Q4 = 0.41 (0.22-0.77)	
		• Characteristics: Cases: Diagnosed MI (non-fatal), no previous history of CHD. Controls: no history of CHD.	Q4 = Highest	*p *= 0.003	
D & B score = 12			Outcome Measure: non-fatal MI	Adjusted OR (95% CI) by PA level, women	
				• Q1 = 1.00 (referent)	
			Moderate- vigorous sports men Cut-points kcal/wk	• Q2 = 1.07 (0.27-4.17)	
			Q1 = Lowest	• Q3 = 2.02 (0.56-7.38)	
			Q2	• Q4 = 1.29 (0.31-5.35)	
			Q3	*p *= 0.51	
			Q4 = Highest		
				Adjusted OR (95% CI) by moderate-vigorous sports, men	
				• Q1 = 1.00 (referent)	
			Moderate- vigorous sports Women	• Q2 = 1.12 (0.60-2.10)	
			Cut-points kcal/wk	• Q3 = 0.61 (0.30-1.24)	
			Q1 = Lowest	• Q4 = 0.43 (0.20-0.92)	
			Q2	*p *= 0.02	
			Q3		
			Q4 = Highest	Adjusted OR (95% CI) by moderate-vigorous sports, women	
			Logistic regression analysis	• Q1 = 1.00 (referent)	
				• Q2 = 1.31 (0.37-4.66)	
				• Q3 = 1.90 (0.44-8.28)	
				• Q4 = 0.35 (0.07-1.84)	
				*p *= 0.62	

Rastogi et al 2004 [245]	To examine the relation between PA and CHD risk in India.	• n = 1,050	PA assessment: Questionnaire	Number of Cases: 350	Observed a strong and dose dependent inverse association between LTPA and non fatal CHD.
		• Sex: Men and women		Multivariate OR (95% CI) by LTPA	
USA		• Age: 21-74 yr	LTPA (MET min/d)	• G1 = 1.00 (referent)	
		• Characteristics: Cases: Diagnosed with MI (non fatal) Controls: non- cardiac patients	G1 = 0	• G2 = 0.96 (0.59-1.55)	
Case control			G2 = 0-145	• G3 = 0.44 (0.27-0.71)	
D & B score = 12			G3 = ≥145	*p *= 0.001	
			Sedentary time (min/d)	Multivariate OR (95% CI) by sedentary time	
			G1 = <70	• G1 = 1.00 (referent)	
			G2 = 70-130	• G2 = 1.15 (0.68-1.95)	
			G3 = 130-215	• G3 = 1.04 (0.61-1.76)	
			G4 = ≥215	• G4 = 1.88 (1.09-3.21)	
				*p *= 0.02	
			Outcome Measure: Non-fatal MI		
			Conditional logistic regression		

Rodriguez et al 1994 [246]	To examine the relationship between PA and 23 yr incidence of CHD morbidity and mortality.	• n = 7,074	23 year follow-up	• Number of Cases: 789	PA was associated with a significant reduction in the risk of CHD morbidity and mortality.
		• Sex: Men			
		• Age: 45-64 yr	PA assessment: Questionnaire for PA index, divided into tertiles	Age adjusted RR (95% CI), CHD incidence	
USA		• Characteristics: Japanese- American living in Oahu, Hawaii in 1965, < 65 years to reduce effect of retirement on PA levels		• T1 = 1.00 (referent)	
				• T2 = 1.01 (.86-1.19)	
Prospective cohort			T1 = Low	• T3 = 0.83 (0.86-1.19)	These data support the hypothesis that PA is associated with a favorable profile of CVD risk factors.
			T2 = Moderate		
			T3 = High	Multivariate adjusted RR (95% CI), CHD incidence	
D & B score = 11			Cox proportional regression model	• T1 = 1.00 (referent)	
				• T2 = 1.07 (0.90-1.26)	This study did not show a dose- response relationship since the medium tertile of PA showed increased rates of CHD compared to the inactive group.
		• The Honolulu Heart Program		• T3 = 0.95 (0.80-1.14)	
				Age adjusted RR (95% CI), CHD mortality	
				• T1 = 1.00 (referent)	
				• T2 = 1.12 (0.88-1.44)	
				• T3 = 0.74 (0.56-0.97)	
				Multivariate adjusted RR (95% CI)	
				• T1 = 1.00 (referent)	
				• T2 = 1.19 (0.93-1.53)	
				• T3 = 0.85 (0.65-1.13)	

Rothenbacher et al 2003 [247]	To estimate the risk for CHD associated with LTPA.	• n = 791 (312 cases; 479 controls)	PA assessment: Interview	Number of Cases: 312	LTPA showed a clear inverse association with risk of CHD.
			LTPA (h/wk)	Multivariate OR (95% CI), LTPA	
Germany		• Sex: Men and Women	G1 = 0	Winter	
			G2 = <1	• G1 = 1.00 (referent)	
Case control		Age: 40-68 yr	G3 = 1-2	• G2 = 0.48 (0.27-0.84)	
		Characteristics: Cases: stable CHD diagnosed within 2 years, no recent MI, Controls: no history of CHD.	G4 = >2	• G3 = 0.54 (0.369-0.82)	
D & B score = 12				• G4 = 0.27 (0.19-0.47)	
			Workday activity by		
			bike/foot, (min/workday)	Summer	
			G1 = <15	• G1 = 1.00 (referent)	
			G2 = 15-30	• G2 = 0.85 (0.47-1.53)	
			G3 = 30-60	• G3 = 0.60 (0.38-0.95)	
			G4 = >60	• G4 = 0.39 (0.26-0.59)	
			Outcome Measure: non fatal CHD	Multivariate OR (95% CI), workday activity by bike/foot	
			Unconditional logistic regression, linear regression model	• G1 = 1.00 (referent)	
				• G2 = 0.53 (0.30-0.93)	
				• G3 = 0.36 (0.21-0.62)	
				• G4 = 0.58 (0.36-0.94)	

Seccareccia and Menotti 1992 [248]	To examine the relationship between OPA and the risk of CHD death.	• n = 1,621	25 year of follow-up	• 189 cases	Increase in OPA is inversely related to risk of CHD death.
		• Sex: Men			
		• Age: 40-59 yr	PA assessment: Questionnaire for OPA (kcal/d), 3 groups	Age Standardized CHD and deaths rates:	
		• Characteristics: Healthy		• G1 = 18.9 ± 3.1	
Italy				• G2 = 13.1 ± 1.7	
			G1 = Sedentary, < 2400	• G3 = 11.0 ± 0.9	
Prospective cohort			G2 = Moderate, 2400-3199		
D & B score = 11			G3 = Heavy ≥ 3200		
			Indicators of PF including HR, vital capacity, FEV in 3/4 of sec, and corrected arm circumference (minus contribution of fat).		
			End Point: Fatal CHD		

Sesso et al 2000 [249]	To examine the association of the quantity and intensity of PA with CHD risk and the impact of other coronary risk factors.	• n = 12,516	PA assessment: Questionnaire	Number of Cases: 2,135	L-Shaped association between PA and the risk of CHD, with a reduction in CHD risk of approximately 20% for total PA levels >4200 kJ/wk
		• Sex: Men			
		• Age: 39-88 yr		Multivariate HR (95% CI)	
USA		• Characteristics: Healthy	PA Index (kJ/wk)	• G1 = 1.00 (referent)	
			G1 = <2100	• G2 = 0.90 (0.79-1.03)	
Prospective cohort		• Harvard Alumni	G2 = 2100-4199	• G3 = 0.81 (0.71-0.92)	
		Study	G3 = 4200-8399	• G4 = 0.80 (0.69-0.93)	
			G4 = 8400-12599	• G5 = 0.81 (0.71-0.94)	
D & B score = 12			G5 = >12600	*p *= 0.003	Suggests that vigorous activities are associated with a reduced risk of CHD, whereas moderate or light PA has no clear association with risk of CHD.
			Cox proportional HR		

Sundquist et al 2005 [250]	To examine the long term effect of LTPA on incident cases of CHD.	• n = 5,196 (2,645 men, 2,551 women)	PA assessment: Questionnaire Levels of PA	Age and sex adjusted RR (95% CI)	Positive long term effect of LTPA on CHD risk among men and women.
				• Q1 = 1.00 (referent)	
Sweden		• Sex: Men and women	Q1 = None	• Q2 = 0.72 (0.51-1.00)	
			Q2 = Occasionally	• Q3 = 0.64 (0.46-0.89)	
Prospective cohort		Age: 35-74 yr	Q3 = 1-2 times per week	• Q4 = 0.46 (0.29-0.74)	
		• Characteristics: Those not hospitalized for CHD in the last 2 years and those who rate their general health as poor were excluded	Q4 = Vigorous ≥2 times per week	Multivariate adjusted RR (95% CI)	
D & B score = 11			Outcome Measure: Fatal or non fatal CHD	• Q1 = 1.00 (referent)	
				• Q2 = 0.76 (0.55-1.07)	
				• Q3 = 0.74 (0.53-1.04)	
				• Q4 = 0.59 (0.37-0.95)	
			Cox regression model		

Talbot et al 2002 [251]	To examine the contributions of LTPA and aerobic fitness to the risk of coronary events in healthy younger and older adults.	• n = 689	Surveys began in 1960 and were completed on every visit	• Number of Cases: 63	In younger men PF predicts a reduced risk of CHD but not LTPA.
		• Sex: Men			
		• Age:		After adjusting for coronary risk factors there was:	
USA		51.6 ± 16.8 yr			
		• Characteristics: Community dwelling	PA assessment: Survey for LTPA (97 activities) at every visit.	RR: 0.53 (p < 0.001) and	In older men, high intensity LTPA and PF appear to be of similar importance in reducing CHD risk.
Prospective cohort				RR: 0.61 (*p *= 0.024) in older men.	
D & B score = 12		• Baltimore Longitudinal Study of Aging	PF assessment: Treadmill VO_2 _max test on alternate visits	Total LTPA was unrelated to coronary risk in either age group.	
				With 3 levels of LTPA intensity substituted for total LTPA:	
			Unpaired t-tests and chi square tests. Cox Proportional hazards Analysis	RR = 0.39 for tertile 3 vs. tertile 1	

Tanasescu et al 2002 [252]	To assess the amount, type and intensity of PA in relation to risk of CHD in men.	• n = 44,452	PA assessment: Questionnaire	• Number of Cases: 1,700	Total PA, running, weight training, and walking were associated with a reduced risk for CVD.
		• Sex: Men			
		• Age: 40-75 yr		Age adjusted HR (95% CI) by total PA	
USA		• Characteristics: Health professionals, no history of CHD and in good health	Total PA (MET hr/wk)	• Q1 = 1.00 (referent)	
			Q1 = 0-6.32	• Q2 = 0.85 (0.74 0.98)	
Prospective cohort			Q2 = 6.33-14.49	• Q3 = 0.78 (0.67-0.92)	
			Q3 = 14.50-25.08	• Q4 = 0.72 (0.62-0.83)	The average exercise intensity was associated with a reduced risk (independent of total PA).
			Q4 = 25.09-41.98	• Q5 = 0.58 (0.49-0.68)	
D & B score = 11			Q5 = > 41.99	*p *= .001	
		• Health Professionals follow-up study	Exercise intensity (METs)	Age adjusted HR (95% CI) by exercise intensity	
			G1 = Low-1-4	• G1 = .00 (referent)	
			G2 = Mod.-4-6	• G2 = 0.94 (0.83-1.04)	
			G3 = High 6-12		
			Walking pace independent of total volume of PA (mph)	• G3 = 0.83 (0.72-0.97)	
				*p *= 0.02	
			Q1 = <2	Age adjusted HR (95% CI) by walking pace	
			Q2 = 2-3	• Q1 = 1.00 (referent)	
			Q3 = 3-4	• Q2 = 0.72 (0.54-0.94)	
			Q4 = > 4	• Q3 = 0.61 (0.45-0.81)	
				• Q4 = 0.51 (0.31-0.84)	
			Outcome Measure: Nonfatal MI or Fatal CHD occurring during follow-up	*p *<0.001	
			Cox proportional HR		

Vatten et al 2006 [253]	To investigate whether obesity- related CV mortality could be modified by PA.	• n = 54,284 (27,769 men; 26,515 women)	Length of Follow-up: 16 years	• Number of Cases: 2,462	Increased PA reduces the risk of death in women, but not in men.
				Multivariate HR (95% CI), men	
Norway		• Sex: Men and women	PA assessment:	• Q1 = 1.00 (referent)	
			Questionnaire	• Q2 = 1.01 (0.89-1.16)	
Prospective cohort		Age: ≥ 20 yr	Divided into 4 groups	• Q3 = 0.98 (0.84-1.14)	
		• Characteristics: Free from CVD at baseline	Q1 = High	• Q4 = 1.18 (1.00-1.38)	
			Q2 = Medium	*p *= 0.11	
D & B score = 12			Q3 = Low		
		• HUNT study	Q4 = Never	Multivariate HR (95% CI), women	
			Outcome Measure: Ischemic heart disease mortality	• Q1 = 1.00 (referent)	
				• Q2 = 1.23 (1.01-1.51)	
				• Q3 = 1.54 (1.24-1.91)	
				• Q4 = 1.52 (1.23-1.88)	
			Cox proportional HR	*p *<0.001	

Wagner et al 2002 [254]	To investigate if the association between PA patterns and incidence of coronary events could explain the gradient in CHD observed between 2 countries.	• n = 9,758	Length of Follow-up: 5 yrs	Number of Cases: 167 hard CHD, 154 angina events	Beneficial effect of LTPA EE on hard CHD incidence in middle aged men.
		• Sex: Men and women	PA assessment: Questionnaire for LTPA, 3 groups:	Number of Dropouts: < 2%	
Ireland/France		• Age: 50-59 yr			
		• Characteristics: Healthy at Baseline		HR (95% CI), hard events	
Prospective cohort			G1 = Lowest	• G1 = 1.00 (referent)	
			G2 = Middle	• G2 = 0.73 (0.51-1.05)	
			G3 = Highest	• G3 = 0.66 (0.46-0.96)	
D & B score = 12			Outcome Measure: CHD hard events and Angina	*p *= 0.04	
				HR (95% CI), angina	
				• G1 = 1.00 (referent)	
			Cox proportional HR	• G2 = 0.83 (0.55-1.25)	
				• G3 = 1.28 (0.88-1.86)	
				*p *= 0.10	

D & B score, Downs and Black quality score; YR, years; G, groups; CHD, coronary heart disease; RR, risk ratio; 95% CI, 95% confidence interval; PA, physical activity; VPA, vigorous physical activity; CV, cardio vascular; MET, metabolic equivalent; kcal/wk, kilocalories per week; Q, quartile or quintile; km/h, kilometers per hour; LTPA, leisure-time physical activity; HR, hazard ratio; OPA, occupational physical activity; kcal/kg/day kilocalories per kilogram per day; MI, myocardial infarction; ECG, electrocardiogram; kcal/kg/h kilocalories per kilogram per hour; mph, miles per hour; CVD, cardiovascular disease.

Similar to the all-cause mortality data, the risk for cardiovascular disease demonstrates a graded inverse dose-response relationship to physical activity and fitness. The relative reduction in the incidence of cardiovascular disease averages 33% (median risk reduction of 36%), with greater risk reductions in studies that employed objective measures of aerobic fitness. It is not uncommon for studies to demonstrate a 50% or higher risk reduction when an objective measure of physical fitness was taken (Table 12). The importance of physical activity may actually be underestimated owing to multivariate control for many confounding factors (as discussed previously) and the fact that effects of within-person variation in physical activity are often not considered [55]. The relative risk reduction appears to be similar for men and women, and also appear to extend to non-Caucasian populations [56]. Some evidence also exists indicating that small amounts of physical activity are associated with lower cardiovascular-disease related mortality [57,58]. Similar to all-cause mortality, physical activity confers health benefits independent of other known risk factors [42,59]. *Collectively, the level of evidence would be considered to be Level 2A based on the presence of overwhelming evidence from observational trials*. The quality of the investigations was generally high with a mean (and median) Downs and Black score of 12 (range 9-14).

#### Implications

Research in the field began with the landmark work of Morris and colleagues, which demonstrated that men in physically demanding occupations (bus conductors and postmen) had a significantly lower risk of heart disease than individuals who worked in less demanding jobs (bus drivers and office workers) [45]. Since then considerable research has examined the relationship between physical activity and the risk for cardiovascular disease. In fact, several systematic reviews of the literature have been developed regarding the role of habitual physical activity in the primary and secondary prevention of cardiovascular disease [33,60-62]. The research to date has been consistent and compelling, habitual physical activity reduces markedly the risk for cardiovascular disease.

Based on the available literature, there is compelling evidence that the recommendation of 30 min of moderate intensity exercise on most days of the week (equivalent to 4.2 MJ/wk or 1000 kcal/wk) reaches a threshold associated with significant reductions in cardiovascular-related mortality [32,63]. Brisk walking has also been shown to be preferable to a slower pace [64]. However, weekly exercise volumes of less than 4.2 MJ (1000 kcal) may be cardio-protective [14,59,65-67]. For instance, Lee et al. (2001) found that as little as 1 hr/wk of walking was associated with a 50% lower cardiovascular disease mortality in one sample of women. Wisloff et al. [58] reported that a single weekly bout of self-reported high intensity exercise was associated with a lower risk of cardiovascular death relative to those reporting no activity in both men (RR = 0.61, 95% CI = 0.49-0.75), and women (RR = 0.49, 95% CI = 0.27-0.89). Moreover, no additional benefit was seen with higher durations or frequency of exercise sessions [58]. The authors stated that this evidence challenges "current recommendations that require at least 1000 kcal of caloric expenditure per week to achieve exercise-induced protection against premature cardiovascular death." However, this research is in fact supportive of the Canadian guidelines which recognize the potential health benefits of low volumes of physical activity as reflected by the statement "Every little bit counts, but more is even better - everyone can do it!" It however should be noted that the statement "more is even better" is supported by a strong evidence base.

*Recommendation #2*

*For a reduced risk for cardiovascular disease-related events and mortality, it is recommended that individuals participate in 30 min or more of moderate to vigorous exercise on most days of the week. Greater health benefits appear to occur with high volume and/or intensities of activity. Health benefits may also occur with as little as one hr of brisk walking per week. [Level 2, Grade A]*

### The Primary Prevention of Stroke

Stroke affects a significant proportion of Canadian society with approximately 50,000 new cases each year [68]. The relationship between physical activity and the risk for stroke is compelling, supporting at least a 25-30% risk reduction in the most active individuals [31]. In fact, in a review of the literature Katzmarzyk and Janssen [20] reported that lack of physical activity carried a relative risk of 1.60 (95% CI = 1.42-1.80) for stroke, similar to or higher than that for coronary heart disease (1.45), hypertension (1.30), colon cancer (1.41), breast cancer (1.31), type 2 diabetes (1.50), and osteoporosis (1.59).

**Figure 5:**
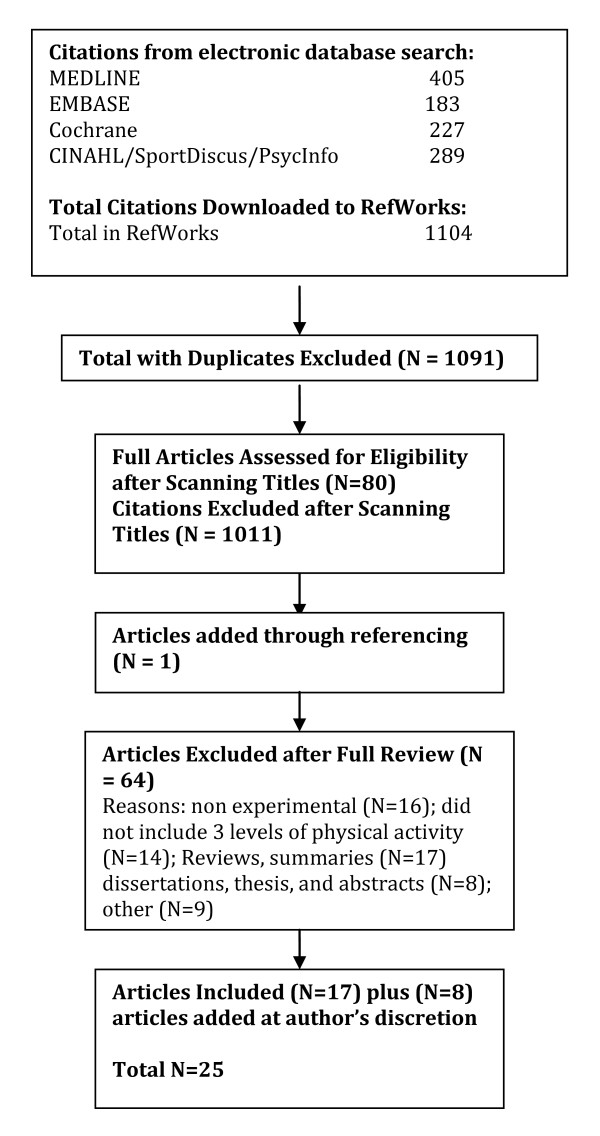
**Results of the Literature Search for Stroke**.

In our systematic review of the literature, a total of 1104 citations were identified during the electronic database search (Figure 5). Of these citations, 405 were identified in MEDLINE, 183 in EMBASE, 227 in Cochrane, and 289 in the CINAHL/SportDiscus/PsychInfo search. A total of 13 duplicates were found, leaving a total of 1091 unique citations. A total of 1011 articles were excluded after scanning, leaving a total of 80 articles for full review. An additional 9 articles were retrieved through cross-referencing and the authors' knowledge of the field. From these articles 64 were excluded after full review leaving 25 articles for inclusion in the systematic review. The reasons for exclusion included non-experimental/weak design (poor execution introducing bias) (n = 16), did not contain three levels of physical activity or not possible to determine dose-response relationship (n = 14), reviews, summaries, meta-analyses (n = 17), dissertations, thesis, abstracts (n = 8), and other (n = 9). Therefore, a total of 25 articles were included in the systematic review of the literature regarding the relationship between physical activity and the primary prevention of stroke (Table 13).

**Table 13:** Studies examining the relationship between physical activity and stroke.

Publication Country Study Design Quality Score	Objective	Population	Methods	Outcome	Comments and Conclusions
Wisloff et al 2006 [58]	To assess exercise amount and intensity in relation to subsequent CVD mortality (including stroke).	• n = 27,143 men, 28,929 women	16 year follow up	Multivariate adjusted RR (95% CI) Men	Both high and low- intensity exercise may be associated with a reduced risk of stroke in both men and women.
Norway		• Sex: Men and women	PA Assessment: Questionnaire	G1 = 1.00 (referent)	
		• Age: ≥ 20 yr		G2 = 0.90 (0.70-1.17)	
		• Characteristics: free from CVD	PA	G3a = 0.90 (0.64-1.26)	
		• HUNT Study	G1 = None	G3b = 0.59 (0.27-1.27)	
			G2 = <1/wk	G3c = 0.62 (0.40-0.95)	
			G3a = 1/wk ≤ 30 min low	G3d = 0.51 (0.31-0.86)	
			G3b = 1/wk ≤ 30 min high	G4a = 0.72 (0.49-1.05)	
			G3c = 1/wk > 30 min low	G4b = 0.63 (0.31-1.30)	
Prospective cohort			G3d = 1/wk > 30 min high	G4c = 1.02 (0.72-1.44)	
			G4a = 2-3/wk ≤ 30 min low	G4d = 0.59 (0.37-0.92)	
			G4b = 2-3/wk ≤ 30 min high	G5a = 0.97 (0.70-1.36)	
D & B score = 12			G4c = 2-3/wk > 30 min low	G5b = 0.68 (0.27-1.66)	
			G4d = 2-3/wk > 30 min high	G5c = 0.81 (0.65-1.20)	
			G5a = ≥ 4/wk ≤ 30 min low	G5d = 0.67 (0.49-1.11)	
			G5b = ≥ 4/wk ≤ 30 min high		
			G5c = ≥ 4wk > 30 min low	RR (95% CI) Women	
			G5d = ≥ 4/wk > 30 min high	G1 = 1.00 (referent)	
			Outcome Measure: IHD mortality	G2 = 1.01 (0.81-1.25)	
			Cox proportional HR	G3a = 0.88 (0.68-1.15)	
				G3b = 0.98 (0.46-2.10)	
				G3c = 0.63 (0.42-0.94)	
				G3d = 1.00 (0.50-1.98)	
				G4a = 0.91 (0.70-1.17)	
				G4b = 1.44 (0.78-2.65)	
				G4c = 0.62 (0.44-0.88)	
				G4d = 0.77 (0.36-1.66)	
				G5a = 0.74 (0.56-0.99)	
				G5b = 0.40 (0.10-1.62)	
				G5c = 0.63 (0.45-0.89)	
				G5d = 0.51 (0.21-1.26)	

Abbott et al 2003 [69]	To examine the way in which risk factor effects on the incidence of thromboembolic and hemorrhagic stroke can change over a broad range of ages.	• n = 7,589	6, 15 and 26 year follow up	Incidence rates per 1000 of stroke:	The protective effect of PA on reducing risk of stroke increased with age.
USA		• Sex: Men		• G1 = 9.0 (49)	
		• Age: 45-93 yr	PA assessment: Using PA index over a 24 hour period PA information collected at study enrolment 1965-1968 and updated at physical examinations that occurred at 6, 15 and 26 years into follow-up.	• G2 = 17.8 (124)	
Prospective cohort		• Characteristics: Free from CHD and stroke at enrolment; Japanese ancestry living on the island of Oahu, Hawaii.	Grouped into 4 age groups, yr:	• G3 = 33.4 (112)	
D & B score = 14		• Honolulu Heart Program	G1 = 45-54	• G4 = 48.1 (111)	
			G2 = 55-64	Incidence of stroke event increased with advancing age p <0.001	
			G3 = 65-74	There appeared to be a small protective effect within each age group. Inverse relations increased with age (p = 0.046). The protective effect of PA became significant in men >77 years (p = 0.032)	
			G4 = 75-93		
			Outcome Measure: diagnosis of fatal and non fatal stroke during 26 years of follow-up		
			Cox proportional HR		

Gillium et al 1996 [70]	To examine the relationship between recreational and non-recreational PA and risk of stroke.	• n = 2,368 men, 2,713 women	11.6 year follow up	Number of Cases: 249 white women, 270 white men, 104 black	Sedentary behaviour was found to be associated with increased risk of stroke.
USA		• Sex: Men and women	PA assessment: Questionnaire divided into tertiles:		
		• Age: 45-74 yr	T1 = Low	RR (95% CI) Black men and women Recreational PA	
Prospective cohort		• Ethnicity: Black and white	T2 = Medium	• T1 = 1.33 (0.67-2.63)	
D & B score = 12		• NHANES I	T3 = High	• T2 = 1.33 (0.63-2.79)	
				• T3 = 1.00 (referent)	
			Outcome Measure: Total Stroke	Non-recreational PA	
			Cox proportional HR	• T1 = 1.40 (0.90-2.16)	
				• T2 = 1.41 (0.74-2.70)	
				• T3 = 1.00 (referent)	
				RR (95% CI) White men age 45-64 Recreational PA	
				• T1 = 1.24 (0.63-2.41)	
				• T2 = 1.17 (0.61-2.27	
				• T3 = 1.00 (referent)	
				Non-recreational PA	
				• T1 = 1.07 (0.40-2.86)	
				• T2 = 1.75 (1.04-2.96)	
				• T3 = 1.00 (referent)	
				RR (95% CI) White women age 45-64 Recreational PA	
				• T1 = 3.13 (0.95-10.32)	
				• T2 = 1.80 (0.52-6.22)	
				• T3 = 1.00 (referent)	
				Non-recreational PA	
				• T1 = 3.51 (1.66-7.46)	
				• T2 = 1.07 (0.57-1.99)	
				• T3 = 1.00 (referent)	
				RR (95% CI) White men age 65-74 Recreational PA	
				• T1 = 1.29 (0.58-1.88)	
				• T2 = 0.86 (0.58-1.28)	
				• T3 = 1.00 (referent)	
				Non-recreational	
				• T1 = 1.82 (1.15-2.88)	
				• T2 = 1.20 (0.88-1.64)	
				• T3 = 1.00 (referent)	
				RR (95% CI) White women age 65-75 Recreational PA	
				• T1 = 1.55 (0.95-2.53)	
				• T2 = 1.27 (0.76-2.12)	
				• T3 = 1.00 (referent)	
				Non-recreational PA	
				• T1 = 1.82 (1.10-3.02)	
				• T2 = 1.42 (1.01-2.00)	
				• T3 = 1.00 (referent)	

Lee and Blair 2002 [71]	To examine the association between PF and stroke mortality in men.	• n = 16,878	Baseline medical evaluation between 1971 and 1994 with average follow up period of 10 years	Average estimated maximal METs	Moderate and high levels of PF were associated with lower risk of stroke mortality in men.
		• Sex: Men		• T1 = 8.5 MET	
		• Age: 40-87 yrs		• T2 = 10.5 MET	
USA		• Aerobics Center Longitudinal Study		• T3 = 13.1 MET	
Prospective cohort			PF assessment: Maximal exercise tolerance test, divided into tertiles	RR (95% CI) adjusted for age and exam year	
				• T1 = 1.00 (referent)	
D & B score = 13			T1 = Low	• T2 = 0.35 (0.16-0.77)	
			T2 = Moderate	• T3 = 0.28 (0.11-0.71)	
			T3 = High	Trend p = 0.005	
			Cox proportional HR		

Hu et al 2000 [72]	To examine the association between PA and risk of total stroke and stroke sub- types in women.	• n = 72,488	Baseline measurement in 1986 with follow-up questionnaire in 1988 and 1992	• 407 cases of stroke (258 ischemic strokes, 67 subarachnoid hemorrhages, 42 intracerebral hemorrhages, and 40 strokes of unknown type)	PA, including moderate-intensity exercise such as walking, is associated with a substantial reduction in risk of total and ischemic stroke in a dose- response manner.
		• Sex: Women			
		• Age:40-65 yr			
USA		• Characteristics: Nurses			
Prospective cohort		• Nurses' Health Study	PA assessment: Questionnaire for total PA (MET h/wk), divided into quintiles, walking activity (MET h/wk), divided into quintiles and walking pace	Multivariate RR (95% CI) for total stroke by total PA level	
				• Q1 = 1.00 (referent)	
D & B score = 13				• Q2 = 0.98	
				• Q3 = 0.82	
				• Q4 = 0.74	
				• Q5 = 0.66	
			Total PA (MET h/wk)		
				p = 0.005	
			Q1 = 0 - 2.0		
			Q2 = 2.1 - 4.6		
				Multivariate RR (95% CI) for ischemic Stroke by total PA level	
			Q3 = 4.7 - 10.4		
			Q4 = 10.5-21.7		
				• Q1 = 1.00 (referent)	
			Q5 = > 21.7		
				• Q2 = 0.87	
			Walking activity (MET h/wk)	• Q3 = 0.83	
			Q1 = 0.5	• Q4 = 0.76	
			Q2 = 0.6 - 2.0	• Q5 = 0.52	
			Q3 = 2.1 - 3.8	p = 0.003	
			Q4 = 3.9 - 10		
			Q5 = 10	Multivariate RR (95% CI) for total stroke by walking activity	
			Walking pace (mph)	• Q1 = 1.00 (referent)	
			G1 < 2.0	• Q2 = 0.76	
			G2 = 2-2.9	• Q3 = 0.78	
			G3 3.0	• Q4 = 0.70	
				• Q5 = 0.66	
			Outcome measure: Stroke incidence	p = 0.01	
				Multivariate RR (95% CI) for ischemic stroke by walking activity	
			Pooled logistic regression		
			Cox proportional HR	• Q1 = 1.00 (referent)	
				• Q2 = 0.77	
				• Q3 = 0.75	
				• Q4 = 0.69	
				• Q5 = 0.60	
				p = 0.02	
				Multivariate RR (95% CI) for total stroke by usual Walking Pace	
				• G1 = 1.00 (referent)	
				• G2 = 0.81	
				• G3 = 0.49	
				p < 0.001	
				Multivariate RR (95% CI) for ischemic stroke by usual walking pace	
				• G1 = 1.00 (referent)	
				• G2 = 0.71	
				• G3 = 0.47	
				p < 0.001	

Lee et al 1999 [74]	To examine the association between exercise and stroke risk.	• n = 21,823	11.1 year follow up	Number of Cases: 533	VPA is associated with a decreased risk of stroke in men.
		• Sex: Men			
		• Age: 40-84 yr	PA assessment: Questionnaire for frequency of VPA, divided into 4 groups	Multivariate RR1 (95% CI) for total stroke by VPA	
USA					
				• G1 = 1.00 (referent)	
Prospective cohort				• G2 = 0.79 (0.61-1.03)	Inverse association with PA seemed to be mediated through beneficial effects on body weight, BP, cholesterol and glucose tolerance.
			G1 < 1 time/week	• G3 = 0.80 (0.65-0.99)	
			G2 = 1 time/week	• G4 = 0.79 (0.61-1.03)	
D & B score = 13			G3 = 2-4 times/week	*p *= 0.04	
			G4 ≥ 5 times/week	RR2 (95% CI) for total stroke by VPA	
				• G1 = 1.00 (referent)	
			RR1 = adjusted for smoking, alcohol consumption, history of angina and parental history of MI at <60 years	• G2 = 0.81 (0.61-1.07)	
				• G3 = 0.88 (0.70-1.10)	
				• G4 = 0.86 (0.65-1.13)	
				*p *= 0.25	
				RR2 (95% CI) for ischemic stroke by	
			RR2 = adjusted for all of the above plus, BMI, history of, hypertension, high cholesterol and diabetes		
				VPA	
				• G1 = 1.00 (referent)	
				• G2 = 0.90 (0.66-1.22)	
				• G3 = 0.95 (0.74-1.22)	
				• G4 = 0.97 (0.71-1.32)	
			Outcome Measure: Total Stroke (Ischemic and Hemorrhagic)	*p *= 0.81	
				RR2 (95% CI) for hemorrhagic stroke by VPA	
			Cox proportional HR	• G1 = 1.00 (referent)	
				• G2 = 0.54 (0.25-1.13)	
				• G3 = 0.71 (0.41-1.23)	
				• G4 = 0.54 (0.26-1.15)	
				*p *= 0.10	

Bijnen et al 1998 [166]	To describe the association between the PA patterns of elderly men and stroke mortality.	• n = 802	10 year follow up	Number of Cases: 47	No significant finding
		• Sex: Men			
		• Age:64-84 yr	PA assessment:	Multivariate adjusted RR (95% CI)	
Denmark		• Characteristics: Not all free from previous stroke	Questionnaire for LTPA, divided into tertiles	• T1= 1. 00 (referent)	
				• T2 = 0.65 (0.33-1.25)	
Prospective cohort			T1 = Lowest	• T3 = 0.55 (0.24-1.26)	
			T2	*p *= 0.12	
			T3 = Highest		
D & B score = 15					
			Outcome Measure: Stroke Mortality		
			Cox proportional HR		

Schnohr et al 2006 [214]	To describe the association between different levels of LTPA and subsequent causes of death (stroke).	• n = 2136 men, 2,758 women	5 year follow up	RR (95% CI), univariate	Although RR for of death from stroke was below 1 for both moderate and high compared with low PA, this association did not reach the level of statistical significance.
				• G1 = 1.00 (referent)	
		• Sex: Men and women	PA assessment:	• G2 = 0.64 (0.39-1.05)	
Copenhagen		• Age: 20 -- 79 yr	Questionnaire for LTPA,	• G3 = 0.70 (0.41-1.21)	
		• Characteristics: Healthy, PA level did not change between 2 examinations, 5 years apart	divided into 3 groups	Trend *p *= 0.4	
Prospective cohort			G1 = Low PA (<4 METS)		
			G2 = Moderate PA (4-6	RR (95% CI), multivariate:	
			METS)	• G1 = 1.00 (referent)	
D & B score = 13			G3 = High PA (>6 METS)	• G2 = 0.67 (0.40-1.12)	
		• Copenhagen City Heart Study		• G3 = 0.76 (0.43-1.34)	
			Multivariate Analysis Kaplan-Meier Plots	Trend *p *= 0.6	
			Linear, Logistical and Cox Regression.		

Vatten et al 2006 [253]	To investigate whether obesity- related CV mortality could be modified by PA.	• n = 26,515 men, 27,769 women	16 year follow up	Number of Cases: 994 women, 771 men	Lower levels of TPA are associated with an increased risk of stroke.
		• Sex: Men and women	PA assessment: Questionnaire for total amount of PA, divided into 4 groups		
Norway		• Age: 20 yr		Multivariate HR (95% CI), men	
		• Characteristics: Free from CVD at baseline		• Q1 = 1.00 (referent)	
Prospective cohort				• Q2 = 1.05 (0.85-1.30)	
		• HUNT study	G1 = High	• Q3 = 1.21 (0.95-1.54)	
			G2 = medium	• Q4 = 1.35 (1.05-1.74)	
D & B score = 14			G3 = low	*p *= 0.009	
			G4 = never		
				Multivariate HR (95% CI), women	
			Outcome Measure: Stroke mortality	• Q1 = 1.00 (referent)	
				• Q2 = 1.16 (0.93-1.45)	
				• Q3 = 1.45 (1.14-1.86)	
			Cox proportional HR		
				• Q4 = 1.45 (1.14-1.83)	
				*p *< 0.001	

Agnarsson et al 1999 [255]	To examine the association of LTPA and pulmonary function with the risk of stroke.	• n = 4,484	Length of Follow-up: 10.6 ± 3.6 years	Number of Cases: 249	Apparent protective effect of regular continued LTPA in middle age men on the risk of ischemic stroke.
		• Sex: Men			
		• Age: 45-80		Adjusted for age and smoking RR (95% CI) for total stroke by LTPA level	
Iceland		• Characteristics: no history of Stroke	PA assessment: Questionnaire for LTPA (h/wk) and type of activity (intensity), each divided into 3 groups		
Prospective cohort		• Reykjavik Study		• G1 = 1.00 (referent)	
				• G2 = 0.84 (0.63-1.13)	
				• G3 = 0.73 (0.40-1.35)	
D & B score = 13			LTPA summer/winter		
			G1 = none	Adjusted for age and smoking RR (95% CI) for ischemic stroke by LTPA level	
			G2 = ≤ 5 h/wk		
			G3 = ≥ 6 h/wk		
				• G1 = 1.00 (referent)	
			Type of Activity	• G2 = 0.72 (0.51-1.01)	
			G1 = none		
				• G3 = 0.78 (0.41-1.48)	
			G2 = low intensity		
			G3 = high Intensity		
				RR (95% CI) for total stroke by type of activity	
			Outcome Measure: Total and ischemic Stroke	• G1 = 1.0,0 (referent)	
				• G2 = 0.75 (0.53-1.08)	
				• G3 = 1.10 (0.78-1.57)	
			Cox proportional HR		
				RR (95% CI) for ischemic stroke by type of activity	
				• G1 = 1.00 (referent)	
				• G2 = 0.72 (0.44-1.07)	
				• G3 = 0.96 (0.64-1.44)	

Ellekjaer et al 2000 [256]	To examine the association between different levels of LTPA and stroke mortality in middle-aged and elderly women.	• n = 14,101	Baseline 1984-1986: 2 self administered questionnaires and clinical measurements included in the screening program.	Number of cases: 457	This study demonstrates a consistent, negative association between PA and stroke mortality in women.
		• Sex: Women			
		• Age: 50 yr		Multivariate RR (95% CI), all age groups	
Norway		• Characteristics: free from stroke at baseline			
				• G1 = 1.00 (referent)	
Prospective cohort				• G2 = 0.77	
			PA assessment: Questionnaire for LTPA, divided into 3 groups	• G3 = 0.52	
D & B score = 14				Multivariate RR (95% CI), age 50--69 years	
			G1 = low		The most active women had approx. 50% lower risk of death from stroke compare to inactive women.
			G2 = medium	• G1 = 1.00 (referent)	
			G3 = high	• G2 = 0.57	
				• G3 = 0.42	
			Outcome Measure: Death from stroke	*p *= 0.0021	
				Multivariate RR (95% CI), age 70-79 years	
			Cox proportional HR		
				• G1 = 1.00 (referent)	
				• G2 = 0.79	
				• G3 = 0.56	
				*p *= 0.0093	
				Multivariate RR (95% CI), age 80-101 years	
				• G1 = 1.00 (referent)	
				• G2 = 0.91	
				• G3 = 0.57	
				*p *= 0.1089	

Evenson et al 1999 [257]	To examine the relationship between PA and ischemic stroke risk.	• n = 14,575	7.2 year follow up	Number of Cases: 189	PA was weakly associated with a reduced risk of ischemic stroke among middle aged adults.
		• Sex: Men and women		Number of Dropouts: 0%	
		• Age: 45-64 yr	PA assessment: Questionnaire (Baecke questionnaire)		
USA		• Atherosclerosis Risk in Communities Study		Sport, Incidence of Ischemic Stroke	
Prospective cohort				Multivariate adjusted RR (95% CI) by sport	
			Outcome Measure:		
			Ischemic Stroke	• Q1 = 1.00 (referent)	
D & B score = 14				• Q3= 0.83 (0.52-1.32)	
			Multivariate Poisson and Cox proportional HR		
				Multivariate adjusted RR (95% CI) by LTPA	
				• Q1 = 1.00 (referent)	
				• Q2 =	
				• Q3 = 0.89 (0.57-1.37)	
				Multivariate adjusted RR (95% CI) by OPA	
				• Q1 = 1.00 (referent)	
				• Q2 =	
				• Q3 = 0.69 (0.47-1.00)	

Haheim et al 1993 [258]	To determine the risk factors of stroke incidence and mortality.	• n = 14,403	Baseline Screening from May 1972- December 1973.	HR (95% CI) for stroke incidence	Increased LTPA is associated with a reduced risk of stroke incidence but not mortality.
		• Sex: Men		• G1 = 1.00 (referent)	
		• Age: 40-49 yr		• G2 = 0.64 (0.38-1.08)	
Norway			PA assessment: Questionnaire for LTPA, divided into groups	• G3 = 0.36 (0.15-0.80)	
Prospective cohort				HR (95% CI) for stroke mortality	
			G1 = Sedentary	• G1 = 1.00, (referent)	
			G2 = Moderate	• G2 = 0.82 (0.33-2.35)	
D & B score = 14			G3 = Intermediate or Great	• G3 = 0.29 (0.03-1.51)	
			Outcome Measure: Incidence of stroke morbidity and mortality until study end date, December 31, 1984.		
			Cox proportional HR		

Hu et al 2005 [259]	To assess the relationship of different types of PA with total and type-specific stroke risk.	• n = 47,721	PA assessement: Mailed questionnaire for LTPA, OPA and commuting PA, divided into groups as follows:	RR (95% CI) by LTPA, men	A high level of LTPA reduces the risk of all subtypes of stroke. Daily active commuting also reduces the risk of ischemic stroke.
		• Sex: Men and women		• G1 = 1.00 (referent)	
				• G2 = 0.83	
Finland		• Age: 25-64		• G3 = 0.72	
		• Characteristics: Healthy at baseline		*p *< 0.001	
Prospective cohort					
			LTPA levels:	RR (95% CI) by LTPA, women	
			G1 = Low	• G1 = 1.00 (referent)	
D & B score = 13			G2 = Moderate	• G2 = 0.86	
			G3 = High	• G3 = 0.75	
				*p *= 0.007	
			OPA:		
			G1 = Light	RR (95% CI) by LTPA, men and women	
			G2 = Moderate		
			G3 = Hard		
				• G1 = 1.00 (referent)	
				• G2 = 0.85	
			Commuting PA:		
			G1 = Motorized or no work,	• G3 = 0.73	
			G2 = walking or cycling 1-29 min G3 = walking or cycling ≥ 30 min.	*p *<0.001	
				RR (95% CI) by OPA, men	
				• Not significant	
			Outcome Measure: Incidence of fatal or non-fatal stroke occurring during follow-up until end of 2003. Mean follow-up of 19 years.		
				RR (95% CI) by OPA, women	
				• Not significant	
				RR (95% CI) by OPA, men and women	
				• G1 = 1.00 (referent)	
			Cox proportional hazard	• G2 = 0.90	
				• G3 = 0.87	
				p = 0.007	
				RR (95% CI) by commuting PA, men	
				• G1 = 1.00 (referent)	
				• G2 = 0.91	
				• G3 = 0.85	
				*p *= 0.047	
				RR (95% CI) by commuting PA, women	
				• G1 = 1.00 (referent)	
				• G2 = 0.86	
				• G3 = 0.85	
				*p *= 0.018	
				RR (95% CI) by commuting PA, men and women	
				• G1 = 1.00 (referent)	
				• G2 = 0.89	
				• G3 = 0.85	
				*p *= 0.002	

Kiely et al 1994 [260]	To examine the influence of increased PA on stroke risk in members of the Framingham study cohort.	• n = 1,897 men 2,299 women	Baseline measurement in 1954-1955 and follow up in either 1968-1969 or 1971- 1972	Multivariate adjusted RR (95% CI) at first examination, men (mean age 50 years)	Medium and high levels of PA among men are protective against stroke relative to low levels.
		• Sex: Men and women			
USA				• G1 = 1.00 (referent)	
		• Age: 28-62 yr		• G2 = 0.90 (0.62-1.31) *p *= 0.59	
Prospective cohort		• Characteristics: Free from stroke	PA assessment: Questionnaire for metabolic work done during a typical 24 hr period, divided into 3 groups	• G3 = 0.84 (0.59-1.18) *p *= 0.31	
				Multivariate adjusted RR (95% CI) at first examination, women (mean age 50 years)	Protective effect of PA was slightly less for high levels of PA compared to medium levels for older men.
D & B score = 12					
			G1 = Low	• G1 = 1.00 (referent)	
			G2 = Medium	• G2 = 1.21 (0.89-1.63) *p *= 0.23	
			G3 = High	• G3 = 0.89 (0.60-1.31) *p *= 0.54	
			Outcome Measure: Incidence of stroke, as defined by the first occurrence of atherothrombotic brain infarctions, cerebral embolism or other type of stroke, during 32 years of follow-up.		
				Multivariate adjusted RR (95% CI) at second examination, men (mean age 63 years)	
				• G1 = 1.00 (referent)	
				• G2 = 0.41 (0.24-0.89) *p *= 0.0007	
				• G3 = 0.53 (0.34-0.84) *p *= 0.007	
				Multivariate adjusted RR (95% CI) at second examination, women (mean age 64 years)	
			Cox proportional HR		
				• G1 = 1.00 (referent)	
				• G2 = 0.97 (0.64-1.47) p = 0.67	
				• G3 = 1.21 (0.75-1.96) p = 0.43	

Krarup et al 2007 [261]	To compare the reported level of PA performed during the week preceding an ischemic stroke with that of community controls.	• n = 127 cases 301 controls	PA assessment:	Univariate OR (95% CI)	Stroke patients are less physically active in the week preceding an ischemic stroke when compared to age and sex-matched controls. Increasing PASE score was inversly, log-linearly and significantly associated with OR for ischemic stroke.
			Questionnaire about PA 1 week prior to stroke (cases) and 1 week prior to questionnaire (controls), divided into PASE scores and quartiles	PASE Score	
		• Sex: Men and women		• Q1 = 1.00 (referent)	
Denmark				• Q2 = 0.51 (0.28-0.95)	
		• Age: ≥ 40 yr		• Q3 = 0.27 (0.14-0.54)	
Case control		• Characteristics: Case: Stroke Patients (20% had history of Stroke), Controls: 4% had history of stroke		• Q4 = 0.08 (0.03-0.20)	
D & B score = 14			Q1 = 0-49	Multivariate OR (95% CI) PASE Score	
			Q2 = 50-99		
			Q3 = 100-149	• Q1 = 1.00 (referent)	
			Q4 = 150+	• Q2 = 0.53 (0.26-1.08)	
				• Q3 = 0.27 (0.12-0.59)	
			Outcome measure:		
			Ischemic stroke	• Q4 = 0.09 (0.03-0.25)	
			Chi squared Kruskal-Wallis Statistics Multivariate conditional logistic regression		

Kurl et al 2003 [262]	To examine the relationship of PF with subsequent incidence of stroke. Also to compare PF with conventional risk factors as a predictor for future stroke.	• n = 2,011	Baseline examinations conducted between March 1984 and December 1989 with average follow up period of 11 years	Multivariate HR (95% CI), any stroke	Low PF was associated with an increased risk of any stroke and ischemic stroke.
		• Sex: Men		• Q1 = 1.00 (referent)	
		• Age: 42, 48, 54 or 60 yrs		• Q2 = 1.39 (0.70-2.77)	
Finland				• Q3 = 1.32 (0.66-2.65)	
		• Characteristics: Free from stroke or pulmonary disease • Kuopio Ischaemic Heart Disease Risk Factor Study		• Q4 = 2.30 (1.18-4.06)	
Prospective cohort				Trend *p *= 0.01	
			PF assessment: Maximal exercise test on cycle ergometer. VO_2 _max (ml/kg/min) divided into quartiles		
				Multivariate HR (95% CI), ischemic stroke	
D & B score = 14					
				• Q1 = 1.00 (referent)	
				• Q2 = 1.28 (0.56-2.94)	
				• Q3 = 1.64 (0.74-3.65)	
			Q1 = >35.3		
				• Q4 = 2.40 (1.09-5.25)	
			Q2 = 30.3-35.3		
				Trend *p *= 0.01	
			Q3 = 25.2-30.2		
			Q4 = <25.2		
			Outcome Measure: Stroke incidence		
			Cox proportional HR		

Myint et al 2006 [263]	To examine the association between a combination of OPA and LTPA with risk of subsequent stroke.	• n = 22,602	Baseline measurement in	Model A: Used all 4 categories of PA	Higher levels of PA assessed using a single simple pragmatic tool based on both OPA and LTPA is associated with reduced stroke risk.
		• Sex: Men	1993-1997	HR (95% CI), men and women	
		• Age: 40-79 yr		• G1 = 1.00 (referent)	
UK		• Characteristics: Healthy at baseline	PA assessment: Questionnaire for PA (includes LTPA and OPA) divided into 4 groups	• G2 = 0.78 (0.61-1.00)	
				• G3 = 0.66 (0.49-0.91)	
Prospective cohort		• European Prospective Investigation in Cancer-Norfolk		• G4 = 0.70 (0.49-0.99)	
				*p *= 0.024	
D & B score = 11			G1 = Inactive	HR (95% CI), men	
			G2 = moderately inactive	• G1 = 1.00 (referent)	
			G3 = moderately active	• G2 = 0.75 (0.52-1.09)	
			G4 = active		
				• G3 = 0.55 (0.35-0.86)	
				• G4 = 0.67 (0.43-1.05)	
			Outcome Measure: Incidence of fatal and non fatal stroke.		
				*p *= 0.41	
				Women not significant *p *= 0.50	
			Cox proportional HR		
				Model B: Used 3 categories of PA (G3 and G4 combined combined)	
				HR (95% CI), men and women	
				• G1 = 1.00 (referent)	
				• G2 = 0.78 (0.61-1.00)	
				• G3 = 0.68 (0.52-0.88)	
				*p *= 0.009	
				HR (95% CI), men	
				• G1 = 1.00 (referent)	
				• G2 = 0.75 (0.52-1.09),	
				• G3 = 0.61 (0.43-0.86)	
				*p *= 0.019	
				Women not significant p = 0.34	

Noda et al 2005 [264]	To examine the impact of exercise on CVD (stroke) mortality in Asian populations.	• n = 31,023 men, 42,242 women	9.7 year follow up	Number of Cases: 186 men, 141 women	PA through walking and sports participation may reduce the risk of mortality from ischemic stroke
		• Sex: Men and women	PA assessment: Questionnaire for PA (walking and sports participation (h/day), divided into quartiles:	Number of Dropouts: 3.4%	
Japan		• Age: 40 -79 yr			
		• Ethnicity: Asian		Multivariate adjusted HR (95% CI) by duration of walking PA, men	
Prospective cohort					
				• Q1 = 1.03 (0.63-1.69)	
			Q1 = <0.5	• Q2 = 1.00 (referent)	
D & B score = 13			Q2 = 0.5	• Q3 = 0.56 (0.35-0.91)	
			Q3 = 0.6-0.9	• Q4 = 0.71 (0.49-1.02)	
			Q4 = >1.0		
				Multivariate adjusted HR (95% CI) by duration of walking PA, women	
			Outcome Measure: Death from ischemic stroke		
				• Q1 = 1.38 (0.82-2.33)	
				• Q2 = 1.00 (referent)	
			Cox proportional HR		
				• Q3 = 0.56 (0.32-0.97)	
				• Q4 = 0.73 (0.48-1.13)	
				Multivariate adjusted HR (95% CI) by sport PA, men	
				• Q1 = 1.34 (0.86-2.08)	
				• Q2 = 1.00 (referent)	
				• Q3 = 1.22 (0.66-2.25)	
				• Q4 = 0.84 (0.45-1.57)	
				Multivariate adjusted HR (95% CI) by sport PA, women	
				• Q1 = 1.07 (0.64-1.77)	
				• Q2 = 1.00 (referent)	
				• Q3 = 0.62 (0.25-1.58)	
				• Q4 = 0.73 (0.31-1.70)	

Paganini-Hill and Barreto 2001 [265]	To identify risk factors and preventative measures for stroke in elderly men and women.	• n = 4,722 men, 8,532 women	Baseline survey in 1981- 1982.	Multivariate adjusted RR (95% CI) for total hemorrhagic occlusion by exercise, men	Emphasized role of lifestyle modification in the primary prevention of stroke.
		• Sex: Men and women			
			PA assessment: Questionnaire on amount of hours per day of exercise	• Q1 = 1.00 (referent)	
USA		Age: 44-101 yr		• Q2 = 0.88	
		• Characteristics: no previous history of stroke. Residence of a retirement community in Southern California		Q3 = 0.83	
Prospective cohort			G1 = <0.5		
			G2 = <0.1	Multivariate adjusted RR (95% CI) for total hemorrhagic occlusion by exercise, women	
			G3 = 1+		
D & B score = 13					
			Outcome Measure: Incidence of hemorrhagic occlusion strokes up until December 31, 1998.	• Q1 = 1.00 (referent)	
				• Q2 = 0.91	
				• Q3 = 0.85	
			Poisson Regression 40 year follow up		

Pitsavos et al 2004 [266]	To investigate the interaction between PA in men with LVH on stroke mortality.	• n = 489		Number of cases: 67	PA reduced the risk of stroke in men without LVH.
		• Sex: Men			
			PA assessment: Questionnaire	RR (95% CI)	
USA		• Age: 40-59 yr		• G1 = 1.00 (referent)	
		• Characteristics: Those without LVH	G1 = Sedentary	• G2 = 0.64 (0.45-0.91)	
Prospective cohort			G2 = Moderate	• G3 = 0.72 (0.51-1.02)	
		• Corfu Cohort (Greece) from Seven Countries Study	G3 = Hard		
D & B score = 12			Outcome Measure: Stroke mortality		
			Cox proportional HR		

Sacco et al 1998 [267]	To investigate the association between LTPA and ischemic stroke.	• n = 369 case, 678 control	Case Subjects were recruited during hospitalization, self referral or from monitoring non hospitalized stroke. Controls were eligible if they had never been diagnosed with stroke and were >39 years.		LTPA was related to a decreased occurrence of ischemic stroke in elderly, multiethnic, urban subjects.
		• Sex: Men and women		O R (95% CI) for duration of LTPA and stroke	
USA					
		• Age: > 39 yr		• G1 = 1.00 (referent)	
Case control		• Characteristics: Case Subjects: Diagnosed with first cerebral infarction after July 1, 1993. Control Subjects: Never diagnosed with stroke		• G2 = 0.42	
				• G3 = 0.35	
D & B score = 14				• G4 = 0.31	
			PA assessment:		
			Questionnaire		
			Divided into duration of LTPA (h/wk)		
		• Northern Manhattan Stroke Study			
			G1 = 0		
			G2 = <2		
			G3 = 2-<5		
			G4 = ≥ 5		
			Multivariate conditional logistic regression Baseline data collection from 1982-1983 in East Boston (MA), New Haven (CT) and Iowa and Washington counties (IA).		

Simonsick et al 1993 [268]	To examine the association between recreational PA among physically capable older adults and incidence of selected chronic diseases and mortality over 3 and 6 years.	• n = 1,815		After 3 years Iowa	No consistent relationship between PA and stroke was found after 3 or 6 years across all 3 population cohorts.
		• Sex: Men and women			
		• Age: ≥ 65 yrs		OR (95% CI) Stroke and activity level	
USA		• Characteristics: Physically capable to do heavy work around the house, walk up and down a flight of stairs and walk a half mile without help.		• T1 = 0.22 (0.08-0.61)	
				• T2 = 1.05 (0.60-1.84)	
Prospective cohort				• T3 = 1.00 (Referent)	
			PA assessment: Questionnaire		
				New Haven	
D & B score = 12			T1 = High	OR (95% CI) Stroke and activity level	
			T2 = Moderate and	• T1 = 1.06 (0.38-2.95)	
			T3 = Inactive	• T2 = 1.26 (0.54-2.92)	
		• Established Populations for Epidemiologic Studies of the Elderly		• T3 = 1.00 (Referent)	
			Outcome Measure: Stroke incidence during 3 and 6 year follow-ups.		
				East Boston	
				OR (95% CI) Stroke and activity level	
				• T1 = 0.59 (0.17-1.95)	
			Logistic Regression		
				• T2 = 1.08 (0.52-2.27)	
				• T3 = 1.00 (Referent)	
				After 6 years	
				Iowa	
				OR (95% CI) Stroke and activity level	
				• T1 = 0.56 (0.31-1.00)	
				• T2 = 0.97 (0.64-1.48)	
				• T3 = 1.00 (Referent)	
				New Haven	
				OR (95% CI) Stroke and activity level	
				• T1 = 1.05 (0.52-2.12)	
				• T2 = 1.29 (0.72-2.32)	
				• T3 = 1.00 (Referent)	
				East Boston	
				OR (95% CI) Stroke and activity level	
				• T1 = 1.21 (0.56-2.61)	
				• T2 = 1.73 (0.98-3.06)	
				• T3 = 1.00 (Referent)	

Thrift et al 2002 [269]	To examine whether intracerebral hemorrhage is associated with dynamic or static exercise.	• n = 662	PA assessment: Interview, divided into 3 groups: frequency of vigorous activity	Number of Cases: 331	Findings not significant after multivariate analysis.
		• Sex: Men and women			
		• Age: 18-80 yr		Multivariate OR (95% CI) by frequency of VPA	
Australia		• Characteristics: Cases: first episode ofintracerebral hemorrhage Controls: Neighbours of cases			
			G1 = Never	• G1 = 1.00 (referent)	
Case control			G2 = Rarely	• G2 = 0.68 (0.36-1.27)	
			G3 = Once or more per month	• G3 = 0.66 (0.39-1.11)	
D & B score = 14				*p *= 0.094	
			OPA level	Multivariate OR (95% CI) by OPA level	
			G1 = Sedentary	• G1 = 1.00 (referent)	
			G2 = Light to moderate	• G2 = 0.94 (0.59-1.48), p = 0.773	
			G3 = Heavy	• G3 = 1.18 (0.57-2.46), p = 0.650	
			Outcome Measure: Intracerebral hemorrhage		
			Multiple logistic regression		

D & B score, Downs and Black quality score; YR, years; wk, week; CVD, cardiovascular disease; G, groups; PA, physical activity; CHD, coronary heart disease; RR, risk ratio; 95% CI, 95% confidence interval; T, tertile; PF, physical fitness; MET, metabolic equivalent; Q, quartile or quintile; OPA, occupational physical activity; LTPA, leisure-time physical activity; HR, hazard ratio; VPA, vigorous physical activity; LVH, left ventricular hypertrophy.

The data providing dose-response information is all observational in nature, involving both case control and cohort investigations. These studies (predominantly prospective cohort designs) included a total of 479,336 participants; averaging 17,753 subjects per study (range 428-73,265). There were a total of 12,361 reported cases of stroke (ranging per study from 32-2,863). The total length of study follow-up for the prospective cohort studies averaged 13.2 yr (ranging from 6-26 yr). The articles were published over a 14 yr period ranging from 1993 to 2007. These studies involved large samples of men and women from regions throughout the world including studies from the USA (11), UK (2), Iceland (1), Denmark (2), Norway (4), Netherlands (1), Finland (2), Japan (1), Australia (1) and Greece (1). Very few studies [69,70] examined non-Caucasian participants.

*We found strong evidence that physical activity was associated with a reduced risk for stroke. The level of evidence was consistent with a Level 3A classification*. We observed an average risk reduction of 31% across all studies (median = 29%). In comparison to cardiovascular disease, there was more variability in the risk reductions in stroke in the highest activity/fitness group. The quality of the investigations was also generally quite good with a mean (and median) Downs and Black score of 13 (range 11-15).

The risk reductions appear to be even greater in studies that assessed physical fitness directly. For instance, in data from the Aerobics Center Longitudinal Study [71] the high fitness group (estimated peak METs = 13.1) and the moderate fitness group (estimated peak METs 10.5) had significantly lower risks of stroke mortality (68 and 63%, respectively) than the least fit men (estimated peak METs 8.5).

A dose-response relationship did emerge when examining the literature. However, as illustrated by others this was extremely variable amongst studies and varied according to the type of stroke (ischemic or haemorrhagic) [52]. For instance, 12 studies (46%) revealed a dose-response relationship in one or more measures of occupational and/or leisure-time physical activity and the risk for stroke. It is difficult to determine the minimal and optimal physical activity dosage for the prevention of stroke. Brisk walking has been associated with a lower risk of total and ischemic stroke [72]. In the Harvard Alumni study, the risk of stroke was lower at a weekly energy expenditure of 4.2-8.4 MJ/wk (1000-1999 kcal/wk) (RR = 0.76 (95% CI, 0.59 to 0.98)). With expenditures of 8.4-12.6 MJ/wk (2000-2999 kcal/wk) the RR dropped to 0.54 (0.38 to 0. 76) [73]. Thus, the recommended daily expenditure of Canada's physical activity guidelines is sufficient to reduce the risk for stroke. Further research is required to clearly determine the risk reductions at exercise volumes less than 4.2 MJ/wk (1000 kcal/wk).

In summary, the results of these studies (taken as a whole) indicate that occupation- and leisure time-related physical activity are inversely related to the risk for stroke. Both physically active men and women have a lower risk of stroke, and it appears that this benefit may be present for both ischemic and haemorrhagic stroke [74]. The relationship between physical activity and stroke appears to be consistent between men and women. Unfortunately, relatively limited data exists in non-Caucasian populations.

*Recommendation #3*

*For a reduced risk of stroke, it is recommended that individuals should participate in 30 min or more of moderate to vigorous exercise on most days of the week. Brisk walking appears to be protective against the development of stroke. It remains to be determined whether lower volumes of physical activity lead to a reduced risk for stroke. [Level 3, Grade A]*

### Primary Prevention of Hypertension

**Figure 6:**
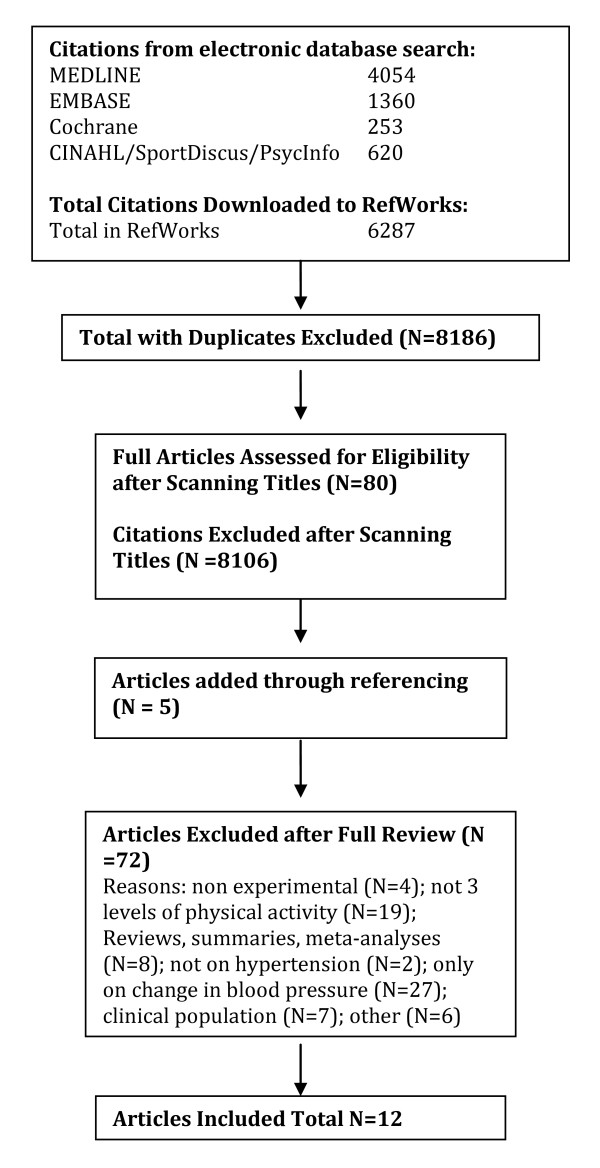
**Results of the Literature Search for Hypertension**.

A total of 6287 citations were identified during the electronic database search (Figure 6). Of these citations, 4054 were identified in MEDLINE, 1360 in EMBASE, 253 in Cochrane, and 620 in the CINAHL/SportDiscus/PsychInfo search. A total of 40 duplicates were found, leaving a total of 6247 unique citations. A total of 6167 articles were excluded after scanning, leaving a total of 80 articles for full review. An additional five articles were found through cross-referencing and the reviewers' personal files. From these articles 72 were excluded after full review for the following reasons: weak design (n = 4), did not contain three levels of physical activity or not possible to determine dose-response relationship (n = 19), reviews, summaries, meta-analyses (n = 8), not dealing with hypertension (n = 2), only reported on changes in blood pressure (n = 27), clinical population (n = 7), and other (n = 6). Therefore, a total of 12 articles were included in the systematic review of the literature regarding the relationship between physical activity and the primary prevention of hypertension. The majority of the literature examining the dose-response (for at least three levels of physical activity/fitness) involved prospective cohort analyses (83%).

As shown in Table 14, 12 investigations examined the dose-response (i.e., three or more levels) relationship between physical activity and the incidence of hypertension. This involved a total of 112,636 participants, averaging 10,240 subjects per study (range 1,243-41,837). There were a total of 11,441 reported cases of hypertension (ranging per study from 118-2,936). The total length of study follow-up averaged 8.6 yr (ranging from 0-16 yr). The articles were published over a 24 yr period ranging from 1983 to 2007.

**Table 14:** Studies examining the relationship between physical activity and hypertension.

Publication Country Study Design Quality Score	Objective	Population	Methods	Outcome	Comments and Conclusions
Rankinen et al 2007 [75]	To investigate the contributions of DNA sequence variation in candidate genes, PF and BMI, as well as their interactions to the incidence of hypertension.	• n = 629 cases; 605 controls	10 year follow up	PF showed the strongest association with HTN risk among all subjects as well as sex-specific models. Each 1- MET increment in PF was associated with 19% (12- 14%), 16% (9-22%), 32% (17- 45%) risk reduction in all subjects, men and women respectively.	PF is a significant predictor of the risk of hypertension.
USA		• Sex: Men and women	All subjects required to have 2 clinic visits at least 2 years apart.		
Case control		• Age: Case: 43.3 (9.2) yr Control: 42.7 (8.9) yr	PF assessment: treadmill test (Blake protocol)		
D & B score = 13		• Characteristics: Healthy with BP 134/86 mmHg or less at their first clinic visit. Cases: those who developed hypertension during the follow-up period. Controls were those who did not develop hypertension	Outcome measure: Incidence of hypertension during follow-up. Incident cases of hypertension were defined as physician diagnosed hypertension with medication or SBP ≥ 140 mmHg and/of DBP ≥ 90 mmHg t-tests and chi-square tests Logistic regression modelling	When divided into quartiles on the basis of sex specific MET cut-offs, the third and fourth quartiles had a 58% (41-71%) and 63% (47-75%) lower risk of hypertension compared to the 1^*st *^quartile.	

Pereira et al 1999 [76]	To examine PA and incident hypertension in men and women.	• n = 7,459	PA Assessment: Questionnaire for leisure, sport and work index, divided into quartiles	White Men	There is an inverse association between PA and incident hypertension in White middle aged men. White men in the highest quartiles of sport and leisure activity had statistically significant reductions in the odds of developing hypertension of 23 and 34% respectively, compared to men in the lower quartiles.
USA		• Sex: Men and women	Q1 = Lowest	Leisure Index Model 1	
Prospective cohort		• Age: 45-65 yr	Q2	• Q1 = 1.00 (referent)	
D & B score = 12		• Characteristics: No history of angina, MI, evidence of MI, angioplasty or other CV surgery or hypertension	Q3	• Q2 = 0.95 (0.70-1.28)	
		• Atherosclerosis Risk in Communities Study	Q4 = Highest	• Q3 = 0.83 (0.63-1.09)	
			Model 1 adjusted for: Age, education, baseline BP and study centre	• Q4 = 0.64 (0.46-0.89)	
			Model 2 adjusted for: Covariates in model 1 and smoking, alcohol consumption, parental history of hypertension, energy, sodium, potassium and caffeine intake, BMI, waist to hip ratio, menopausal status and hormone use	Trend *p *= 0.01	
			Outcome Measure: Incidence of hypertension as defined as a SBP 140 mmHg and/or a DBP 90 mmHg or use of antihypertensive medications.	Leisure Index Model 2	
			Unconditional logistic regression Orthogonal polynomial coefficients	• Q1 = 1.00 (referent)	
				• Q2 = 0.99 (0.72-1.35)	
				• Q3 = 0.86 (0.65-1.13)	
				• Q4 = 0.66 (0.47-0.94)	
				Trend *p *= 0.01	
				Women	
				Sport Index Model 1	
				• Q1 = 1.00 (referent)	
				• Q2 = 1.26 (0.78-2.05)	
				• Q3 = 1.06 (0.61-1.84)	
				• Q4 = 1.92 (1.12-3.29)	
				Trend *p *= 0.04	
				Men	
				Sport Index Model 1	
				• Q1 = 1.00 (referent)	
				• Q2 = 1.23 (0.91-1.66)	
				• Q3 = 0.92 (0.70-1.22)	
				• Q4 = 0.74 (0.54-1.02)	
				Trend *p *= 0.02	
				Sport Index Model 2	
				• Q1 = 1.00 (referent)	
				• Q2 = 1.26 (0.93-1.71)	
				• Q3 = 0.95 (0.71-1.26)	
				• Q4 = 0.77 (0.55-1.08)	
				Trend *p *= 0.05	

Haapanen et al 1997 [77]	To assess the association between PA and hypertension.	• n = 732 men; 796 women	10 year follow up (1980 baseline)	Age adjusted incidence rates ofhypertension Total energy expenditure High as referent:	Increased EE during LTPA and increased intensity of these activities were associated with reduced risk for incident hypertension (age adjusted) in men but not women.
Finland		• Sex: Men and women	PA assessement: Questionnaire for EE (kcal/wk), divided into tertiles		
Prospective cohort		• Age: 35-65 years	Male	Male:	
D & B score = 11		• Characteristics: Free of hypertension at baseline. Excluded those unable to participate in regular PA due to poor health	T1 = Low = 0-1100	• T1 = 1.00 (referent)	
			T2 = Medium = 1101-1900	• T2 = 1.66	
			T3 = High >1900	• T3 = 1.73	
				Trend *p *= 0.021	
			Female	Female:	
			T1 = Low = 0-900	• T1 = 1.00 (referent)	
			T2 = Medium = 901-1500	• T2 = 0.94	
			T3 = High = >1500	• T3 = 1.16	
			Outcome measure: Incidence of hypertension through self reported diagnosis and death certificates	Trend *p *= 0.648	
			Cox proportional HR		

Paffenbarger et al 1983 [78]	To examine the relationship of student and alumnus PA patterns and other characteristics with incident hypertension.	• n = 14,998	PA Assessment: Questionnaire for PA based on number of stairs ascended, blocks walked and hours per week of light and vigorous sports play, yard work etc.	There was no significantly reduced risk for hypertension in men who climbed 50 plus stairs per day (compared to < 50 stairs); who walked 5 plus blocks per day (compared to < 5 blocks); or who played light sports (compared to those who did not).	Contemporary vigorous exercise was inversely related to hypertension risk.
USA		• Sex: Men			
Prospective cohort		• Age: 35-74 yr	Outcome measure: Diagnosis of hypertension by physicians using criteria of SBP > 160 mmHg and/or DBP > 95 mmHg	The 59% of men who did engage in vigorous sports were at 35% greater risk of hypertension than the 41% who did not.	
				RR = 1.35	
				Trend *p *= <0.001	
D & B score = 12		• Characteristics: free of hypertension Harvard Alumni Study	Multivariate estimates	Alumni on the low side of the physical activity index (< 2000 kcal/wk) had a 30% increased risk of hypertension then those ≥ 2000 kcal/wk.	
				RR = 1.30	
				Trend *p *= 0.004	

Paffenbarger et al 1997 [79]	To investigate the quantity and intensity of energy expenditure required to delay hypertension and prevent premature death.	• n = 6,390	PA Assessment: Questionnaire for weekly sports play, divided into tertiles	RR (95% CI)	Lack of vigorous sports play independently increased the risk of developing hypertension.
USA		• Sex: Men	T1 = None	• T1 = 1.00 (referent)	
Prospective cohort		• Age: 45-84 yr	T2 = Light Only (< 4.5 METs)	• T2 = 1.04 (0.77-1.40)	
D & B score = 12		• Characteristics: Free of hypertension, CHD, diabetes, COPD and potentially malignant cancer in 1977	T3 = Moderately vigorous (≥ 4.5 METs)	• T3 = 0.77 (0.62-0.96)	
		• Harvard Alumni Study	Outcome measure: Incident hypertension	Trend *p *= 0.004	

Hu et al 2004 [81]	To discover whether regular PA can reduce the risk of hypertension in normal weight and overweight men and women.	• n = 8,302 men; 9,139 women	11 year follow up	Multivariate adjusted HR (95% CI), men	Regular PA can reduce the risk of hypertension. The protective effect of PA was observed in both sexes regardless of level of obesity.
Finland		• Sex: Men and women	PA assessement: Questionnaire for OPA, LTPA and commuting PA, divided into tertiles	• T1 = 1.00 (referent)	
Prospective cohort		Age: 25-64 yr	T1 = Low	• T2 = 0.63	
D & B score = 13		Characteristics: Healthy and free of hypertension at baseline	T2 = Medium	• T3 = 0.59	
			T3 = High	Trend *p *= < 0.001	
			Outcome Measure: Incidence of drug treated hypertension	Multivariate adjusted HR (95% CI), women	
			Cox proportional HR	• T1 = 1.00	
				• T2 = 0.82	
				• T3 = 0.71	
				Trend *p *= 0.005	

Gu et al 2007 [82]	To determine the 8-year incidence of HTN and its risk factors among Chinese adults.	• n = 10,525	Baseline Examination in 1991 with 8 year follow up	RR (95% CI), men	Increasing PA has the potential to reduce incidence of hypertension.
China		• Sex: Men and women	PA assessment: Questionnaire administered by trained staff, divided into groups	• G1 = 1.00 (referent)	
Prospective cohort		Age: ≥ 40 yr	G1 = Low	• G2 = 1.12 (0.86-1.46)	
D & B score = 13		Characteristics: Healthy and free from hypertension at baseline.	G2 = Medium	• G3 = 1.27 (1.10-1.47)	
			G3 = High	RR (95% CI), women	
			Outcome measure: HTN as defined at SBP ≥ 140 mmHg and/or DBP ≥ 90 mmHg or current use of antihypertensive medication	• G1 = 1.00 (referent)	
			t-tests, chi squared tests, Cochran-Armitage modeling, Modified Poisson approach	• G2 = 1.14 (0.98-1.34)	
				• G3 = 1.22 (1.02-1.45)	

Hayashi et al 1999 [83]	To investigate the association of the duration of the walk to work and LTPA with the risk for hypertension.	• n = 6,017	PA assessment: Questionnaire on health related behaviours and exercise Walk time to work	RR (95% CI) Frequency walk time to work (minutes	The duration of walk to work was associated with a decreased risk of hypertension even after adjustment.
Japan		• Sex: Men	T1 = 0-10 min	• T1 = 1.00 (referent)	Regular PA (at least once weekly) was inversely related to the risk of incident hypertension
Prospective cohort		• Age: 35-60 yr	T2 = 11-20 min	• T2 = 0.65 (0.47-0.90)	
D & B score = 12		• Characteristics: Free from HTN at baseline. All employees at gas company in Osaka Japan. All had sedentary jobs.	T3 = ≥ 21 min	• T3 = 0.72 (0.59-0.88)	
			Outcome measure: Diagnosed with hypertension (as defined by a SBP ≥ 160 mmHg, a DBP ≥ 95 mmHg, or use of antihypertensive medication)	Trend *p *= < 0.001	
			Cox proportional HR		

Nakanishi et al 2005 [84]	To examine the relationship of overall PA to the risk of developing hypertension in normotensive Japanese male office workers over a 7 year observation period.	• n = 2,548	7 year follow up	Multivariate adjusted RR (95% CI) by PA level only	The rate of rise in both SBP and DBP in each follow-up year decreased with higher EE and that the risk of developing hypertension decreased in a dose dependent manner with higher daily life activity level.
Japan		• Sex: Men		Q1 = 1.00 (referent)	Analysis stratified by the presence of or absence of a risk factor showed the negative association of daily life activity with the risk of developing hypertension for men at both low and high risk. This tendency was also observed among men in all 3 categories of normotension.
Prospective cohort		• Age: 35-59 yr	PA assessment: 1-day activity record and reported the type and frequency on a weekly basis of LTPA, divided into quartiles (kcal/kg/d)	Q2 = 0.84 (0.72-0.98)	
D & B score = 12		• Characteristics Healthy at baseline. No hypertension or CHD. All office workers for a Japanese company	• Q1 = <33.3	• Q3 = 0.75 (0.63-0.88)	
			• Q2 = 33.3-36.9	• Q4 = 0.54 (0.45-0.64)	
			• Q3 = 37.0-40.3	Trend *p *= < 0.001	
			• Q4 = 40.4	Multivariate adjusted RR (95% CI) by PA level, low normal BP	
			3 categories of normotensive BP Low Normal: SBP < 120, DBP < 80 Normal: SBP 120-130, DBP 80- 85 High Normal: SBP 130-139 DBP 85-89	• Q1 = 1.00 (referent)	
			3 categories of normotensive BP Low Normal: SBP < 120, DBP < 80 Normal: SBP 120-130, DBP 80- 85 High Normal: SBP 130-139 DBP 85-89	• Q2 = 0.70 (0.47-1.05)	
			Cox proportional hazard model	• Q3 = 0.55 (0.37-0.83)	
				• Q4 = 0.43 (0.28-0.65)	
				Trend *p *= <0.001	
				Multivariate adjusted RR (95% CI) by PA level, normal	
				BP	
				• Q1 = 1.00 (referent)	
				• Q2 = 0.89 (0.68-1.16)	
				• Q3 = 0.69 (0.52-0.91)	
				• Q4 = 0.50 (0.37-0.68)	
				Trend *p *= <0.001	
				Multivariate adjusted RR (95% CI) by PA level, high normal BP	
				• Q1 = 1.00 (referent)	
				• Q2 = 0.86 (0.69-1.07)	
				• Q3 = 0.88 (0.69-1.11)	
				• Q4 = 0.60 (0.46-0.78)	
				Trend *p *= 0.001	

Foy et al 2006 [85]	To examine whether insulin resistance is associated with the effect of vigorous or moderate PA on baseline BP.	• n = 1,599	Baseline examination in 1992-1993	Unadjusted OR (95% CI)	Participants who meet or exceed current caloric expenditure recommendations for VPA demonstrate significantly less hypertension than do sedentary or underactive individuals.
USA		• Sex: Men and women	PA assessment: VPA over the past year was determined via a 1-year recall of physical activity (kcal/d), divided into 3 groups	• T1 = 1.00 (referent)	
Cross sectional		• Age: 40-69 yr	• T1 = O	• T2 = 0.69 (0.53-0.88)	
D & B score = 12		• Characteristics: Community dwelling adults	• T2 = 1-149 kcal/day	• T3 = 0.57 (0.45-0.74)	
		• Insulin Resistance Atherosclerosis Study	• T3 = >150 kcal/day	• Trend *p *= < 0.001	
				Adjusted OR (95% CI)	
				• T1 = 1.00 (referent)	
				• T2 = 0.82 (0.62-1.09)	
				• T3 = 0.73 (0.55-0.98)	
				Trend *p *= 0.004	

Folsom et al 1990 [270]	To examine the relationship between fat distribution and the 2-yr incidence of hypertension and stroke.	• n = 41,837	Baseline mailed survey in 1986: Pa assessment: Questionnaire for LTPA	• 978 cases	High PA reduced the risk of hypertension only before adjusting for other factors.
USA		• Sex: Women	T1 = Low	Age Adjusted RR (95% CI)	
Prospective cohort		• Age: 55-69 years (yr)	T2 = Medium	• T1 = 1.00 (referent)	
D & B score = 12		• Characteristics: All free of HTN at baseline	T3 = High	• T2 = 0.9 (0.7-1.1)	
			Mantel-Haenszel method	• T3 = 0.7 (0.6-0.9)	
			Multiple logistic regression		

Levenstein et al 2001 [271]	To examine the effects of a variety of psychosocial factors on the development of HTN in men and women in the general population.	• n = 1,031 men, 1,326 women	Questionnaires in 1965 and 1974, cohort followed until 1994	LTPA predictor of hypertension OR (95% CI)	Risk of HTN was reduced with increases in LTPA in women.
USA		• Sex: Men and women	PA assessment: LTPA rated on a scale of 0-16 points and analysed as a continuous variable	• All Subjects: 0.94 (0.91-0.97)	
Prospective cohort		• Characteristics: Free of hypertension at baseline	Outcome measure: Incidence of hypertension (defined as those who are taking antihypertensive medications)	• Women: 0.90 (0.87-0.94)	
D & B score = 13		• Alameda cohort study	Logistic regression analysis	• Men: 0.98 (0.94-1.02)	

D & B score, Downs and Black quality score; YR, years; PF, physical fitness; BMI, body mass index; MET, metabolic equivalent; PA, physical activity; MI, myocardial infarction; G, groups; Q, quartile or quintile; 95% CI, confidence interval; SBP, systolic blood pressure; DBP, diastolic blood pressure; EE, energy expenditure; kcal/wk, kilocalories per week; T, tertile; RR, risk ratio; HR, hazard ratio; CHD, coronary heart disease; COPD, chronic obstructive pulmonary disease; OPA, occupational physical activity; LTPA, leisure-time physical activity; BP, blood pressure; kcal/day, kilocalories per day.

All studies reviewed demonstrated positive effects of physical activity on the risk for hypertension. Of these studies all (7; 58%) revealed an inverse and graded relationship between hypertension and at least one measure of physical activity or fitness. Across all studies, when comparing the most active/fit group versus the least active/fit group we found an average RR of 0.68 (median = 0.70, range 0.37 to 0.90). Therefore, we observed that physical activity/fitness was associated with an average risk reduction of 32% for hypertension. It should be noted that the study [75] demonstrating the largest risk reduction (63%) evaluated cardiorespiratory fitness directly during a maximal treadmill test. This supports research (as discussed previously) which indicates that physical fitness is a better predictor of chronic disease than physical activity [6,18,19,32,33]. *Taken as a whole, the level of evidence can be classified as Level 3A*. The quality of studies was generally good with a mean Downs and Black score of 11 (median = 11, range = 10-12).

Five studies showed variable results (i.e., no clearly defined dose-response) while generally supporting the inverse relationship between physical activity/fitness and hypertension [76-80]. The variability in the response appears to be the result of different activity/fitness classifications and/or differing subject populations. For instance, some studies revealed that the dose-response relationships differed between genders and/or ethnicities [76,77]. Pereira et al. [76] revealed a 30% reduction in the risk for hypertension in the most active white men. There were graded dose-response relationships between indices of both leisure and sport activities in the white men.

However, there was a lack of association between physical activity and hypertension in white women and African American men and women. Similarly, Haapenen et al. [77] revealed a stronger association in men than in women. However, it should be noted clearly that other studies included in this systematic review evaluated women demonstrating a graded response [81]. Moreover, several studies were conducted with non-Caucasian populations and demonstrated a dose-dependent benefit [82-85]. In fact, data was obtained from varied regions of the world including USA (7), Japan (2), China (1), and Finland (1). Therefore, there is evidence to suggest that the protective effects of physical activity with respect to hypertension are transferable to women and non-Caucasian populations. However, further research is clearly warranted that examines the relationship between physical activity and hypertension in persons of different ethnicities. Moreover, further research is needed to determine the effects of impact of socio-economic status on the observed relationships.

Some studies have indicated that vigorous activity is required to reduce the risk for hypertension. For instance, Paffenbarger [78] revealed that Harvard Alumni who did not engage in vigorous sports play were at a 35% higher risk for developing hypertension. However, there was no difference in the risk for hypertension in men who climbed >50 stairs per day, walked more than 5 city blocks daily, or engaged in light sports only. Similarly, the Paffenbarger and Lee [79] study revealed that moderately vigorous sports play was associated with a lower risk for hypertension, but physical activity (kcal/wk), walking distance (km/wk) and the amount of stairs climbed (floors/wk) were not significant predictors of the risk for hypertension. Collectively, this research group concluded that these findings highlighted the importance of the intensity of effort.

However, it should be noted that many of the studies in our systematic review observed the protective effect with moderate intensity physical activities. Findings from randomized controlled trials have also provided strong evidence that moderate intensity aerobic exercise is sufficient to reduce blood pressure and the risk for hypertension, particularly in at risk individuals [86,87]. The American College of Sports Medicine [88] recently advocated that to prevent hypertension, individuals should exercise on most, and preferably all, days of the week at a moderate intensity, for 30 min or more per day (continuous or accumulated). They also recommended supplementing endurance type activities with resistance exercise. This is supported by research indicating that moderate intensity resistance training can reduce blood pressure [89]. Collectively, this research and our current summary of the dose-response literature indicates that physical activity levels that are of a moderate to vigorous intensity are sufficient to lead to marked reductions in the risk for hypertension.

#### Implications

The impact of hypertension on North American society is enormous. In the US, 31% of non-institutionalized adults over the ages of 20 are currently thought to have hypertension [90]. In Canada, approximately 20% of adults report a diagnosis of hypertension including over 4 million Canadians [91-93]. It has been estimated that a 55 yr old Canadian with normal blood pressure has a greater than 90% chance of developing hypertension before the age of 80 yr [92]. The primary prevention of hypertension is of paramount importance to the attenuation of the risks and costs associated with hypertension and related comorbidities.

There is clear evidence that routine physical activity and/or increased physical fitness reduce greatly the risk for hypertension in both normotensive and hypertensive individuals [18,19]. Extensive research has been conducted in the area including numerous prospective trials and various randomized controlled trials. Numerous reviews of the literature (of epidemiological and randomized controlled trials) have supported an inverse relationship between physical activity/fitness and in the incidence of hypertension [20,87,89,94-102]. In a recent systematic review of the prospective literature, Katzmarzyk and Janssen (2004) calculated that physically inactive individuals were at a 30% higher risk for hypertension (RR = 1.30 (95% CI = 1.16-1.46)) with a population attributable risk of 13.8% in Canada [20]. Acute bouts of exercise have also been shown to lead to transient changes in blood pressure that are potentially of health benefit [98]. For instance, blood pressure is often reduced after a single exercise session for 12-22 hr [88,103].

It is clear that routine physical activity is effective in both the primary and secondary prevention of hypertension. However, the optimal dosage of physical activity/exercise remains somewhat unclear. Our review of the literature examined critically the relationship between multiple levels of physical activity/fitness and the incidence of hypertension (in individuals without diagnosed hypertension). As identified above this evidence was compelling supporting the protective effects of habitual physical activity in the primary prevention of hypertension.

*Recommendation #4*

*For a reduced risk for hypertension, it is recommended that individuals should participate in 30 min or more of moderate to vigorous exercise on most days of the week. [Level 3, Grade A]*

### Primary Prevention of Colon and Breast Cancer

#### Colon Cancer

**Figure 7:**
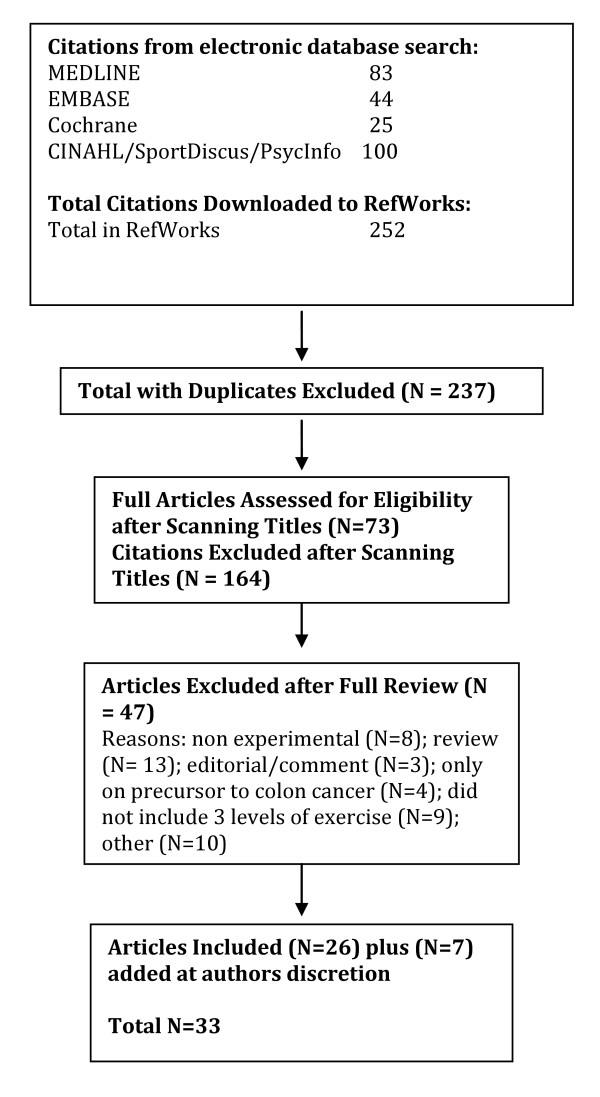
**Results of the Literature Search for Colon Cancer**.

In our systematic search of the colon cancer literature, a total of 252 citations were identified during the electronic database search (Figure 7). Of these citations, 83 were identified in MEDLINE, 44 in EMBASE, 25 in Cochrane, and 100 in the CINAHL/SportDiscus/PsychInfo search. A total of 15 duplicates were found, leaving a total of 237 unique citations. A total of 164 articles were excluded after screening, leaving a total of 73 articles for full review. From these articles 47 were excluded after full-text review leaving 26 articles for inclusion, and an additional 7 articles were added from the authors' personal files. The reasons for exclusion included non-experimental/weak design (n = 8), reviews, summaries, meta-analyses (n = 13), editorial/comment (n = 3), not dealing specifically with colon cancer (n = 4), did not contain three levels of physical activity or not possible to determine dose-response relationship (n = 9), and other (n = 10). Therefore, a total of 33 articles were included in the systematic review of the literature regarding the relationship between physical activity and the primary prevention of colon cancer.

These studies involved a total of 1,433,103 participants; averaging 43,427 participants per study (range 142-413,044). There were a total of 17,959 reported cases of colon cancer (ranging per study from 93-1,993). The total length of study follow-up for the prospective cohort studies averaged 10.7 yr (ranging from 4-26 yr). The articles were published over a 23 yr period ranging from 1985 to 2008. These studies involved large samples of men and women from regions throughout the world.

A dose-dependency of this relationship was present in the majority of the studies. When comparing the most active/fit group versus the least active/fit group we found a mean risk reduction of 30% (median = 32%) across all studies. The most compelling literature was that which evaluated the relationship between moderate-to-vigorous leisure time physical activity. Based on the literature reviewed and the volume of activity assessed it would appear that Canada's guidelines for physical activity are sufficient to lower the risk for the development of colon cancer in asymptomatic adults. *The level of evidence would be considered to be Level 2A*. The studies were generally of a higher quality with a mean Downs and Black score of 13 (median = 14, range = 11-15).

It should be noted that there was considerable variability in the findings and conclusions of the studies (Table 15). As discussed later, the literature was further confounded by the fact that the relative risks associated with physical activity were often controlled (through multivariate analyses) for various potential confounding factors, which may actually inappropriately decrease the level of risk reduction associated with physical activity [31]. Moreover, similar to other chronic conditions this literature was limited greatly by the lack of consistent physical activity assessment and description. In many instances, it was difficult to determine the actual absolute volume and/or intensity of activity for each category of comparison. However, despite these limitations the results of these studies (taken as a whole) indicate that both occupation- and leisure time-related physical activity are inversely related to the risk of colon cancer.

**Table 15:** Studies examining the relationship between physical activity and colon cancer.

Publication Country Study Design Quality Score	Objective	Population	Methods	Outcome	Comments and Conclusions
Hou et al 2004 [272]	To examine the effect of various forms of PA on colon cancer risk, with particular attention to commuting PA.	• n = 931 case, 1,552 control	PA assessment: Interview for the following variables	• Number of cases: 931	Regular frequent PA over a long period of time reduces risk of CC.
China		• Sex: Men and women		Multivariate OR (95% CI) by OPA, men	
				• G1 = 1.00 (referent)	
Case control		• Age: 30-74 yr	OPA (kJ/min)	• G2 = 1.23 (0.93-1.64)	
D & B score = 14		• Characteristics: Case: diagnosed with CC. controls: selected randomly from residents of urban Shanghai.	G1 = <8	• G3 = 0.81 (0.59-1.19)	
			G2 = 8-12	*p *= 0.10	
			G3 = >12		
			Commuting PA (MET hr/wk)	Multivariate OR (95% CI) by OPA, women	
			G1 = <48.3	• G1 = 1.00 (referent)	
			G2 = 48.3-94.3	• G2 = 0.96 (0.69-1.16)	
			G3 = >94.3	• G3 = 0.64 (0.39-1.02)	
				*p *= 0.009	
			LTPA (MET hr/wk)	Multivariate OR (95% CI) Commuting PA, men	
			G1 = < 9.2	• G1 = 1.00 (referent)	
			G2 = 9.2-13.6	• G2 = 1.11 (0.31-1.23)	
			G3 = >13.6	• G3 = 0.52 (0.27-0.87)	
			Outcome Measure: incident CC	*p*<0.001	
			Multiple logistic regression	Multivariate OR (95% CI) Commuting PA, women	
				• G1 = 1.00 (referent)	
				• G2 = 0.87 (0.42-1.52)	
				• G3 = 0.56 (0.21-0.91)	
				*p *= 0.007	
				Multivariate OR (95% CI) LTPA, men	
				• G1 = 1.00 (referent)	
				• G2 = 1.17 (0.13-1.95)	
				• G3 = 0.72 (0.41-1.07)	
				*p *= 0.06	
				Multivariate OR (95% CI) LTPA, women	
				• G1 = 1.00 (referent)	
				• G2 = 1.03 (0.41-1.59)	
				• G3 = 0.84 (0.13-2.25)	
				*p *= 0.15	

Boutron-Ruault et al 2001 [273]	To determine which step of the adenoma-carcinoma pathway was influenced by OPA and recreational PA.	• n = 480	PA assessment: Questionnaire and classified into 3 groups	Number of cases: 171	A sedentary lifestyle was associated with a high risk of CC.
France		• Sex: Men and women	G1 = Low	Age and gender adjusted OR (95% CI), OPA	
Case control		• Age: 30-79 years	G2 = Medium	• G1 = 1.00 (referent)	
D & B score = 13		• Characteristics: Cases had 1^st^diagnosis of colorectal adenoma, controls were polyp free.	G3 = High	• G2 = 1.3 (0.8-2.0)	
				• G3 = 0.5 (0.3-0.9)	
				*p *= 0.005	
			Outcome Measure: Incident CC		
			Multiple logistic regression	Age and gender adjusted OR (95% CI), LTPA	
				• G1 = 1.00 (referent)	
				• G2 = 0.7 (0.4-1.1)	
				• G3 = 0.3 (0.2-0.5)	
				*p *= <0.0001	
				Age and gender adjusted OR (95% CI), Global PA	
				• G1 = 1.00 (referent)	
				• G2 = 0.8 (0.5-1.2)	
				• G3 = 0.3 (0.2-0.6)	
				*p *= 0.0003	

Brownson et al 1991 [274]	To investigate the risks of 16 cancer types in relation to OPA.	• n = 17,147	PA assessment: Medical records and classified into 3 groups:	Number of cases: 1,838	OPA is inversely related to risk of CC.
		• Sex: Men		Multivariate OR (95% CI)	
USA		• Age: ≥ 20 yr	OPA	G1 = 1.00 (referent)	
		• Characteristics: White, working	G1 = Low - Activity required <20% of time	G2 = 1.2 (1.0-1.5)	
Case controll			G2 = Moderate - Activity required 20-80% of time	G3 = 1.1 (1.0-1.3)	
D & B score = 15			G3 = High - Activity required >80% of time	*p *= 0.05	
			Outcome Measure: CC		
			Maximum likelihood estimates		

Calton et al 2006 [275]	To examine the relationship between PA and colon cancer risk in women.	• n = 31,783	11 year follow up	Number of cases: 243	Results do not support the hypothesis that PA is related to a lower incidence of CC in women.
USA		• Sex: Women	PA Assessment: Questionnaire / Phone interviews for the following variables, divided into 4 or 5 groups		
Prospective cohort		• Age: 61.1 yr		Multivariate RR (95% CI), TPA	
D & B score = 12		• Characteristics: Free from cancer at baseline		• G1 = 1.00 (referent)	
				• G2 = 1.45 (0.98-2.15)	
				• G3 = 1.16 (0.77-1.75)	
				• G4 = 1.27 (0.84-1.91)	
				• G5 = 1.15 (0.76-1.75)	
				*p *= 0.77	
			TPA (MET h/d)		
			G1 = 34.0-48.5	Multivariate RR (95% CI), MPA	
			G2 = 48.51-54.3	• G1 = 1.00 (referent)	
			G3 = 54.31-59.0	• G2 = 1.23 (0.82-1.83)	
			G4 = 59.1-64.9	• G3 = 1.47 (0.99-2.21)	
			G5 = 65.0-98.1	• G4 = 0.94 (0.61-1.46)	
				• G5 = 1.07 (0.70-1.62)	
			MPA (h/d)	*p *= 0.80	
			G1 = 0-3.0		
			G2 = 3.01-5.0		
			G3 = 5.01-6.70	Multivariate RR (95% CI), VPA	
			G4 = 6.71-8.14	• Q1 = 1.00 (referent)	
			G5 = 8.15-18.0	• Q2 = 1.19 (0.85-1.66)	
				• Q3 = 0.87 (0.59-1.29)	
			VPA (h/d)	• Q4 = 1.10 (0.78-1.55)	
			Q1 = 0	*p *= 0.80	
			Q2 = 0.1-1.0		
			Q3 = 1.1-2.0		
			Q4 = 2.1-14.0		
			Outcome Measure: Incidence of CC		
			Cox proportional HR		

Chao et al 2004 [276]	To examine how the characteristics of recreational PA affect its association with colon cancer incidence among older.	• n = 151,174 (70,403 men; 80,771 women)	7 year follow up	Number of cases: 940	Increased amounts of time spent in recreational PA is associated with substantially lower risk of CC.
USA		• Sex: Men and women	PA assessment: Questionnaire for the following variables	Multivariate RR (95% CI) by recreational PA, men	
Prospective cohort		• Age: mean 63 yr		• G1 = 1.00 (referent)	
D & B score = 12		• Cancer prevention study II Nutrition Cohort		• G2 = 0.91 (0.69-1.19)	
			Recreational PA (h/wk)	• G3 = 0.72 (0.52-1.01)	
			G1 = None	• G4 = 0.86 (0.64-1.15)	
			G2 = <2	• G5 = 0.77 (0.54-1.08)	
			G3 = 2-3	• G6 = 0.58 (0.39- 0.87)	
			G4 = 4-6	*p *= 0.007	
			G5 = 7		
			G6 = ≥ 8	Multivariate RR (95% CI) by recreational PA, women	
			Recreational (MET h/wk)	• G1 = 1.00 (referent)	
			G1 = None	• G2 = 1.01 (0.70-1.44)	
			G2 = <7, 7-13	• G3 = 1.01 (0.68-1.49)	
			G3 = 14-23	• G4 = 0.97 (0.66-1.43)	
			G4 = 24-29	• G5 = 1.03 (0.65-1.65)	
			G5 = ≥ 30	• G6 = 0.65 (0.39-1.11)	
				*p *= 0.14	
			Walking (h/wk)		
			Q1 = None		
			Q2 = <4	Multivariate RR (95% CI) by recreational PA, men and women	
			Q3 = 4-6	• G1 = 1.00 (referent)	
			Q4 = ≥ 7	• G2 = 0.94 (0.75-1.16)	
				• G3 = 0.83 (0.65-1.07)	
			Walking plus other	• G4 = 0.89 (0.71-1.12)	
			activities (h/wk)	• G5 = 0.85 (0.64-1.12)	
			Q1 = None	• G6 = 0.60 (0.44-0.83)	
			Q2 = <4	*p *= 0.002	
			Q3 = 4-6		
			Q4 = ≥ 7	Multivariate RR (95% CI) by MET h/wk men	
			Outcome Measure: Incidence of CC	• G1 = 1.00 (referent)	
				• G2 = 0.90 (0.68-1.18)	
			Cox proportional HR	• G3 = 0.83 (0.59-1.16)	
				• G4 = 0.75 (0.55-1.01)	
				• G5 = 0.86 (0.63-1.19)	
				• G6 = 0.60 (0.41-0.87)	
				*p *= 0.005	
				Multivariate RR (95% CI) by MET h/wk women	
				• G1 = 1.00 (referent)	
				• G2 = 1.02 (0.71-1.46)	
				• G3 = 0.98 (0.65-1.47)	
				• G4 = 1.0 (0.68-1.47)	
				• G5 = 0.94 (0.60-1.48)	
				• G6 = 0.77 (0.48-1.24)	
				*p *= 0.15	
				Multivariate RR (95% CI) by MET h/wk men and women	
				• G1 = 1.00 (referent)	
				• G2 = 0.93 (0.75-1.16)	
				• G3 = 0.88 (0.68-1.13)	
				• G4 = 0.84 (0.66-1.06)	
				• G5 = 0.89 (0.68-1.15)	
				• G6 = 0.65 (0.49-0.87)	
				*p *= 0.002	
				Multivariate RR (95% CI) by walking, Men	
				• Q1 = 1.00 (referent)	
				• Q2 = 0.87 (0.66-1.15)	
				• Q3 = 0.83 (0.60-1.16)	
				• Q4 = 0.88 (0.61-1.25)	
				*p *= 0.34	
				Multivariate RR (95% CI) by walking, women	
				• Q1 = 1.00 (referent)	
				• Q2 = 1.00 (0.70-1.44)	
				• Q3 = 1.08 (0.71-1.63)	
				• Q4 = 1.18 (0.71-1.95)	
				*p *= 0.41	
				Multivariate RR (95% CI) by walking plus other activities, men	
				• Q1 = 1.00 (referent)	
				• Q2 = 0.73 (0.53-1.02)	
				• Q3 = 0.85 (0.58-1.24)	
				• Q4 = 0.53 (0.36-0.78)	
				*p *= 0.02	
				Multivariate RR (95% CI) by walking plus other activities, women	
				• Q1 = 1.00 (referent)	
				• Q2 = 0.99 (0.67-1.47)	
				• Q3 = 0.72 (0.43-1.19)	
				• Q4 = 0.59 (0.36-0.98)	
				*p *= 0.07	

Colbert et al 2001 [277]	To examine the association between OPA and LTPA and colon cancer in male smokers.	• n = 29,133	12 year follow-up	Number of cases: 152	OPA is protective against CC in a dose-response manner.
USA		• Sex: Men			
Prospective cohort		• Age: 50-69 yr	PA assessment: Interview for OPA and LTPA	Multivariate RR (95% CI) by OPA	
D & B score = 13		• Characteristics: Smokers		• G1 = 0.61 (0.39-0.98)	
		• Alpha- Tocopherol, Beta-Carotene Cancer Prevention Study		• G2 = 1.00 (referent)	
				• G3 = 0.60 (0.34-1.04)	
			OPA	• G4 = 0.45 (0.26-0.78)	
			G1 = Non-worker	*p *= 0.003	
			G2 = Sedentary		
			G3 = Light	Multivariate RR (95% CI), by LTPA	
			G4 = Moderate	• G1 = 1.00 (referent)	
				• G2 = 0.82 (0.59-1.13)	
			LTPA		
			G1 = Sedentary		
			G2 = Active		
			Outcome Measure: incident CC		
			Cox proportional HR		

Dosemeci et al 1993 [278]	To examine associations between PA and cancer sites among workers in Turkey.	• n = 6,236 (3,486 cases in men and 379 cases in women; 2,127 control men and 244 control women)	PA assessment: Stanford Occupational Classification code system.	Number of cases: 93	Occupational EE is inversely related to risk of CC.
Turkey		• 93 cases for CC		Multivariate OR (95% CI) by total occupational EE	
Case control		• Sex: Men and women		• G1 = 1.6 (0.9-2.8)	
		• Age: not indicated	Total Occupational EE (kj/min)	• G2 = 1.1 (0.6-2.0)	
D & B score = 13		• Characteristics: All hospitalized Cases: Diagnosed with CC. Controls: included subjects diagnosed as non-cancers and cancers which there is no suggestion of an association with PA.	G1 = <8	• G3 = 1.0 (referent)	
			G2 = 8-12	*p *= 0.04	
			G3 = >12	When adjusted for socioeconomic status *p *= 0.03	
			Sitting time at work (h/d) Levels:	Multivariate OR (95% CI) by sitting time at work	
			G1 = <2	• G1 = 1.00 (referent)	
			G2 = 2-6	• G2 = 1.5 (0.9-2.5)	
			G3 = >6	• G3 = 1.5 (0.8-3.0)	
				*p *= 0.03	
			Outcome Measure: Incident CC	When adjusted for socioeconomic status p = 0.03	
			Maximum likelihood estimates		

Friedenreich et al 2006 [279]	To investigate the role of PA in the development of colon cancer.	• n = 413,044	4 year follow-up	Multivariate RR (95% CI), TPA	Inverse association between PA and risk of CC, particularly for right sided tumours.
		• Sex: Men and women	PA assessment: modified Baecke Questionnaire	• Q1 = 1.00 (referent)	
UK		• Age: 35-70 yr		• Q2 = 0.92 (0.76-1.12)	
		• Characteristics: Free of cancer at baseline		• Q3 = 0.86 (0.70-1.04)	
Prospective cohort		• European Prospective Investigation into Nutrition and Cancer. (EPIC)	TPA	• Q4 = 0.78 (0.59-1.03)	
			Q1 = Inactive	*p *= 0.04	
D & B score = 14			Q2 = Moderately inactive	Multivariate RR (95% CI), TPA and right sided CC	
			Q3 = Moderately active	• Q1 = 1.00 (referent)	
			Q4 = Active	• Q2 = 1.79 (0.59-1.06)	
			Household PA (MET-h/wk)	• Q3 = 0.64 (0.47-0.86)	
			Q1 = <19.5	• Q4 = 0.65 (0.43-1.00)	
			Q2 = 19.5-39.6	*p *= 0.004	
			Q3 = 39.6-73.9		
			Q4 = ≥ 73.9	Multivariate RR (95% CI), household PA and right sided CC	
				• Q1 = 1.00 (referent)	
				• Q2 = 0.97 (0.75-1.27)	
			Outcome Measure: Incident CC	• Q3 = 0.84 (0.64-1.12)	
				• Q4 = 0.74 (0.54-1.02)	
				*p *= 0.05	
			Cox proportional HR		

Giovannucci et al 1995 [280]	To examine the association between PA and colon cancer.	• n = 47,723	6 year follow-up	Multivariate RR (95% CI)	A moderate level of PA was related to a substantially lower risk of CC in this cohort of middle age to elderly men.
		• Sex: Men		• G1 = 1.00 (referent)	
		• Age: 40-75 yr	PA assessment: Questionnaire	• G2 = 0.73 (0.48-1.10)	
USA		• Characteristics: Health professionals		• G3 = 0.94 (0.63-1.39)	
				• G4 = 0.78 (0.51-1.20)	
Prospective cohort		• Health Professionals Follow-up Study	Outcome Measure: Incidence of colon cancer	• G5 = 0.53 (0.32-0.88)	
				*p *= 0.03	
D & B score = 12					
			Mantel-Haeszel estimator and logistic regression		

Isomura et al 2006 [281]	To examine the relationship of OPA, LTPA, commuting, housework and shopping with colorectal cancer risk.	• n = 1545 (778 cases, 767 controls)	PA assessment: Questionnaire and interview for the following variables	• Number of cases: 778	Adds to the evidence that PA confers decreased risk of CC, especially of distal CC in both men and women.
Japan		• Sex: Men and women		Multivariate OR (95% CI) for all CC by OPA, men	
				• G1 = 1.00 (referent)	
Case control		• Age: 20-74 yr	OPA, men	• G2 = 0.9 (0.6-1.4)	
		• Characteristics: Free from cancer at baseline	G1 = Sedentary	• G3 = 0.7 (0.4-1.0)	
D & B score = 12			G2 = Moderate	*p *= 0.06	
	.		G3 = Hard		
		• Fukuoka colorectal cancer study		Multivariate OR (95% CI) for proximal	
			OPA, women	CC by OPA, men	
			G1 = Sedentary	• G1 = 1.00 (referent)	
			G2 = Active	• G2 = 1.2 (0.6-2.2)	
				• G3 = 0.7 (0.4-1.4)	
			Total non-OPA, men (MET-h/wk)	*p *= 0.45	
			G1 = 0.0	Multivariate OR (95% CI) for distal CC by OPA, men	
			G2 = 0.1-15.9	• G1 = 1.00 (referent)	
			G3 = 16.0	• G2 = 0.8 (0.4-1.4)	
				• G3 = 0.6 (0.4-1.0)	
				*p *= 0.047	
			Total non-OPA women (MET hr/wk)		
			G1 = 0.0		
			G2 = 0.1-15.9		
			G3 = 16.0	Multivariate OR (95% CI) for all CC by non-OPA, men	
			Moderate or hard non-OPA, men (MET hr/wk)	• G1 = 1.00 (referent)	
			G1 = 0.0	• G2 = 0.9 (0.6-1.4)	
			G2 = 0.1-14.9	• G3 = 0.8 (0.5-1.2)	
			G3 = ≥15.0	*p *= 0.22	
				Multivariate OR (95% CI) for proximal CC by non-OPA, men	
			Moderate or hard non-OPA, women (MET hr/wk)	• G1 = 1.00 (referent)	
			G1 = 0.0	• G2 = 1.2 (0.6-2.1)	
			G2 = 0.1-14.9	• G3 = 0.9 (0.5-1.7)	
			G3 = 15.0	*p *= 0.69	
			Outcome Measure: Incident CC	Multivariate OR (95% CI) for distal CC by non-OPA, men	
				• G1 = 1.00 (referent)	
			Multiple logistic regression analysis	• G2 = 0.8 (0.5-1.3)	
				• G3 = 0.7 (0.4-1.1)	
				*p *= 0.19	
				Multivariate OR (95% CI) for all CC by non-OPA, women	
				• G1 = 1.00 (referent)	
				• G2 = 0.9 (0.5-1.5)	
				• G3 = 0.8 (0.5-1.4)	
				*p *= 0.45	
				Multivariate OR (95% CI) for proximal CC by non-OPA, women	
				• G1 = 1.00 (referent)	
				• G2 = 1.5 (0.7-3.3)	
				• G3 = 1.6 (0.7-3.6)	
				*p *= 0.41	
				Multivariate OR (95% CI) for distal CC by non-OPA, women	
				• G1 = 1.00 (referent)	
				• G2 = 0.7 (0.4-1.3)	
				• G3 = 0.6 (0.3-1.1)	
				*p *= 0.12	
				Multivariate OR (95% CI) for all CC by moderate or hard non-OPA, men	
				• G1 = 1.00 (referent)	
				• G2 = 0.8 (0.6-1.2)	
				• G3 = 0.8 (0.5-1.1)	
				*p *= 0.24	
				Multivariate OR (95% CI) for proximal CC by moderate or hard non-OPA, men	
				• G1 = 1.00 (referent)	
				• G2 = 1.1 (0.6-2.1)	
				• G3 = 1.0 (0.6-1.8)	
				*p *= 0.99	
				Multivariate OR (95% CI) for distal CC by moderate or hard non-OPA, men	
				• G1 = 1.00 (referent)	
				• G2 = 0.7 (0.4-1.1)	
				• G3 = 0.7 (0.4-1.0)	
				*p *= 0.12	
				Multivariate OR (95% CI) for all CC by moderate or hard non-OPA, women	
				• G1 = 1.00 (referent)	
				• G2 = 1.0 (0.6-1.6),	
				• G3 = 0.8 (0.5-1.4)	
				*p *= 0.35	
				Multivariate OR (95% CI) for proximal CC by moderate or hard non-OPA, women	
				• G1 = 1.00 (referent)	
				• G2 = 1.3 (0.6-2.5)	
				• G3 = 1.3 (0.6-2.7)	
				*p *= 0.59	
				Multivariate OR (95% CI) for distal CC by moderate or hard non-OPA, women	
				• G1 = 1.00 (referent)	
				• G2 = 0.8 (0.5-1.5)	
				• G3 = 0.5 (0.3-1.1)	
				*p *= 0.41	

Johnsen at el 2006 [282]	To investigate the effects of OPA on colon cancer incidence.	• n = 54,478 (28,356 men, 26,122 women)	7.6 year follow-up	• Number of cases: 140 women, 157 men	No support for the hypothesis that OPA measured by MET-score may be associated with a lower risk of CC.
		• Sex: Men and women	PA assessment: Questionnaire for OPA by MET score, 4 groups	• Number of dropouts: <0.8%	
Denmark		• Age: 50-64 yr		Multivariate RR (95% CI), men	
Prospective cohort		• Characteristics: Free of Cancer at baseline	Q1 = Sitting	• Q1 = 1.00 (referent)	
		• Diet, Cancer and Health Study	Q2 = Standing	• Q2 = 1.11 (0.69-1.77)	
			Q3 = Manual	• Q3 = 1.17 (0.77-1.79)	
D & B score = 13			Q4 = Not working	• Q4 = 0.95 (0.58-1.55)	
					
			Outcome Measure: Incidence of colon cancer	Multivariate RR (95% CI), women	
				• Q1 = 1.00 (referent)	
				• Q2 = 1.15 (0.68-1.93)	
				• Q3 = 1.34 (0.83-2.16)	
			Cox proportional HR	• Q4 = 0.96 (0.60-1.53)	

Larsen et al 2006 [283]	To examine the relationship between PA and colorectal cancer.	• n = 6,961	PA assessment: Questionnaire (scored from 2-12), divided into quartiles:	Number of cases: 108	Inactivity was not a significant risk factor for advanced colonic neoplasia.
		• Sex: Men and women		RR (95% CI)	
Norway		• Age: 50-64		• Q1 = 1.00 (referent)	
		• Characteristics: No history of colorectal surgery, radiotherapy, cardiopulmonary disease, anticoagulant therapy, coronary episode.	Q1 = 2-4	• Q2 = 0.61 (0.32-1.16)	
Cross-sectional evaluation within a randomized controlled trial			Q2 = 5	• Q3 = 0.75 (0.45-1.26)	
			Q3 = 6	• Q4 = 0.56 (0.34-0.92)	
			Q4 = 7-12	*p *= 0.04	
			Outcome Measure: Positive test for colonic neoplasia	Multivariate RR (95% CI)	
D & B score = 13				• Q1 = 1.00 (referent)	
				• Q2 = 0.64 (0.33-1.25)	
				• Q3 = 0.82 (0.47-1.43)	
			Multivariate logistic regression analysis	• Q4 = 0.67 (0.39-1.16)	
				*p *= 0.23	

Larsson et al 2006 [284]	To investigate the association between PA and colorectal cancer.	• n = 45,906	7.1 year follow-up	Number of cases: 309 (133 proximal, 138 distal)	Results support a role of PA in reducing the risk of CC.
		• Sex: Men			
		• Age: 45-79 yr	PA assessment: Questionnaire for the following variables		
Sweden		• Characteristics: Free of cancer at baseline		Multivariate HR (95% CI) by LTPA	
				• Q1 = 1.00 (referent)	
Prospective cohort				• Q2 = 0.66 (0.43-1.02)	
			LTPA (min/day)	• Q3 = 0.68 (0.46-1.01)	
			Q1 = <10	• Q4 = 0.56 (0.37-0.83)	
D & B score = 14			Q2 = 10-29	*p *= 0.01	
			Q3 = 30-59		
			Q4 = ≥ 60	Multivariate HR (95% CI) by home/housework PA	
			Home/housework PA (h/day)	• Q1 = 1.00 (referent)	
				• Q2 = 0.75 (0.58-0.97)	
			Q1 = none	• Q3 = 0.75 (0.58-0.97)	
			Q2 = <1	• Q4 = 0.68 (0.48-0.96)	
			Q3 = 1-2	*p *= 0.01	
			Q4 = ≥ 3		
			Incidence of Proximal CC(h/day)	Multivariate HR (95% CI) for distal CC by LTPA	
			G1 = <1	• Q1 = 1.00 (referent)	
			G2 = 1-2		
			G3 = ≥ 3	• Q2 = 0.51 (0.28-0.93)	
				• Q3 = 0.50 (0.29-0.87)	
				• Q4 = 0.40 (0.22-0.70)	
				*p *= 0.01	
			Outcome Measure: Incident CC		
				Multivariate HR (95% CI) for proximal CC by home/housework PA	
			Cox proportional HR	• G1 = 1.00 (referent)	
				• G2 = 0.78 (0.53-1.14)	
				• G3 = 0.50 (0.29-0.89)	
				*p *= 0.02	

Lee and Paffenbarger 1994 [285]	To predict cancer risk using prospective assessments of PA.	• n = 17,607	26 year follow-up	• Number of cases: 280	Found a trend, of borderline statistical significance toward decreasing CC risk with increasing PA.
		• Sex: Men		• Number of dropouts: 14%	
		• Age: 30-79 yr	PA assessment: Questionnaire for PA level (kcal/wk)		
USA		• Characteristics: Healthy at baseline		Multivariate RR (95% CI), Model A: PA in 1962/1966 and updated in 1977	
		• Harvard College Alumni		• G1 = 1.00 (referent)	
Prospective cohort			G1 = <1000	• G2 = 1.07 (0.81-1.42)	
			G2 = 1000-2499	• G3 = 1.08 (0.81-1.46)	
			G3 = ≥ 2500	*p *= 0.58	
D & B score = 13					
			Outcome Measure: Incidence of fatal and non fatal CC	Multivariate RR (95% CI), Model B: PA in both 1962/1966 and 1977	
				• G1 = 1.00 (referent)	
				• G2 = 0.75 (0.42-1.35)	
			Cox proportional HR	• G3 = 0.94 (0.54-1.64)	
				p = 0.76	

Lee et al 1997 [286]	To investigate whether PA alters the risk of developing CC in men.	• n = 20,614	10.9 year follow-up	Number of cases: 217	Data does not support the hypothesis that PA is related inversely to risk of developing CC.
		• Sex: Men			
		• Age: 40-84 yrs	PA assessment: Questionnaire for the following variables	Multivariate RR (95% CI), frequency of	
USA		• Characteristics: Physicians, free of cancer at baseline		PA at baseline	
				• G1 = 1.00 (referent)	
Prospective cohort				• G2 = 1.1 (0.7-1.7)	
			Frequency of PA at baseline (times/week)	• G3 = 1.2 (0.8-1.6)	
		Physicians Health Study		• G4 = 1.1 (0.7-1.6)	
D & B score = 15			G1 = <1	*p *= 0.6	
			G2 = 1		
			G3 = 2-4	RR (95% CI), frequency of PA at baseline and 36 months	
			G4 = 5+		
				• G1 = 1.00 (referent)	
			Frequency of PA at baseline and 36 months	• G2 = 1.2 (0.5-2.7)	
			G1 = 1/<1	• G3 = 1.4 (0.9-2.3)	
			G2 = <1/1+	• G4 = 1.3 (0.9-2.0)	
			G3 = 1+/< 1		
			G4 = 1+/1+		
			Outcome Measure: Incidence of fatal and non-fatal CC		
			Cox proportional HR		

Lee et al 2007 [287]	To examine the association between PA and the risk of developing CRC in Japanese men and women.	• n = 65,022	6 year follow-up	Number of cases: 154 proximal CC, 166 distal CC	PA may prevent CC among Japanese men.
		• Sex: Men and women			
Japan		• Age: 40-69 yr		Multivariate RR (95% CI) for CC men	
		• Characteristics	PA assessment: Questionnaire for PA level (median MET hr/d)	• Q1 = 1.00 (referent)	
Prospective cohort		• Ethnicity: Japanese	Q1 = 28.25	• Q2 = 0.87 (0.61-1.26)	
			Q2 = 33.25	• Q3 = 0.62 (0.41-0.95)	
			Q3 = 35.25	• Q4 = 0.58 (0.39-0.87)	
D & B score = 13			Q4 = 43.75	p = 0.006	
			Outcome Measure: Incidence of CC	Multivariate RR (95% CI) for proximal CC men	
			Cox proportional HR	• Q1 = 1.00 (referent)	
				• Q2 = 0.89 (0.52-1.51)	
				• Q3 = 0.44 (0.22-0.86)	
				• Q4 = 0.29 (0.14-0.60)	
				p < 0.001	
				Multivariate RR (95% CI) for distal CC Men	
				• Q1 = 1.00 (referent)	
				• Q2 = 0.92 (0.54-1.54)	
				• Q3 = 0.75 (0.42-1.33)	
				• Q4 = 0.89 (0.53-1.51)	
				p = 0.685	
				PA level and incidence of CC women	
				Total CC	
				• Q1 = 1.00 (referent)	
				• Q2 = 1.03 (0.65-1.64)	
				• Q3 = 0.91 (0.57-1.47)	
				• Q4 = 0.89 (0.54-1.49)	
				*p *= 0.610	
				Proximal CC women	
				• Q1 = 1.00 (referent)	
				• Q2 = 1.14 (0.61-2.12)	
				• Q3 = 1.01 (0.53-1.89)	
				• Q4 = 0.55 (0.24-1.26)	
				*p *= 0.151	
				Distal CC women	
				• Q1 = 1.00 (referent)	
				• Q2 = 1.09 (0.52-2.29)	
				• Q3 = 0.77 (0.34-1.74)	
				• Q4 = 1.37 (0.66-2.85)	
				*p *= 0.401	

Longnecker et al 1995 [288]	To examine the relationship between OPA and vigorous LTPA and the risk of cancer of the right colon and rectum.	• n = 242 rectal cancer and 703 controls	PA assessment: Interview for vigorous LTPA and OPA (coded and self-reported), divided into groups:	Number of cases: 163	The amount of time spent at vigorous LTPA was associated with a decreased risk of cancer of the right colon.
		• Sex: Men		RR (95% CI) by vigorous LTPA	
USA		• Age: ≥ 31 yr		• G1 = 1.00 (referent)	
		• Characteristics: Case: Diagnosed with adenocarcinoma of the right colon or rectum. Controls: Both community and hospital. No history of large bowel cancer.		• G2 = 0.73 (0.23-2.29)	
Case control				• G3 = 0.47 (0.16-1.36)	
			Vigorous LTPA (h/wk)	• G4 = 0.60 (0.35-1.00)	
D & B score = 14			G1 = 0	p = 0.03	
			G2 = ≤ 0.5		
			G3 = 1	Multivariate OR (95% CI) by vigorous	
			G4 = >1	LTPA	
				• G1 = 1.00 (referent)	
			Coded lifetime OPA	• G2 = 0.81 (0.26-2.54)	
			G1 = Sedentary	• G3 = 0.36 (0.11-1.14)	
			G2 = light work	• G4 = 0.57 (0.33-0.97)	
			G3 = moderate	p = 0.06	
			G4 = heavy		
			Self reported lifetime	Multivariate OR (95% CI) by coded lifetime OPA	
			OPA		
			G1 = Sedentary	• G1 = 1.00 (referent)	
			G2 = light work	• G2 = 0.79 (0.39-1.61)	
			G3 = more than light work	• G3 = 0.79 (0.36-1.74)	
				• G4 = 0.99 (0.30-3.22)	
				*p *= 0.42	
			Outcome Measure: Diagnosed with CC		
				Multivariate OR (95% CI) by self reported lifetime OPA	
			Conditional Logistic Regression	• G1 = 1.00 (referent)	
				• G2 = 0.85 (0.41-1.76)	
				• G3 = 0.68 (0.31-1.52)	
				*p *= 0.15	

Mai et al 2007 [289]	To examine in detail the relationship between recreational PA and invasive CC among women.	• n = 120,147	7 year follow-up	Number of cases: 395	Modest inverse association between recreational PA and CC.
		• Sex: Women			
		• Age: 22-84 yr	PA assessment: Questionnaire	RR (95% CI) by MPA over past 3 years	
USA		• Characteristics: no prior history of CC		• G1 = 1.00 (referent)	
				• G2 = 0.95 (0.72-1.24)	
Prospective cohort			MPA over past 3 yrs (h/wk/yr)	• G3 = 0.78 (0.62-0.97)	
		• California Teachers Study		p = 0.02	
			G1 = 0-0.50		
D & B score = 15			G2 = 0.51-1.99	RR (95% CI) by strenuous + moderate (lifetime) PA:	
			G3 = ≥ 2.00	• G1 = 1.00 (referent)	
				• G2 = 0.79 (0.56-1.11)	
			Strenuous + Moderate (lifetime) PA (h/wk/yr)	• G3 = 0.64 (0.44-0.93)	
				p = 0.04	
			G1 = 0.0-0.50		
			G2 = 0.51-3.99		
			G3 = ≥ 4.00		
			Outcome Measure: Incidence of invasive adenocarcinoma of the colon		
			Cox proportional HR		

Martinez et al 1997 [290]	To examine whether LTPA could significantly influence the risk of CC in women.	• n = 89,448	6 year follow-up	Number of cases: 212	Significant inverse association between LTPA and incidence of CC in women.
		• Sex: Women			
		• Age: 30-55 yr	PA assessment: Questionnaire for LTPA	Multivariate RR (95% CI) for all CC	
USA		• Characteristics: Nurses, free from cancer at baseline		• G1 = 1.00 (referent)	
			G1 = <2	• G2 = 0.71 (0.44-1.15)	
Prospective			G2 = 2-4	• G3 = 0.78 (0.50-1.20)	
cohort			G3 = 5-10	• G4 = 0.67 (0.42-1.07)	
			G4 = 11-21	• G5 = 0.54 (0.33-0.90)	
D & B score = 14			G5 = >21	*p *= 0.03	
			Outcome Measure: Incidence of CC	Multivariate RR (95% CI) for distal CC	
				• G1 = 1.00 (referent)	
				• G2 = 0.92 (0.48-1.79)	
			Mantel-Haenszel Estimator and logistic regression models	• G3 = 0.81 (0.43-1.55)	
				• G4 = 0.71 (0.36-1.41)	
				• G5 = 0.31 (0.12-0.77)	
				*p *= 0.01	
				Multivariate RR (95% CI) for proximal	
				CC	
				• G1 = 1.00 (referent)	
				• G2 = 0.54 (0.23-1.22)	
				• G3 = 0.79 (0.40-1.56)	
				• G4 = 0.62 (0.30-1.32)	
				• G5 = 0.77 (0.38-1.58)	
				*p *= 0.67	

Nilsen et al 2008 [291]	To study the separate associations of recreational PA with the incidence of, and mortality from cancer in the ascending, transverse, descending and sigmoid segments of the colon.	• n = 59,369	17 year follow-up	Number of cases: 736	Strong inverse associations between recreational PA and risk of cancer morbidity and mortality of the transverse and sigmoid colon but no association for cancer in the ascending and descending colon.
		• Sex: Men and women	PA assessment: Questionnaire for frequency and duration of recreational PA	HR (95% CI) by frequency of recreational PA, men	
Norway		• Age: not indicated		• G1 = 1.00 (referent)	
Prospective cohort		• Characteristics: Free from cancer at baseline		• G2 = 0.84 (0.60-1.19)	
		• Nord-Trondelag Health Study		• G3 = 0.82 (0.58-1.17)	
				• G4 = 0.81 (0.57-1.15)	
D & B score = 14			Frequency of Recreational PA (times per week)	• G5 = 0.77 (0.54-1.09)	
			G1 = none	*p *= 0.18	
			G2 = <1	HR (95% CI) by frequency of	
			G3 = 1	recreational PA, women	
			G4 = 2-3	• G1 = 1.00 (referent)	
			G5 = ≥ 4	• G2 = 0.91 (0.66-1.25)	
				• G3 = 0.79 (0.57-1.09)	
			Duration of recreational PA (min per exercise)	• G4 = 0.66 (0.47-0.92)	
				• G5 = 0.99 (0.72-1.36)	
			G1 = none	*p *= 0.35	
			G2 = <15		
			G3 = 15-30	HR (95% CI) by duration of recreational	
			G4 = 31-60	PA, men	
			G5 = >60	• G1 = 1.00 (referent)	
				• G2 = 1.07 (0.71-1.60)	
			Intensity of recreational PA	• G3 = 0.80 (0.57-1.12)	
				• G4 = 0.68 (0.48-0.97)	
			G1 = none	• G5 = 0.74 (0.50-1.08)	
			G2 = Low	*p *= 0.02	
			G3 = Moderate/High	HR (95% CI) by duration of recreational PA, women	
			Summary score for recreational PA	• G1 = 1.00 (referent)	
			G1 = None	• G2 = 0.85 (0.59-1.23)	
			G2 = Low	• G3 = 0.81 (0.60-1.09)	
			G3 = High	• G4 = 0.73 (0.53-1.01)	
			By subsite-specific (transverse colon, decending colon, sigmoid colon) CC	• G5 = 0.84 (0.53-1.34)	
				*p *= 0.10	
				HR (95% CI) by intensity of recreational PA, men	
			Levels of REC PA:		
			G1 = None	• G1 = 1.00 (referent)	
			G2 = < 1 x/wk	• G2 = 0.83 (0.62-1.12)	
			G3 = low score	• G3 = 0.74 (0.52-1.06)	
			G4 = high score	*p *= 0.11	
			Outcome Measure: incidence of fatal and non fatal CC	HR (95% CI) by intensity of recreational PA, women	
			Cox proportional HR		
				• G1 = 1.00 (referent)	
				• G2 = 0.77 (0.59-1.01)	
				• G3 = 0.89 (0.60-1.32)	
				*p *= 0.33	
				HR (95% CI) by summary score for recreational PA, men	
				• G1 = 1.00 (referent)	
				• G2 = 0.85 (0.62-1.16)	
				• G3 = 0.69 (0.48-0.98)	
				*p *= 0.06	
				HR (95% CI) by summary score for recreational PA, women	
				• G1 = 1.00 (referent)	
				• G2 = 0.86 (0.64-1.01)	
				• G3 = 0.72 (0.53-0.98)	
				*p *= 0.03	
				HR (95% CI) by total CC and recreational PA, incidence	
				• G1 = 1.00 (referent)	
				• G2 = 0.88 (0.70-1.12)	
				• G3 = 0.87 (0.70-*1.08)	
				• G4 = 0.73 (0.58-0.92)	
				*p *= 0.009	
				HR (95% CI) by subsite specific CC and recreational PA, death	
				• G1 = 1.00 (referent)	
				• G2 = 0.87 (0.64-1.18)	
				• G3 = 0.79 (0.59-1.04)	
				• G4 = 0.56 (0.41-0.78)	
				*p *<0.001	
				HR (95% CI) for transverse CC incidence and recreational PA	
				• G1 = 1.00 (referent)	
				• G2 = 0.75 (0.44-1.28)	
				• G3 = 0.66 (0.41-1.08)	
				• G4 = 0.44 (0.25-0.78)	
				*p *= 0.004	
				HR (95% CI) for transverse CC death and recreational PA	
				• G1 = 1.00 (referent)	
				• G2 = 0.73 (0.36-1.49)	
				• G3 = 0.40 (0.19-0.82)	
				• G4 = 0.33 (0.14-0.76)	
				*p *= 0.002	
				HR (95% CI) for sigmoid CC incidence and recreational PA	
				• G1 = 1.00 (referent)	
				• G2 = 0.88 (0.59-1.32)	
				• G3 = 0.68 (0.46-1.01)	
				• G4 = 0.48 (0.31-0.75)	
				p <0.001	
				HR (95% CI) for sigmoid CC death and recreational PA	
				• G1 = 1.00 (referent)	
				• G2 = 0.78 (0.45-1.35)	
				• G3 = 0.51 (0.30-0.87)	
				• G4 = 0.29 (0.15-0.56)	
				*p *<0.001	

Schnohr et al 2005 [292]	To assess the association between LTPA and incidence of cancer in the general population.	• n = 28,259 (15,043 men,13,216 women)	14 year follow-up	• Number of cases: 215 men, 108 women	For the most active men, VPA was associated with a non-significant lower risk of CC.
Denmark			PA assessment: Questionnaire for LTPA	Multivariate RR (95% CI), men	
		• Sex: Men and women	G1 = Low	• G1 = 1.00 (referent)	
Prospective cohort			G2 = Moderate	• G2 = 1.08 (0.74-1.57)	
		• Age: 20-93 yr	G3 = Vigorous	• G3 = 0.72 (0.47-1.11)	
D & B score = 13		• Characteristics: Free from cancer at baseline	Outcome Measure: Incidence of CC	*p *=0.06	
				Multivariate RR (95% CI), women	
		• Copenhagen Heart Study, The Copenhagen County Centre of Preventive Medicine and the Copenhagen Male Study		• G1 = 1.00 (referent)	
			Cox proportional HR	• G2 = 1.02 (0.70-1.50)	
				• G3 = 0.90 (0.56-1.46)	
				*p *= 0.68	

Slattery et al 1988 [293]	To assess the relationship of PA and diet with the development of CC in Utah.	• n = 229 cases, 384 controls	PA assessment: Interview for the following variables	• Number of cases: 229	PA shows an inverse relationship with incidence of CC.
USA		• Sex: Men and women		Multivariate OR (95% CI) by TPA, men	
Case control		• Age: 40-79 yr		• Q1 = 1.00 (referent)	
		• Characteristics: Case: Diagnosed with CC Controls: no history of cancer	TPA	• Q2 = 1.19 (0.67-2.13)	
			Q1 = Low	• Q3 = 0.88 (0.48-1.69)	
			Q2	• Q4 = .70 (0.38-1.29)	
D & B score = 13			Q3		
			Q4 = high	Multivariate OR (95% CI) by TPA, women	
			Intense PA	• Q1 = 1.00 (referent)	
			G1 = none	• Q2 = 0.97 (0.56-1.69)	
			G2 = low	• Q3 = 0.91 (0.52-1.60)	
			G3 = high	• Q4 = 0.48 (0.27-0.87)	
			Non-intense PA	Multivariate OR (95% CI) by intense PA, men	
			Q1 = Low		
			Q2	• G1 = 1.00 (referent)	
			Q3	• G2 = 0.83 (0.40-1.75)	
			Q4 = high	• G3 = 0.27 (0.11-0.65)	
			Outcome Measure: Diagnosed with CC	Multivariate OR (95% CI) by intense PA, women	
			Multiple logistic regression analysis	• G1 = 1.00 (referent)	
				• G2 = 0.55 (0.23-1.34)	
				Multivariate OR (95% CI) by non-intense PA, men	
				• Q1 = 1.00 (referent)	
				• Q2 = 1.40 (0.76-2.57)	
				• Q3 = 0.93 (0.51-1.72)	
				• Q4 = 1.25 (0.68-2.29)	
				Multivariate OR (95% CI) by non-intense PA, women	
				• Q1 = 1.00 (referent)	
				• Q2 = 1.09 (0.62-1.90)	
				• Q3 = 0.94 (0.53-1.66)	
				• Q4 = 0.53 (0.29-0.95)	

Slattery et al 1997 [294]	To examine the relationship between weekly PA patterns (source, duration and frequency) and CC.	• n = 1,993 cases, 2,410 controls	PA Assessment: Interview, adapted CARDIA PA history	Number of cases: 1,993	High level of leisure time VPA during the past 20 yrs was associated with a reduced risk of CC in both men and women. The same associations were not observed with leisure time MPA.
USA		• Sex: Men and women		Multivariate OR (95% CI) by recent leisure time VPA, men	
Case control		• Age: 30-79 yr	Recent leisure time	• Q1 = 1.00 (referent)	
		• Characteristics: Cases: diagnosed with first primary CC. Controls: no history of CC	VPA	• Q2 = 0.80 (0.64-1.01)	
			Q1 = None	• Q3 = 0.84 (0.66-1.05)	
D & B score = 14			Q2	• Q4 = 0.69 (0.55-0.87)	
			Q3		
			Q4 = High	Multivariate OR (95% CI) by recent leisure time VPA, women	The greatest inverse association was observed when activities were performed for longer periods of time per session.
		The Three Centered Diet, Activity and Lifestyle Colon Cancer Study	Leisure time VPA	• Q1 = 1.00 (referent)	
			Q1 = Low	• Q2 = 0.79 (0.61-1.02)	
			Q2	• Q3 = 0.83 (0.64-1.07)	
			Q3	• Q4 = 0.86 (0.67-1.10)	
			Q4 = High		
			Current PA (min)	Multivariate OR (95% CI) by leisure time VPA, men	
			G1 = <30		
			G2 = 30-60	• Q1 = 1.00 (referent)	
			G3 = ≥ 60	• Q2 = 0.97 (0.76-1.25)	
				• Q3 = 0.86 (0.67-1.09)	
			LTPA (ranked by time per session)	• Q4 = 0.61 (0.47-0.79)	
			Q1 = None	Multivariate OR (95% CI) by leisure time VPA, women	
			Q2 = Low - <30 min		
			Q3 = moderate - 30-60 min	• Q1 = 1.00 (referent)	
			Q4 = high ->60 min	• Q2 = 0.75 (0.59-0.95)	
				• Q3 = 0.68 (0.53-0.87)	
			Number of activity session per week	• Q4 = 0.63 (0.48-0.83)	
			G1 = None	Multivariate OR (95% CI) by current MPA time per week	
			G2 = 1		
			G3 = 2-4	• Q1 = 1.00 (referent)	
			G4 = 5-7	• Q2 = 1.00 (0.83-1.21)	
			G5 = >7	• Q3 = 0.90 (0.76-1.07)	
				• Q4 = 0.92 (0.77-1.10)	
			Outcome Measure: Diagnosed with CC	Multivariate OR (95% CI) by current VPA time per week	
			Unconditional regression models	• Q1 = 1.00 (referent)	
				• Q2 = 0.90 (0.73-1.12)	
				• Q3 = 0.89 (0.71-1.10)	
				• Q4 = 0.83 (0.69-0.98)	
				Multivariate OR (95% CI) by leisure time MPA time per session	
				• Q1 = 1.00 (referent)	
				• Q2 = 1.20 (0.91-1.59)	
				• Q3 = 1.09 (0.83-1.42)	
				• Q4 = 1.08 (0.82-1.42)	
				Multivariate OR (95% CI) by leisure time VPA time per session	
				• Q1 = 1.00 (referent)	
				• Q2 = 0.86 (0.74-0.99)	
				• Q3 = 0.76 (0.64-0.90)	
				• Q4 = 0.68 (0.52-0.87)	
				Multivariate OR (95% CI) by number of MPA sessions/wk	
				• G1 = 1.00 (referent)	
				• G2 = 1.02 (0.79-1.30)	
				• G3 = 0.86 (0.72-1.02)	
				• G4 = 0.91 (0.81-1.14)	
				• G5 = 1.02 (0.82-1.27)	
				Multivariate OR (95% CI) by number of VPA sessions/wk	
				• G1 = 1.00 (referent)	
				• G2 = 0.72 (0.56-0.92)	
				• G3 = 0.87 (0.73-1.03)	
				• G4 = 1.00 (0.81-1.25)	
				• G5 = 0.84 (0.61-1.15)	

Slattery et al 1997 [295]	To determine how physical inactivity interacts with other components of energy balance in determining risk of CC.	• n = 1,993 cases, 2,410 controls	PA Assessment: Interview for lifetime VPA (PA index)	Number of cases: 1,993	These results support previous findings that physical inactiity is associated with an increased risk of developing CC.
USA		• Sex: Men and women	Q1 = 10-12	Multivariate OR (95% CI), men	
		• Age: 30-79 yr	Q2 = 7-9	• Q1 = 1.00 (referent)	
		• Characteristics: Cases: diagnosed with first primary CC. Controls: no history of CC	Q3 = 4-6	• Q2 = 1.60 (1.11-1.75)	
Case control		• The Three Centered Diet, Activity and Lifestyle Colon Cancer Study	Q4 = <4	• Q3 = 1.59 (1.26-2.01)	
D & B score = 14				• Q4 = 1.63 (1.26-2.12)	
			Outcome Measure: Diagnosed with CC	Multivariate OR (95% CI), women	
				• Q1 = 1.00	
				• Q2 = 1.14 (0.86-1.52)	
			Unconditional regression models	• Q3 = 1.13 (0.85-1.49	
				• Q4 = 1.59 (1.21-2.10)	

Takahashi et al 2007 [296]	To investigate the association between time spent walking each day and the risk of CRC.	• n = 20,519 men, 21,469 women	7 year follow-up	• Number of cases: 101	Time spent walking per day was associated with a lower risk of colon cancer in men but not in women.
		• Sex: Men and women	PA assessment: Questionnaire for time spent walking (h/day)	• Number of dropouts: 3.5%	
Japan		• Age: 40-64 yr		Multivariate RR (95% CI), men	
		• Characteristics: Free from cancer at baseline		• G1 = 1.00 (referent)	
Prospective cohort			G1 = <0.5	• G2 = 0.72 (0.43-1.21)	
			G2 = 0.5-1	• G3 = 0.38 (0.22-0.64)	
			G3 = >1	*p *< 0.001	
D & B score = 12			Outcome Measure: Incidence of CC	Time spent walking and incidence of CC	
				Multivariate RR (95% CI), women	
			Cox proportional HR	• G1 = 1.00	
				• G2 = 2.68 (0.94-7.68)	
				• G3 = 1.79 (0.64-4.96)	
				*p *= 0.42	

Tang et al 1999 [297]	To investigate the association between PA, water intake and risk of CRC in a hospital based case controlled study.	• n = 163 cases, 163 controls	PA assessment: Interview	• Number of cases: 163	Found a negative association between LTPA and the risk of CC among men.
		• Sex: Men and women		Multivariate RR (95% CI), men	
Taiwan			LTPA METs	• G1 = 1.00 (referent)	
		• Age: 33-80 yr	G1 = Sedentary	• G2 = 2.22 (0.68-7.21)	
Case control		• Characteristics: Cases: Hospital patients diagnosed with colorectal cancer Controls: Hospital patients in hospital for other reasons, free of CRC.	G2 = Moderate (< 20 MET)	• G3 = 0.19 (0.05-0.77)	
D & B score = 14			G3 = Active (≥20 MET)	*p *= 0.03	
				Multivariate RR (95% CI), women	
			Outcome Measure: Diagnosis of CC	• G1 = 1.00 (referent)	
				• G2 = 0.52 (0.13-2.03)	
				• G3 = 0.63 (0.18-2.18)	
			Conditional logistic regression analysis	*p *= 0.48	

Tavani et al 1999 [298]	To investigate the relationship between PA and risk of CC in both sexes at different ages.	• n = 5,379 (1,225 cases and 4,154 controls)	PA assessment: Questionnaire on activity at work and during leisure time	• Number of cases: 537 women, 688 men	The study confirms that OPA is protective against CC.
Italy		• Sex: Men and women		Multivariate OR (95% CI) for CC by OPA at age 15-19 yr, men	
Case control		• Age: 19-74 yr	G1 = Highest	• G1 = 1.00 (referent)	
			G2	• G2 = 0.89 (0.64-1.23)	
D & B score = 13			G3	• G3 = 0.72 (0.54-0.97)	
			G4	• G4 = 0.54 (0.40-0.74)	
			G5 = Lowest	• G5 = 0.47 (0.31-0.71)	
				*p *< 0.01	
			OPA at 30-39 yrs old		
			Q1 = Highest	Multivariate OR (95% CI) for CC by OPA at age 15-19 yr, women	
			Q2	• G1 = 1.00 (referent)	
			Q3	• G2 = 0.73 (0.55-0.96)	
			Q4 = Lowest	• G3 = 0.91 (0.69-1.21)	
			Outcome Measure: Diagnosis of CC	• G4 = 0.62 (0.44-0.89)	
			Unconditional multiple Logistic Regression	*p *< 0.05	
				Multivariate OR (95% CI) for CC by OPA at age 30-39 yr, men	
				• G1 = 1.00 (referent)	
				• G2 = 1.01 (0.75-1.37)	
				• G3 = 0.79 (0.59-1.06)	
				• G4 = 0.71 (0.52-0.97)	
				• G5 = 0.64 (0.44-0.93)	
				p < 0.01	
				Multivariate OR (95% CI) for CC by OPA at age 30-39 yr, women	
				• G1 = 1.00 (referent)	
				• G2 = 0.65 (0.46-0.93)	
				• G3 = 0.57 (0.41-0.79)	
				• G4 = 0.49 (0.33-0.72)	
				*p *< 0.01	
				Multivariate OR (95% CI) for CC by OPA at age 50-59 yr, men	
				• G1 = 1.00 (referent)	
				• G2 = 1.06 (0.78-1.43)	
				• G3 = 0.85 (0.63-1.14)	
				• G4 = 0.68 (0.49-0.95)	
				• G5 = 0.69 (0.45-1.05)	
				*p *< 0.01	
				Multivariate OR (95% CI) for CC by OPA at age 50-59 yr, women	
				• G1 = 1.00 (referent)	
				• G2 = 0.69 (0.47-1.00)	
				• G3 = 0.68 (0.46-1.00)	
				• G4 = 0.75 (0.47-1.20)	
				*p *= > 0.05	
				Multivariate OR (95% CI) for ascending CC by OPA at age 30-39 yr No significant associations for men or women	
				Multivariate OR (95% CI) for transverse and descending CC by OPA at age 30-39 yr, men	
				• Q1 = 1.00 (referent)	
				• Q2 = 0.92 (0.51-1.67)	
				• Q3 = 0.76 (0.43-1.37)	
				• Q4 = 0.46 (0.24-0.87)	
				*p *< 0.05	
				Multivariate OR (95% CI) for transverse and descending CC by OPA at age 30-39 yr, women	
				• Q1 = 1.00 (referent)	
				• Q2 = 0.51 (0.23-1.10)	
				• Q3 = 0.39 (0.19-0.80)	
				• Q4 = 0.29 (0.12-0.71)	
				*p *< 0.01	
				Multivariate OR (95% CI) for sigmoid CC by OPA at age 30-39 yr, men	
				• Q1 = 1.00 (referent)	
				• Q2 = 1.02 (0.65-1.57)	
				• Q3 = 0.78 (0.51-1.20)	
				• Q4 = 0.54 (0.34-0.85)	
				*p *< 0.01	
				Multivariate OR (95% CI) for sigmoid CC by OPA at age 30-39 yr, women	
				• Q1 = 1.00 (referent)	
				• Q2 = 0.62 (0.36-1.05)	
				• Q3 = 0.71 (0.44-1.15)	
				• Q4 = 0.58 (0.32-1.03)	
				*p *> 0.05	

Thune et al 1996 [299]	To examine the association between self-reported OPA and LTPA and the subsequent risk of CC.	• n = 81,516 (53,242 men, 28,274 women)	16.3 year follow up	Number of cases: 236 men, 99 women	An inverse dose-response relationship between TPA and risk of CC was observed in women. In men this inverse dose-response was found only for those 45 yrs or older at study entry.
			PA assessment: Questionnaire for TPA (OPA plus recreational PA (combined)	Multivariate RR (95% CI) for total CC, men	
Norway		• Sex: Men and women	G1 = Sedentary	• G1 = 1.00 (referent)	
Prospective cohort		• Age: 20-49 yr	G2 = Moderate	• G2 = 1.18 (0.76-1.82)	
		• Characteristics: Free from cancer at baseline	G3 = Active	• G3 = 0.97 (0.63-1.50)	
D & B score = 14				*p *= 0.49	
				Multivariate RR (95% CI) for total CC, women	
			Outcome Measure: Incidence of CC	• G1 = 1.00 (referent)	
			Cox proportional HR	• G2 = 0.97 (0.33-2.77)	
				• G3 = 0.63 (0.39-1.04)	
				*p *= 0.04	
				Multivariate RR (95% CI) for proximal CC, men	
				• G1 = 1.00 (referent)	
				• G2 = 1.16 (0.57-2.34)	
				• G3 = 0.96 (0.47-1.93)	
				*p *= 0.64	
				Multivariate RR (95% CI) for proximal CC, women	
				• G1 = 1.00 (referent)	
				• G2 = 1.22 (0.51-2.94)	
				• G3 = 0.62 (0.30-1.28)	
				*p *= 0.10	
				Multivariate RR (95% CI) for distal CC, men	
				• G1 = 1.00 (referent)	
				• G2 = 1.29 (0.72-2.33)	
				• G3 = 0.99 (0.55-1.80)	
				*p *= 0.53	
				Multivariate RR (95% CI) for distal CC, women	
				• G1 = 1.00 (referent)	
				• G2 = 0.84 (0.32-2.17)	
				• G3 = 0.61 (0.30-1.23)	
				*p *= 0.15	
				Multivariate RR (95% CI) for total CC, men < 45 yrs at entry	
				• G1 = 1.00 (referent)	
				• G2 = 2.02 (0.78-5.21)	
				• G3 = 2.23 (0.88-5.66)	
				*p *= 0.13	
				Multivariate RR (95% CI) for total CC, women < 45 yrs at entry	
				• G1 = 1.00 (referent)	
				• G2 = 0.96 (0.39-2.40)	
				• G3 = 0.62 (0.31-1.23)	
				*p *= 0.13	
				Multivariate RR (95% CI) for total CC, men ≥ 45 yrs at entry	
				• G1 = 1.00 (referent)	
				• G2 = 0.96 (0.59-1.58)	
				• G3 = 0.66 (0.40-1.10)	
				*p *= 0.04	
				Multivariate RR (95% CI) for total CC, women ≥ 45 yrs at entry	
				• G1 = 1.00 (referent)	
				• G2 = 0.99 (0.41-2.39)	
				• G3 = 0.66 (0.33-1.33)	
				*p *= 0.19	

Vena et al 1985 [300]	To assess the relationship between lifetime OPA and the risk of CC.	• n = 1,641 (210 cases, 1,431 control)	PA assessment: Questionnaire	• Number of cases: 210	CC risk increased with increasing amount and proportion of time in jobs involving only sedentary or light work.
USA		• Sex: Men	Number of work years in jobs with sedentary or light work (yr)	OR (95% CI) by number of work years in jobs with sedentary or light work	
Case control		• Age: 30-79 yr	G1 = None	• G1 = 1.00 (referent)	
		• Characteristics: Cases: admitted to hospital. Diagnosis of CC Controls: Admitted to hospital. Diagnosed with non-neoplastic non-digestive diseases	G2 = 1-20	• G2 = 1.49	
D & B score = 15			G3 = >20	• G3 = 1.97	
				OR (95% CI) by proportion of years in jobs with sedentary or light work	
					
			Proportion of years in jobs with sedentary or light work	• G1 = 1.00 (referent)	
				• G2 = 1.53	
				• G3 = 1.58	
			G1 = None	• G4 = 2.10	
			G2 = 0.01-0.50		
			G3 = 0.41-0.99	OR (95% CI) by proportion of life in jobs with sedentary or light work	
			G4 = 1.00 (referent)		
				• G1 = 1.00 (referent)	
			Proportion of life in jobs with sedentary or light work	• G2 = 1.66	
				• G3 = 1.83	
			G1 = None		
			G2 = 0.01-0.40		
			G3 = 0.41-1.00		
			Outcome Measure: diagnosed with CC		
			Multiple logistic regression		

Vetter et al 1992 [301]	To investigate the influence of OPA on the risk of CC in a developing country.	• n = 87 men cases, 13 women cases, 371 controls	PA assessment: Questionnaire Job title and industry names	Number of cases: 87 men, 13 women	This study presents a weak inverse association between CC and PA.
USA		• Sex: Men and women		OR (95% CI) Sitting time and CC	Only 2 of the 4 measures of activity showed evidence of an increased CC risk for sedentary jobs (time spent sitting and occupational EE) though neither was statistically significant.
Case control		• Age: 14-97 yr	Levels (Sitting time, energy expenditure	• G1 = 1.00 (referent)	
D & B score = 11		• Characteristics: Cases: Diagnosed with CC Controls: cancer cases other then colon, rectum and lung cancer.	G1 = High	• G2 = 1.0 (0.5-2.0)	
			G2 = Moderate	• G3 = 1.5 (0.7-2.9)	
			G3 = Sedentary	*p *= 0.145	
			Outcome Measure: Diagnosed with CC	OR (95% CI) Energy Expenditure and CC	
				• G1 = 1.00 (referent)	
				• G2 = 1.5 (0.7-3.3)	
				• G3 = 1.6 (0.8-3.6)	
				*p *= 0.143	

White et al 1996 [302]	To assess the relationship between PA and CC among men and women.	• n = 871 (251 men, 193 women cases. 233 men & 194 women controls)	PA assessment: Phone interview	• Number of cases: 251 men & 193 women	The results of this study show modest support that recreational PA is associated with a reduced risk of CC.
USA			Total PA (episodes/wk)	RR (95% CI) by total PA, men	
			G1 = 0	• G1 = 1.00 (referent)	
Case control		• Sex: Men and women	G2 = <1	• G2 = 0.81 (0.45-1.44)	
			G3 = 1-<2	• G3 = 0.53 (0.30-0.94)	
D & B score = 14		• Age: 30-62 yr	G4 = 2-< 4	• G4 = 0.57 (0.33-1.00)	
		• Characteristics: Cases: Diagnosed with CC, no previous history or CC or inflammatory bowel	G5 = ≥ 4	• G5 = 0.57 (0.40-1.11)	
			Moderate-high intensity PA (epsiodes/wk)	*p *= 0.03	
				RR (95% CI) by total PA, women	
			G1 = 0	• G1 = 1.00 (referent)	
			G2 = <1	• G2 = 1.17 (0.57-2.40)	
			G3 = 1-<2	• G3 = 1.27 (0.65-2.45)	
		Controls: No history of CC or inflammatory bowel	G4 = ≥ 2	• G4 = 0.59 (0.34-1.04)	
				• G5 = 1.09 (0.61-1.97)	
			High intensity PA (episodes/wk)	*p *= 0.52	
			G1 = 0	RR (95% CI) by total PA, men and women	
			G2 = <1	• G1 = 1.00 (referent)	
			G3 = ≥ 1	• G2 = 0.94 (0.60-1.47)	
			METS/wk	• G3 = 0.77 (0.50-1.19)	
			Q1 = 0	• G4 = 0.57 (0.39-0.85)	
			Q2 = <7.30		
			Q3 = 7.30-17.88	• G5 = 0.83 (0.57-1.22)	
			Q4 = ≥ 17.88	*p *= 0.04	
			Outcome Measure: Diagnosed with CC	RR (95% CI) by moderate-high intensity PA, men	
					
				• G1 = 1.00 (referent)	
			Unconditional logistic regression	• G2 = 0.84 (0.49-1.43)	
				• Q3 = 0.75 (0.42-1.36)	
				• Q4 = 0.66 (0.41-1.05)	
				*p *= 0.07	
				RR (95% CI) by moderate-high intensity PA, women	
				• G1 = 1.00 (referent)	
				• G2 = 1.07 (0.58-1.97)	
				• G3 = 1.00 (0.51-1.98)	
				• G4 = 0.74 (0.42-1.29)	
				*p *= 0.37	
				RR (95% CI) by moderate-high intensity PA, men and women	
				• Q1 = 1.00 (referent)	
				• Q2 = 0.93 (0.62-1.39)	
				• Q3 = 0.86 (0.55-1.34)	
				• Q4 = 0.70 (0.49-1.00)	
				*p *= 0.05	
				RR (95% CI) by high intensity PA, men	
				• G1 = 1.00 (referent)	
				• G2 = 0.85 (0.48-1.52)	
				• G3 = 0.57 (0.35-0.92)	
				*p *= 0.02	
				RR (95% CI) by high intensity PA, Women	
				• G1 = 1.00 (referent)	
				• G2 = 1.02 (0.51-2.04)	
				• G3 = 0.74 (0.43-1.28)	
				*p *= 0.31	
				RR (95% CI) by high intensity PA, men and women	
				• G1 = 1.00 (referent)	
				• G2 = 0.93 (0.59-1.44)	
				• G3 = 0.64 (0.45-0.92)	
				*p *= 0.02	
				RR (95% CI) by METs/wk, men	
				• Q1 = 1.00 (referent)	
				• Q2 = 0.64 (0.38-1.07)	
				• Q3 = 0.59 (0.37-0.96)	
				• Q4 = 0.69 (0.42-1.13)	
				p = 0.05	
				RR (95% CI) by METs/wk, women	
				• Q1 = 1.00 (referent)	
				• Q2 = 0.87 (0.51-1.49)	
				• Q3 = 1.20 (0.69-2.08)	
				• Q4 = 0.74 (0.41-1.34)	
				*p *= 0.62	
				RR (95% CI) by METs/wk, women	
				• Q1 = 1.00 (referent)	
				• Q2 = 0.73 (0.50-1.06)	
				• Q3 = 0.80 (0.56-1.16)	
				• Q4 = 0.73 (0.50-1.06)	
				*p *= 0.08	

Wolin et al 2007 [303]	To assess the relationship between PA and risk of CC in women.	• n = 79,295	16 year follow-up	Number of cases: 547 (245 distal, 302 proximal) Number of dropouts: 10%	A significant inverse association exists between PA, including moderate intensity, such as walking, and risk of CC in women that is more pronounced for distal tumours.
		• Sex: Women			
		• Age: 40-65 yr	PA assessment: Questionnaire		
USA		• Characteristics: Nurses, no history of CC, ulcerative colitis and Crohn's disease			
Prospective cohort			Level of PA	Multivariate RR (95% CI) for distal CC by level of PA	
			G1 = <2	• G1 = 1.00 (referent)	
			G2 = 2.1-4.5	• G2 = 0.93 (0.64-1.36)	
D & B score = 14			G3 = 4.6-10.3	• G3 = 0.99 (0.68-1.44)	
		Nurses' Health Study	G4 = 10.4 - 21.4	• G4 = 0.87 (0.59-1.29	
			G5 = ≥ 21.5	• G5 = 0.54 (0.34-0.84)	
			MPA or VPA (hr/wk)	*p *= 0.004	
			G1 = 0	Multivariate RR (95% CI) for proximal CC by level of PA not significant p = 0.77	
			G2 = <1		
			G3 = 1-1.9		
			G4 = 2-3.9		
			G5 = ≥ 4	Multivariate RR (95% CI) for all CC by MPA or VPA	
			Outcome Measure: Fatal and non fatal CC	• G1 = 1.00 (referent)	
				• G2 = 0.85, (0.64-1.14)	
			Cox proportional HR	• G3 = 0.74 (0.53-1.04)	
				• G4 = 0.56 (0.33-0.94)	
				*p *= 0.01	
				Multivariate RR (95% CI) for distal CC by MPA or VPA	
				• G1 = 1.00 (referent)	
				• G2 = 1.10 (0.73-1.66)	
				• G3 = 0.63 (0.36-1.10)	
				• G4 = 0.51 (0.22-1.17)	
				*p *= 0.04	
				Multivariate RR (95% CI) for proximal CC by MPA or VPA not significant p = 0.12	

Zhang et al 2006 [304]	To examine the relationship between LTPA and OPA and the risk of CC by anatomic site and to evaluate their joint effect on the risk of CC.	• n = 585 cases 2,172 controls	PA assessment: Questionnaire for the following variables	Number of cases: 585	Found a significant inverse association between reported LTPA and risk of CC with a slightly stronger association for the right colon than the left in both men and women.
USA		• Sex: Men and women		Multivariate OR (95% CI) by moderate- strenuous LTPA, men and women	
		• Age: 40-85 yr	Moderate-Strenuous LTPA	• G1 = 1.00 (referent)	
Case control		• Characteristics: Case: diagnosed with CC Control: no history of CC.	G1 = <1 month	• G2 = 0.7 (0.5-1.1)	
			G2 = 1-4 months	• G3 = 0.6 (0.4-0.8)	
D & B score = 15			G3 = ≥ 2 weeks	*p *= 0.003	
				Multivariate OR (95% CI) by moderate- strenuous LTPA, men	The joint effect of OPA and LTPA suggested that the risk was lowest for those with high OPA and non-OPA.
				• G1 = 1.00 (referent)	
			Outcome Measure: CC	• G2 = 0.9 (0.5-1.7)	
				• G3 = 0.5 (0.3-0.9)	
			Unconditional logistic regression models	*p *= 0.02	
				Multivariate OR (95% CI) by moderate-strenuous LTPA, women	
				• G1 = 1.00 (referent)	
				• G3 = 0.5 (0.3-1.0)	
				• G3 = 0.6 (0.4-0.9)	
				*p *= 0.02	
				Multivariate OR (95% CI) by moderate-strenuous LTPA, men and women	
				• G1 = 1.00 (referent)	
				• G2 = 0.7 (0.5-1.1)	
				• G3 = 0.8 (0.6-1.1)	
				*p *= 0.53	
				Multivariate OR (95% CI) by moderate-strenuous LTPA, men	
				• G1 = 1.00 (referent)	
				• G2 = 0.9 (0.5-1.5)	
				• G3 = 0.8 (0.6-1.2)	
				*p *= 0.55	
				Multivariate OR (95% CI) by moderate- strenuous LTPA, women	
				• G1 = 1.00 (referent)	
				• G2 = 0.6 (0.3-1.1)	
				• G3 = 0.8 (0.5-1.2)	
				*p *= 0.62	
				Multivariate OR (95% CI) by moderate- strenuous LTPA and OPA, OPA-Low	
				• G1 = 1.00 (referent)	
				• G2 = 0.5 (0.3-0.9)	
				• G3 = 0.8 (0.5-1.2)	
				*p *= 0.41	
				Multivariate OR (95% CI) by moderate-strenuous LTPA and OPA, OPA-Medium	
				• G1 = 0.7 (0.5-1.1)	
				• G2 = 0.7 (0.4-1.3)	
				• G3 = 0.5 (0.3-0.8)	
				*p *= 0.04	
				Multivariate OR (95% CI) by moderate-strenuous LTPA and OPA, OPA-High	
				• G1 = 0.9 (0.5-1.6)	
				• G2 = 0.6 (0.3-1.3)	
				• G3 = 0.5 (0.3-0.8)	

D & B score, Downs and Black quality score; YR, years; PA, physical activity; OPA, occupational physical activity; kJ/min, kilojoules per minute; G, groups; MET, metabolic equivalent; HR, hazard ratio; RR, risk ratio; OR, odds ratio; 95% CI, confidence interval; LTPA, leisure-time physical activity; CC, colon cancer; TPA, total physical activity; MDA, moderate physical activity; h/d, hours per day; VPA, vigorous physical activity; h/wk, hours per week.

#### Breast Cancer

As reviewed eloquently by others, the epidemiological evidence relating physical activity to a decreased incidence of breast cancer is persuasive. A recent systematic review of the literature found that more than 60 observational trials have examined the relationship between physical activity and breast cancer [31]. Previous reviews of the literature have revealed compelling and consistent findings indicating that habitual physical activity is associated with a reduced risk for breast cancer ranging from 20-80% [31,104].

Various investigations have attempted to evaluate the dose-response relationship between physical activity and the incidence of breast cancer (Table 16). Despite the volume of evidence available questions still remain regarding the minimal and optimal volume of exercise required to reduce the risk for breast cancer. As discussed by others [31,104] the findings are as varied as the investigations.

**Table 16:** Studies examining the relationship between physical activity and breast cancer.

Publication Country Study Design Quality Score	Objective	Population	Methods	Outcome	Comments and Conclusions
Rockhill et al 1999 [106]	To examine the effect of PA on the risk for BC.	• n = 121,701	PA assessment: Self-reported LTPA, grouped into hr/wk	3,137 cases of BC	Women who engaged in 7 or more hours per week of MVPA had a 20% lower risk of BC. An inverse dose-response relationship existed between PA and BC incidence.
		• Sex: Women			
		• Age: 30-55 yr		RR (95% CI) for BC and LTPA	
USA		• 16-yr follow-up	G1 = <1		
		• Characteristics: Free of BC	G2 = 1.0-1.9	• G1 = 1.00 (referent)	
Prospective cohort			G3 = 2.0-3.9	• G2 = 0.88 (0.79-0.98)	
		• The nurses Health Study	G4 = 4.0-6.9	• G3 = 0.89 (0.81-0.99)	
D & B score = 13			G5 = ≥7	• G4 = 0.85 (0.77-0.94)	
			Multivariate pooled logistic regression	• G5 = 0.82 (0.70-0.97)	
				Trend *p *= 0.004	

Sesso et al 1998 [107]	To examine the association between PA and BC among postmenopausal women.	• n = 1,566	31-yr follow-up	109 cases of BC	There is an inverse relationship between PA and BC in post-menopausal women.
		• Sex: Women			
USA		• Age: 45.5	PA assessment: Questionnaire at baseline, divided into tertiles (kcal/wk)	RR (95% CI) for BC and PA	
		• Characteristics: Free of BC			
Prospective cohort				• T1 = 1.00 (referent)	
			T1 = <500	• T2 = 0.92 (0.58-1.45)	
D & B score = 14			T2 = 500-999		
			T3 = ≥ 1,000	• T3 = 0.73 (0.46-1.14)	
				RR (95% CI), post-menopausal women only	
				• T1 = 1.00 (referent)	
				• T2 = 0.95 (0.58-1.57)	
				• T3 = 0.49 (0.28-0.86)	

Dosemeci et al 1993 [278]	To conduct a multiple-site case-control study of 15 cancers to examine associations between PA, SES, and these cancer sites among workers.	• n = 2,643 control group	Cases: obtained from an oncological treatment center from 1979-1984	31 men had BC and 241 women had BC	This study shows the sitting-time index showed an elevated risk of female BC for sedentary jobs without SES adjustment.
		• n = 2,127 men and n = 244 women)			
		• Sex: Men and women			
Turkey		• Characteristics: Cases - diagnosed with one of the 15 cancers being examined. Control Group - subjects diagnosed with non-cancers, cancers of the buccal cavity, esophagus, liver, bone, soft tissue, brain, lymphoma and other cancer sites for which there is no suggestion on an association with PA		Adjusted SES OR (95%CI), men	
			Controls: pulled from the same hospital as the cases		
Case control				• G1 = 1.40 (0.60-3.90)	
D & B score = 12			PA assessment: OPA (kJ/min)	• G2 = 1.10 (0.40-3.10)	
			G1 = <8	• G3 = 1.00 (referent)	The slightly elevated risk of male BC was based on a small number and disappeared when the risk was adjusted for SES.
			G2 = 8-12	Trend *p *= 0.34	
			G3 = >12		
				Adjusted SES OR (95%CI), women	
			Gart's method and Mantel's chi-square test		
				• G1 = 1.10 (0.60-2.10)	
				• G2 = 0.90 (0.50-1.80)	
				• G3 = 1.00 (referent)	
				Trend *p *= 0.23	

Bernstein et al 1994 [305]	To determine whether young women who regularly participate in PA during their reproductive years had a reduced risk of BC.	• n = 1,090 (545 cases; 545 controls)	PA assessment: Questionnaire for overall participation in PA after menarche (h/wk), PA within 10 years after menarche (h/wk), each divided into 5 groups:	Adjusted OR (95% CI) by PA after menarche	PA may substantially reduce a women's lifetime risk of BC.
		• Sex: Women		• G1 = 1.00 (referent)	
USA		• Age: ≤ 40 yr		• G2 = 0.95 (0.64-1.41)	
		• Characteristics: White women matched for age and parity			
Case control				• G3 = 0.65 (0.45-0.96)	
D & B score = 15			G1 = none	• G4 = 0.80 (0.54-1.17)	
			G2 = 0.1-0.7		
			G3 = 0.8-1.6	• G5 = 0.42 (0.27-0.64)	
			G4 = 1.7-3.7		
			G5 = ≥ 3.8	Trend *p *= 0.0001	
			Logistic regression	Adjusted OR (95% CI) by PA within 10 years after menarche	
				• None = 1.00 (referent)	
				• 0.1-1.2 = 0.93 (0.63-1.38)	
				• 1.3-2.9 = 0.78 (0.52-1.19)	
				• 3.0-5.5 = 0.69 (0.45-1.05)	
				• ≥5.6 = 0.70 (0.47-1.06)	
				Trend *p *= 0.027	
				Adjusted OR (95% CI) by PA after menarche, nulliparous women	
				• G1 = 1.00 (referent)	
				• G2 = 0.81 (0.42-1.57)	
				• G3 = 0.65 (0.35-1.21)	
				• G4 = 0.94 (0.53-1.67)	
				• G5 = 0.73 (0.38-1.41)	
				Trend *p *= 0.43	
				Adjusted OR (95% CI) by PA after menarche, parous women	
				• G1 = 1.00 (referent)	
				• G2 = 1.06 (0.65-1.74)	
				• G3 = 0.65 (0.40-1.06)	
				• G4 = 0.70 (0.42-1.18)	
				• G5 = 0.38 (0.16-0.50)	
				Trend *p *< 0.0001	

Bernstein et al 2005 [306]	To examine the relationship between BC risk and lifetime and time- or age-specific measures of LTPA among white and black women.	• n = 9,187 (4,538 cases; 4,649 control)	Cases: histologically confirmed cases of BC	4,538 cases of BC	This study supports an inverse association between PA and BC among black women and among white women.
		• Sex: Women			
		• Age: 35-64		Multivariate adjusted OR (95%CI) annual MET h/wk, White participants	
USA		• Ethnicity: White (including Hispanics) or Black	Controls: random-digit dialing methods		
		• Characteristics: Case Group: histologically confirmed cases of invasive BC			
Case control		• Control Group: healthy		• G1 = 1.00 (referent)	
			PA assessment:	• G2 = 0.84 (0.71-0.99)	
D & B score = 13			Questionnaire for lifetime PA (MET h/wk), divided into 5 groups	• G3 = 0.89 (0.75-1.04)	
			G1 = Inactive	• G4 = 0.82 (0.69-0.97)	The relationship appears to be similar between black and white women.
			G2 = ≤ 2.2	• G5 = 0.81 (0.69-0.96)	
			G3 = 2.3-6.6	Trend *p *= 0.09	
			G4 = 6.7-15.1		
			G5 = ≥ 15.2		
			Unconditional logistic regression modeling	Multivariate adjusted OR (95%CI) annual MET h/wk, Black participants	
				• G1 = 1.00 (referent)	
				• G2 = 1.11 (0.91-1.35)	
				• G3 = 0.83 (0.67-1.03)	
				• G4 = 0.79 (0.63-0.99)	
				• G5 = 0.77 (0.62-0.95)	
				Trend *p *= 0.003	

Carpenter et al. 1999 [307]	To examine whether lifetime exercise activity is related to BC risk in post-menopausal women.	• n = 2,027 (1,123 case; 904 control)	Cases: diagnosed with primary invasive or in situ BC	1,123 cases of BC	Strenuous exercise appears to reduce BC risk among post-menopausal women who do not gain sizable amounts of weight during adulthood.
		• Sex: Women		Multivariate adjusted OR (95%CI)	
USA		• Age: 55-64 yr			
		• Ethnicity: White (including Hispanic)	Controls: individually matched to each case patient based on birth date and race	• G1 = 1.00 (referent)	
Case control		• Characteristics: post-menopausal, English-speaking, born in USA, Canada or Western Europe		• G2 = 0.88 (0.72-1.07)	
D & B score = 15				• G3 = 0.55 (0.37-0.83)	
			PA assessment: Questionnaire for lifetime PA (MET hr/wk), divided into 3 groups	Trend *p *= 0.01	
			G1 = no activity		
			G2 = 0.1-17.59		
			G3 = ≥17.6		
			Conditional logistic regression		

Carpenter et al 2003 [308]	To examine the effects of obesity and lifetime exercise patterns on post-menopausal BC risk according to family history.	• n = 3,511 (cases n = 1,883, controls) n = 1,628	PA assessment: Interview for the following PA variables	1,883 cases of BC	Exercise independent of body size seemed to exert a protective effect primarily among women with a negative family history.
		• Sex: Women		Adjusted OR (95% CI) by lifetime exercise between menarche and reference date (MET hr/wk)	
USA		• Age: 55-72			
		• Characteristics: Postmenopausal Women	Lifetime exercise between menarche and reference date (MET hr/wk)		
Case control					
D & B score = 15			G1 = 0	• G1 = 1.00 (referent)	
			G2 = 0.1-3.74	• G2 = 0.85 (0.71-1.03)	
			G3 = 3.75-8.74		
			G4 = 8.75-17.59	• G3 = 0.87 (0.69-1.10)	
			G5 = ≥17.60		
				• G4 = 1.02 (0.79-1.30)	
			Average exercise activity in 10 years prior to reference date (MET hr/wk)		
				• G5 = 0.66 (0.48-0.90)	
			G1 = 0	Trend *p *= 0.07	
			G2 = 0.1-6.9		
			G3 = 7.0-13.9	Adjusted OR (95% CI) by average exercise activity in 10 years prior to reference date (MET hr/wk)	
			G4 = 14.0-24.4		
			G5 = ≥24.5		
				• G1 = 1.00 (referent)	
				• G2 = 0.93 (0.71-1.22)	
				• G3 = 0.92 (0.70-1.19)	
				• G4 = 0.86 (0.65-1.11)	
				• G5 = 0.75 (0.55-1.02)	
				Trend *p *= 0.05	

Chang et al 2006 [309]	To address the independent and combined effects of energy intake, BMI, and PA on BC incidence in women.	• n = 27,541	9.3 year follow-up (median 4.9 yr)	764 women developed BC	The study suggests that energy intake, BMI and physical inactivity are each independently and positively associated with BC risk.
		• Sex: Women			
USA		• Age: 55-74			
		• Characteristics: no history of any cancer (nonmelanoma skin cancer patients were included in the trial)	PA assessment: Questionnaire for vigorous PA (h/wk), divided into 6 groups	Multivariate adjusted RR (95%CI)	
Prospective cohort		• Prostate, Lung, colorectal, and Ovarian Cancer Screening Trial	G1 = 0		
			G2 = <1	• G1 = 1.00 (referent)	
D & B score = 13			G3 = 1	• G2 = 0.89 (0.69-1.15)	
			G4 = 2	• G3 = 0.96 (0.73-1.26)	
			G5 = 3	• G4 = 0.90 (0.70-1.16)	
			G6 = ≥4	• G5 = 1.02 (0.79-1.30)	
				• G6 = 0.78 (0.61-0.99)	
			Cox proportional HR	Trend *p *= 0.153	

Colditz et al 2003 [310]	To evaluate the relationship between PA and risk of pre-menopausal BC by type of activity and within subgroups of adiposity and oral contraceptive use.	• n = 110,468	PA assessment: Self report on 8 activities (walking or hiking, jogging (>10 min mile), running, Biking, racquet sports, lap swimming, calisthenics/aerobics other aerobic activities) to calculate MET scores (MET hrwk), divided into 5 groups:	Total cases diagnosed n = 849	These data among pre-menopausal women suggest that there is no overall association between PA and risk of BC. The findings also suggest that the effect of PA could be substantially modified by the underlying degree of adiposity.
		• Sex: Women			
USA		• Age: 25-42			
		• Characteristics: pre-menopausal, no history of cancer other than nonmelanoma skin cancer		Multivariate adjusted RR (95% CI)	
Prospective cohort				• G1 = 1.00 (referent)	
D & B score = 12				• G2 = 1.05 (0.82-1.34)	
				• G3 = 0.96 (0.75-1.23)	
			G1 = <3	• G4 = 1.05 (0.80-1.37)	
			G2 = 3-8.9		
			G3 = 9-17.9	• G5 = 1.07 (0.84-1.36)	
			G4 = 18-26.9		
			G5 = ≥27	Trend *p *= 0.69	
			Cox proportional HR		

Coogan et al 1997 [311]	To evaluate the effect of OPA on BC risk.	• n = 11,646 (4,863 cases and 6,783 controls).	PA assessment: Telephone interview to estimate OPA, divided into tertiles:	4,863 cases of BC	There was evidence of a graded inverse relationship between the intensity of work related activity and the incidence of BC.
				OR (95% CI)	
USA		• Sex: Women	T1 = Sedentary	• T1 = 1.00 (referent)	
		• Age: <74 yr	T2 = Medium activity jobs	• T2 = 0.86 (0.77-0.97)	
Case control			T3 = Heavy jobs		
				• T3 = 0.82 (0.63-1.08)	
D & B score = 14			Logistic regression models		

Coogan and Aschengrau 1999 [312]	To evaluate the effect of OPA on BC risk.	• n = 903 (233 case; 670 control)	PA assessment: Telephone interview to estimate OPA, divided into tertiles:	233 cases of BC	There was no evidence that holding a job of medium/heavy activity reduced BC.
					
		• Sex: Women		OR (95%CI)	
		• Age: <50 - 80+	T1 = Exclusively sedentary	• T1 = 1.00 (referent)	
USA		• Ethnicity: White, Black or Other	T2 = Exclusively light	• T2 = 1.20 (0.70-1.90)	
		• Characteristics: must have worked outside the home. Cases: All incident cases of BC reported to the Massachusetts Cancer Registry from 1983 to 1986 were eligible	T3 = Exclusively medium or heavy	• T3 = 0.90 (0.40-1.90)	The study was limited by OPA misclassification and by the lack of information on LTPA.
Case control				Trend *p *= 0.63	
D & B score = 14			Miettinen's test-based method and Fisher's exact method		

Dallal et al 2007 [313]	To examine the relationship between LTPA and invasive and in situ BC among women.	• n = 110,599	6.6 yr follow-up	2,649 cases of invasive	The results support a protective role of strenuous long-term exercise activity against invasive and in situ BC and suggest differing effects by hormone receptor status.
		• Sex: Women		BC	
USA		• Age: 20-79	PA assessment: Self-reported participation in moderate and strenuous activities to estimate annual strenuous physical activity (hr/wk), divided into quintiles	593 cases of in situ BC	
		• Ethnicity: White, Black, Hispanic, Asian, American Indian or other			
Prospective cohort		• Characteristics: California resident at baseline and no history of BC		Multivariate adjusted RR (95% CI) for invasive BC	
D & B score = 12		• California Teachers Study cohort		• Q1 = 1.00 (referent)	
				• Q2 = 0.93 (0.85-1.02)	
			Q1 = 0.00-0.50		
			Q2 = 0.51-2.00	• Q3 = 0.88 (0.78-0.99)	
			Q3 = 2.01-3.50		
			Q4 = 3.51-5.00	• Q4 = 1.02 (0.88-1.18)	
			Q5 = >5		
				• Q5 = 0.80 (0.69-0.94)	
			Cox proportional HR		
				Trend *p *= 0.02	
				Multivariate adjusted RR (95% CI) for in situ BC	
				• Q1 = 1.00 (referent)	
				• Q2 = 0.96 (0.79-1.17)	
				• Q3 = 0.86 (0.67-1.11)	
				• Q4 = 0.95 (0.70-1.30)	
				• Q5 = 0.69 (0.48-0.98)	
				Trend *p *= 0.40	

Dirx et al 2001 [314]	To evaluate the relationship between PA and BC risk with specific emphasis on interaction with other aspects of energy balance.	• n = 62,537	7.3 yr follow-up	1,208 cases of incident BC	The current study supports the hypotheses that PA is related inversely to BC risk in postmenopausal women.
		• Sex: Women			
Netherlands		• Age: 55-69	PA assessment: Questionnaire for total recreational PA (min/day), divided into quartiles		
		• Characteristics: healthy, postmenopausal		Multivariate adjusted RR (95% CI)	
Case study					
				• Q1 = 1.00 (referent)	
D & B score = 11			Q1 = <30	• Q2 = 0.84 (0.67-1.07)	
			Q2 = 30-60		
			Q3 = 61-90	• Q3 = 0.78 (0.60-1.00)	
			Q4 = >90		
				• Q4 = 0.76 (0.58-0.99)	
			Exponentially distributed failure time regression models		
				Trend *p *= 0.003	

Dorn et al 2003 [315]	To examine the associations between LTPA and OPA across the lifespan and pre- and post-menopausal BC.	• n = 1,550 (740 case; 810 control)	Cases: women diagnosed and histologically confirmed with BC	740 cases of BC	The study supports the hypothesis that strenuous LTPA is associated with a reduced risk of BC risk in both pre- and post menopausal women
USA		• Sex: Women		Multivariate adjusted OR (95%CI), pre- menopausal	
		• Age: 40-85			
Case control		• Characteristics: Case Group -- histologically confirmed incidence of BC. Control Group -- healthy	Controls: randomly selected and frequency matched on age and county with the cases.		
				• G1 = 1.00 (referent)	
D & B score = 13				• G2 = 0.94 (0.64-1.38)	
				• G3 = 0.73 (0.44-1.22)	
			PA assessment: Questionnaire for lifetime strenuous PA (hr/yr)	• G4 = 1.07 (0.57-2.02)	
				Trend *p *= 0.82	
			G1 = 0		
			G2 = 1-273		
			G3 = 274-545	Multivariate adjusted OR (95%CI), post-menopausal	
			G4 = >546		
			Logistic regression	• G1 = 1.00 (referent)	
				• G2 = 0.85 (0.61-1.19)	
				• G3 = 0.73 (0.45-1.17)	
				• G4 = 0.78 (0.47-1.29)	
				Trend *p *= 0.19	

Drake 2001 [316]	To evaluate PA as a predictor of BC and describe BC risk factors in this sample.	• n = 4,520	PA assessment: Self-report of type, intensity, duration and frequency of walking, jogging, biking, stationary biking, swimming, dancing, racket sports, stretching, participating in other exercise, calisthenics, weight-lifting and treadmill exercises, divided into groups	150 incident cases of breast cancer	Increased frequency of a specific PA (jogging) was found to have an important protective role in BC incidence.
		• Sex: Women			
USA		• Age: 21-86			
		• Characteristics: no diagnosis of BC at entry		OR (95% CI) for BC and PA	
Prospective cohort					
D & B score = 11		• Aerobic Center Longitudinal Study		Activity type	
				• G1 = 1.32	
				• G2 = 1.08	
				• G3 = 1.35	
				Trend *p *= 0.05	
			G1 = Aerobic (job, bike, aerobic dance)		
			G2 = Moderate (golf, walk)		
			G3 = Weight training		
			Chi-square		

Friedenreich et al 2001 [317]	To examine the type and dose of PA and the time periods in life when PA may be specifically associated with BC risk.	• n = 2,470 (1,233 case; 1,237 control)	Cases: in situ and invasive cases of BC from 1995-1997	1,233 cases of BC	This study provides evidence that lifetime PA reduces risk of post-menopausal BC.
Canada		• Sex: Women		OR (95%CI), pre-menopausal	
Case Control		• Age: ≤ 80	Controls: matched to cases on age and place of residence	• Q1 = 1.00 (referent)	
		• Characteristics: Case Group - Alberta residents, English speaking, capable of completing an in-person interview. Control Group -- no history of cancer diagnoses excluding nonmelanoma skin cancer		• Q2 = 1.15 (0.78- 1.70)	
D & B score = 13			PA assessment: Questionnaire for lifetime PA (MET hr/wk/yr), divided into quartiles by menopausal status	• Q3 = 1.15 (0.78- 1.69)	
			Pre-menopausal	• Q4 = 1.07 (0.72- 1.61)	
			Q1 = <86.6	Trend *p *= 0.50	
			Q2 = 86.6-108.3	OR (95%CI), post- menopausal	
			Q3 = 108.3-134.9	• Q1 = 1.00 (referent)	
			Q4 = ≥ 134.9	• Q2 = 0.73 (0.55- 0.98)	
			Post-menopausal	• Q3 = 0.75 (0.56- 1.00)	
			Q1 = <104.8	• Q4 = 0.70 (0.52- 0.94)	
			Q2 = 104.8-128.1	Trend *p *= 0.003	
			Q3 = 128.1-160.9		
			Q4 = ≥ 160.9		
			Logistic regression		

Friedenreich et al 2001 [318]	To examine the influence of frequency, duration, and intensity of PA on risk of BC and to compare BC risks associated with self-reported versus assigned intensity of PA.	• n = 2,470 (1,233 case; 1,237 control)	Cases: in situ and invasive cases of BC	1,233 cases of BC	This study found that moderate- intensity activities were the major contributors to the decrease in BC risk found in this study.
Canada		• Sex: Women		Multivariate adjusted OR (95% CI), pre- menopausal	
Case control		• Age: ≤ 80	Controls: matched to cases on age and place of residence	• Q1 = 1.00 (referent)	
D & B score = 13		• Characteristics: Case Group -- resident of Alberta, English speaking and able to complete an in-person interview. Control Group -- free of any cancer diagnosis excluding nonmelanoma skin cancer	PA assessment: Questionnaire for lifetime PA questionnaire (MET hr/wk/yr), divided into quartiles	• Q2 = 1.19 (0.80- 1.76)	
			Q1 = <28.8	• Q3 = 1.33 (0.90- 1.96)	
			Q2 = 28.8-35.4	• Q4 = 1.07 (0.71- 1.62)	
			Q3 = 35.4-42.7	Trend *p *= 0.52	
			Q4 = ≥ 42.7	Multivariate adjusted OR (95% CI), post- Menopausal	
			Logistic regression	• Q1 = 1.00 (referent)	
				• Q2 = 0.85 (0.64- 1.14)	
				• Q3 = 0.83 (0.62- 1.10)	
				• Q4 = 0.69 (0.51- 0.93)	
				Trend *p *= 0.006	

Friedenreich and Rohan 1995 [319]	To describe the association between LTPA and BC.	• n = 902 (451 case; 451 control)	Cases: first diagnosis of BC in 1982 and 1984	Adjusted OR (95%CI), pre-menopausal	This study found some evidence (of borderline statistical significance) that recreational PA is associated with decreased risk of BC.
Australia		• Sex: Women		• Q1 = 1.00 (referent)	
Case control		• Age: 20-74 yr	Controls: Randomly selected from the electoral roll, matched on date of birth to each case	• Q2 = 0.77 (0.36- 1.65)	
D & B score = 13		• Characteristics: Australian women	PA assessment: Self reported PA (kcal/wk), divided into quartiles	• Q3 = 0.48 (0.22- 1.03)	
			Q1 = 0	• Q4 = 0.60 (0.30- 1.17)	
			Q2 = 1-2,000	Trend *p *= 0.09	
			Q3 = 2000-4000	Adjusted OR (95%CI), post-menopausal	
			Q4 = >4000	• Q1 = 1.00 (referent)	
			Logistic regression models	• Q2 = 0.74 (0.46- 1.18)	
				• Q3 = 0.88 (0.53- 1.48)	
				• Q4 = 0.73 (0.44- 1.20)	
				Trend *p *= 0.32	

Gammon et al 1998 [320]	To examine the association between LTPA and BC among young women.	• n = 3,173 (1,668 case; 1,505 control)	Cases: women diagnosed with BC between 1990-1992	1,668 cases of BC	The study's data does not support the hypothesis of a reduced risk of BC among young women with increased recreational PA in adolescence, young adulthood or during the year prior to the interview, or with the average PA over the three time periods
USA		• Sex: Women		Multivariate adjusted OR (95%CI)	
Case control		• Age: <45	Controls: were matched to cases by age group and geographic center	• Q1 = 1.00 (referent)	
D & B score = 13		• Characteristics: Case Group -- diagnosed with invasive or in situ BC. Control Group - healthy	PA assessment: Questionnnaire for recreational PA, for ages 12- 13 yr, age 20 yr and 1 year prior to the interview. Divided into quartiles MET score	• Q2 = 0.79 (0.63- 0.98)	
			Q1 = 1.62-18.07	• Q3 = 0.98 (0.79- 1.22)	
			Q2 = 18.08-30.00	• Q4 = 1.01 (0.81- 1.25)	
			Q3 = 30.01-42.95	Trend *p *= 0.42	
			Q4 = 42.96-98.00		
			Logistic regression		

Gilliland et al 2001 [321]	To investigate the relationship of PA with BC risk in Hispanic and non- Hispanic White women	• n = 1,556 (712 case; 844 control)	Cases: diagnosed with BC between 1992-1994	712 cases of BC	Hispanic and non- Hispanic women with high PA during non-OPA were at substantially reduced risk of BC.
USA		• Sex: Women			
Case control		• Age: between 35-74 at diagnosis	Controls: matched on ethnicity, age and seven health planning districts	Adjusted OR (95%CI), pre-menopausal Hispanic	
D & B score = 13		• Ethnicity: Hispanic and non-Hispanic White	PA assessment: Self-reported non-OPA (MET hr/wk score)	• G1 = 1.00 (referent)	
		• Characteristics: Case Group -- diagnosed with in situ or invasive BC and residents of New Mexico at time of diagnosis. Control Group -- healthy	G1 = <25	• G2 = 1.17 (0.53- 2.55)	
			G2 = 25-50	• G3 = 0.49 (0.22- 1.07)	
			G3 = 50-80	• G4 = 0.29 (0.12- 0.72)	
			G4 = ≥ 80	Trend *p *< 0.001	
			Logistic regression	Adjusted OR (95%CI), pre-menopausal non- Hispanic	
				• G1 = 1.00 (referent)	
				• G2 = 1.35 (0.64- 2.85)	
				• G3 = 1.44 (0.67- 3.10)	
				• G4 = 1.13 (0.49- 2.61)	
				Trend *p *= 0.741	
				Adjusted OR (95%CI), post-menopausal Hispanic	
				• G1 = 1.00 (referent)	
				• G2 = 0.74 (0.40- 1.36)	
				• G3 = 0.37 (0.18- 0.75)	
				• G4 = 0.38 (0.18- 0.77)	
				Trend *p *= 0.002	
				Adjusted OR (95%CI), post-menopausal non- Hispanic	
				• G1 = 1.00 (referent)	
				• G2 = 0.45 (0.26- 0.78)	
				• G3 = 0.49 (0.28- 0.86)	
				• G4 = 0.45 (0.24- 0.85)	
				Trend *p *= 0.019	

Hsing et al 1998 [322]	To evaluate the role of selected demographic, lifestyle, and anthropometric factors in the risk for male BC.	• n = 690 (178 case; 512 control)	Cases: selected from 18,733 decedents included in the 1986 NMFS conducted by the US	178 cases of BC	This study suggests that obesity increases the risk of male BC, possibly through hormonal mechanisms, while dietary factors, PA and SES indicators also deserve further investigation.
USA		• Sex: Men	National Center for Health Statistics (NCHS)	Adjusted OR (95%CI)	
		• Age: 25-74		• G1 = 1.00 (referent)	
Case control		• Ethnicity: Black and White		• G2 = 0.60 (0.30- 1.10)	
D & B score = 12		• Characteristics: Case Group -- deceased. Control Group -- dying (or deceased) of causes other than BC	Controls: selected from male decedents dying of causes other than BC	• G3 = 1.30 (0.80- 2.00)	
			PA assessment: Questionnaire (frequency and intensity), divided into groups		
			G1 = Regular		
			G2 = Irregular		
			G3 = Hardly any		
			Logistic regression analysis		

Hu et al 1997 [323]	To study breast cancer focusing on breast-feeing, body weight, and PA as well as reproductive histories on pre- and post- menopausal Japanese women.	• n = 526 (157 case; 369 control)	Cases: Histologically confirmed cases of BC from 1989-1993.	157 cases of BC	Reduced risk of pre- menopausal BC was associated with high EE in PA during teenage years, although the trend was not statistically significant.
Japan		• Sex: Women		Unadjusted RR (95%CI), pre-menopausal	
Case control		• Age: 26-75		• G1 = 1.00 (referent)	
D & B score = 13		• Characteristics: Case Group -- histologically confirmed cases of BC and resident of Gifu prefecture at time of diagnosis. Control Group -- no breast disease or hormone- related (ovarian, endometrial and thyroid) cancers	Controls: individuals who had the screening test for BC during the same period	• G2 = 0.74 (0.38- 1.44)	
			PA assessment: Questionnaire for TPA (kcal/wk), divided into groups	• G3 = 1.01 (0.54- 1.87)	
			G1 = 0	Trend *p *= 0.876	
			G2 = 1-649	Unadjusted RR (95%CI), post-menopausal:	
			G3 = ≥ 650	• G1 = 1.00 (referent)	
				• G2 = 1.53 (0.69- 3.54)	
			Logistic regression models	• G3 = 1.39 (0.61- 3.13)	

John et al 2003 [324]	To examine BC risk in relation to lifetime histories of MPA and VPA including LTPA, transportation household and outdoor chores, and OPA in a multiethnic population.	• n = 2,870 (1,277 case; 1,593 control)	Cases: diagnosed between 1995-1998	1,277 cases of BC	This study supports previous reports of a reduced risk of BC in physically active women.
USA		• Sex: Women		Multivariate adjusted OR (95%CI), pre- menopausal Latinas	
Case control		• Age: 35-79		• G1 = 1.00 (referent)	
D & B score = 12		• Ethnicity: Latina, African-American and White	Controls: randomly selected according race/ethnicity and age distribution of cases	• G2 = 0.84 (0.49- 1.45)	
			PA assessment: In-person interview for lifetime PA (hr/wk), divided into groups	• G3 = 0.73 (0.42- 1.28)	
			Pre-menopausal	Multivariate adjusted OR (95%CI), pre- menopausal African Americans	
			G1 = <9.1	• G1 = 1.00 (referent)	
			G2 = 9.1-20.7	• G2 = 1.00 (0.55- 1.84)	
			G3 = ≥ 20.7	• G3 = 0.68 (0.35- 1.34)	
			Post-menopausal		
			G1 = <9.6		
			G2 = 9.6-21.6		
			G3 = ≥ 21.7		
			Logistic regression modeling	Multivariate adjusted OR (95%CI), pre- menopausal Whites	
				• G1 = 1.00 (referent)	
				• G2 = 0.82 (0.42- 1.58)	
				• G3 = 0.76 (0.36- 1.61)	
				Multivariate adjusted OR (95%CI), post- menopausal Latinas	
				• G1 = 1.00 (referent)	
				• G2 = 0.82 (0.55- 1.24)	
				• G3 = 0.81 (0.54- 1.22)	
				Multivariate adjusted OR (95%CI), post- menopausal African Americans	
				• G1 = 1.00 (referent)	
				• G2 = 0.78 (0.52- 1.17)	
				• G3 = 0.71 (0.47- 1.07)	
				Multivariate adjusted OR (95%CI), post- menopausal Whites	
				• G1 = 1.00 (referent)	
				• G2 = 0.94 (0.64- 1.37)	
				• G3 = 0.91 (0.60- 1.41)	

Kruk 2007 [325]	To examine the association between all types of PA and BC risk among Polish women.	• n = 590 (268 case; 322 control)	PA assessment: Questionnaire for lifetime PA (MET hr/wk/yr), divided into groups	268 cases of BC	The results of this study provide evidence of an inverse association between PA and the risk of BC.
Poland		• Sex: Women	G1 = <110	Multivariate adjusted OR (95%CI), pre- menopausal	
Case control		• Age: 35-75 yr	G2 = 110-150	• G1 = 1.00 (referent)	
D & B score = 13		• Characteristics: Polish women. Cases: identified from the Szczecin Regional Cancer Registry. Controls: matched on age and place of residence	G3 = >150	• G2 = 0.45 (0.14- 1.44)	
			Logistic regression analysis	• G3 = 0.44 (0.14- 1.37)	
				Trend *p *= 0.42	
				Multivariate adjusted OR (95%CI), post- menopausal	
				• G1 = 1.00 (referent)	
				• G2 = 0.60 (0.33- 1.09)	
				• G3 = 0.31 (0.21- 0.70)	
				Trend *p *= 0.002	

Kruk 2007 [326]	To examine the relationship between LTPA and BC risk.	• n = 822 (cases n = 257, control n = 565	PA assessment: Questionnaire for LTPA (METs), divided into groups	Adjusted OR (95% CI)	The findings provide further support to the hypothesis that increased LTPA throughout life is associated with a decreased risk of BC.
Poland		• Sex: Women	G1 = Low	• G1 = 1.00 (referent)	
Case control		• Age: 35-93 yr	G2 = Medium	• G2 = 0.57 (0.36- 0.89)	
D & B score = 13			G3 = High	• G3 = 0.22 (0.14- 0.35)	
				Trend *p *< 0.0001	

Lahmann et al 2007 [327]	To examine the association of PA with pre- and post-menopausal BC risk.	• n = 218,169	Baseline and 6.4 year follow-up	3,423 cases of BC	Increasing PA reduces BC risk.
Europe (9 countries)		• Sex: Women		Multivariate adjusted HR (95% CI) by TPA, pre- menopausal	
Prospective cohort		• Age: 20-80		• Q1 = 1.00 (referent)	
D & B score = 12		• The European Prospective Investigation into Cancer and nutrition study	PA assessment: Interviews and questionnaire for TPA and recreational PA, each divided into quartiles	• Q2 = 1.02 (0.84- 1.24)	
			TPA Index	• Q3 = 0.84 (0.68- 1.04)	
			Q1 = Inactive	• Q4 = 1.02 (0.77- 1.36)	
			Q2 = Moderately inactive	Trend *p *= 0.267	
			Q3 = Moderately active	Multivariate adjusted HR (95% CI) by TPA, Post- menopausal	
			Q4 = Active	• Q1 = 1.00 (referent)	
			Recreational PA (MET hr/wk)	• Q2 = 0.89 (0.79- 1.00)	
			Q1 = <14	• Q3 = 0.84 (0.74- 0.96)	
			Q2 = 14-24	• Q4 = 0.92 (0.76- 1.12)	
			Q3 = 25-42	Trend *p *= 0.06	
			Q4 = >42	Multivariate adjusted HR (95% CI) by recreational PA, pre-menopausal	
			Cox proportional index	• Q1 = 1.00 (referent)	
				• Q2 = 0.91 (0.75- 1.10)	
				• Q3 = 0.95 (0.78- 1.14)	
				• Q4 = 0.94 (0.76- 1.15)	
				Trend *p *= 0.580	
				Multivariate adjusted HR (95% CI) by recreational PA, post-menopausal	
				• Q1 = 1.00 (referent)	
				• Q2 = 1.05 (0.94- 1.17)	
				• Q3 = 0.92 (0.83- 1.03)	
				• Q4 = 0.96 (0.85- 1.08)	
				Trend *p *= 0.176	

Lee et al 2001 [328]	To examine the association between PA and BC risk.	• n = 39,322	Baseline and 4 year follow- up	411 cases of BC	The data suggest that PA during middle age and older is not uniformly associated with decreased BC risk. Among post- menopausal women only, higher levels of PA may decrease the risk of BC.
USA		• Sex: Women			
		• Age: ≥ 45 yr		Multivariate adjusted RR (95% CI) by PA, all women	
Prospective cohort		• Characteristics: Healthy women	PA assessment: Questionnaire	• Q1 = 1.00 (referent)	
D & B score = 12		Women's Health Study	PA (kJ/wk), divided into quartiles	• Q2 = 1.04 (0.77- 1.40)	
			Q1 = <840	• Q3 = 0.86 (0.64- 1.17)	
			Q2 = 840-2519	• Q4 = 0.80 (0.58- 1.12)	
			Q3 = 2520-6299	Trend *p *= 0.11	
			Q4 = ≥ 6300	Multivariate adjusted RR (95% CI) by PA, post- menopausal only	
			VPA (kJ/wk), divided into quintiles	• Q1 = 1.00 (referent)	
			Q1 = none	• Q2 = 0.97 (0.68- 1.39)	
			Q2 = 1-839	• Q3 = 0.78 (0.54- 1.12)	
			Q3 = 840-2099	• Q4 = 0.67 (0.44- 1.02)	
			Q4 = 2100-4199	Trend *p *= 0.03	
			Q5 = ≥ 4200	Multivariate adjusted RR (95% CI) by VPA, all women	
			Proportional hazard regression	• Q1 = 1.00 (referent)	
				• Q2 = 1.02 (0.70- 1.48)	
				• Q3 = 1.11 (0.78- 1.58)	
				• Q4 = 0.97 (0.66- 1.44)	
				• Q5 = 0.98 (0.69- 1.40)	
				Trend *p *= 0.98	
				Multivariate adjusted RR (95% CI) by VPA, post- menopausal only	
				• Q1 = 1.00 (referent)	
				• Q2 = 0.93 (0.57- 1.50)	
				• Q3 = 0.91 (0.57- 1.47)	
				• Q4 = 0.93 (0.57- 1.50)	
				• Q5 = 0.76 (0.47- 1.24)	
				Trend *p *= 0.29	

Magnusson et al 2005 [329]	To report the relationship between pre-menopausal BC, body fatness at age 10 years and in adulthood, and sports participation during puberty, late adolescence and early adulthood from three related case-control studies.	• n = 3,108 (1,560 cases; 1,548 controls)	PA assessment: Interview for sports participation (h/wk in the following age categories (12-14 yr, 16-18 yr, 20-30 yr, 12-30 yr, around age of diagnosis)	Adjusted RR (95% CI), 12-14 yr	An inverse association between body fatness but not PA at a young age and the risk of BC in pre-menopausal women.
UK		• Sex: Women		• G1 = 1.00 (referent)	
Case control		• Age: Study 1 = 36 yr, study 2 = 36-45 yr, study 3 = 46-54 yr		• G2 = 1.04 (0.93- 1.17)	
D & B score = 13		• Characteristics: White women with no previous malignancy, mental handicap or illness	Sports participation (h/wk)	• G3 = 1.03 (0.93- 1.14)	
			G1 = 0-1	Trend *p *= 0.95	
			G2 = 2-3	Adjusted RR (95% CI), 16-18 yr	
			G3 = ≥ 4	• G1 = 1.00 (referent)	
				• G2 = 0.95 (0.83- 1.09)	
				• G3 = 0.89 (0.79- 1.02)	
				Trend *p *= 0.20	
				Adjusted RR (95% CI), 20-30 yr	
				• G1 = 1.00 (referent)	
				• G2 = 0.90 (0.76- 1.08)	
				• G3 = 1.01 (0.81- 1.26)	
				Trend *p *= 0.73	
				Adjusted RR (95% CI), 12-30 yr	
				• G1 = 1.00 (referent)	
				• G2 = 0.99 (0.89- 1.11)	
				• G3 = 1.01 (0.88- 1.16)	
				Trend *p *= 0.94	
				Adjusted RR (95% CI), around age of diagnosis	
				• G1 = 1.00 (referent)	
				• G2 = 0.84 (0.71- 1.00)	
				• G3 = 1.06 (0.86- 1.32)	
				Trend *p *= 0.82	

Malin et al 2005 [330]	To evaluate a pattern of behavioral exposures indicating positive energy balance would be associated with increased BC risk.	• n = 3,015 (1,459 cases; 1,556 control)	PA assessment: Questionnaire for PA (MET hr/d/yr), divided into groups	OR (95% CI)	The study suggests that promotion of behavioral patterns that optimize energy balance maybe a viable option for BC prevention.
China		• Sex: Women	G1 = 0	• G1 = 1.86 (1.44- 2.41)	
Case control		• Age: Mean ~47 yr	G2 = 0.1-2.92	• G2 = 1.33 (0.96- 1.83)	
D & B score = 12		• Characteristics: Residents of urban Shanghai	G3 = >2.92	• G3 = 1.00 (referent)	
		Shanghai Breast Cancer Study			

Margolis et al 2005 [331]	To study the association between PA and incident invasive BC.	• n = 99,504	Baseline and 9.1 year follow-up	1,166 cases of BC	No evidence of a protective effect of PA on BC risk was found.
Norway/Sweden		• Sex: Women			
Prospective cohort		• Age: 30-49 (mean 41 yr)	PA assessment: Questionnaire for PA using a 5 point scale and for competitive PA (years of participation), each divided into groups	Multivariate adjusted RR (95% CI) by PA level, at enrollment	
D & B score = 13		• The Norwegian- Swedish Women's Lifestyle and Health Study	PA level (5 point scale)	• G1 = 1.00 (referent)	
			G1 = None	• G2 = 1.35 (0.96- 1.90)	
			G2 = Low	• G3 = 1.26 (0.91- 1.74)	
			G3 = Moderate	• G4 = 1.19 (0.85- 1.67)	
			G4 = High	• G5 = 1.24 (0.85- 1.82)	
			G5 = Vigorous	Trend *p *= 0.85	
			Competitive PA (years)	Multivariate adjusted RR (95% CI) by PA level, at age 30	
			G1 = None	• G1 = 1.00 (referent)	
			G2 = 1-4	• G2 = 1.03 (0.64- 1.66)	
			G3 ≥ 5	• G3 = 1.16 (0.74- 1.81)	
				• G4 = 1.06 (0.67- 1.68)	
				•G5 = 1.20 (0.77- 1.95)	
				Trend *p *= 0.60	
				Multivariate adjusted RR (95% CI) by PA level, at age 14	
				• G1 = 1.00 (referent)	
				• G2 = 0.93 (0.62- 1.39)	
				• G3 = 0.94 (0.65- 1.35)	
				• G4 = 1.07 (0.73- 1.55)	
				• G5 = 1.05 (0.72- 1.54)	
				Trend *p *= 0.14	
				Multivariate adjusted RR (95% CI) by years of competitive PA	
				• G1 = 1.00 (referent)	
				• G2 = 1.21 (0.95- 1.54)	
				• G3 = 0.95 (0.75- 1.19)	
				Trend *p *= 0.96	

McTiernan et al 1996 [332]	To investigate the relationship between LTPA and BC.	• n = 1,029 (cases n = 537, controls n = 492)	PA assessment: Questionnaire (Minnesota LTPA Questionnaire) for LTPA (hr/wk), divided into groups	Adjusted OR (95% CI) by LTPA during adulthood, all ages and menopausal status	The results indicate a weak negative association between PA and risk of BC in middle-aged women.
USA		• Sex: Women	G1 = None	• G1 = 1.00 (referent)	
Case control		• Age: 50-64	G2 = 0.1-1.5	• G2 = 1.1 (0.7-1.6)	
D & B score = 13			G3 = 1.6-2.5	• G3 = 0.7 (0.4-1.1)	
			G4 = 2.6-3.5	• G4 = 0.7 (0.4-1.1)	
			G5 = 3.6-5.0	• G5 = 0.6 (0.4-0.9)	
			G6 = >5	• G6 = 1.1 (0.7-1.6)	
			Calculated categories of EE (total time x intensity code)	Trend *p *= 0.29	
			G1 = Lowest	Adjusted OR (95% CI) by LTPA during adulthood, aged ≥ 55 yr, post-menopausal only	
			G6 = Highest	• G1 = 1.00 (referent)	
				• G2 = 0.8 (0.5-1.3)	
				• G3 = 0.5 (0.3-0.9)	
				• G4 = 0.6 (0.4-1.1)	
				• G5 = 0.4 (0.2-0.8)	
				• G6 = 0.8 (0.5-1.3)	
				Trend *p *= 0.03	
				Adjusted OR (95% CI) by category of total EE in adulthood, all ages and menopausal status	
				• G1 = 1.00 (referent)	
				• G2 = 1.2 (0.8-2.0)	
				• G3 = 0.9 (0.6-1.3)	
				• G4 = 0.6 (0.4-0.9)	
				• G5 = 0.9 (0.6-1.5)	
				• G6 = 0.9 (0.6-1.4)	
				Trend *p *= 0.25	
				Adjusted OR (95% CI) by category of total EE in adulthood, aged ≥ 55 yr, post-menopausal only	
				• G1 = 1.00 (referent)	
				• G2 = 0.8 (0.4-1.4)	
				• G3 = 0.7 (0.4-1.2)	
				• G4 = 0.5 (0.3-0.8)	
				• G5 = 0.8 (0.5-1.3)	
				• G6 = 0.6 (0.4-1.0)	
				Trend *p *= 0.009	

McTiernan et al 2003 [333]	To examine the association between current and past LTPA and incidence of BC in post-menopausal women.	• n = 74,171	Baseline and mean follow-up of 4.7 years	1,780 cases of BC	Increased PA is associated with reduced risk for BC in post-menopausal women.
USA		• Sex: Women	PA assessment: Questionnaire for TPA (MET hr/wk), moderate or strenuous PA (hr/wk) and strenuous PA (hr/wk), each divided into groups	Adjusted RR (95% CI) by TPA	Longer duration provides the most benefit however need not be strenuous.
Prospective cohort		• Age: 50-79	TPA (MET hr/wk)	• G1 = 1.00 (referent)	
D & B score = 13		• Characteristics: Women from the Women's Health Initiative Observational Study	G1 = none	• G2 = 0.90 (0.77-1.07)	
			G2 = 0-5.0	• G3 = 0.82 (0.68-0.97)	
			G3 = 5.1-10.0	• G4 = 0.89 (0.76-1.00)	
			G4 = 10.1-20.0	• G5 = 0.83 (0.70-0.98)	
			G5 = 20.1-40	• G6 = 0.78 (0.62-1.00)	
			G6 = ≥ 40.0	Trend *p *= 0.03	
			Moderate or strenuous PA (hr/wk)	Adjusted RR (95% CI) by TPA,	
			G1 = none	BMI ≤ 24.13	
			G2 = ≤ 1	• G1 = 1.00 (referent)	
			G3 = 1.1-2.0	• G2 = 0.78 (0.57-1.10)	
			G4 = 2.1-3.0	• G3 = 0.70 (0.51-0.97)	
			G5 = 3.1-4.0	• G4 = 0.80 (0.60-1.10)	
			G6 = 4.1-7.0	• G5 = 0.68 (0.51-0.92)	
			G7 = >7.0	• G6 = 0.63 (0.43-0.93)	
			Strenuous PA (hr/wk)	Trend *p *= 0.03	
			G1 = none	Adjusted RR (95% CI) by TPA, BMI 24.14-28.44	
			G2 = ≤ 1.0	• G1 = referent	
			G3 = 1.1-2.0	• G2 = 0.72 (0.53-0.98)	
			G4 = 2.1-4.0	• G3 = 0.78 (0.57-1.10)	
			G5 = >4.0	• G4 = 0.77 (0.58-1.00)	
			Cox proportional hazard ratio	• G5 = 0.85 (0.64-1.10)	
				• G6 = 0.78 (0.52-1.20)	
				Trend *p *= 0.74	
				Adjusted RR (95% CI) by TPA, BMI >28.44	
				• G1 = 1.00 (referent)	
				• G2 = 1.10 (0.88-1.50)	
				• G3 = 0.90 (0.67-1.20)	
				• G4 = 1.00 (0.79-1.30)	
				• G5 = 0.89 (0.65-1.20)	
				• G6 = 0.94 (0.57-1.60)	
				Trend *p *= 0.30	
				Adjusted RR (95% CI) by current moderate or strenuous PA	
				• G1 = 1.00 (referent)	
				• G2 = 0.92 (0.78-1.10)	
				• G3 = 0.91 (0.79-1.10)	
				• G4 = 0.94 (0.81-1.10)	
				• G5 = 0.99 (0.83-1.20)	
				• G6 = 0.91 (0.78-1.10)	
				• G7 = 0.79 (0.63-0.99)	
				Trend *p *= 0.12	
				Adjusted RR (95% CI) by current strenuous PA	
				• G1 = 1.00 (referent)	
				• G2 = 0.94 (0.80-1.10)	
				• G3 = 0.95 (0.80-1.10)	
				• G4 = 0.93 (0.78-1.10)	
				• G5 = 0.91 (0.67-1.20)	
				Trend *p *= 0.25	

Navarro Silvera et al 2006 [334]	To study the independent and combined associations of VPA, energy consumption and BMI with risk of subsequent BC.	• n = 40,318 in analysis (49,613 prior to exclusion)	Baseline and 16.4 year follow-up	1,673 cases of BC from the 40,318 included in the analysis (2,545 cases from total prior to exclusion)	The results of the study suggest that BC risk may vary according to various combinations of the components of energy balance.
Canada		• Sex: Women	PA assessment: Questionnaire for VPA (min/d), divided into groups	Adjusted HR (95% CI) by VPA	
Prospective cohort		• Age: 40-59	G1 = none	• G1 = 1.00 (referent)	
D & B score = 13		• Characteristics: Canadian women with no history of BC	G2 = Any	• G2 = 0.98 (0.85-1.13)	
		• National Breast Screening Study (NBSS)	G3 = 0-30	• G3 = 1.06 (0.88-1.27)	
			G4 = 30-60	• G4 = 0.98 (0.83-1.16)	
			G5 > 60	• G5 = 0.93 (0.78-1.10)	
			Cox proportional hazard ratio	Trend *p *= 0.38	
				Adjusted HR (95% CI) by VPA, pre-menopausal	
				• G1 = 1.00 (referent)	
				• G2 = 0.91 (0.75-1.10)	
				• G3 = 1.02 (0.80-1.31)	
				• G4 = 0.88 (0.70-1.11)	
				• G5 = 0.87 (0.68-1.09)	
				Trend *p *= 0.23	
				Adjusted HR (95% CI) by VPA, post- menopausal	
				• G1 = 1.00 (referent)	
				• G2 = 1.06 (0.87-1.30)	
				• G3 = 1.08 (0.81-1.42)	
				• G4 = 1.11 (0.87-1.41)	
				• G5 = 1.00 (0.78-1.29)	
				Trend *p *= 0.96	

Patel et al 2003 [335]	To examine the association between various measures of PA and post-menopausal BC risk.	• n = 72,608	Baseline and 5 year follow-up	1,520 cases of breast cancer	The study shows a lower risk of post- menopausal BC is associated with regular PA.
USA		• Sex: Women	PA assessment: Questionnaire for LTPA (METs hr/wk) at various times during life, divided into groups	Adjusted RR (95% CI), LTPA at study entry	
Prospective cohort		• Age: 50-74	G1 = none	• G1 = 0.86 (0.70- 1.04)	
D & B score = 14		• Characteristics: Postmenopausal women	G2 = 0.1-6.9	• G2 = 1.00 (referent)	
		• The American Cancer Society Cancer Prevention Study II (CPS-II) Nutritional Cohort	G3 = 7.0-17.5	• G3 = 0.92 (0.81- 1.04)	
			G4 = 17.6-31.5	• G4 = 0.94 (0.81- 1.09)	
			G5 = 31.6-42.0	• G5 = 0.77 (0.56- 1.06)	
			G6 = >42.0	• G6 = 0.71 (0.49- 1.02)	
			LTPA at 10 years prior to study, calculated MET score and categorized into groups:	Trend *p *= 0.08 (among active women p = 0.03)	
			None	Adjusted RR (95% CI), LTPA at age 40 yr	
			Slight	• G1 = 1.03 (0.88- 1.21)	
			Moderate	• G2 = 1.00 (referent)	
			Heavy	• G3 = 1.05 (0.92- 1.20)	
			Cox proportional hazard ratio	• G4 = 1.01 (0.87-1.18)	
				• G5 = 1.16 (0.92- 1.46)	
				• G6 = 0.79 (0.61- 1.03)	
				Trend *p *= 0.31 (among active women p = 0.36)	
				Adjusted RR (95% CI), LTPA at 10 years prior to study entry	
				• None = 0.80 (0.51- 1.25)	
				• Slight = 1.00 (referent)	
				• Moderate = 0.93 (0.83-1.04)	
				• Heavy = 0.87 (0.68- 1.13)	
				Trend *p *= 0.32 (among active women, trend *p *= 0.16)	

Patel et al 2003 [336]	To evaluate the association between lifetime LTPA and BC risk.	• n = 1,183 (cases n = 616) n = 567, controls n = 616)	PA assessment: Calendar reporting for lifetime exercice activity (MET h/wk), divided into groups	Adjusted OR (95% CI)	The findings suggest that PA may modify the risk of in situ BC particularly in women without a family history of BC.
USA		• Sex: Women	G1 = None	• G1 = 1.00 (referent)	
Case control		• Age: 35-64	G2 = 0.0-3.0	• G2 = 0.70 (0.48- 1.03)	
D & B score = 14		• Characteristics: White and Black women	G3 = 3.0-8.0	• G3 = 0.65 (0.44- 0.96)	
			G4 = 8.0-16.0	• G4 = 0.61 (0.41- 0.92)	
			G5 = 16.0-32.0	• G5 = 0.63 (0.40- 0.98)	
			G6 = >32.0	• G6 = 0.65 (0.39- 1.08)	
			Unconditional logistical regressioin	Trend *p *= 0.27 (among exercisers only *p *= 0.81)	

Rintala et al 2002 [337]	To obtain an estimate of BC incidence in association with self-rated OPA.	• n = 680,000	PA assessment: Self-reported OPA in 5 classes (1=low, 5=high)	17,986 cases of BC	The results support the hypothesis that OPA, if high enough, markedly reduced BC risk.
Finland		• Sex: Women	Class 1 = Jobs sitting and light hand tasks	Adjusted RR (95% CI), age 25-39 years	
Prospective cohort		• Age: Women born in 1930-1969	Class 2 = Handling of heavier items (conveyor belt)	• C1+2 = 1.00 (referent)	
D & B score = 11		• Characteristics: Finish women	Class 3 = Jobs involving body motion	• C3 = 0.99 (0.85- 1.17)	
			Class 4 = Jobs involving walking up stairs or long distances, bending and carrying	• C4 = 0.90 (0.76- 1.07)	
			Class 5 = Same as class 4 except heavy tasks were performed for most of the day	• C5 = 0.68 (0.51- 1.93)	
			Poisson regression models	Trend	
				Adjusted RR (95% CI), age 40-54 years	
				• C1+2 = 1.00 (referent)	
				• C3 = 1.02 (0.94- 1.11)	
				• C4 = 0.99 (0.91- 1.09)	
				• C5 = 0.84 (0.70- 1.00)	
				Trend	
				Adjusted RR (95% CI), age ≥ 55 years	
				• C1+2 = 1.00 (referent)	
				• C3 = 1.01 (0.96- 1.07)	
				• C4 = 1.04 (0.98- 1.11)	
				• C5 = 0.82 (0.71- 0.94)	
				Trend	

Rockhill et al 1998 [338]	To examine the association between PA at two different times in life and BC risk.	• n = 372	Baseline and 6 year follow-up	372 cases of BC	The findings do not support a link between PA in late adolescence or in the recent past and BC risk among young adult women.
USA		• Sex: Women	PA assessment: Questionnaire for MVPA (h/wk)	Multivariate adjusted RR (95% CI)	
Prospective cohort		• Age: 25-42	G1 = <1	• G1 = 1.00 (referent)	
D & B score = 12		• Characteristics: Nurses	G2 = 1.0-1.9	• G2 = 1.1 (0.8-1.4)	
		• The Nurses Health Study	G3 = 2.0-3.9	• G3 = 1.1 (0.8-1.4)	
			G4 = 4.0-6.9	• G4 = 1.0 (0.7-1.4)	
			G5 = ≥ 7	• G5 = 1.1 (0.8-1.5)	
			Logistic regression		
Slattery et al 2007 [339]	To evaluate the BC risk associated with TPA and VPA at ages 15, 30 and 50 years and the referent year prior to diagnosis/selection.	• n = 4,850 Non-Hispanic white: n = 3,128 (cases n = 1,527 controls n = 1601); Hispanic American Indian: n = 1,722 (cases n = 798, controls n = 924)	PA assessment: Questionnaire for TPA (activity score) and lifetime VPA (h/wk)	1527 cases of BC (non- Hispanic white; 798 cases of BC (Hispanic American Indian)	The data suggest that PA is important in reducing risk of BC in non-Hispanic white and Hispanic American Indian women.
USA		• Sex: Women	TPA score		
Case control		• Age: <50 yr	G1 = 0-3		
D & B score = 12		• Characteristics: Non- Hispanic white and Hispanic American Indian	G2 = 4-6	OR (95% CI) by TPA score, non-Hispanic white	
			G3 = 7-9	• G1 = 1.00 (referent)	
			G4 = 10-12	• G2 = 0.78 (0.52- 1.17)	
			Lifetime VPA	• G3 = 0.84 (0.57- 1.22)	
			G1 = None	• G4 = 0.70 (0.44- 1.12)	
			G2 = <1.0	Trend *p *= 0.26	
			G3 = 1.0-2.9	OR (95% CI) by TPA score, Hispanic American Indian	
			G4 = ≥ 3.0	• G1 = 1.00 (referent)	
			Multivariable logistic regression	• G2 = 1.49 (0.98- 2.26)	
				• G3 = 1.21 (0.80- 1.84)	
				• G4 = 0.97 (0.53- 1.76)	
				Trend *p *= 0.90	
				OR (95% CI) by lifetime VPA, non-Hispanic white	
				• G1 = 1.00 (referent)	
				• G2 = 0.66 (0.36- 1.23)	
				• G3 = 0.73 (0.40- 1.34)	
				• G4 = 0.69 (0.37- 1.27)	
				Trend *p *= 0.68	
				OR (95% CI) by lifetime VPA, Hispanic American Indian	
				• G1 = 1.00 (referent)	
				• G2 = 1.15 (0.67- 1.96)	
				• G3 = 1.19 (0.70- 2.03)	
				• G4 = 1.09 (0.62- 1.90)	
				Trend *p *= 0.84	

Sprague et al 2007 [340]	To investigate the relationship between LTPA and strenuous OPA and BC risk.	• n = 15,710 (1,689 cases in situ; 6,391 invasive and 7,630 controls)	PA assessment: Questionnaire for lifetime TPA (hr/wk and MET hr/wk), divided into groups	Adjusted OR (95% CI) for in situ BC by lifetime TPA (hr/wk)	The results provide further evidence that for most women, PA may reduce the risk of invasive BC.
USA		• Sex: Women	Lifetime total PA (hr/wk)	• G1 = 1.00 (referent)	
Case control		• Age: 20-69	G1 = 0	• G2 = 0.92 (0.72-1.19)	
D & B score = 13		• The Collaborative Breast Cancer Study	G2 = 0.1-15.0	• G3 = 0.83 (0.62-1.13)	
			G3 = 15.1-30.0	• G4 = 0.86 (0.59- 1.24)	
			G4 = > 30.0	Trend *p *= 0.22	
			MET hr/wk	Adjusted OR (95% CI) for in situ BC by lifetime TPA (MET hr/wk)	
			G1 = 0.0	• G1 = 1.00 (referent)	
			G2 = 0.1-62.5	• G2 = 0.93 (0.72- 1.20)	
			G3 = 62.6-125.0	• G3 = 0.82 (0.61- 1.10)	
			G4 = >125.0	• G4 = 0.82 (0.57- 1.17)	
				Trend *p *= 0.10	
				Adjusted OR (95% CI) for invasive BC by lifetime TPA (hr/wk)	
				• G1 = 1.00 (referent)	
				• G2 = 0.88 (0.76- 1.03)	
				• G3 = 0.87 (0.73- 1.05)	
				• G4 = 0.85 (0.67- 1.07)	
				Trend *p *= 0.22	
				Adjusted OR (95% CI) for invasive BC by lifetime TPA (MET hr/wk)	
				• G1 = 1.00 (referent)	
				•G2 = 0.89 (0.76- 1.04)	
				• G3 = 0.82 (0.68- 0.99)	
				• G4 = 0.88 (0.71- 1.09)	
				Trend *p *= 0.12	

Steindorf et al 2003 [341]	To clarify the relationship between PA and BC risk.	• n = 1,246 (360 cases; 886 controls)	PA assessment: Computer assisted telephone interview for TPA (MET hr/wk) at various ages	360 cases of BC	The data do not suggest an inverse association between PA and BC risk in pre-menopausal women.
Germany		• Sex: Women	TPA at age 12-19 yr	Multivariate adjusted OR (95% CI) by TPA at age 12-19 yr	
Case control		• Age: Mean. cases 41.9 yr; controls 42.5 yr	G1 = 13.0-55.7	• G1 = 1.00 (referent)	
D & B score = 13			G2 = 55.8-88.7	• G2 = 1.07 (0.75- 1.52)	
			G3 = 88.8-134.0	• G3 = 1.00 (0.70- 1.42)	
			G4 = 134.1-695.9	• G4 = 0.73 (0.50- 1.07)	
			TPA at age 20-30 yr	Trend *p *= 0.44	
			G1 = 6.4-69.0	Multivariate adjusted OR (95% CI) by TPA at age 20-30 yr	
			G2 = 69.1-109.0	• G1 = 1.00 (referent)	
			G3 = 109.1-160.4	• G2 = 0.95 (0.67- 1.37)	
			G4 = 160.5-728.8	• G3 = 0.85 (0.59- 1.23)	
			TPA at age 12-30 yr (both)	• G4 = 0.96 (0.67- 1.39)	
			G1 = 17.2-70.4	Trend *p *= 0.32	
			G2 = 70.5-104.0	Multivariate adjusted OR (95% CI) by TPA at age 12-30 yr	
			G3 = 104.1-145.5	• G1 = 1.00 (referent)	
			G4 = 145.6-564.4	• G2 = 0.97 (0.68- 1.38)	
			Logistic regression	• G3 = 0.68 (0.46- 0.99)	
				G4 = 0.94 (0.65- 1.35)	
				Trend *p *= 0.29	

Tehard et al 2006 [342]	To investigate the type, duration, frequency and intensity of PA required to reduce the risk of BC.	• n = 90,509	Baseline and follow-up every 2 years for 12 years	3,424 cases of BC	BC risk was reduced, especially with VPA.
France		• Sex: Women	PA assessment: Questionnaire for various PA variables, all divided into groups	Multivariate adjusted RR (95% CI) by TPA	
Prospective cohort		• Age: 40-65	TPA (MET hr/wk)	• G1 = 1.00 (referent)	
D & B score = 13		• Characteristics: French women insured with Mutuelle Generale de l'Education Nationale	G1 = <28.3	• G2 = 1.05 (0.93- 1.17)	
		• E3N Cohort Study	G2 = 28.3-41.8	• G3 = 0.94 (0.83- 1.05)	
			G3 = 41.8-57.8	• G4 = 0.90 (0.80- 1.02)	
				Trend *p *< 0.05	
			G4 = ≥ 57.8	Multivariate adjustedRR (95% CI) by total recreational PA	
			Total recreational PA (MET hr/wk)	• G1 = 1.00 (referent)	
			G1 = Inactive	• G2 = 0.82 (0.71- 0.93)	
			G2 = <16.0	• G3 = 0.94 (0.84- 1.06)	
			G3 = 16.0-22.3	• G4 = 0.88 (0.79- 0.98)	
			G4 = 22.3-33.8	• G5 = 0.81 (0.72- 0.92)	
			G5 = ≥33.8 = 0.81	Trend *p *< 0.01	
			Walking (min/d)	Multivariate adjusted RR (95% CI) by walking duration	
			G1 = <500	• G1 = 1.00 (referent)	
			G2 = 500-2000	• G2 = 1.03 (0.95- 1.11)	
			G3 = >2000	• G3 = 0.91 (0.81- 1.02)	
			MPA (hr/wk)	Trend *p *= 0.45	
			G1 = Inactive	Multivariate adjusted RR	
			G2 = 0	(95% CI) by MPA	
			G3 = 1-4	• G1 = 1.00 (referent)	
			G4 = 5-13	• G2 = 0.80 (0.60- 1.05)	
			G5 = 14	• G3 = 0.87 (0.79- 0.94)	
			VPA (hr/wk)	• G4 = 0.86 (0.74- 0.99)	
			G1 = Inactive	• G5 = 0.89 (0.65- 1.24)	
			G2 = 0	Trend *p*<0.01	
			G3 = 1-2	Multivariate adjusted RR (95% CI) by VPA	
			G4 = 3-4	• G1 = 1.00 (referent)	
			G5 = 5	• G2 = 0.90 (0.81- 0.99)	
			Cox proportional hazard ratio	• G3 = 0.88 (0.79- 0.97)	
				• G4 = 0.82 (0.71- 0.95)	
				• G5 = 0.62 (0.49- 0.78)	
				Trend *p *< 0.0001	

Thune et al 1997 [343]	To investigate whether everyday exercise is related to the risk of BC.	• n = 25,624	Baseline and mean follow- up of 14 years	351 cases of BC (110 pre-menopausal and 251 post-menopausal women)	LTPA and OPA are associated with a reduced risk of BC.
Norway		• Sex: Women	PA assessment: Self-reported LTPA and OPA, divided into groups	• G1 = 1.00 (referent)	Adjusted RR (95% CI) by LTPA
Prospective cohort		• Age: 20-54	LTPA	• G2 = 0.93 (0.71- 1.22)	
D & B score = 14			G1 = Sedentary	• G3 = 0.63 (0.42- 0.95)	
			G2 = Moderate	Trend *p *= 0.04	
			G3 = Regular exercise OPA	Adjusted RR (95% CI) by LTPA, pre- menopausal	
			G1 = Sedentary	• G1 = 1.00 (referent)	
			G2 = Walking	• G2 = 0.77 (0.46- 1.27)	
			G3 = Lifting	• G3 = 0.53 (0.25- 1.14)	
			G4 = Heavy manual labor	Trend *p *= 0.10	
			During work	Adjusted RR (95% CI) by LTPA, post- menopausal	
			Pre-menopausal	• G1 = 1.00 (referent)	
			G1 = Sedentary	• G2 = 1.00 (0.72- 1.39)	
			G2 = Walking	• G3 = 0.67 (0.41- 1.10)	
			G3 = Lifting or heavy manual labor	Trend *p *= 0.15	
				Adjusted RR (95% CI) by OPA	
				• G1 = 1.00 (referent)	
				• G2 = 0.84 (0.63- 1.12)	
				• G3 = 0.74 (0.52- 1.06)	
				• G4 = 0.48 (0.25- 0.92)	
				Trend *p *= 0.02	
				Adjusted RR (95% CI) by OPA, pre- menopausal	
				• G1 = 1.00 (referent)	
				• G2 = 0.82 (0.50- 1.34)	
				• G3 = 0.48 (0.24- 0.95)	
				Trend *p *= 0.03	
				Adjusted RR (95% CI) by OPA, post- menopausal	
				• G1 = 1.00 (referent)	
				• G2 = 0.87 (0.61- 1.24)	
				• G3 = 0.78 (0.52- 1.18)	
				Trend *p *= 0.24	

Zheng et al 1993 [344]	To assess the role of OPA in the risk of BC.	• n= 3,783 (BC = 2,736)	PA assessment: Interview for OPA, divided into groups	2,736 cases of BC	Women with low OPA had an increased risk of BC; the incidence of BC was reduced in women with high-activity jobs.
China		• Sex: Women	G1 = Low	Standardized incidence ratios	
D & B score = 9		• Age: 30	G2 = Moderate	• G1= 131	
			G3 = High	• G2 = 95	
				• G3 = 79	

D & B score, Downs and Black quality score; YR, years; PA, physical activity; BC, breast cancer; LTPA, leisure-time physical activity; g, group; HR, hazard ratio; RR, risk ratio; OR, odds ratio; 95% CI, confidence interval; T, tertile; MET, metabolic equivalent; MET/wk, metabolic equivalent per week; OPA, occupational physical activity; MET h/wk/yr, metabolic equivalent per hour per week per year; kcal/wk, kilocalories per week; TPA, total physical activity; VPA, vigorous physical activity.

**Figure 8:**
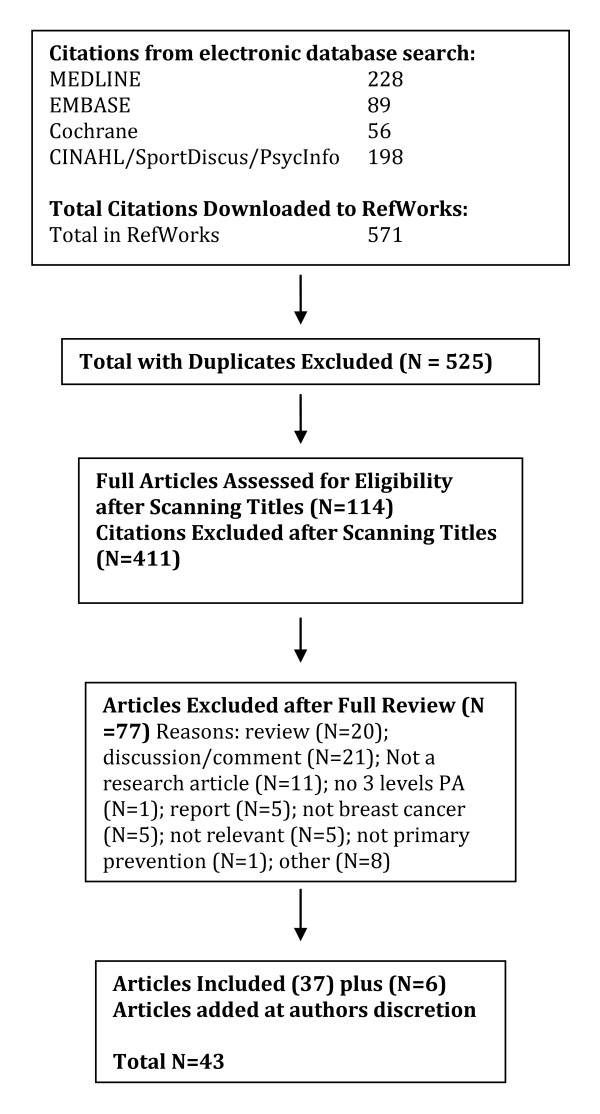
**Results of the Literature Search for Breast Cancer**.

In our systematic search of the literature, a total of 571 citations were identified during the electronic database search (Figure 8). Of these citations, 228 were identified in MEDLINE, 89 in EMBASE, 56 in Cochrane, and 198 in the CINAHL/SportDiscus/PsychInfo search. A total of 46 duplicates were found, leaving a total of 571 unique citations. A total of 411 articles were excluded after scanning, leaving a total of 114 articles for full review. From these articles 77 were excluded after full review leaving 37 articles for inclusion in the systematic review. An additional 6 articles were found through the reviewers' personal files. The reasons for exclusion included not containing three levels of physical activity or not possible to determine dose-response relationship (n = 1), reviews, summaries, meta-analyses (n = 20), report (n = 5), editorial/comment (n = 21), not a research article (N = 11), not dealing specifically with breast cancer (n = 4), not relevant (n = 5), not primary prevention (n = 3), and other (n = 10). Therefore, a total of 43 articles were included in the systematic review of the literature regarding the relationship between physical activity and the primary prevention of breast cancer.

The data providing dose-response information is all observational in nature, involving both case control and cohort investigations. These studies involved a total of 1,861,707 participants averaging 44,326 subjects per study (range 526-680,000). There were a total of 80,247 reported cases of breast cancer (ranging per study from 109-17,986). The total length of study follow-up for the prospective cohort studies averaged 10.5 yr (ranging from 4-31 yr). The articles were published over a 14 yr period ranging from 1993-2007. These studies involved large samples of men and women from regions throughout the world.

The literature with respect to the primary prevention of breast cancer is as compelling as that found with respect to colon cancer. There is strong evidence that routine physical activity is associated with a reduced risk for the development of breast cancer. However, this literature is also confounded by many shortcomings (similar to other cancer literature) including considerable variability in the statistical analyses employed, the physical activity measurement tools used, and the experimental designs.

The overall risk reduction for breast cancer for individuals that are habitually physically active (at or above Canada's guidelines for physical activity) is thought to approximate 20-40% [31,105]. In our analyses, we found very similar findings. When comparing the most active group versus the least active group we found a mean (and median) risk reduction of 20% across all studies. *The level of evidence would be considered to be Level 2A*. Generally, the articles were of high quality with a mean Downs and Black score of 13 (median = 13, range = 9-14).

A dose-dependency of this relationship is also generally present in the majority of the studies. For instance, greater than 50% studies revealed a dose-response relationship in one or more measures of occupational and/or leisure-time physical activity and the risk for breast cancer. Moreover, the majority of studies demonstrated the greatest risk reduction at the highest activity level. With respect to the minimal and optimal volume of exercise required, Lee [105] stated that 30-60 min/day of moderate-to-vigorous physical activity is required to decrease the risk for breast cancer. This belief is strongly supported by the literature. However, others have shown significant risk reductions at lower exercise volumes. For instance, Rockhill et al. [106] showed significant reductions (12% or greater) in the risk for breast cancer in women who accumulated at least 1 hr of moderate or vigorous physical activity per week. Similarly, Sesso et al. (1998) revealed that there was an 8% reduction in the risk for breast cancer with a relatively small energy expenditure of 500-999 kcal/wk. Further risk reductions were observed with higher energy expenditures (= 1000 kcal/wk = 51% reduction in the risk). As discussed above, Monninkhof et al. revealed a 6% decrease in breast cancer risk for each additional hour of physical activity per week [104]. Taken as a whole, it would therefore appear that Canada's guidelines for physical activity are more than appropriate for reducing the risk for breast cancer. Further research however is required to determine the minimal volume of exercise that is effective in the primary prevention of breast cancer.

#### Implications

There is a preponderance of data linking physical inactivity to site-specific cancers, particularly of the breast and colon [31,104-109]. The protective effects of physical activity also appear with other forms of cancer (such as endometrial cancer) [110]. In an important review of the literature Lee revealed that physically active women have a 20-30% lower risk of breast cancer, and physically active men and women have a 30-40% lower risk of colon cancer [105]. A more recent systematic review of the literature revealed a 20-80% lower risk of breast cancer in post-menopausal women [104], with a weaker association in pre-menopausal women. Considering data from both pre- and post-menopausal women the authors demonstrated that physically active individuals had a 15-20% lower risk of breast cancer. Monninkhof et al. also reported a 6% lower risk of breast cancer for each additional hour of physical activity per week [104]. This level of risk reduction was also supported by the U.S. Department of Health and Human Services during its recent evaluation of the literature [31].

Our current reviews of the literature support previous work in the field including the finding of a dose-response relationship between physical activity and cancers of the breast and colon [104,105,109]. It would appear that 30-60 min/day of moderate-to-vigorous physical activity is associated with a lower risk of breast and colon cancer.

*Recommendation #5*

*For a reduced risk for site specific cancers (such as colon cancer and breast cancer), it is recommended that individuals should participate in 30 min or more of moderate to vigorous exercise on most days of the week. [Level 2, Grade A]*

### Primary Prevention of Type 2 Diabetes

**Figure 9:**
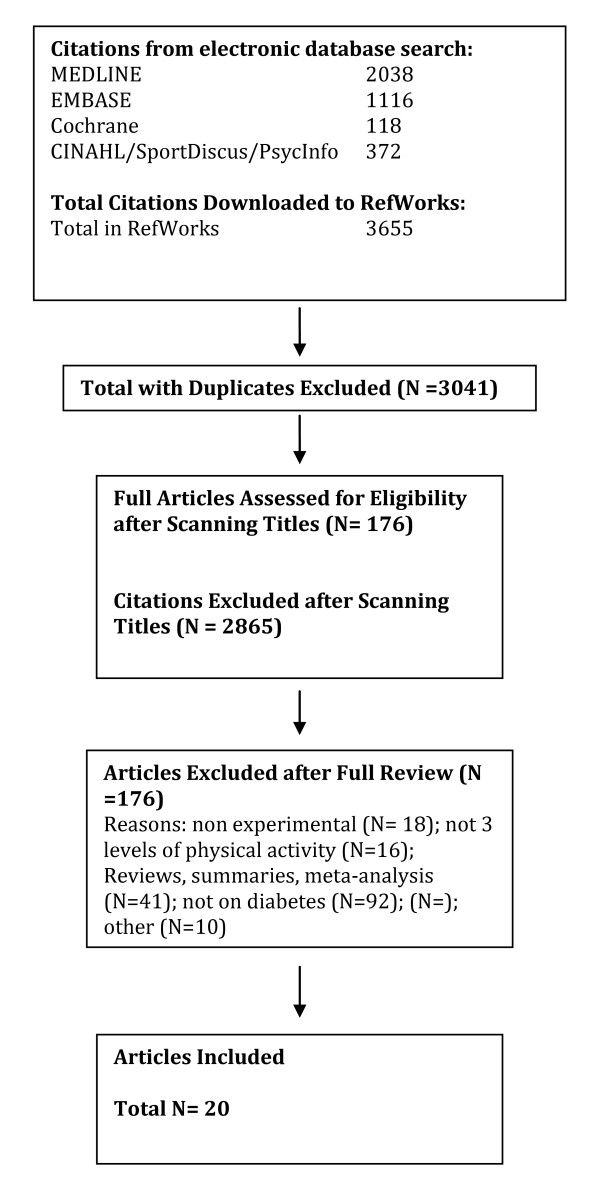
**Results of the Literature Search for Diabetes**.

In comparison to other chronic conditions, there is relatively limited literature examining the relationship between multiple levels of physical activity/fitness and the incidence of type 2 diabetes. All of the literature examining the dose-response (for at least three levels of physical activity/fitness) involved prospective cohort analyses. A total of 3655 citations were identified during the electronic database search (Figure 9). Of these citations, 2038 were identified in MEDLINE, 1116 in EMBASE, 118 in Cochrane, and 372 in the CINAHL/SportDiscus/PsychInfo search. A total of 614 duplicates were found, leaving a total of 3041 unique citations. A total of 2865 articles were excluded after scanning, leaving a total of 176 articles for full review. From these articles 156 were excluded after full review leaving 20 articles for inclusion in the systematic review of the literature regarding the relationship between physical activity and type 2 diabetes. The reasons for exclusion included non-experimental/weak design (N = 18), three levels of physical activity not reported (N = 16), reviews, summaries, or meta-analyses (N = 41), not related to type 2 diabetes (N = 71), and other (N = 10).

As shown in Table 17, 20 investigations examined the dose-response (i.e., three or more levels) relationship between physical activity and the incidence of type 2 diabetes. This involved a total of 624,952 subjects, averaging 32,892 subjects per study (range 1,543-87,907). There were a total of 19,325 cases of type 2 diabetes (ranging per study from 78-4,030). The total length of follow-up averaged 9.3 yr (ranging from 3 -16.8 yr). The articles were published over a 16 yr period ranging from 1991 to 2007.

**Table 17:** The relationship between physical activity and the development of type 2 diabetes.

Publication Country Study Design Quality Score	Objective	Population	Methods	Outcome	Comments and Conclusions
Haapanen et al 1997 [77]	To examine the association of PA and the risk of CHD, hypertension and T2D.	• n = 1,340 men, 1,500 women	10 yr follow-up	Number of cases: 118	LTPA has a preventive effect on T2D.
		• Age: 35-63 yr	PA assessment: Self-reported	Age-adjusted RR (95% CI), men	
Finland			LTPA (kcal/wk), divided into groups	• G1 = 1.54 (0.83-2.84)	
				• G2 = 1.21 (0.63-2.31)	
Prospective cohort				• G3 = 1.00 (referent)	
				*p *= 0.374	
			Men		
D & B score = 14			G1 = 0-1100	Age-adjusted RR (95% CI), women	
			G2 = 1101-1900	• G1 = 2.64 (1.28-5.44)	
			G3 = >1900	• G2 = 1.17 (0.50-2.70)	
				• G3 = 1.00 (referent)	
			Women (kcal/wk)	*p *< 0.006	
			G1 = 0-900		
			G2 = 901-1500		
			G3 = >1500		
			Cox proportional HR		

Hu et al 2003 [111]	To examine the relationship between sedentary behaviours (particularly prolonged television watching) and risk of obesity and T2D in women.	• n = 68,497 (diabetes specific analyses)	6 yr follow-up	Number of cases: 1515	Sedentary behaviours (especially television watching) are associated with an increased risk for obesity and T2D.
USA		• n = 50,277 (obesity specific analyses)	PA assessment: Self-reported PA and sedentary behaviour	Each 2-h/d increment in TV watching was associated with a 23% (95% CI, 17%-30%) increase in obesity and a 14% (95% CI, 5%- 23%) increase in risk of T2D	
Prospective cohort					
		• Age: 30-55 yr	Outcome measure: onset of obesity and T2D	Each 2-h/d increment in sitting at work was associated with a 5% (95% CI, 0%-10%) increase in obesity and a 7% (95% CI, 0%- 16%) increase in T2D	Light to moderate PA was associated with a significantly lower risk for obesity and T2D.
D & B score = 13		• Sex: Women	Multivariate analyses adjusting for age, smoking, dietary factors, and other covariates	Standing or walking around at home (2 h/d) was associated with a 9% (95% CI, 6%-12%) reduction in obesity and a 12% (95% CI, 7%- 16%) reduction in T2D	
		• Characteristics:		Each 1 hour per day of brisk walking was associated with a 24% (95% CI, 19%-29%) reduction in obesity and a 34% (95% CI, 27%- 41%) reduction in T2D	
		Free of T2D, CVD, or cancer at baseline			
		• Nurses' Health Study			

Manson et al 1992 [112]	To examine the association between regular exercise and the subsequent development of T2D.	• n = 21,271	5 yr follow-up	Number of cases: 285	Exercise appears to reduce the development of T2D even after adjusting for BMI.
		• Sex: Men	PA assessment: Questionnaire Fpr VPA (enough to develop sweat)		
		• Age: 40-84 yr		The age-adjusted incidence of T2D:	
USA		• Characteristics:		• 369 cases per 100,000 person- years in men who engaged in VPA less than once weekly • 214 cases per 100,000 person- years in those exercising at least five times per week (p trend < 0.001)	
		Free of diagnosed diabetes, CVD and cancer at baseline			
Prospective cohort					
D & B score = 14			Exercise frequency (times/wk)		
			G1 = < Weekly		
			G2 = At least weekly		
				Age-adjusted RR (95% CI) by exercise frequency	
			Times per week		
			G1 = 0	• G1 = 1.00 (referent)	
			G2 = 1	• G2 = 0.64 (0.51- 0.82)	
			G3 = 2-4		
			G4 = >5	Age-adjusted RR (95% CI) by exercise frequency	
				• G1 = 1.00 (referent)	
			Outcome measure: Incidence T2D	• G2 = 0.77 (0.55-1.07)	
				• G3 = 0.62 (0.46-0.82)	
				• G4 = 0.58 (0.40-0.84)	
				Age- and BMI-adjusted RR (95%	
				CI) by exercise frequency	
				• G1 = 1.00 (referent)	
				• G2 = 0.71 (0.56- 0.91)	
				Age- and BMI-adjusted RR (95% CI) by exercise frequency	
				• G1 = 1.00 (referent)	
				• G2 = 0.78 (0.56-1.09)	
				• G3 = 0.68 (0.51-0.90)	
				• G4 = 0.71 (0.49-1.03)	

Hu et al. 2001[114]	To examine the relationship between dietary and lifestyle factors in relation to the risk for T2D.	• n = 84,941	16 yr follow-up	Number of cases: 3300	The majority of T2D could be prevented through healthy living.
		• Sex: Women			
		• Age: 40-75 yr	PA assessment: Questionnaire For PA (h/wk), divided into groups	Multivariate-adjusted RR (95%)	
USA		• Characteristics: participants had no history of diabetes, CVD, or cancer.		• Q1 = 1.00 (referent)	
				• Q2 = 0.89 (0.77-1.02)	
Retrospective cohort				• Q3 = 0.87 (0.75-1.00)	
			Q1 = <0.5	• Q4 = 0.83 (0.71-0.96)	
			Q2 = 0.5--1.9	• Q5 = 0.71 (0.56--0.90)	
D & B score = 13		Nurses' Health Study	Q3 = 2.0--3.9		
			Q4 = 4.0--6.9		
			Q5 = ≥7.0		
			Outcome measure: Incidence of T2D		
			Cox regression		

Sato et al 2007 [116]	To examine the relationship between walking to work and the development of T2D.	• n = 8,576	4 yr follow-up	Number of cases: 878	The duration of a walk to work is an independent predictor of the risk for T2D.

		• Sex: Men			
		• Age: 40--55 yr	PA assessment: For time spent walking to work, divided into tertiles	OR (95% CI)	
Japan		• Kansai Healthcare Study		• T1 = 1.00 (referent)	
				• T2 = 0.86 (0.70-1.06)	
Prospective cohort			T1 = 0-10 min	• T3 = 0.73 (0.58-0.92)	
			T2 = 11-20 min	Significant difference was seen between ≤ 10 min and ≤ 20 min only (p = 0.007)	
			T3 = ≥20 min		
D & B score = 14					
			Outcome measure: Incidence of T2D		

Hu G et al 2003 [117]	To examine the relationship of OPA, commuting and LTPA with the incidence of T2D.	• n = 14,290	PA assessment: Questionnaire For OPA, LTPA and commuting PA	Multivariate adjusted HR (95% Cl) for OPA, men	Moderate and high OPA, commuting PA or LTPA significantly reduces risk of T2D in middle aged adults.
		• Sex: Men and women			
				• G1 = 1.00 (referent)	
Finland		• Age: 35-64 yr		• G2 = 0.67 (0.44-1.01)	
		• Characteristic:	OPA	• G3 = 0.73 (0.52-1.02)	
Prospective cohort		Asymptomatic for stroke, CHD, or diabetes at baseline.	G1 = Light (sitting)		
			G2 = Moderate (standing, walking)	Multivariate adjusted HR (95% Cl) for OPA, women	
D & B score = 12			G3 = Active (walking, lifting)	• G1 = 1.00 (referent)	
				• G2 = 0.72 (0.46-1.12)	
				• G3 = 0.78 (0.52-1.18)	
			Commuting PA (min/d)		
			G1 = None	Multivariate adjusted HR (95% Cl) for OPA, men and women	
			G2 = 1-29		
			G3 = ≥ 30		
				G1 = 1.00 (referent)	
				G2 = 0.70 (0.52-0.96)	
			LTPA		
				G3 = 0.74 (0.57-0.95)	
			• G1 = Low (inactive)		
			• G2 = Moderate (walking, cycling >4 hr/wk)		
				Multivariate adjusted HR (95% Cl) for commuting PA, men	
			• G3 = High (running, jogging >3 hr/wk)		
				• G1 = 1.00 (referent)	
				• G2 = 1.00 (0.71-1.42)	
			Outcome measure: incidence of T2D	• G3 = 0.75 (0.46-1.23)	
				Multivariate adjusted HR (95% Cl) for commuting PA, women	
			Cox proportional HR		
				• G1 = 1.00 (referent)	
				• G2 = 0.94 (0.63-1.42)	
				• G3 = 0.57 (0.34-0.96)	
				Multivariate adjusted HR (95% Cl) for commuting PA, men and women	
				• G1 = 1.00 (referent)	
				• G2 = 0.96 (0.74-1.25)	
				• G3 = 0.64 (0.45-0.92)	
				Multivariate adjusted HR (95% Cl) for LTPA, men	
				• G1 = 1.00 (referent)	
				• G2 = 0.78 (0.57-1.06)	
				• G3 = 0.84 (0.52-1.37)	
				Multivariate adjusted HR (95% Cl) for LTPA, women	
				• G1 = 1.00 (referent)	
				• G2 = 0.81 (0.58-1.15)	
				• G3 = 0.85 (0.43 -1.66)	
				Multivariate adjusted HR (95% Cl) for LTPA, men and women	
				• G1 = 1.00 (referent)	
				• G2 = 0.81 (0.64-1.20)	
				• G3 = 0.84 (0.57-1.25)	

Hsia et al 2005 [118] USA	To evaluate the relationship between PA and the incidence of T2D in a large, diverse group of older women.	• n = 87,907	PA assessment: Questionnaire for frequency and duration of 4 walking speeds and 3 other activities classified by intensity (light, moderate, strenuous)	Number of cases: 2,271	There is a strong inverse relationship between PA and T2D. There is a stronger relationship between PA and T2D in Caucasian women than in minority women. This may be explained by less precise risk estimates in minority women.
		• Sex: Women			
		• Age: White 63.8 ± 7.3, African American 61.9 ± 7.3, Hispanic 60.5 ± 7.1, Asian/Pacific Islander 63.7 ± 7.6, American Indian 61.5 ± 8.0		Multivariate adjusted HR (95% CI) by walking, Caucasian	
				• Q1 = 1.00 (referent)	
Prospective cohort				• Q2 = 0.85 (0.74-0.87)	
				• Q3 = 0.87 (0.75-1.01)	
				• Q4 = 0.75 (0.64-0.89)	
D & B score = 11			Q1 = Low	• Q5 = 0.74, (0.62-0.89)	
			Q2 =	Trend *p *< 0.001	
			Q3 =		
			Q4 =	Multivariate adjusted HR (95% CI) by TPA, Caucasian	
			Q5 = High		
		• Ethnicity: White n = 74,240; African American n = 6,465; Hispanic n = 3,231; Asian/Pacific Islander 2,445; American Indian n = 327		• Q1 = 1.00 (referent)	
			Cox proportional HR		
				• Q2 = 0.88 (0.76- 1.01)	
				• Q3 = 0.74 (0.64- 0.87)	
				• Q4 = 0.80 (0.68- 0.94)	
				• Q5 = 0.67 (0.56- 0.81) Trend *p *= 0.002	
		• Characteristics: participants had no history of diabetes, were not on any antidiabetic medications			
		• Women's Health Initiative			

Wannamethee et al 2000 [120]	To examine the role of components of the insulin resistance syndrome in the relationship between PA and the incidence of T2D and CHD.	• n = 5,159	16.8 yr follow-up	Number of cases: 196	The relationship between PA and T2D appears to be mediated by serum insulin and components of the insulin resistance syndrome. However, these factors do not appear to explain the inverse relationship between PA and T2D.
		• Sex: Men			
		• Age: 40-59 yr	PA assessment: Questionnaire for TPA Physical activity groups were identified and scored:	Multivariate adjusted RR (95% CI)	
England, Wales and Scotland		• Characteristics: No history of heart disease, diabetes or stroke		Q1 = 1.00 (referent)	
				Q2 = 0.66 (0.42-1.02)	
				Q3 = 0.65 (0.41-1.03)	
Prospective cohort				Q4 = 0.48 (0.28-0.83)	
			Q1 = None	Q5 = 0.46 (0.27-0.79)	
			Q2 = Occasional	*p *< 0.005	
D & B score = 14			Q3 = Light		
			Q4 = Moderate		
			Q5 = Moderately vigorous/vigorous		MPA (sporting activity once a week or frequent lighter- intensity activities such as walking, gardening, do-it yourself projects) are sufficient to produce a significant reduction in risk of both CHD and T2D.
			The men were classified according to current smoking status, alcohol consumption, and social class		
			Cox proportional HR		

Manson et al 1991 [121]	To examine the association between regular VPA and the incidence of T2D.	• n = 87,253	8 yr follow-up	Number of cases: 1303 Women who engage in VPA at least once per week had reduced adjusted RR of T2D RR = 0.66 (0.6- 0.75)	PA is promising in the primary prevention of T2D.
		• Sex: Women			
		• Age: 34-59 yr	PA assessment:		
USA		• Characteristics: Free of diagnosed diabetes, cardiovascular disease and cancer	Questionnaire		
			Frequency of weekly exercise (0-+4)		
Prospective cohort					
				The reduction in risk remained significant after adjustment for BMI RR = 0.84 (0.75-0.95)	
D & B score = 13			Analysis also restricted to the first 2 yr after the assessment of PA level and to symptomatic diabetes		
				When analysis was restricted to the first 2 years after ascertainment of PA level and to symptomatic disease as the outcome, the age- adjusted RR of those who exercised was 0.50, and age and body-mass index adjusted RR was 0.69 (0.48-1.0)	
			Multivariate adjustments for age, body-mass index, family history of diabetes, and other variables did not alter the reduced risk found with exercise		
			Multivariate analysis	Family history of diabetes did not modify the effect of exercise, and risk reduction with exercise was evident among both obese and non-obese women	

Helmrich et al 1994 [122]	To examine the relationship between PA and the development of T2D.	• n = 5,990	98,524 man-years of follow-up (1962-1976)	Number of cases: 202	Increased PA is effective in preventing T2D.
		• Sex: Men			
		• Age: 39-68 yr		RR (95% CI) by blocks walked per day	
USA		• Characteristics: healthy, asymptomatic	PA assessment: Questionnaire for LTPA (walking, stair climbing, sports etc; kcal/wk) Blocks walked/day		The protective benefit is especially pronounced in those individuals who have the highest risk of disease.
				• T1 = 1.00 (referent)	
Further review of the data reported by Helmich et al. 1991				• T2 = 1.30	
		University of Pennsylvania Alumni Health Study		• T3 = 0.92	
				*p *= 0.80	
			LTPA (kcal/wk) kcal were assigned to each activity and added together	LTPA was inversely related to the development of T2D	
Prospective cohort					
				Same findings to that reported in 1991	
D & B score = 14			Lowest < 500		
			Highest ≥ 3500		
			Blocks walked/day		
			T1 = <5		
			T2 = 5-14		
			T3 = ≥15		
			Cox proportional HR		

Helmrich et al 1991 [123]	To examine the Relationship between PA and the Subsequent development of T2D.	• n = 5,990	98,524 man-years of follow-up (1962-1976)	Number of cases: 202	Increased PA is effective in preventing T2D.
		• Sex: Men			
		• Age: 39-68 yr		LTPA was inversely related to the development of type 2 diabetes	
USA		• Characteristics: healthy, asymptomatic	PA assessment: Questionnaire for LTPA kcal/wk: stairs climbed/day and blocks walked/day, divided into groups		The protective benefit is especially pronounced in those individuals who have the highest risk of disease.
Prospective cohort				RR (95% CI) by sports played	
		• University of Pennsylvania Alumni Health Study		• G1 = 1.00 (referent)	
				• G2 = 0.90	
D & B score = 13				• G3 = 0.69	
				• G4 = 0.65	
			All activities LTPA	Trend *p *= 0.02	
			Q1 = <500		
			Q2 = 500-999	RR (95% CI) by Flights of stairs climbed/day	
			Q3 = 1000-1499		
			Q4 = 1500-1999	• T1 = <5 = 1.00 (referent)	
			Q5 = 2000-2499	• T2 = 0.78	
			Q6 = 2500-2999		
				• T3 = 0.75	
			Q7 = 3000-3499		
				Trend *p *= 0.07	
			Q8 = ≥ 3500		
				RR (95% CI) by Blocks walked/day	
			Sports played		
				• T1 = 1.00 (referent0	
			G1 = None		
				• T2 = 1.31	
			G2 = Moderate		
			G3 = Vigorous	• T3 = 0.93 Trend *p *= 0.80	
			G4 = Moderate and Vigorous		
				Age adjusted RR (95% CI) by all activities	
			Stairs climbed per day		
			T1 = <5	• Q1 = 1.00 (referent)	
			T2 = 5-14	• Q2 = 0.94	
			T3 = ≥ 15	• Q3 = 0.79	
				• Q4 = 0.78	
			Blocks walked per day	• Q5 = 0.68	
			T1 = <5	• Q6 = 0.90	
			T2 = 5-14	• Q7 = 0.86	
			T3 = ≥ 15	• Q8 = 0.52	
				*p *= 0.01 for trend	
			Cox proportional HR		
				Age adjusted RR (95% CI) by all activities except vigorous sports	
				• Q1 = 1.00 (referent)	
				• Q2 = 0.97	
				• Q3 = 0.87	
				• Q4 = 0.92	
				• Q5 = 0.75	
				• Q6 = 1.29	
				• Q7 = 1.03	
				• Q8 = 0.48	
				Trend *p *= 0.07	
				Age adjusted RR (95% CI) by vigorous sports only	
				• Q1 = 1.00 (referent)	
				• Q2 = 0.69	
				• Q3 = N/A	
				• Q4 = 0.53	
				• Q5 = 0.86	
				• Q6 = 0.56	
				• Q7 = 0.40	
				• Q8 = 0.46	
				Trend *p *= 0.05	

Wei et al 1999 [124]	To determine whether PF is associated with risk for impaired fasting glucose and T2D.	• n = 8,633	6 yr follow-up	Number of cases: 149	High PF is associated with a reduced risk for impaired fasting glucose and T2D.
USA		• Sex: Men			
		• Age: 43.5 yr	PF assessment: Maximal treadmill exercise test (METs), divided into 3 groups	593 patients developed impaired fasting glucose	
		• Characteristics: Non-diabetic men		OR (95% CI) for developing glucose intolerance	
Prospective cohort			T1 = Low	• T1 = 1.9 (1.5--2.4)	
			T2 = Moderate	• T2 = 1.5 (1.2--1.8)	
			T3 = High	• T3 = 1.00 (referent)	
D & B score = 12			Outcome measure: Incidence of impaired fasting glucose and T2D	OR (95% CI) for developing T2D	
				• T1 = 3.7 (2.4 --5.8)	
				• T2 = 1.7 (1.1--2.7)	
				• T3 = 1.00 (referent)	
			Statistics: GLM		

Katzmarzyk et al 2007 [126]	To examine the relationships among adiposity, PA, PF and the development of T2D in a diverse sample of Canadians.	• n = 1,543 (709 men and 834 women)	6 yr follow-up	Number of cases: 78 (37 in men, 41 in women)	Adiposity and PF are important predictors of the development of T2D.
Canada		• Sex: Men and women	PF assessment: Questionnaire	PA was associated with 23% lower odds of developing diabetes and maximal METs was also associated with significantly lower odds of developing diabetes (OR = 0.28)	
Prospective cohort		• Age: 36.8 - 37.5	PA assessment: LTPA Questionnaire		
D & B score = 13		• Characteristics: Free of diabetes at baseline			
		• Canadian Physical Activity Longitudinal Study			

Burchfiel et al 1995 [345]	To examine the relationship between PA and T2D.	• n = 6,815	6 yr follow-up	Number of cases: 391	PA is associated inversely and independently with incident T2D.
USA		• Sex: Men (Japanese- American)	PA assessment: Questionnaire PA index (based on intensity and duration of activity)	The age-adjusted 6-year cumulative incidence of diabetes decreased progressively with increasing quintile of physical activity from 73.8 to 34.3 per 1,000 (p < 0.0001, trend)	
		• Age: 45-68 yr	Levels of activity:		
Prospective cohort		• Characteristics: Free of diabetes at entry	Q1 = Basal - Sleeping reclining		
D & B score = 13		• The Honolulu Heart Program	Q2 = Sedentary		
			Q3 = Slight - Casual walking		
			Q4 = Moderate -- Gardening		
			Q5 = Heavy - Lifting, shoveling		
			Outcome measure: Self-reported T2D (clinically recognized)		

Dziura et al 2004 [346]	To determine the prospective relation between reports of habitual PA, 3-year change in body weight, and the subsequent risk of T2D in an older cohort.	• n = 2,135	PA assessment: Questionnaire for 4 types of activities (walking, gardening/housework, physical exercises, active sports or swimming) and frequency of participation measured with a PA score:	118 cases of T2D	Observation of an inverse relationship between reported PA and rate of T2DM.
USA		• Sex: Men and women		Incident density of T2D = 6.6/1000 person years	
		• Age: ≥ 65 yr			
Prospective cohort		• Ethnicity: 83% White, 15% African American, 2% Non-white		Diabetes (n = 118) PA score: 2.17 ± 1.7 'Some' PA: 78%	Subjects reporting some PA at baseline experienced a rate of T2D over 50% lower relative to those reporting no PA.
D & B score = 12		• Characteristics: Healthy asymptomatic	Never (score 0) Sometimes (score 1) Often (score 2)	Non-Diabetes (n = 2017) PA score: 2.34 ± 1.7 'Some' PA: 84%	
			Pearson product moment correlation coefficient and Cox proportional HR		

Hu et al. 1999 [347]	To quantify the dose-response relationship between total PA and incidence of T2D in women.	• n = 70,102	8 yr of follow-up	Number of cases: 1419	Increased PA is associated with substantial reduction in risk of T2D including PA of moderate intensity and duration.
USA		• Sex: Women	PA assessment: Questionnaire for TPA (MET hr/wk) and VPA (6 METs)	Multivariate-adjusted RR (95% CI) of by TPA	
		• Age: 40-65 yr		• Q1 = 1.0 (referent)	
Prospective cohort	To examine the health benefits of walking in comparison to more vigorous activity.	• Characteristics: participants had no history of diabetes, CVD, or cancer	TPA (MET hr/wk)	• Q2 = 0.77 (0.66-0.90)	
D & B score = 12		Nurses' Health Study	Q1 = 0-2.0	• Q3 = 0.75 (0.65-0.88)	
			Q2 = 2.1-4.6	• Q4 = 0.62 (0.52-0.73)	
			Q3 = 4.7-10.4	• Q5 = 0.54 (0.45-0.64)	
			Q4 = 10.5-21.7	Trend *p *< 0.001	
			• Q5 = ≥ 21.8		
			MET score	Multivariate-adjusted RR (95% CI) among women who did not perform vigorous exercise (MET's):	
			Q1 = ≤ 0.5	• Q1 = 1.0 (referent)	
			Q2 = 0.6-2.0	• Q2 = 0.91 (0.75-1.09)	
			Q3 = 2.1-3.8	• Q3 = 0.73 (0.59-0.90)	
			Q4 = 3.9-9.9	• Q4 = 0.69 (0.56-0.86)	
			Q5 = ≥ 10.0	• Q5 = 0.58 (0.46-0.73)	
			Outcome measures:	Trend p < 0.001	
			Incidence of T2D		

Hu et al 2001 [348]	To examine the role of prolonged television watching on the risk for T2D.	• n = 37,918	10 year follow-up	Number of cases: 1058	Increasing PA is associated with a significant reduction in risk for T2D, whereas a sedentary lifestyle indicated by prolonged TV watching is related directly to increased risk.
USA		• Sex: Men			
		• Age: 40-75 yr	PA assessment: Questionnaire for PA (MET hr/wk) and TV watching (h/wk), each divided into quintiles	Multivariate-adjusted RR (95% CI) by PA	
Prospective cohort		• Characteristics: participants had no history of diabetes, CVD, or cancer	Q1 = 0-5.9	• Q1 = 1.00 (referent)	
D & B score = 11		• Health Professionals' Follow-up Study	Q2 = 6.0-13.7	• Q2 = 0.78 (0.66 -- 0.93)	
			Q3 = 13.8-24.2	• Q3 = 0.65 (0.54 -- 0.78)	
			Q4 = 24.3-40.8	• Q4 = 0.58 (0.48 -- 0.70)	
			Q5 = ≥ 40.9	• Q5 = 0.51 (0.41 -- 0.63)	
				Trend p < 0.001	
			Time spent watching television per week (h/wk)	Multivariate-adjusted RR (95% CI) by TV time	
			Q1 = 0-1	• Q1 = 1.00 (referent)	
			Q2 = 2-10	• Q2 = 1.66 (1.15 - 2.39)	
			Q3 = 11-20	• Q3 = 1.64 (1.12 - 2.41)	
			Q4 = 21-40	• Q4 = 2.16 (1.45 - 3.22)	
			Q5 = >40	• Q5 = 2.87 (1.46 - 5.65)	
				Trend p < 0.001	

Rana et al 2007 [349]	To examine the individual and combined association of obesity and physical inactivity with the incidence of T2D.	• n = 68,907	16 yr follow-up	Number of cases: 4,030	This study found that obesity and physical inactivity independently contributed to the development of T2D.
USA		• Sex: Women			
Prospective cohort		• Age: 30-55 years age range in 1976 (note: 1986 was the baseline year for the study)	PA assessment: Questionnaire for average amount of time/week MET hours per week spent in MVPA (≥ 3 METs), divided into quintiles	Multivariate-adjusted RR (95% CI) by MVPA:	The benefits of PA were not limited to lean women; among those who were overweight and obese, physically active women tended tobe at lower risk for T2D than sedentary women.
D & B score = 12		• Characteristics: No history of diabetes, CVD or cancer	Q1 = <2.1	• Q1 = 2.37 (2.15--2.16)	
		• Nurses' Health Study	Q2 = 2.1-4.6	• Q2 = 1.92 (1.73--2.13)	
			Q3 = 4.7-10.4	• Q3 = 1.48 (1.34--1.64)	
			Q4 = 10.5-21.7	• Q4 = 1.40 (1.26--1.55)	
			Q5 = ≥ 21.8	• Q5 = 1.00 (referent)	
				Trend *p *< 0.001	
			Cox proportional HR		

Sawada et al 2003 [350]	To examine the association between PF and the incidence of T2D.	• n = 4,747	14 yr follow-up	Number of cases: 280	Low PF is associated with a higher risk for the development of T2D.
Japan		• Sex: Men			
		• Age: 20-40 yr	PF assessment: Maximal aerobic power estimate ml/kg/min using a submaximal cycle ergometer test, divided into quartiles	Age-adjusted RR (95% CI)	
Prospective cohort		• Characteristics: Free of diabetes, CVD, hypertensin, tuberculosis, and gastrointestinal disease at baseline	Q1 = 32.4 ± 3.1	• Q1 = 1.00 (referent)	
D & B score = 13			Q2 = 38.0 ± 2.5	• Q2 = 0.56 (0.42-- 0.75)	
			Q3 = 42.4 ± 3.0	• Q3 = 0.35 (0.25-- 0.50)	
			Q4 = 51.1 ± 6.2	• Q4 = 0.25 (0.17-- 0.37)	
				Trend *p *< 0.001	
			Outcome measure: Incidence of T2D	Multivariate adjusted RR (95% CI)	
				• Q1 = 1.00 (referent)	
				• Q2 = 0.78 (0.58--1.05)	
				• Q3 = 0.63 (0.45--0.89)	
				• Q4 = 0.56 (0.37--0.84)	
				Trend *p *= 0.001	
			Cox proportional HR		

Weinstein et al 2004 [351]	To examine the relative contributions and joint association of PA and BMI with T2D.	• n = 37,878	6.9 year follow up	Number of cases: 1,361	Although BMI and physical inactivity are independent predictors of incident diabetes, the magnitude of the association with BMI was greater than with PA in combined analyses. These findings underscore the critical importance of adiposity as a determinant of T2D.
USA		• Sex: Women	PA assessment: Questionnaire for walking per week (h/wk) and TPA (kcal/wk), divided into groups and quartiles respectively	Multivariate-adjusted HR (95% CI) by time spent walking	
		• Age: 45+ years		• G1 = 1.00 (referent)	
Prospective cohort		• Health care professionals		• G2 = 0.95 (0.82-1.10)	
D & B score = 12		• Characteristics: No history of CVD, cancer or diabetes		• G3 = 0.87 (0.73 -1.02)	
				• G4 = 0.66 (0.54-0.81)	
				• G5 = 0.89 (0.73-1.09)	
			Walking per week (h/wk)	Trend *p *= 0.004	
			G1 = no walking	Multivariate-adjusted HR (95% CI) by TPA	
			G2 = <1	• Q1 = 1.00 (referent)	
			G3 = 1-1.5	• Q2 = 0.91 (0.79-1.06)	
			G4 = 2-3	• Q3 = 0.86 (0.74-1.01)	
			G5 = ≥ 4	• Q4 = 0.82 (0.70-0.97)	
			TPA (kcal/wk)	Trend *p *= 0.01	
			Q1 < 200		
			Q2 = 200-599		
			Q3 = 600-1,499		
			Q4 ≥ 1500		
			Cox proportional HR		

D & B score, Downs and Black quality score; YR, years; PA, physical activity; CHD, coronary heart disease; T2D, type 2 diabetes; LTPA, leisure-time physical activity; g, group; kcal/wk, kilocalories per week; HR, hazard ratio; RR, risk ratio; OR, odds ratio; 95% CI, confidence interval; CVD, cardiovascular disease; OPA, occupational physical activity; PF, physical fitness; MET, metabolic equivalent; MET/wk, metabolic equivalent per week.

Of these studies 100% revealed an inverse relationship between type 2 diabetes and levels of physical activity or fitness. When comparing the most active/fit group versus the least active/fit group we found an average risk reduction of 42% (median = 44%). Therefore in our analyses the most physically active/fit had a 42% lower risk of developing type 2 diabetes. The majority (84%) of these studies revealed incremental reductions in the risk for type 2 diabetes with increasing activity/fitness levels. Therefore, the health benefits with respect to type 2 diabetes prevention appear to continue across the physical activity/fitness continuum. Similar to other clinical conditions, the dose-response relationship is such that small changes in activity levels yield marked reductions in the risk for type 2 diabetes. The health benefits of exercise appear to be particularly prevalent in individuals at high risk for developing type 2 diabetes (e.g., those with a high body mass index, the metabolic syndrome, a history of hypertension and/or a family history of type 2 diabetes). *The level of evidence relating physical activity to the primary prevention of type 2 diabetes would be considered to be Level 2A*. The quality of the investigations was generally high with a mean (and median) Downs and Black score of 13 (range 11-14).

As with other conditions is it difficult to separate the effects of volume and intensity of exercise. However, small changes in activity levels clearly can have a large effect on the risk for and incidence of type 2 diabetes. For instance, Hu and coworkers [111] revealed that nurses (n = 68,497) who engaged in 1 hr/day of brisk walking had 24% less obesity and 34% less type 2 diabetes (over a 6-year follow-up). These authors estimated that approximately 30% of new cases of obesity and 43% of new cases of type 2 diabetes could be prevented by adopting an active lifestyle including less than 10 hr/wk of television watching and ≥ 30 min/d of brisk walking. Similarly, over a 5-year period, male physicians who exercised vigorously at least once weekly had a 29% lower incidence of type 2 diabetes than individuals who did not exercise regularly [112]. These authors also revealed that physical activity that was sufficient to cause sweating was associated with a lower incidence of type 2 diabetes. Other research has also demonstrated that moderate-to-vigorous physical activity (≥ 5.5 METs for at least 40 min per week) and/or aerobic fitness levels above 31 mL·kg^-1^·min^-1 ^are associated with a lower risk of type 2 diabetes in middle-aged men [113] with the effect being the greatest in high-risk individuals. Therefore, it would appear that Canada's recommendations for physical activity are sufficient to reduce the risk for type 2 diabetes.

In 2001, Hu et al. [114] reported very interesting and compelling research regarding the role of lifestyle factors in the development of type 2 diabetes. Using data from the Nurses' Health Study, they defined a low-risk group according to five lifestyle factors including BMI, a healthy diet, the participation in moderate-to-vigorous physical activity for at least 30 min per day, no current smoking, and the consumption of an average of at least one-half serving of an alcoholic beverage per day. They revealed that the women in the low risk group had a RR for type 2 diabetes of only 0.09 (CI 0.05-0.17) in comparison to the rest of the cohort. They also found that 91% of the cases of type 2 diabetes in this cohort (CI 83-95%) could be attributed to the five lifestyle factors. This research provided compelling evidence that the majority of type 2 diabetes could be prevented through healthy living [115].

As reviewed in Table 17 there is evidence that leisure-time, occupational and commuting-related leisure time activities significantly reduce the risk for the development of type 2 diabetes. For instance, a recent study by Sato and colleagues [116] revealed that the walking distance to work was directly related to the incidence of type 2 diabetes in 8,576 Japanese men followed for 4 years. The risk reduction was approximately 27% in the participants who walked to work for ≥21 min compared to those who did so for ≥10 min. These findings are similar to that found by Hu et al. who reported that moderate occupational, commuting and leisure-time physical activities all had a significant inverse relationship to risk in middle-aged men and women [117].

Although ethnicity is often not reported in the current research, the studies examined in our systematic review came from a variety of countries and regions. Data was obtained from studies from the USA, Canada, UK, Japan, and Finland. For instance, Hsia et al. (2005) conducted a prospective 5-year study of 87,907 post-menopausal women, finding a strong graded inverse relationship between physical activity and type 2 diabetes. The relationship was stronger in "Caucasian" than in minority (African-American, Hispanic or Asian) women. The authors postulated this finding might reflect less precise risk assessments in minority women [118]. As we have outlined previously, further research is clearly warranted that examines the relationship between physical activity and type 2 diabetes in persons of different ethnicities. Moreover, further research is needed to determine the effects of socio-economic status on the observed relationships. Nonetheless, the research is compelling, habitual physical activity appears to be highly effective in the primary prevention of type 2 diabetes.

#### Implications

In 1992, the consensus panel from the International Conference on Physical Activity, Fitness and Health (held in Toronto, Canada) [17] indicated that physical activity can effectively reduce the risk for, and incidence of, type 2 diabetes. Over 15 years later, the research is compelling; habitual physical activity is an effective primary preventative strategy against the development of type 2 diabetes [111-113,118-123]. As shown in our analyses, numerous observational studies have revealed that regular physical activity is associated with a lower risk of developing type 2 diabetes [111-113,118-123]. Moreover, increased aerobic fitness is inversely associated with the risk of type 2 diabetes [113,124]. It is also apparent that both aerobic and resistance type activities reduce the risk for type 2 diabetes [125,126].

Although it is difficult to determine the dose-response between physical activity and type 2 diabetes in the majority of the current randomized controlled trials, these trials have revealed important findings. Influential exercise and/or lifestyle intervention trials have demonstrated clearly the health benefits of physical activity/exercise in the prevention of type 2 diabetes. For instance, in the Diabetes Prevention Program (US), 3,234 high-risk participants were randomly assigned to one of three groups: a) a placebo control, b) metformin drug therapy (850 mg twice daily), and c) a lifestyle intervention. The authors revealed that the lifestyle intervention (including physical activity for at least 150 minutes per week) was more effective than metformin (alone) (respective reductions in incidence 58% and 31%) [127]. Similarly, Tuomilehto et al. (2001) conducted a randomized controlled trial with middle-aged, overweight subjects with impaired glucose tolerance (172 males and 350 females). The authors reported a significant reduction in the incidence of type 2 diabetes in the intervention group (which received advice regarding moderate intensity exercise (30 min/day) and dietary control). The lifestyle intervention reduced the risk of type 2 diabetes by approximately 54% in women and 63% in men [128]. In a review of the literature, Williamson et al. revealed modest weight loss via diet and physical activity reduced the incidence of type 2 diabetes in high risk individuals by 40-60% over a 3-4 year period [129]. Collectively, the epidemiological and randomized controlled trials provide compelling evidence supporting the role of habitual physical activity in the primary prevention of type 2 diabetes.

*Recommendation #6*

*For a reduced risk for type 2 diabetes, it is recommended that individuals should participate in 30 min or more of moderate to vigorous exercise on most days of the week. [Level 2, Grade A]*

### Primary Prevention of Osteoporosis

The protective effects of physical activity and exercise training on bone health are well documented. In fact, the relationship between indicators of bone health (such as bone mineral density or bone mineral content) and physical activity have been evaluated extensively (see Table 18). Numerous exercise intervention trials have revealed that aerobic and resistance activities have a beneficial effect on bone mineral density across the lifespan [16]. In fact, several systematic reviews of the literature [130-135] and major consensus statements [136] have shown clearly the potential benefits of both aerobic and resistance training on bone health (particularly in post-menopausal women). It has been estimated that exercise interventions prevent or reverse at least 1% of bone loss per year in the lumbar spine and the femoral neck of pre- and post-menopausal women [130,137].

**Table 18:** Studies examining the relationship between physical activity and osteoporosis.

Publication Country Study Design Quality Score	Objective	Population	Methods	Outcome	Comments and Conclusions
Robitaille et al 2008 [150]	To assess the relationship between the physician- diagnosised osteoporosis and family history and examine whether osteoporosis risk factors account for this relationship.	• n = 8,073	PA assessment: Questionnaire. Level of PA was expressed in MET (hr/wk)	Prevalence of reported osteoporosis in US women by PA level	Prevalence of osteoporosis declines with increasing PA in a dose-response manner.
		• Sex: Women			
		• Age: ≥ 20 yrs			
USA		• Characteristics: American women			
		• Study: NHANES (1999-2004)	G1 = 0	PA level (% prevalence)	
Cross-sectional			G2 = <30	• G1 = 11.0 (9.8-12.4)	
			G3 = ≥ 30	• G2 = 7.1 (6.0-8.4)	
D & B score = 10				• G3 = 3.9 (2.8-5.4)	
			Muscle strengthening activities were expressed in frequency/wk Times/week	*p *< 0.001	
				PA level (age adjusted)	
				• G1 = 8.9 (7.7-10.1)	
			G1 = 0	• G2 = 8.4 (7.3-9.7)	
			G2 = <2	• G3 = 6.2 (4.4-8.5)	
			G3 = ≥ 2	*p *< 0.01	
			Criteria for diagnosis of osteoporosis: Self-reported physician diagnosed	Muscle strengthening (%prevalence)	
				• G1 = 8.1 (7.2-9.1)	
			Chi-square	• G2 = 3.1 (1.7-5.5)	
				• G3 = 7.4 (5.8-9.4)	
				*p *< 0.001	
				Muscle strengthening (age adjusted)	
				• G1 = 7.8 (6.9-8.7)	
				• G2 = 6.7 (3.8-11.8)	
				• G3 = 9.5 (7.6-11.9)	
				*p *< 0.05	

Keramat et al 2008 [151]	To assess risk factors for osteoporosis in postmenopausal women from selected BMD centers in Iran and India.	• Iran n = 363; 178 case, 185 control	Study period 2002 -- 2005	OR (95% CI) of osteoporosis in exercisers vs. non-exercisers. Iran (age adjusted)	Exercise was shown as protective factor in both countries and it remained significant after adjustment for age weight and height in Iran.
		• India n = 354; 203 case, 151 control	PA assessment: Questionnaire. PA was categorized as exercises, other exercises (e.g., swimming, aerobics, weight training) and walking		
Iran and India					
		• Sex: Women		• Exercises = 0.4 (0.2-0.7)	
Case control		• Age: Iran Case = 58.2 (7.1) yr; Iran Control = 55.7 (6.0) yr; India Case = 58.9 (8.1) yr; India Control = 56.4 (7.5) yr	BMD assessment: DEXA	• Other exercises = 0.4 (0.2-0.6)	
		• Characteristics: Cases had BMD > 2.5 SD below average of young normal bone density in L1-L4 spine region and/or total femoral region. Controls had BMD < 1 SD below normal	Multinominal logistic regression	• Regular Walking = 0.5 (0.3- 0.8)	
D & B score = 11					Walking and other exercises were shown as protective factors in Iranian subjects.
				Iran (age, weight, height adjusted)	
				• Exercises = 0.4 (0.2-0.7)	
				• Other exercises = 0.3 (0.2-0.6)	
				• Regular Walking = 0.4 (0.2- 0.8) I	
				ndia (age adjusted)	
				• Exercises = 0.4 (0.3-0.9)	
				• Other exercises = NS	
				• Regular Walking = NS	
				India (age, weight, height adjusted)	
				• Exercises = NS	
		• Ethnicity: Indian and Iranian		• Other exercises = NS	
				• Regular Walking = 0.4 (0.2- 0.8)	

D & B score, Downs and Black quality score; YR, years; MET/wk, metabolic equivalent per week; G, groups; PA, physical activity; BMD, bone mineral density; SD, standard deviation; DEXA, dual energy x-ray absorptiometry; NS, not significant.

Exercise has also been shown to significantly reduce the risk and/or number of falls in comparison to inactive controls [138-142]. Moreover, fracture risk and/or incidence has been shown to be reduced in active individuals [143-145]. Case-control studies of older persons who suffered a hip fracture have revealed that these individuals had significantly lower physical activity levels throughout adulthood [136,146]. Observational studies have also revealed an inverse relationship between the incidence of fractures and physical activity [147-149]. For instance, Joakimsen et al. revealed lower fracture rates in individuals who performed more weight-bearing activities [148]. Similarly, Kujala et al. [147] in a 21-year prospective study revealed that intense activity was associated with a lower incidence of hip fracture (Hazard Ratio = 0.38, 95% CI = 0.16-0.91). Feskanich et al. (2002) also revealed that moderate physical activity was inversely related to the risk of hip fracture in postmenopausal women [149]. In a review of observational trials, Katzmarzyk and Janssen [20] revealed that the fracture risk was markedly higher in habitually inactive individuals (RR = 1.59 (95% CI = 1.40-1.80)) with a population attributable risk of 24% in Canada.

**Figure 10:**
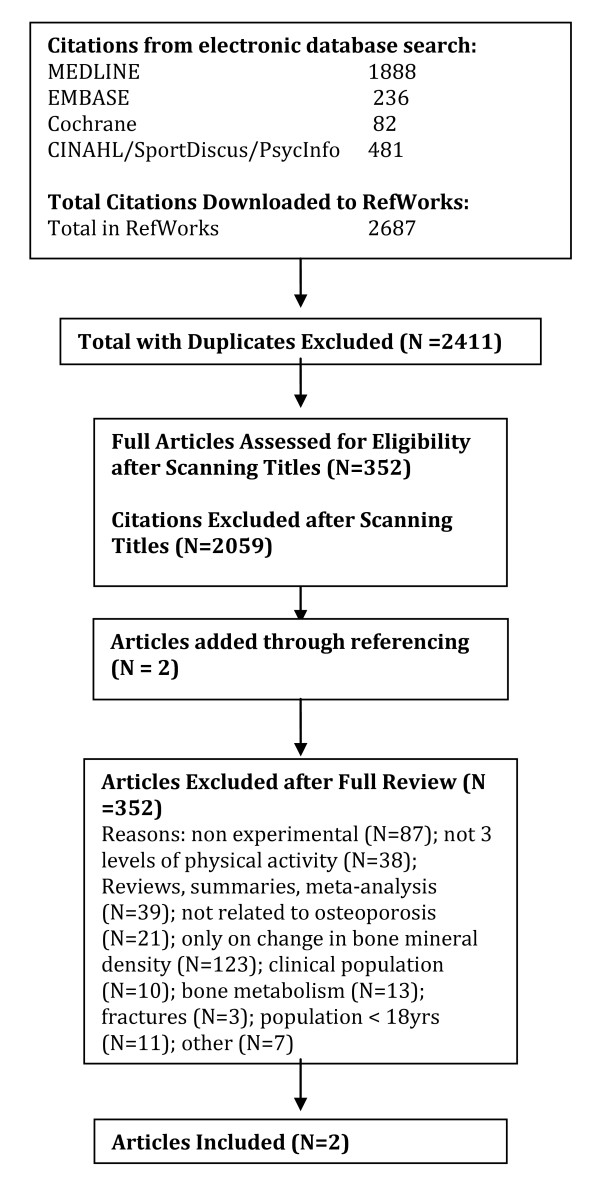
**Results of the Literature Search for Osteoporosis**.

There is clear evidence that exercise training is of benefit for bone health and accordingly reduces the risk for osteoporosis. However, remarkably limited research has actually examined the relationship between routine physical activity and the prevalence and/or incidence of osteoporosis (Figure 10). In our systematic search of the osteoporosis literature, a total of 3655 citations were identified during the electronic database search (Figure 7). Of these citations, 1888 were identified in MEDLINE, 236 in EMBASE, 82 in Cochrane, and 481 in the CINAHL/SportDiscus/PsychInfo search. A total of 276 duplicates were found, leaving a total of 2411 unique citations. A total of 2059 articles were excluded after screening, leaving a total of 352 articles for full review. From these articles all 352 were excluded after full-text review. The reasons for exclusion included non-experimental/weak design (n = 87), did not contain three levels of physical activity or not possible to determine dose-response relationship (n = 38), reviews, summaries, meta-analyses (n = 39), not dealing specifically with osteoporosis (n = 21), only on change in bone mineral density (N = 123), clinical population (N = 10), bone metabolism (N = 13), fractures (N = 3), population < 18 yrs (N = 11), and other (N = 7). An additional 2 articles were found through the authors' knowledge of the field.

As identified in our systematic search, the majority of the literature has dealt with the relationship between physical activity and indicators of bone health and/or the incidence of fractures. However, a recent observational trial [150] has provided evidence supporting the ability of physical activity to reduce the incidence of osteoporosis. For instance, Robitaille et al. revealed a dose-response relationship between physical activity level and the prevalence of reported osteoporosis in 8073 women aged ≥ 20 yr in the National Health and Nutrition Examination Survey, 1999-2004 [150]. Those performing no physical activity were at a higher risk than those who engaged in moderate (<30 MET hr/wk) and high (>30 MET hr/wk) levels of physical activity. There was a dose-response relationship with the highest physical activity group having the lowest prevalence of osteoporosis. Similarly, Keramat et al. [151] in a case-control investigation revealed that physical activity was protective against the development of osteoporosis.

At this time it is difficult to define clearly the actual dose-response required to cause a reduction in the incidence of osteoporosis. It is clear that bone adaptations to exercise are load dependent and site specific [9,10,16,152]. As such, physical activities that involve significantly loading/impact are often advocated for the prevention of osteoporosis. It is has been shown that running 15-20 miles per week is associated with bone mineral accrual or maintenance. Longer distances however may be associated with reduced bone mineral density [136].

Feskanich et al. reported that the risk of hip fracture was lowered by 6% for each increase of 3 MET-hours per week of activity (or 1 hr/wk of walking at an average pace) [149]. There was a linear reduction with increasing physical activity level. Walking for at least 4 hr/wk was also associated with a 41% lower risk of hip fracture compared to less than 1 hr/wk [149]. The work of Robitaille et al. also indicated that moderate levels of physical activity are sufficient to reduce the prevalence of osteoporosis [150].

In summary, there is preliminary evidence to indicate that the current Canadian physical activity guidelines are sufficient to maintain and/improve bone health. However, further research is clearly required, in particular research that examines the relationship between physical activity and the incidence of osteoporosis in both men and women from varied ethnic backgrounds. Currently, the level of evidence would be considered to be at a Level 3A. The quality of the investigations was generally low with a mean (and median) Downs and Black score of 11.

*Recommendation #7*

*For a reduced risk for osteoporosis, it is recommended that individuals should participate in load bearing activities for 30 min or more on most days of the week. [Level 3, Grade A]*

### Other Considerations

#### Musculoskeletal Fitness and Health

In the present analyses, all indices of physical activity/fitness were incorporated into our systematic reviews. Although the majority of the data is related to aerobic activities, it should be noted that many of these activities also had a significant musculoskeletal component. Moreover, direct measurements of musculoskeletal fitness were included in various studies included in our review. Although there is limited information available (in comparison to aerobic activities) there is compelling evidence that musculoskeletal fitness is also positively associated with health status [9,10,16].

Warburton and colleagues [9,10] in two reviews of the literature reported that enhanced musculoskeletal fitness is associated positively with glucose homeostasis, bone health, functional independence, mobility, psychological well-being, and overall quality of life and negatively associated with fall risk, morbidity and premature mortality. They also reported that interventions that increase musculoskeletal fitness also have a significant positive effect on the health status of the individuals with a low musculoskeletal reserve (e.g., the frail elderly).

In an evaluation of the current literature some key findings emerge. Grip strength has particularly been shown to be inversely related to premature mortality and/or morbidity (e.g., functional limitations) [153-156]. Rantanen et al. (1998) reported that those individuals with the lowest grip strength had a higher rate of mortality at a younger age (over a 27- year period) than their counterparts with higher muscular strength. Furthermore, they revealed that those with a faster rate of decline in muscular strength (>1.5% per year) or a very low grip strength (<21 kg) had a greater incidence of chronic diseases, such as type 2 diabetes, stroke, arthritis, coronary heart disease, and pulmonary disorders. It was shown that those in the lowest grip strength tertile had an 8-fold increased risk for disability. Individuals with high muscular strength have also been shown to develop less functional limitations in comparison to their counterparts with lower strength over a 5-year period [157].

Katzmarzyk and colleagues [126,154,158] in Canada have also demonstrated a positive relationship between musculoskeletal fitness and health status. For instance, Katzmarzyk and Craig (2002) revealed that there was a significantly higher risk of premature mortality in the lower quartile of sit-ups in both men (RR = 2.72) and women (RR = 2.26). Grip strength was also predictive of mortality in men (RR = 1.49), but not women. In a recent study, Mason et al. revealed that musculoskeletal fitness was a significant predictor of weight gain over a 20-year period [158]. Importantly, they also reported that individuals with low musculoskeletal fitness had 78% greater odds of significant weight gain (≥ 10 kg) compared to those with high musculoskeletal fitness. These studies provide direct support for the inclusion of resistance and flexibility training in Canada's physical activity guidelines for adults [3,159].

*Recommendation #8*

*For improved health status and reduced risk for chronic disease and disability, it is recommended that individuals should include daily activities that tax the musculoskeletal system [Level 2, Grade A]*

## Limitations

It is important to note that for each chronic condition, the methods used to determine the relationship between physical activity and the specific clinical outcome were often quite varied. For instance, early work in the field generally controlled for few confounding variables (such as age). In comparison, current literature often controls for a myriad of potential confounding variables. These discrepancies make the comparison of the relative risk reductions between studies and across clinical conditions more difficult. Moreover, the multivariate analyses (controlling for various potential confounding factors) may inappropriately decrease the level of risk reduction associated with physical activity and the clinical endpoint [31]. This is owing to the fact that some of the health benefits associated with physical activity may be mediated through these variables [31].

There was often considerable variability in the findings and major conclusions of the studies examined. Often the available literature was limited by the lack of a clear standard for assessing physical activity. In many instances, it was extremely difficult to determine the actual dosage of physical activity used to group the participants. This lack of clarity makes it very difficult to clearly define the dose-response relationship between physical activity and various chronic conditions.

## Conclusions

There is incontrovertible evidence that regular exercise is an effective preventative strategy against premature mortality, cardiovascular disease, stroke, hypertension, colon cancer, breast cancer, and type 2 diabetes. There is also compelling indirect evidence to support the protective effects of physical activity with respect to osteoporosis. In many instances the dose-response relationship is linear with further health benefits with increasing levels of activity. The current Canadian physical activity guidelines for adults are sufficient to reduce the risk for multiple chronic diseases simultaneously. The acknowledgement that every bit of exercise counts towards health benefits (with greater benefits at higher energy expenditures) is consistent with the literature and a reasonable message to promote to the general population. However, further investigation is likely required to evaluate the relationship between physical activity and health status in non-Caucasian populations.

## Competing interests

The authors declare that they have no competing interests.

## Authors' contributions

DW was responsible for the conceptualization and design of the systematic review, the generation of the systematic review terms, oversaw the data collection, evaluated each article included in the review, and was responsible for creating and revising the manuscript. SC was involved in the data collection, the critical review of the articles, the creation of the tables contained in the article and the revision of the manuscript. AI assisted with the data collection, the critical review of the articles, and the creation and the revision of tables in the manuscript. LN assisted with the generation of the systematic review terms, the retrieval of articles, and the generation and revision of the tables. SB was involved in the conceptualization and design of the systematic review, the generation of the systematic review terms, oversaw the data collection, the review of the articles, and was responsible for creating and revising the manuscript. All authors have read and approved the final manuscript.

## References

[B1] Bouchard C Shephard RJ Bouchard C, Shephard RJ, Stephens T Physical activity fitness and health: the model and key concepts Physical activity fitness and health: International proceedings and consensus statement 1994 Champaign, IL: Human Kinetics 77 88

[B2] Blair SN Brodney S Effects of physical inactivity and obesity on morbidity and mortality: current evidence and research issues Med Sci Sports Exerc 1999 7 S646 662 10.1097/00005768-199911001-00025 10593541

[B3] American College of Sports Medicine Position stand: Exercise and physical activity for older adults Med Sci Sports Exerc 1998 7 992 1008 10.1097/00005768-199806000-00033 9624662

[B4] McAuley E Bouchard C, Shephard RJ, Stephens T Physical activity and psychosocial outcomes Physical activity, fitness and health: the consensus knowledge 1994 Champaign, IL: Human Kinetics 551 568

[B5] Taylor RS Brown A Ebrahim S Jolliffe J Noorani H Rees K Skidmore B Stone JA Thompson DR Oldridge N Exercise-based rehabilitation for patients with coronary heart disease: systematic review and meta-analysis of randomized controlled trials Am J Med 2004 7 682 692 10.1016/j.amjmed.2004.01.009 15121495

[B6] Blair SN Cheng Y Holder JS Is physical activity or physical fitness more important in defining health benefits? Med Sci Sports Exerc 2001 7 S379 399 discussion S419-320 10.1097/00005768-200105001-01549 11427763

[B7] Blair SN Kohl HW Paffenbarger RS Jr Clark DG Cooper KH Gibbons LW Physical fitness and all-cause mortality. A prospective study of healthy men and women JAMA 1989 7 2395 2401 10.1001/jama.262.17.2395 2795824

[B8] Paffenbarger RS Jr Hyde RT Hsieh CC Wing AL Physical activity, other life-style patterns, cardiovascular disease and longevity Acta Med Scand Suppl 1986 7 85 91 3535417 10.1111/j.0954-6820.1986.tb08936.x

[B9] Warburton DE Gledhill N Quinney A Musculoskeletal fitness and health Can J Appl Physiol 2001 7 217 237 11312417 10.1139/h01-013

[B10] Warburton DE Gledhill N Quinney A The effects of changes in musculoskeletal fitness on health Can J Appl Physiol 2001 7 161 216 11312416 10.1139/h01-012

[B11] U.S. Department of Health and Human Services Healthy People 2000: National Health Promotion and Disease Prevention Objectives 1991 Washington, D.C.: U.S. Department of Health and Human Services

[B12] Puett DW Griffin MR Published trials of nonmedicinal and noninvasive therapies for hip and knee osteoarthritis Ann Intern Med 1994 7 133 140 8017727 10.7326/0003-4819-121-2-199407150-00010

[B13] Shephard RJ Absolute versus relative intensity of physical activity in a dose-response context Med Sci Sports Exerc 2001 7 S400 418 discussion S419-420 10.1097/00005768-200106001-00008 11427764

[B14] Lee IM Skerrett PJ Physical activity and all-cause mortality: what is the dose-response relation? Med Sci Sports Exerc 2001 7 S459 471 discussion S493-454 10.1097/00005768-200106001-00016 11427772

[B15] Warburton DE Nicol C Bredin SS Prescribing exercise as preventive therapy CMAJ 2006 7 961 974 16567757 10.1503/cmaj.1040750 PMC1405860

[B16] Warburton DE Nicol C Bredin SS Health benefits of physical activity: the evidence CMAJ 2006 7 801 809 16534088 10.1503/cmaj.051351 PMC1402378

[B17] Bouchard C Shephard RJ Stephens T Bouchard C, Shephard RJ, Stephens T The consensus statement Physical activity fitness and health: International proceedings and consensus statement 1994 Champaign, IL: Human Kinetics 9 76

[B18] Warburton DER Katzmarzyk PT Rhodes RE Shephard RJ Evidence-informed physical activity guidelines for Canadian adults Appl Physiol Nutr Metab 2007 7 S17 74 10.1139/H07-168 19377540

[B19] Warburton DER Katzmarzyk PT Rhodes RE Shephard RJ Evidence-informed physical activity guidelines for Canadian adults Can J Pub Health 2007 7 S16 S68 18213940

[B20] Katzmarzyk PT Janssen I The economic costs associated with physical inactivity and obesity in Canada: an update Can J Appl Physiol 2004 7 90 115 15001807 10.1139/h04-008

[B21] Health Canada and Canadian Society for Exercise Physiology Canada's Physical Activity Guide to Healthy Active Living 1998 Ottawa, ON: Health Canada (Cat. No. H39-429/1998-1E) http://www.paguide.com

[B22] Health Canada and Canadian Society for Exercise Physiology Canada's Physical Activity Guide to Healthy Active Living for Older Adults 1999 Ottawa, ON: Health Canada http://www.paguide.com

[B23] Health Canada and Canadian Society for Exercise Physiology Canada's Physical Activity Guide to Healthy Active Living for Children and Youths 2002 Ottawa, ON: Health Canada http://www.paguide.com

[B24] Canadian Society for Exercise Physiology Canadian Physical Activity, Fitness and Lifestyle Approach 2003 3 Ottawa: Canadian Society for Exercise Physiology

[B25] Lau DC Douketis JD Morrison KM Hramiak IM Sharma AM Ur E 2006 Canadian clinical practice guidelines on the management and prevention of obesity in adults and children [summary] CMAJ 2007 7 S1 13 17420481 10.1503/cmaj.061409 PMC1839777

[B26] Gorber SC Tremblay M Moher D Gorber B A comparison of direct vs. self-report measures for assessing height, weight and body mass index: a systematic review Obes Rev 2007 7 307 326 10.1111/j.1467-789X.2007.00347.x 17578381

[B27] Downs SH Black N The feasibility of creating a checklist for the assessment of the methodological quality both of randomised and non-randomised studies of health care interventions J Epidemiol Community Health 1998 7 377 384 10.1136/jech.52.6.377 9764259 PMC1756728

[B28] Prince SA Adamo KB Hamel ME Hardt J Gorber SC Tremblay M A comparison of direct versus self-report measures for assessing physical activity in adults: a systematic review Int J Behav Nutr Phys Act 2008 7 56 10.1186/1479-5868-5-56 18990237 PMC2588639

[B29] Macera CA Hootman JM Sniezek JE Major public health benefits of physical activity Arthritis Rheum 2003 7 122 128 10.1002/art.10907 12579603

[B30] Macera CA Powell KE Population attributable risk: implications of physical activity dose Med Sci Sports Exerc 2001 7 S635 639 discussion 640-631 10.1097/00005768-200106001-00032 11427788

[B31] Physical Activity Guidelines Advisory Committee Physical Activity Guidelines Advisory Committee Report 2008 Washington, DC: U.S. Department of Health and Human Services 683

[B32] Myers J Kaykha A George S Abella J Zaheer N Lear S Yamazaki T Froelicher V Fitness versus physical activity patterns in predicting mortality in men Am J Med 2004 7 912 918 10.1016/j.amjmed.2004.06.047 15629729

[B33] Williams PT Physical fitness and activity as separate heart disease risk factors: a meta-analysis Med Sci Sports Exerc 2001 7 754 761 11323544 10.1097/00005768-200105000-00012 PMC2821586

[B34] Erikssen G Physical fitness and changes in mortality: the survival of the fittest Sports Med 2001 7 571 576 10.2165/00007256-200131080-00001 11475318

[B35] Erikssen G Liestol K Bjornholt J Thaulow E Sandvik L Erikssen J Changes in physical fitness and changes in mortality Lancet 1998 7 759 762 10.1016/S0140-6736(98)02268-5 9737279

[B36] Blair SN Kohl HW Barlow CE Paffenbarger RS Jr Gibbons LW Macera CA Changes in physical fitness and all-cause mortality. A prospective study of healthy and unhealthy men JAMA 1995 7 1093 1098 10.1001/jama.273.14.1093 7707596

[B37] Bijnen FC Feskens EJ Caspersen CJ Nagelkerke N Mosterd WL Kromhout D Baseline and previous physical activity in relation to mortality in elderly men: the Zutphen Elderly Study Am J Epidemiol 1999 7 1289 1296 10604771 10.1093/oxfordjournals.aje.a009960

[B38] Johansson SE Sundquist J Change in lifestyle factors and their influence on health status and all-cause mortality Int J Epidemiol 1999 7 1073 1080 10.1093/ije/28.6.1073 10661650

[B39] Gregg EW Cauley JA Stone K Thompson TJ Bauer DC Cummings SR Ensrud KE Relationship of changes in physical activity and mortality among older women JAMA 2003 7 2379 2386 10.1001/jama.289.18.2379 12746361

[B40] Wannamethee SG Shaper AG Walker M Changes in physical activity, mortality, and incidence of coronary heart disease in older men Lancet 1998 7 1603 1608 10.1016/S0140-6736(97)12355-8 9620713

[B41] Kujala UM Kaprio J Koskenvuo M Modifiable risk factors as predictors of all-cause mortality: the roles of genetics and childhood environment Am J Epidemiol 2002 7 985 993 10.1093/aje/kwf151 12446254

[B42] Wessel TR Arant CB Olson MB Johnson BD Reis SE Sharaf BL Shaw LJ Handberg E Sopko G Kelsey SF Pepine CJ Merz NB Relationship of physical fitness vs body mass index with coronary artery disease and cardiovascular events in women JAMA 2004 7 1179 1187 10.1001/jama.292.10.1179 15353530

[B43] Katzmarzyk PT Church TS Blair SN Cardiorespiratory fitness attenuates the effects of the metabolic syndrome on all-cause and cardiovascular disease mortality in men Arch Intern Med 2004 7 1092 1097 10.1001/archinte.164.10.1092 15159266

[B44] Morris JN Heady JA Mortality in relation to the physical activity of work: a preliminary note on experience in middle age Br J Ind Med 1953 7 245 254 13106231 10.1136/oem.10.4.245 PMC1037497

[B45] Morris JN Heady JA Raffle PA Roberts CG Parks JW Coronary heart-disease and physical activity of work Lancet 1953 7 1111 1120 10.1016/S0140-6736(53)91495-0 13110075

[B46] Paffenbarger RS Jr Brand RJ Sholtz RI Jung DL Energy expenditure, cigarette smoking, and blood pressure level as related to death from specific diseases Am J Epidemiol 1978 7 12 18 685971

[B47] Paffenbarger RS Hale WE Work activity and coronary heart mortality N Engl J Med 1975 7 545 550 1128551 10.1056/NEJM197503132921101

[B48] United States Department of Health and Human Services Physical activity and health: a report of the Surgeon General 1996 Atlanta, G.A.: Department of Health and Human Services, Centers for Disease Control and Prevention, National Center for Chronic Disease Prevention and Health Promotion

[B49] Booth FW Gordon SE Carlson CJ Hamilton MT Waging war on modern chronic diseases: primary prevention through exercise biology J Appl Physiol 2000 7 774 787 10658050 10.1152/jappl.2000.88.2.774

[B50] Katzmarzyk PT Gledhill N Shephard RJ The economic burden of physical inactivity in Canada CMAJ 2000 7 1435 1440 11192648 PMC80410

[B51] Oguma Y Sesso HD Paffenbarger RS Jr Lee IM Physical activity and all cause mortality in women: a review of the evidence Br J Sports Med 2002 7 162 172 10.1136/bjsm.36.3.162 12055109 PMC1724493

[B52] Katzmarzyk P Kohl HW Physical Activity Status and Chronic Diseases ACSM's Resource Manual for Guidelines for Exercise Testing and Prescription 2005 5 Philadelphia: Lippincott, Williams and Wilkins 122 135

[B53] Statistics Canada Deaths, 2002 2004 Ottawa: Statistics Canada http://www.statcan.ca/Daily/English/040927/d040927a.htm

[B54] World Health Organization The World Health Report: Reducing risks, promoting healthy life 2002 Geneva: World Health Organization

[B55] Emberson JR Whincup PH Morris RW Wannamethee SG Shaper AG Lifestyle and cardiovascular disease in middle-aged British men: the effect of adjusting for within-person variation Eur Heart J 2005 7 1774 1782 10.1093/eurheartj/ehi224 15821008

[B56] Manson JE Greenland P LaCroix AZ Stefanick ML Mouton CP Oberman A Perri MG Sheps DS Pettinger MB Siscovick DS Walking compared with vigorous exercise for the prevention of cardiovascular events in women N Engl J Med 2002 7 716 725 10.1056/NEJMoa021067 12213942

[B57] Oguma Y Shinoda-Tagawa T Physical activity decreases cardiovascular disease risk in women: review and meta-analysis Am J Prev Med 2004 7 407 418 10.1016/j.amepre.2004.02.007 15165657

[B58] Wisloff U Nilsen TI Droyvold WB Morkved S Slordahl SA Vatten LJ A single weekly bout of exercise may reduce cardiovascular mortality: how little pain for cardiac gain? 'The HUNT study, Norway' Eur J Cardiovasc Prev Rehabil 2006 7 798 804 10.1097/01.hjr.0000216548.84560.ac 17001221

[B59] Lee IM Rexrode KM Cook NR Manson JE Buring JE Physical activity and coronary heart disease in women: is "no pain, no gain" passe? JAMA 2001 7 1447 1454 10.1001/jama.285.11.1447 11255420

[B60] Berlin JA Colditz GA A meta-analysis of physical activity in the prevention of coronary heart disease Am J Epidemiol 1990 7 612 628 2144946 10.1093/oxfordjournals.aje.a115704

[B61] Bauman AE Updating the evidence that physical activity is good for health: an epidemiological review 2000-2003 J Sci Med Sport 2004 7 6 19 10.1016/S1440-2440(04)80273-1 15214597

[B62] Kohl HW Physical activity and cardiovascular disease: evidence for a dose response Med Sci Sports Exerc 2001 7 S472 483 discussion S493-474 10.1097/00005768-200105001-01015 11427773

[B63] Paffenbarger RS Jr Hyde RT Wing AL Hsieh CC Physical activity, all-cause mortality, and longevity of college alumni N Engl J Med 1986 7 605 613 3945246 10.1056/NEJM198603063141003

[B64] Schnohr P Scharling H Jensen JS Intensity versus duration of walking, impact on mortality: the Copenhagen City Heart Study Eur J Cardiovasc Prev Rehabil 2007 7 72 78 10.1097/HJR.0b013e3280144470 17301630

[B65] Kushi LH Fee RM Folsom AR Mink PJ Anderson KE Sellers TA Physical activity and mortality in postmenopausal women JAMA 1997 7 1287 1292 10.1001/jama.277.16.1287 9109466

[B66] Leon AS Connett J Jacobs DR Jr Rauramaa R Leisure-time physical activity levels and risk of coronary heart disease and death. The Multiple Risk Factor Intervention Trial JAMA 1987 7 2388 2395 10.1001/jama.258.17.2388 3669210

[B67] Paffenbarger RS Jr Hyde RT Wing AL Lee IM Jung DL Kampert JB The association of changes in physical-activity level and other lifestyle characteristics with mortality among men N Engl J Med 1993 7 538 545 10.1056/NEJM199302253280804 8426621

[B68] Heart and Stroke Foundation of Canada Press Releases - Canadian leads effort to raise awareness of stroke as a global health epidemic 2007 Ottawa: Heart and Stroke Foundation of Canada

[B69] Abbott RD Curb JD Rodriguez BL Masaki KH Popper JS Ross GW Petrovitch H Age-related changes in risk factor effects on the incidence of thromboembolic and hemorrhagic stroke J Clin Epidemiol 2003 7 479 486 10.1016/S0895-4356(02)00611-X 12812823

[B70] Gillum RF Mussolino ME Ingram DD Physical activity and stroke incidence in women and men. The NHANES I Epidemiologic Follow-up Study Am J Epidemiol 1996 7 860 869 8610699 10.1093/oxfordjournals.aje.a008829

[B71] Lee CD Blair SN Cardiorespiratory fitness and stroke mortality in men Med Sci Sports Exerc 2002 7 592 595 10.1097/00005768-200205001-01747 11932565

[B72] Hu FB Stampfer MJ Colditz GA Ascherio A Rexrode KM Willett WC Manson JE Physical activity and risk of stroke in women JAMA 2000 7 2961 2967 10.1001/jama.283.22.2961 10865274

[B73] Lee IM Paffenbarger RS Jr Physical activity and stroke incidence: the Harvard Alumni Health Study Stroke 1998 7 2049 2054 9756580 10.1161/01.str.29.10.2049

[B74] Lee IM Hennekens CH Berger K Buring JE Manson JE Exercise and risk of stroke in male physicians Stroke 1999 7 1 6 9880379 10.1161/01.str.30.1.1

[B75] Rankinen T Church TS Rice T Bouchard C Blair SN Cardiorespiratory fitness, BMI, and risk of hypertension: the HYPGENE study Med Sci Sports Exerc 2007 7 1687 1692 10.1249/mss.0b013e31812e527f 17909393

[B76] Pereira MA Folsom AR McGovern PG Carpenter M Arnett DK Liao D Szklo M Hutchinson RG Physical activity and incident hypertension in black and white adults: the Atherosclerosis Risk in Communities Study Prev Med 1999 7 304 312 10.1006/pmed.1998.0431 10072750

[B77] Haapanen N Miilunpalo S Vuori I Oja P Pasanen M Association of leisure time physical activity with the risk of coronary heart disease, hypertension and diabetes in middle-aged men and women Int J Epidemiol 1997 7 739 747 10.1093/ije/26.4.739 9279605

[B78] Paffenbarger RS Jr Wing AL Hyde RT Jung DL Physical activity and incidence of hypertension in college alumni Am J Epidemiol 1983 7 245 257 6829553 10.1093/oxfordjournals.aje.a113537

[B79] Paffenbarger RS Jr Lee IM Intensity of physical activity related to incidence of hypertension and all-cause mortality: an epidemiological view Blood Press Monit 1997 7 115 123 10234104

[B80] Hernelahti M Kujala UM Kaprio J Sarna S Long-term vigorous training in young adulthood and later physical activity as predictors of hypertension in middle-aged and older men Int J Sports Med 2002 7 178 182 10.1055/s-2002-23176 11914980

[B81] Hu G Barengo NC Tuomilehto J Lakka TA Nissinen A Jousilahti P Relationship of physical activity and body mass index to the risk of hypertension: a prospective study in Finland Hypertension 2004 7 25 30 10.1161/01.HYP.0000107400.72456.19 14656958

[B82] Gu D Wildman RP Wu X Reynolds K Huang J Chen CS He J Incidence and predictors of hypertension over 8 years among Chinese men and women J Hypertens 2007 7 517 523 10.1097/HJH.0b013e328013e7f4 17278966

[B83] Hayashi T Tsumura K Suematsu C Okada K Fujii S Endo G Walking to work and the risk for hypertension in men: the Osaka Health Survey Ann Intern Med 1999 7 21 26 10391811 10.7326/0003-4819-131-1-199907060-00005

[B84] Nakanishi N Suzuki K Daily life activity and the risk of developing hypertension in middle-aged Japanese men Arch Intern Med 2005 7 214 220 10.1001/archinte.165.2.214 15668369

[B85] Foy CG Foley KL D'Agostino RB Jr Goff DC Jr Mayer-Davis E Wagenknecht LE Physical activity, insulin sensitivity, and hypertension among US adults: findings from the Insulin Resistance Atherosclerosis Study Am J Epidemiol 2006 7 921 928 10.1093/aje/kwj113 16554349

[B86] Haennel RG Lemire F Physical activity to prevent cardiovascular disease. How much is enough? Can Fam Physician 2002 7 65 71 11852614 PMC2213927

[B87] Fagard RH Exercise characteristics and the blood pressure response to dynamic physical training Med Sci Sports Exerc 2001 7 S484 492 discussion S493-484 10.1097/00005768-200106001-00018 11427774

[B88] Pescatello LS Franklin BA Fagard R Farquhar WB Kelley GA Ray CA American College of Sports Medicine position stand. Exercise and hypertension Med Sci Sports Exerc 2004 7 533 553 10.1249/01.MSS.0000115224.88514.3A 15076798

[B89] Cornelissen VA Fagard RH Effect of resistance training on resting blood pressure: a meta-analysis of randomized controlled trials J Hypertens 2005 7 251 259 10.1097/00004872-200502000-00003 15662209

[B90] National Center for Health Statistics Fast Stats: Hypertension 2008 Hyattsville, MD: Centers for Disease Control and Prevention

[B91] Statistics Canada CANSIM 2007 Ottawa: Statistics Canada

[B92] McAlister FA Wooltorton E Campbell NR The Canadian Hypertension Education Program (CHEP) recommendations: launching a new series CMAJ 2005 7 508 509 16129873 10.1503/cmaj.050737 PMC1188189

[B93] Joffres MR Ghadirian P Fodor JG Petrasovits A Chockalingam A Hamet P Awareness, treatment, and control of hypertension in Canada Am J Hypertens 1997 7 1097 1102 10.1016/S0895-7061(97)00224-0 9370379

[B94] Fagard RH Physical activity in the prevention and treatment of hypertension in the obese Med Sci Sports Exerc 1999 7 S624 630 10.1097/00005768-199911001-00022 10593538

[B95] Kelley GA Aerobic exercise and resting blood pressure among women: a metaanalysis Prev Med 1999 7 264 275 10.1006/pmed.1998.0417 10072745

[B96] Whelton SP Chin A Xin X He J Effect of aerobic exercise on blood pressure: a meta-analysis of randomized, controlled trials Ann Intern Med 2002 7 493 503 11926784 10.7326/0003-4819-136-7-200204020-00006

[B97] Dickinson HO Mason JM Nicolson DJ Campbell F Beyer FR Cook JV Williams B Ford GA Lifestyle interventions to reduce raised blood pressure: a systematic review of randomized controlled trials J Hypertens 2006 7 215 233 10.1097/01.hjh.0000199800.72563.26 16508562

[B98] Hamer M Taylor A Steptoe A The effect of acute aerobic exercise on stress related blood pressure responses: a systematic review and meta-analysis Biol Psychol 2006 7 183 190 10.1016/j.biopsycho.2005.04.004 15979232

[B99] Fagard RH Cornelissen VA Effect of exercise on blood pressure control in hypertensive patients Eur J Cardiovasc Prev Rehabil 2007 7 12 17 10.1097/HJR.0b013e3280128bbb 17301622

[B100] Fagard RH Exercise is good for your blood pressure: effects of endurance training and resistance training Clin Exp Pharmacol Physiol 2006 7 853 856 10.1111/j.1440-1681.2006.04453.x 16922820

[B101] Fagard RH Effects of exercise, diet and their combination on blood pressure J Hum Hypertens 2005 7 Suppl 3 S20 24 10.1038/sj.jhh.1001956 16302006

[B102] Cornelissen VA Fagard RH Effects of endurance training on blood pressure, blood pressure-regulating mechanisms, and cardiovascular risk factors Hypertension 2005 7 667 675 10.1161/01.HYP.0000184225.05629.51 16157788

[B103] Thompson PD Crouse SF Goodpaster B Kelley D Moyna N Pescatello L The acute versus the chronic response to exercise Med Sci Sports Exerc 2001 7 S438 445 discussion S452-433 10.1097/00005768-200106001-00012 11427768

[B104] Monninkhof EM Elias SG Vlems FA Tweel I van der Schuit AJ Voskuil DW van Leeuwen FE Physical activity and breast cancer: a systematic review Epidemiology 2007 7 137 157 10.1097/01.ede.0000251167.75581.98 17130685

[B105] Lee IM Physical activity and cancer prevention--data from epidemiologic studies Med Sci Sports Exerc 2003 7 1823 1827 10.1249/01.MSS.0000093620.27893.23 14600545

[B106] Rockhill B Willett WC Hunter DJ Manson JE Hankinson SE Colditz GA A prospective study of recreational physical activity and breast cancer risk Arch Intern Med 1999 7 2290 2296 10.1001/archinte.159.19.2290 10547168

[B107] Sesso HD Paffenbarger RS Jr Lee IM Physical activity and breast cancer risk in the College Alumni Health Study (United States) Cancer Causes Control 1998 7 433 439 10.1023/A:1008827903302 9794176

[B108] Shephard RJ Futcher R Physical activity and cancer: how may protection be maximized? Crit Rev Oncog 1997 7 219 272 9570295 10.1615/critrevoncog.v8.i2-3.40

[B109] Thune I Furberg AS Physical activity and cancer risk: dose-response and cancer, all sites and site-specific Med Sci Sports Exerc 2001 7 S530 550 discussion S609-510 10.1097/00005768-200106001-00025 11427781

[B110] Cust AE Armstrong BK Friedenreich CM Slimani N Bauman A Physical activity and endometrial cancer risk: a review of the current evidence, biologic mechanisms and the quality of physical activity assessment methods Cancer Causes Control 2007 7 243 58 10.1007/s10552-006-0094-7 17206535

[B111] Hu FB Li TY Colditz GA Willett WC Manson JE Television watching and other sedentary behaviors in relation to risk of obesity and type 2 diabetes mellitus in women JAMA 2003 7 1785 1791 10.1001/jama.289.14.1785 12684356

[B112] Manson JE Nathan DM Krolewski AS Stampfer MJ Willett WC Hennekens CH A prospective study of exercise and incidence of diabetes among US male physicians JAMA 1992 7 63 67 10.1001/jama.268.1.63 1608115

[B113] Lynch J Helmrich SP Lakka TA Kaplan GA Cohen RD Salonen R Salonen JT Moderately intense physical activities and high levels of cardiorespiratory fitness reduce the risk of non-insulin-dependent diabetes mellitus in middle-aged men Arch Intern Med 1996 7 1307 1314 10.1001/archinte.156.12.1307 8651839

[B114] Hu FB Manson JE Stampfer MJ Colditz G Liu S Solomon CG Willett WC Diet, lifestyle, and the risk of type 2 diabetes mellitus in women N Engl J Med 2001 7 790 797 10.1056/NEJMoa010492 11556298

[B115] Schulze MB Hu FB Primary prevention of diabetes: what can be done and how much can be prevented? Annu Rev Public Health 2005 7 445 467 10.1146/annurev.publhealth.26.021304.144532 15760297

[B116] Sato KK Hayashi T Kambe H Nakamura Y Harita N Endo G Yoneda T Walking to work is an independent predictor of incidence of type 2 diabetes in Japanese men: the Kansai Healthcare Study Diabetes Care 2007 7 2296 2298 10.2337/dc07-0090 17536075

[B117] Hu G Qiao Q Silventoinen K Eriksson JG Jousilahti P Lindstrom J Valle TT Nissinen A Tuomilehto J Occupational, commuting, and leisure-time physical activity in relation to risk for Type 2 diabetes in middle-aged Finnish men and women Diabetologia 2003 7 322 329 12687329 10.1007/s00125-003-1031-x

[B118] Hsia J Wu L Allen C Oberman A Lawson WE Torrens J Safford M Limacher MC Howard BV Physical activity and diabetes risk in postmenopausal women Am J Prev Med 2005 7 19 25 10.1016/j.amepre.2004.09.012 15626551

[B119] Folsom AR Kushi LH Hong CP Physical activity and incident diabetes mellitus in postmenopausal women Am J Public Health 2000 7 134 138 10.2105/AJPH.90.1.134 10630154 PMC1446129

[B120] Wannamethee SG Shaper AG Alberti KG Physical activity, metabolic factors, and the incidence of coronary heart disease and type 2 diabetes Arch Intern Med 2000 7 2108 2116 10.1001/archinte.160.14.2108 10904453

[B121] Manson JE Rimm EB Stampfer MJ Colditz GA Willett WC Krolewski AS Rosner B Hennekens CH Speizer FE Physical activity and incidence of non-insulin-dependent diabetes mellitus in women Lancet 1991 7 774 778 10.1016/0140-6736(91)90664-B 1681160

[B122] Helmrich SP Ragland DR Paffenbarger RS Jr Prevention of non-insulin-dependent diabetes mellitus with physical activity Med Sci Sports Exerc 1994 7 824 830 7934754

[B123] Helmrich SP Ragland DR Leung RW Paffenbarger RS Jr Physical activity and reduced occurrence of non-insulin-dependent diabetes mellitus N Engl J Med 1991 7 147 152 2052059 10.1056/NEJM199107183250302

[B124] Wei M Gibbons LW Mitchell TL Kampert JB Lee CD Blair SN The association between cardiorespiratory fitness and impaired fasting glucose and type 2 diabetes mellitus in men Ann Intern Med 1999 7 89 96 10068380 10.7326/0003-4819-130-2-199901190-00002

[B125] Eriksson J Tuominen J Valle T Sundberg S Sovijarvi A Lindholm H Tuomilehto J Koivisto V Aerobic endurance exercise or circuit-type resistance training for individuals with impaired glucose tolerance? Horm Metab Res 1998 7 37 41 10.1055/s-2007-978828 9503037

[B126] Katzmarzyk PT Craig CL Gauvin L Adiposity, physical fitness and incident diabetes: the physical activity longitudinal study Diabetologia 2007 7 538 544 10.1007/s00125-006-0554-3 17221212

[B127] Knowler WC Barrett-Connor E Fowler SE Hamman RF Lachin JM Walker EA Nathan DM Reduction in the incidence of type 2 diabetes with lifestyle intervention or metformin N Engl J Med 2002 7 393 403 10.1056/NEJMoa012512 11832527 PMC1370926

[B128] Tuomilehto J Lindstrom J Eriksson JG Valle TT Hamalainen H Ilanne-Parikka P Keinanen-Kiukaanniemi S Laakso M Louheranta A Rastas M Salminen V Uusitupa M Prevention of type 2 diabetes mellitus by changes in lifestyle among subjects with impaired glucose tolerance N Engl J Med 2001 7 1343 1350 10.1056/NEJM200105033441801 11333990

[B129] Williamson DF Vinicor F Bowman BA Primary prevention of type 2 diabetes mellitus by lifestyle intervention: implications for health policy Ann Intern Med 2004 7 951 957 15172920 10.7326/0003-4819-140-11-200406010-00036

[B130] Wolff I van Croonenborg JJ Kemper HC Kostense PJ Twisk JW The effect of exercise training programs on bone mass: a meta-analysis of published controlled trials in pre- and postmenopausal women Osteoporos Int 1999 7 1 12 10.1007/s001980050109 10367023

[B131] Berard A Bravo G Gauthier P Meta-analysis of the effectiveness of physical activity for the prevention of bone loss in postmenopausal women Osteoporos Int 1997 7 331 337 10.1007/BF01623773 9373566

[B132] Kelley GA Exercise and regional bone mineral density in postmenopausal women: a meta-analytic review of randomized trials Am J Phys Med Rehabil 1998 7 76 87 10.1097/00002060-199801000-00018 9482383

[B133] Kelley GA Aerobic exercise and bone density at the hip in postmenopausal women: a meta-analysis Prev Med 1998 7 798 807 10.1006/pmed.1998.0360 9922061

[B134] Kelley GA Kelley KS Efficacy of resistance exercise on lumbar spine and femoral neck bone mineral density in premenopausal women: a meta-analysis of individual patient data J Womens Health (Larchmt) 2004 7 293 300 10.1089/154099904323016455 15130258

[B135] Bonaiuti D Shea B Iovine R Negrini S Robinson V Kemper HC Wells G Tugwell P Cranney A Exercise for preventing and treating osteoporosis in postmenopausal women Cochrane Database Syst Rev 2002 CD000333 12137611 10.1002/14651858.CD000333

[B136] Brown JP Josse RG 2002 clinical practice guidelines for the diagnosis and management of osteoporosis in Canada CMAJ 2002 7 S1 34 12427685 PMC134653

[B137] Wallace BA Cumming RG Systematic review of randomized trials of the effect of exercise on bone mass in pre- and postmenopausal women Calcif Tissue Int 2000 7 10 18 10.1007/s00223001089 10908406

[B138] Tinetti ME Baker DI McAvay G Claus EB Garrett P Gottschalk M Koch ML Trainor K Horwitz RI A multifactorial intervention to reduce the risk of falling among elderly people living in the community N Engl J Med 1994 7 821 827 10.1056/NEJM199409293311301 8078528

[B139] Wolf SL Barnhart HX Kutner NG McNeely E Coogler C Xu T Reducing frailty and falls in older persons: an investigation of Tai Chi and computerized balance training. Atlanta FICSIT Group. Frailty and Injuries: Cooperative Studies of Intervention Techniques J Am Geriatr Soc 1996 7 489 497 8617895 10.1111/j.1532-5415.1996.tb01432.x

[B140] Carter ND Khan KM Petit MA Heinonen A Waterman C Donaldson MG Janssen PA Mallinson A Riddell L Kruse K Prior JC Flicker L McKay HA Results of a 10 week community based strength and balance training programme to reduce fall risk factors: a randomised controlled trial in 65-75 year old women with osteoporosis Br J Sports Med 2001 7 348 351 10.1136/bjsm.35.5.348 11579072 PMC1724401

[B141] Liu-Ambrose T Khan KM Eng JJ Janssen PA Lord SR McKay HA Resistance and agility training reduce fall risk in women aged 75 to 85 with low bone mass: a 6- month randomized, controlled trial J Am Geriatr Soc 2004 7 657 665 10.1111/j.1532-5415.2004.52200.x 15086643 PMC3344816

[B142] Shaw JM Snow CM Weighted vest exercise improves indices of fall risk in older women J Gerontol A Biol Sci Med Sci 1998 7 M53 58 9467434 10.1093/gerona/53a.1.m53

[B143] Gregg EW Pereira MA Caspersen CJ Physical activity, falls, and fractures among older adults: a review of the epidemiologic evidence J Am Geriatr Soc 2000 7 883 893 10968291 10.1111/j.1532-5415.2000.tb06884.x

[B144] Stevens JA Powell KE Smith SM Wingo PA Sattin RW Physical activity, functional limitations, and the risk of fall-related fractures in community-dwelling elderly Ann Epidemiol 1997 7 54 61 10.1016/S1047-2797(96)00110-X 9034407

[B145] Carter ND Kannus P Khan KM Exercise in the prevention of falls in older people: a systematic literature review examining the rationale and the evidence Sports Med 2001 7 427 438 10.2165/00007256-200131060-00003 11394562

[B146] Boyce WJ Vessey MP Habitual physical inertia and other factors in relation to risk of fracture of the proximal femur Age Ageing 1988 7 319 327 10.1093/ageing/17.5.319 3232586

[B147] Kujala UM Kaprio J Kannus P Sarna S Koskenvuo M Physical activity and osteoporotic hip fracture risk in men Arch Intern Med 2000 7 705 708 10.1001/archinte.160.5.705 10724057

[B148] Joakimsen RM Fonnebo V Magnus JH Stormer J Tollan A Sogaard AJ The Tromso Study: physical activity and the incidence of fractures in a middle-aged population J Bone Miner Res 1998 7 1149 1157 10.1359/jbmr.1998.13.7.1149 9661079

[B149] Feskanich D Willett W Colditz G Walking and leisure-time activity and risk of hip fracture in postmenopausal women JAMA 2002 7 2300 2306 10.1001/jama.288.18.2300 12425707

[B150] Robitaille J Yoon PW Moore CA Liu T Irizarry-Delacruz M Looker AC Khoury MJ Prevalence, family history, and prevention of reported osteoporosis in U.S. women Am J Prev Med 2008 7 47 54 10.1016/j.amepre.2008.03.027 18541176

[B151] Keramat A Patwardhan B Larijani B Chopra A Mithal A Chakravarty D Adibi H Khosravi A The assessment of osteoporosis risk factors in Iranian women compared with Indian women BMC Musculoskelet Disord 2008 7 28 10.1186/1471-2474-9-28 18304358 PMC2289820

[B152] Kerr D Morton A Dick I Prince R Exercise effects on bone mass in postmenopausal women are site-specific and load-dependent J Bone Miner Res 1996 7 218 225 10.1002/jbmr.5650110211 8822346

[B153] Rantanen T Masaki K Foley D Izmirlian G White L Guralnik JM Grip strength changes over 27 yr in Japanese-American men J Appl Physiol 1998 7 2047 2053 9843525 10.1152/jappl.1998.85.6.2047

[B154] Katzmarzyk PT Craig CL Musculoskeletal fitness and risk of mortality Med Sci Sports Exerc 2002 7 740 744 10.1097/00005768-200205001-01269 11984288

[B155] Metter EJ Talbot LA Schrager M Conwit R Skeletal muscle strength as a predictor of all-cause mortality in healthy men J Gerontol A Biol Sci Med Sci 2002 7 B359 365 12242311 10.1093/gerona/57.10.b359

[B156] Fujita Y Nakamura Y Hiraoka J Kobayashi K Sakata K Nagai M Yanagawa H Physical-strength tests and mortality among visitors to health- promotion centers in Japan J Clin Epidemiol 1995 7 1349 1359 10.1016/0895-4356(95)00533-1 7490598

[B157] Brill PA Macera CA Davis DR Blair SN Gordon N Muscular strength and physical function Med Sci Sports Exerc 2000 7 412 416 10.1097/00005768-200002000-00023 10694125

[B158] Mason C Brien SE Craig CL Gauvin L Katzmarzyk PT Musculoskeletal fitness and weight gain in Canada Med Sci Sports Exerc 2007 7 38 43 17218882 10.1249/01.mss.0000240325.46523.cf

[B159] Blair SN LaMonte MJ Nichaman MZ The evolution of physical activity recommendations: how much is enough? Am J Clin Nutr 2004 7 913S 920S 15113739 10.1093/ajcn/79.5.913S

[B160] Paterson DH Warburton DER Physical activity and functional limitations in older adults: a systematic review related to Canada's Physical Activity Guidelines Int J Behav Nutr Phys Act 2010 7 38 20459782 10.1186/1479-5868-7-38 PMC2882898

[B161] Stephenson JB Armstrong A Smith T Bellew B The costs of illness attributable to physical inactivity in Australia: A preliminary study Commonwealth Department of Health and Aged Care and the Australian Sports Commission 2000 http://www.health.gov.au/internet/main/Publishing.nsf/Content/health-pubhlth-publicatdocument- phys_costofillness-cnt.htm/$FILE/phys_costofillness.pdf

[B162] Colditz GA Economic costs of obesity and inactivity Med Sci Sports Exerc 1999 7 S663 667 10.1097/00005768-199911001-00026 10593542

[B163] Andersen LB Schnohr P Schroll M Hein HO All-cause mortality associated with physical activity during leisure time, work, sports, and cycling to work Arch Intern Med 2000 7 1621 1628 10.1001/archinte.160.11.1621 10847255

[B164] Barengo NC Hu G Lakka TA Pekkarinen H Nissinen A Tuomilehto J Low physical activity as a predictor for total and cardiovascular disease mortality in middle-aged men and women in Finland Eur Heart J 2004 7 2204 2211 10.1016/j.ehj.2004.10.009 15589637

[B165] Bath PA Differences between older men and women in the self-rated healthmortality relationship Gerontologist 2003 7 387 395 discussion 372-385 12810903 10.1093/geront/43.3.387

[B166] Bijnen FC Caspersen CJ Feskens EJ Saris WH Mosterd WL Kromhout D Physical activity and 10-year mortality from cardiovascular diseases and all causes: The Zutphen Elderly Study Arch Intern Med 1998 7 1499 1505 10.1001/archinte.158.14.1499 9679790

[B167] Blair SN Kohl HW Barlow CE Physical activity, physical fitness, and all-cause mortality in women: do women need to be active? J Am Coll Nut 1993 7 368 371 10.1080/07315724.1993.10718324 8409097

[B168] Blair SN Kampert JB Kohl HW Barlow CE Macera CA Paffenbarger RS Jr Gibbons LW Influences of cardiorespiratory fitness and other precursors on cardiovascular disease and all-cause mortality in men and women JAMA 1996 7 205 210 10.1001/jama.276.3.205 8667564

[B169] Boyle PA Buchman AS Wilson RS Bienias JL Bennett DA Physical activity is associated with incident disability in community-based older persons J Am Geriatr Soc 2007 7 195 201 10.1111/j.1532-5415.2007.01038.x 17302655

[B170] Bucksch J Physical activity of moderate intensity in leisure time and the risk of all cause mortality Br J Sports Med 2005 7 632 638 10.1136/bjsm.2004.015768 16118301 PMC1725317

[B171] Bucksch J Helmert U Leisure time sports activity and all-cause-mortality in West-Germany (1984-1998) Z Gesundheitswiss 2004 7 351 358 10.1007/s10389-004-0069-7

[B172] Carlsson S Andersson T Wolk A Ahlbom A Low physical activity and mortality in women: baseline lifestyle and health as alternative explanations Scand J Public Health 2006 7 480 487 10.1080/14034940600551293 16990159

[B173] Crespo CJ Palmieri MR Perdomo RP McGee DL Smit E Sempos CT Lee IM Sorlie PD The relationship of physical activity and body weight with all-cause mortality: results from the Puerto Rico Heart Health Program Ann Epidemiol 2002 7 543 552 10.1016/S1047-2797(01)00296-4 12495827

[B174] Davey Smith G Shipley MJ Batty GD Morris JN Marmot M Physical activity and cause-specific mortality in the Whitehall study Public Health 2000 7 308 315 10.1038/sj.ph.1900675 11035446

[B175] Eaton CB Medalie JH Flocke SA Zyzanski SJ Yaari S Goldbourt U Self-reported physical activity predicts long-term coronary heart disease and all-cause mortalities. Twenty-one-year follow-up of the Israeli Ischemic Heart Disease Study Arch Fam Med 1995 7 323 329 10.1001/archfami.4.4.323 7711918

[B176] Fang J Wylie-Rosett J Alderman MH Exercise and cardiovascular outcomes by hypertensive status: NHANES I epidemiological follow-up study, 1971-1992 Am J Hypertens 2005 7 751 758 10.1016/j.amjhyper.2004.12.020 15925731

[B177] Fried LP Kronmal RA Newman AB Bild DE Mittelmark MB Polak JF Robbins JA Gardin JM Risk factors for 5-year mortality in older adults: the Cardiovascular Health Study JAMA 1998 7 585 592 10.1001/jama.279.8.585 9486752

[B178] Fujita K Takahashi H Miura C Ohkubo T Sato Y Ugajin T Kurashima K Tsubono Y Tsuji I Fukao A Hisamichi S Walking and mortality in Japan: the Miyagi Cohort Study J Epidemiol 2004 7 Suppl 1 S26 32 10.2188/jea.14.S26 15143875 PMC8828278

[B179] Glass TA de Leon CM Marottoli RA Berkman LF Population based study of social and productive activities as predictors of survival among elderly Americans BMJ 1999 7 478 483 10454399 10.1136/bmj.319.7208.478 PMC28199

[B180] Gulati M Pandey DK Arnsdorf MF Lauderdale DS Thisted RA Wicklund RH Al-Hani AJ Black HR Exercise capacity and the risk of death in women: the St James Women Take Heart Project Circulation 2003 7 1554 1559 10.1161/01.CIR.0000091080.57509.E9 12975254

[B181] Haapanen N Miilunpalo S Vuori I Oja P Pasanen M Characteristics of leisure time physical activity associated with decreased risk of premature all-cause and cardiovascular disease mortality in middle-aged men Am J Epidemiol 1996 7 870 880 8610700 10.1093/oxfordjournals.aje.a008830

[B182] Hakim AA Petrovitch H Burchfiel CM Ross GW Rodriguez BL White LR Yano K Curb JD Abbott RD Effects of walking on mortality among nonsmoking retired men N Engl J Med 1998 7 94 99 10.1056/NEJM199801083380204 9420340

[B183] Hillsdon M Thorogood M Murphy M Jones L Can a simple measure of vigorous physical activity predict future mortality? Results from the OXCHECK study Public Health Nutr 2004 7 557 562 10.1079/PHN2003548 15153262

[B184] Hu G Tuomilehto J Silventoinen K Barengo NC Peltonen M Jousilahti P The effects of physical activity and body mass index on cardiovascular, cancer and all-cause mortality among 47 212 middle-aged Finnish men and women Int J Obes (Lond) 2005 7 894 902 10.1038/sj.ijo.0802870 15724141

[B185] Hu FB Willett WC Li T Stampfer MJ Colditz GA Manson JE Adiposity as compared with physical activity in predicting mortality among women N Engl J Med 2004 7 2694 2703 10.1056/NEJMoa042135 15616204

[B186] Kampert JB Blair SN Barlow CE Kohl HW Physical activity, physical fitness, and all-cause and cancer mortality: a prospective study of men and women Ann Epidemiol 1996 7 452 457 10.1016/S1047-2797(96)00059-2 8915477

[B187] Kaplan GA Strawbridge WJ Cohen RD Hungerford LR Natural history of leisuretime physical activity and its correlates: associations with mortality from all causes and cardiovascular disease over 28 years Am J Epidemiol 1996 7 793 797 8857828 10.1093/oxfordjournals.aje.a009003

[B188] Khaw KT Jakes R Bingham S Welch A Luben R Day N Wareham N Work and leisure time physical activity assessed using a simple, pragmatic, validated questionnaire and incident cardiovascular disease and all-cause mortality in men and women: The European Prospective Investigation into Cancer in Norfolk prospective population study Int J Epidemiol 2006 7 1034 1043 10.1093/ije/dyl079 16709620

[B189] Kohl HW Nichaman MZ Frankowski RF Blair SN Maximal exercise hemodynamics and risk of mortality in apparently healthy men and women Med Sci Sports Exerc 1996 7 601 609 10.1097/00005768-199605000-00011 9148091

[B190] Kujala UM Kaprio J Sarna S Koskenvuo M Relationship of leisure-time physical activity and mortality: the Finnish twin cohort JAMA 1998 7 440 444 10.1001/jama.279.6.440 9466636

[B191] LaCroix AZ Leveille SG Hecht JA Grothaus LC Wagner EH Does walking decrease the risk of cardiovascular disease hospitalizations and death in older adults? J Am Geriatr Soc 1996 7 113 120 8576498 10.1111/j.1532-5415.1996.tb02425.x

[B192] Lam TH Ho SY Hedley AJ Mak KH Leung GM Leisure time physical activity and mortality in Hong Kong: case-control study of all adult deaths in 1998 Ann Epidemiol 2004 7 391 398 10.1016/j.annepidem.2003.09.005 15246327

[B193] Lan TY Chang HY Tai TY Relationship between components of leisure physical activity and mortality in Taiwanese older adults Prev Med 2006 7 36 41 10.1016/j.ypmed.2006.03.016 16678894

[B194] Laukkanen JA Lakka TA Rauramaa R Kuhanen R Venalainen JM Salonen R Salonen JT Cardiovascular fitness as a predictor of mortality in men Arch Intern Med 2001 7 825 831 10.1001/archinte.161.6.825 11268224

[B195] Lee IM Paffenbarger RS Jr Associations of light, moderate, and vigorous intensity physical activity with longevity. The Harvard Alumni Health Study Am J Epidemiol 2000 7 293 299 10670554 10.1093/oxfordjournals.aje.a010205

[B196] Lee IM Hsieh CC Paffenbarger RS Jr Exercise intensity and longevity in men. The Harvard Alumni Health Study JAMA 1995 7 1179 1184 10.1001/jama.273.15.1179 7707624

[B197] Lee IM Sesso HD Oguma Y Paffenbarger RS Jr The "weekend warrior" and risk of mortality Am J Epidemiol 2004 7 636 641 10.1093/aje/kwh274 15383407

[B198] Leitzmann MF Park Y Blair A Ballard-Barbash R Mouw T Hollenbeck AR Schatzkin A Physical activity recommendations and decreased risk of mortality Arch Intern Med 2007 7 2453 2460 10.1001/archinte.167.22.2453 18071167

[B199] Leon AS Myers MJ Connett J Leisure time physical activity and the 16-year risks of mortality from coronary heart disease and all-causes in the Multiple Risk Factor Intervention Trial (MRFIT) Int J Sports Med 1997 7 Suppl 3 S208 215 10.1055/s-2007-972717 9272851

[B200] Lissner L Bengtsson C Bjorkelund C Wedel H Physical activity levels and changes in relation to longevity. A prospective study of Swedish women Am J Epidemiol 1996 7 54 62 8533747 10.1093/oxfordjournals.aje.a008657

[B201] Manini TM Everhart JE Patel KV Schoeller DA Colbert LH Visser M Tylavsky F Bauer DC Goodpaster BH Harris TB Daily activity energy expenditure and mortality among older adults JAMA 2006 7 171 179 10.1001/jama.296.2.171 16835422

[B202] Matthews CE Jurj AL Shu XO Li HL Yang G Li Q Gao YT Zheng W Influence of exercise, walking, cycling, and overall nonexercise physical activity on mortality in Chinese women Am J Epidemiol 2007 7 1343 1350 10.1093/aje/kwm088 17478434

[B203] Menotti A Seccareccia F Physical activity at work and job responsibility as risk factors for fatal coronary heart disease and other causes of death J Epidemiol Community Health 1985 7 325 329 10.1136/jech.39.4.325 4086963 PMC1052466

[B204] Mensink GB Deketh M Mul MD Schuit AJ Hoffmeister H Physical activity and its association with cardiovascular risk factors and mortality Epidemiology 1996 7 391 397 10.1097/00001648-199607000-00009 8793365

[B205] Morgan K Clarke D Customary physical activity and survival in later life: a study in Nottingham, UK J Epidemiol Community Health 1997 7 490 493 10.1136/jech.51.5.490 9425457 PMC1060533

[B206] Myers J Prakash M Froelicher V Do D Partington S Atwood JE Exercise capacity and mortality among men referred for exercise testing N Engl J Med 2002 7 793 801 10.1056/NEJMoa011858 11893790

[B207] Ostbye T Taylor DH Jung SH A longitudinal study of the effects of tobacco smoking and other modifiable risk factors on ill health in middle-aged and old Americans: results from the Health and Retirement Study and Asset and Health Dynamics among the Oldest Old survey Prev Med 2002 7 334 345 10.1006/pmed.2001.0991 11902850

[B208] Paffenbarger RS Jr Kampert JB Lee IM Hyde RT Leung RW Wing AL Changes in physical activity and other lifeway patterns influencing longevity Med Sci Sports Exerc 1994 7 857 865 7934759

[B209] Richardson CR Kriska AM Lantz PM Hayward RA Physical activity and mortality across cardiovascular disease risk groups Med Sci Sports Exerc 2004 7 1923 1929 10.1249/01.MSS.0000145443.02568.7A 15514508

[B210] Rockhill B Willett WC Manson JE Leitzmann MF Stampfer MJ Hunter DJ Colditz GA Physical activity and mortality: a prospective study among women Am J Public Health 2001 7 578 583 10.2105/AJPH.91.4.578 11291369 PMC1446638

[B211] Rosengren A Wilhelmsen L Physical activity protects against coronary death and deaths from all causes in middle-aged men. Evidence from a 20-year follow-up of the primary prevention study in Goteborg Ann Epidemiol 1997 7 69 75 10.1016/S1047-2797(96)00106-8 9034409

[B212] Schnohr P Scharling H Jensen JS Changes in leisure-time physical activity and risk of death: an observational study of 7,000 men and women Am J Epidemiol 2003 7 639 644 10.1093/aje/kwg207 14507599

[B213] Schnohr C Hojbjerre L Riegels M Ledet L Larsen T Schultz-Larsen K Petersen L Prescott E Gronbaek M Does educational level influence the effects of smoking, alcohol, physical activity, and obesity on mortality? A prospective population study Scand J Public Health 2004 7 250 256 15370764 10.1080/14034940310019489

[B214] Schnohr P Lange P Scharling H Jensen JS Long-term physical activity in leisure time and mortality from coronary heart disease, stroke, respiratory diseases, and cancer. The Copenhagen City Heart Study Eur J Cardiovasc Prev Rehabil 2006 7 173 179 10.1097/01.hjr.0000198923.80555.b7 16575269

[B215] Schooling CM Lam TH Li ZB Ho SY Chan WM Ho KS Tham MK Cowling BJ Leung GM Obesity, physical activity, and mortality in a prospective chinese elderly cohort Arch Intern Med 2006 7 1498 1504 10.1001/archinte.166.14.1498 16864760

[B216] Sundquist K Qvist J Sundquist J Johansson SE Frequent and occasional physical activity in the elderly: a 12-year follow-up study of mortality Am J Prev Med 2004 7 22 27 10.1016/j.amepre.2004.03.011 15212771

[B217] Talbot LA Morrell CH Fleg JL Metter EJ Changes in leisure time physical activity and risk of all-cause mortality in men and women: the Baltimore Longitudinal Study of Aging Prev Med 2007 7 169 176 10.1016/j.ypmed.2007.05.014 17631385

[B218] Trolle-Lagerros Y Mucci LA Kumle M Braaten T Weiderpass E Hsieh CC Sandin S Lagiou P Trichopoulos D Lund E Adami HO Physical activity as a determinant of mortality in women Epidemiology 2005 7 780 785 10.1097/01.ede.0000181312.35964.22 16222168

[B219] Villeneuve PJ Morrison HI Craig CL Schaubel DE Physical activity, physical fitness, and risk of dying Epidemiology 1998 7 626 631 10.1097/00001648-199811000-00011 9799172

[B220] Weller I Corey P The impact of excluding non-leisure energy expenditure on the relation between physical activity and mortality in women Epidemiology 1998 7 632 635 10.1097/00001648-199811000-00012 9799173

[B221] Yu S Yarnell JW Sweetnam PM Murray L What level of physical activity protects against premature cardiovascular death? The Caerphilly study Heart 2003 7 502 506 10.1136/heart.89.5.502 12695452 PMC1767647

[B222] Altieri A Tavani A Gallus S La Vecchia C Occupational and leisure time physical activity and the risk of nonfatal acute myocardial infarction in Italy Ann Epidemiol 2004 7 461 466 10.1016/j.annepidem.2003.11.005 15301782

[B223] Batty GD Shipley MJ Marmot MG Smith GD Leisure time physical activity and disease-specific mortality among men with chronic bronchitis: evidence from the Whitehall study Am J Public Health 2003 7 817 821 10.2105/AJPH.93.5.817 12721150 PMC1447845

[B224] Chen J Millar WJ Health effects of physical activity Health Rep 1999 7 21-30(Eng); 21-31(Fre) 11965821

[B225] Conroy MB Cook NR Manson JE Buring JE Lee IM Past physical activity, current physical activity, and risk of coronary heart disease Med Sci Sports Exerc 2005 7 1251 1256 10.1249/01.mss.0000174882.60971.7f 16118569

[B226] Dorn JP Cerny FJ Epstein LH Naughton J Vena JE Winkelstein W Jr Schisterman E Trevisan M Work and leisure time physical activity and mortality in men and women from a general population sample Ann Epidemiol 1999 7 366 373 10.1016/S1047-2797(99)00025-3 10475536

[B227] Folsom AR Arnett DK Hutchinson RG Liao F Clegg LX Cooper LS Physical activity and incidence of coronary heart disease in middle-aged women and men Med Sci Sports Exerc 1997 7 901 909 9243489 10.1097/00005768-199707000-00009

[B228] Fransson E de Faire U Ahlbom A Reuterwall C Hallqvist J Alfredsson L The risk of acute myocardial infarction: interactions of types of physical activity Epidemiology 2004 7 573 582 10.1097/01.ede.0000134865.74261.fe 15308957

[B229] Fransson E de Faire U Ahlbom A Reuterwall C Hallqvist J Alfredsson L The effect of leisure-time physical activity on the risk of acute myocardial infarction depending on body mass index: a population-based case-control study BMC Public Health 2006 7 296 10.1186/1471-2458-6-296 17156418 PMC1712343

[B230] Haapanen-Niemi N Miilunpalo S Pasanen M Vuori I Oja P Malmberg J Body mass index, physical inactivity and low level of physical fitness as determinants of all-cause and cardiovascular disease mortality--16 y follow-up of middle-aged and elderly men and women Int J Obes Relat Metab Disord 2000 7 1465 1474 10.1038/sj.ijo.0801426 11126344

[B231] Kannel WB Belanger A D'Agostino R Israel I Physical activity and physical demand on the job and risk of cardiovascular disease and death: the Framingham Study Am Heart J 1986 7 820 825 10.1016/0002-8703(86)90480-1 3766383

[B232] Kaprio J Kujala UM Koskenvuo M Sarna S Physical activity and other risk factors in male twin-pairs discordant for coronary heart disease Atherosclerosis 2000 7 193 200 10.1016/S0021-9150(99)00368-8 10781651

[B233] Lakka TA Venalainen JM Rauramaa R Salonen R Tuomilehto J Salonen JT Relation of leisure-time physical activity and cardiorespiratory fitness to the risk of acute myocardial infarction N Engl J Med 1994 7 1549 1554 10.1056/NEJM199406023302201 8177243

[B234] Laukkanen JA Kurl S Salonen R Rauramaa R Salonen JT The predictive value of cardiorespiratory fitness for cardiovascular events in men with various risk profiles: a prospective population-based cohort study Eur Heart J 2004 7 1428 1437 10.1016/j.ehj.2004.06.013 15321701

[B235] Lee IM Sesso HD Paffenbarger RS Jr Physical activity and coronary heart disease risk in men: does the duration of exercise episodes predict risk? Circulation 2000 7 981 986 10961961 10.1161/01.cir.102.9.981

[B236] Lee IM Sesso HD Oguma Y Paffenbarger RS Jr Relative intensity of physical activity and risk of coronary heart disease Circulation 2003 7 1110 1116 10.1161/01.CIR.0000052626.63602.58 12615787

[B237] Lemaitre RN Siscovick DS Raghunathan TE Weinmann S Arbogast P Lin DY Leisure-time physical activity and the risk of primary cardiac arrest Arch Intern Med 1999 7 686 690 10.1001/archinte.159.7.686 10218747

[B238] Lemaitre RN Heckbert SR Psaty BM Siscovick DS Leisure-time physical activity and the risk of nonfatal myocardial infarction in postmenopausal women Arch Intern Med 1995 7 2302 2308 10.1001/archinte.155.21.2302 7487254

[B239] Li TY Rana JS Manson JE Willett WC Stampfer MJ Colditz GA Rexrode KM Hu FB Obesity as compared with physical activity in predicting risk of coronary heart disease in women Circulation 2006 7 499 506 10.1161/CIRCULATIONAHA.105.574087 16449729 PMC3210835

[B240] Lopes C Santos AC Azevedo A Maciel MJ Barros H Physical activity and risk of myocardial infarction after the fourth decade of life Rev Port Cardiol 2005 7 1191 1207 16398237

[B241] Lovasi GS Lemaitre RN Siscovick DS Dublin S Bis JC Lumley T Heckbert SR Smith NL Psaty BM Amount of leisure-time physical activity and risk of nonfatal myocardial infarction Ann Epidemiol 2007 7 410 416 10.1016/j.annepidem.2006.10.012 17321755

[B242] Manson JE Hu FB Rich-Edwards JW Colditz GA Stampfer MJ Willett WC Speizer FE Hennekens CH A prospective study of walking as compared with vigorous exercise in the prevention of coronary heart disease in women N Engl J Med 1999 7 650 658 10.1056/NEJM199908263410904 10460816

[B243] Mora S Cook N Buring JE Ridker PM Lee IM Physical activity and reduced risk of cardiovascular events: potential mediating mechanisms Circulation 2007 7 2110 2118 10.1161/CIRCULATIONAHA.107.729939 17967770 PMC2117381

[B244] O'Connor GT Hennekens CH Willett WC Goldhaber SZ Paffenbarger RS Jr Breslow JL Lee IM Buring JE Physical exercise and reduced risk of nonfatal myocardial infarction Am J Epidemiol 1995 7 1147 1156 7485061 10.1093/oxfordjournals.aje.a117573

[B245] Rastogi T Vaz M Spiegelman D Reddy KS Bharathi AV Stampfer MJ Willett WC Ascherio A Physical activity and risk of coronary heart disease in India Int J Epidemiol 2004 7 759 767 10.1093/ije/dyh042 15044412

[B246] Rodriguez BL Curb JD Burchfiel CM Abbott RD Petrovitch H Masaki K Chiu D Physical activity and 23-year incidence of coronary heart disease morbidity and mortality among middle-aged men. The Honolulu Heart Program Circulation 1994 7 2540 2544 8205662 10.1161/01.cir.89.6.2540

[B247] Rothenbacher D Hoffmeister A Brenner H Koenig W Physical activity, coronary heart disease, and inflammatory response Arch Intern Med 2003 7 1200 1205 10.1001/archinte.163.10.1200 12767957

[B248] Seccareccia F Menotti A Physical activity, physical fitness and mortality in a sample of middle aged men followed-up 25 years J Sports Med Phys Fitness 1992 7 206 213 1434592

[B249] Sesso HD Paffenbarger RS Jr Lee IM Physical activity and coronary heart disease in men: The Harvard Alumni Health Study Circulation 2000 7 975 980 10961960 10.1161/01.cir.102.9.975

[B250] Sundquist K Qvist J Johansson SE Sundquist J The long-term effect of physical activity on incidence of coronary heart disease: a 12-year follow-up study Prev Med 2005 7 219 225 10.1016/j.ypmed.2004.09.043 15917014

[B251] Talbot LA Morrell CH Metter EJ Fleg JL Comparison of cardiorespiratory fitness versus leisure time physical activity as predictors of coronary events in men aged < or = 65 years and > 65 years Am J Cardiol 2002 7 1187 1192 10.1016/S0002-9149(02)02302-0 12008173

[B252] Tanasescu M Leitzmann MF Rimm EB Willett WC Stampfer MJ Hu FB Exercise type and intensity in relation to coronary heart disease in men JAMA 2002 7 1994 2000 10.1001/jama.288.16.1994 12387651

[B253] Vatten LJ Nilsen TI Romundstad PR Droyvold WB Holmen J Adiposity and physical activity as predictors of cardiovascular mortality Eur J Cardiovasc Prev Rehabil 2006 7 909 915 10.1097/01.hjr.0000239463.80390.52 17143122

[B254] Wagner A Simon C Evans A Ferrieres J Montaye M Ducimetiere P Arveiler D Physical activity and coronary event incidence in Northern Ireland and France: the Prospective Epidemiological Study of Myocardial Infarction (PRIME) Circulation 2002 7 2247 2252 10.1161/01.CIR.0000016345.58696.4F 12010905

[B255] Agnarsson U Thorgeirsson G Sigvaldason H Sigfusson N Effects of leisure-time physical activity and ventilatory function on risk for stroke in men: the Reykjavik Study Ann Intern Med 1999 7 987 990 10383369 10.7326/0003-4819-130-12-199906150-00006

[B256] Ellekjaer H Holmen J Ellekjaer E Vatten L Physical activity and stroke mortality in women. Ten-year follow-up of the Nord-Trondelag health survey, 1984-1986 Stroke 2000 7 14 18 10625709 10.1161/01.str.31.1.14

[B257] Evenson KR Rosamond WD Cai J Toole JF Hutchinson RG Shahar E Folsom AR Physical activity and ischemic stroke risk. The atherosclerosis risk in communities study Stroke 1999 7 1333 1339 10390304 10.1161/01.str.30.7.1333

[B258] Haheim LL Holme I Hjermann I Leren P Risk factors of stroke incidence and mortality. A 12-year follow-up of the Oslo Study Stroke 1993 7 1484 1489 8378951 10.1161/01.str.24.10.1484

[B259] Hu G Sarti C Jousilahti P Silventoinen K Barengo NC Tuomilehto J Leisure time, occupational, and commuting physical activity and the risk of stroke Stroke 2005 7 1994 1999 10.1161/01.STR.0000177868.89946.0c 16081862

[B260] Kiely DK Wolf PA Cupples LA Beiser AS Kannel WB Physical activity and stroke risk: the Framingham Study Am J Epidemiol 1994 7 608 620 7942761 10.1093/oxfordjournals.aje.a117298

[B261] Krarup LH Truelsen T Pedersen A Lerke H Lindahl M Hansen L Schnohr P Boysen G Level of physical activity in the week preceding an ischemic stroke Cerebrovasc Dis 2007 7 296 300 10.1159/000105683 17646694

[B262] Kurl S Laukkanen JA Rauramaa R Lakka TA Sivenius J Salonen JT Cardiorespiratory fitness and the risk for stroke in men Arch Intern Med 2003 7 1682 1688 10.1001/archinte.163.14.1682 12885683

[B263] Myint PK Luben RN Wareham NJ Welch AA Bingham SA Day NE Khaw KT Combined work and leisure physical activity and risk of stroke in men and women in the European prospective investigation into Cancer-Norfolk Prospective Population Study Neuroepidemiology 2006 7 122 129 10.1159/000095551 16946623

[B264] Noda H Iso H Toyoshima H Date C Yamamoto A Kikuchi S Koizumi A Kondo T Watanabe Y Wada Y Inaba Y Tamakoshi A Walking and sports participation and mortality from coronary heart disease and stroke J Am Coll Cardiol 2005 7 1761 1767 10.1016/j.jacc.2005.07.038 16256882

[B265] Paganini-Hill A Perez Barreto M Stroke risk in older men and women: aspirin, estrogen, exercise, vitamins, and other factors J Gend Specif Med 2001 7 18 28 11480094

[B266] Pitsavos C Panagiotakos DB Chrysohoou C Kokkinos P Menotti A Singh S Dontas A Physical activity decreases the risk of stroke in middle-age men with left ventricular hypertrophy: 40-year follow-up (1961-2001) of the Seven Countries Study (the Corfu cohort) J Hum Hypertens 2004 7 495 501 10.1038/sj.jhh.1001692 14985777

[B267] Sacco RL Gan R Boden-Albala B Lin IF Kargman DE Hauser WA Shea S Paik MC Leisure-time physical activity and ischemic stroke risk: the Northern Manhattan Stroke Study Stroke 1998 7 380 387 9472878 10.1161/01.str.29.2.380

[B268] Simonsick EM Lafferty ME Phillips CL Mendes de Leon CF Kasl SV Seeman TE Fillenbaum G Hebert P Lemke JH Risk due to inactivity in physically capable older adults Am J Public Health 1993 7 1443 1450 10.2105/AJPH.83.10.1443 8214236 PMC1694862

[B269] Thrift AG Donnan GA McNeil JJ Reduced risk of intracerebral hemorrhage with dynamic recreational exercise but not with heavy work activity Stroke 2002 7 559 564 10.1161/hs0202.102878 11823670

[B270] Folsom AR Prineas RJ Kaye SA Munger RG Incidence of hypertension and stroke in relation to body fat distribution and other risk factors in older women Stroke 1990 7 701 706 2339449 10.1161/01.str.21.5.701

[B271] Levenstein S Smith MW Kaplan GA Psychosocial predictors of hypertension in men and women Arch Intern Med 2001 7 1341 1346 10.1001/archinte.161.10.1341 11371264

[B272] Hou L Ji BT Blair A Dai Q Gao YT Chow WH Commuting physical activity and risk of colon cancer in Shanghai, China Am J Epidemiol 2004 7 860 867 10.1093/aje/kwh301 15496538

[B273] Boutron-Ruault MC Senesse P Meance S Belghiti C Faivre J Energy intake, body mass index, physical activity, and the colorectal adenoma-carcinoma sequence Nutr Cancer 2001 7 50 57 10.1207/S15327914nc391_7 11588902

[B274] Brownson RC Chang JC Davis JR Smith CA Physical activity on the job and cancer in Missouri Am J Public Health 1991 7 639 642 10.2105/AJPH.81.5.639 2014869 PMC1405078

[B275] Calton BA Lacey JV Jr Schatzkin A Schairer C Colbert LH Albanes D Leitzmann MF Physical activity and the risk of colon cancer among women: a prospective cohort study (United States) Int J Cancer 2006 7 385 391 10.1002/ijc.21840 16489545

[B276] Chao A Connell CJ Jacobs EJ McCullough ML Patel AV Calle EE Cokkinides VE Thun MJ Amount, type, and timing of recreational physical activity in relation to colon and rectal cancer in older adults: the Cancer Prevention Study II Nutrition Cohort Cancer Epidemiol Biomarkers Prev 2004 7 2187 2195 15598779

[B277] Colbert LH Hartman TJ Malila N Limburg PJ Pietinen P Virtamo J Taylor PR Albanes D Physical activity in relation to cancer of the colon and rectum in a cohort of male smokers Cancer Epidemiol Biomarkers Prev 2001 7 265 268 11303597

[B278] Dosemeci M Hayes RB Vetter R Hoover RN Tucker M Engin K Unsal M Blair A Occupational physical activity, socioeconomic status, and risks of 15 cancer sites in Turkey Cancer Causes Control 1993 7 313 321 10.1007/BF00051333 8347780

[B279] Friedenreich C Norat T Steindorf K Boutron-Ruault MC Pischon T Mazuir M Clavel-Chapelon F Linseisen J Boeing H Bergman M Johnsen NF Tjonneland A Overvad K Mendez M Quiros JR Martinez C Dorronsoro M Navarro C Gurrea AB Bingham S Khaw KT Allen N Key T Trichopoulou A Trichopoulos D Orfanou N Krogh V Palli D Tumino R Panico S Vineis P Bueno-de-Mesquita HB Peeters PH Monninkhof E Berglund G Manjer J Ferrari P Slimani N Kaaks R Riboli E Physical activity and risk of colon and rectal cancers: the European prospective investigation into cancer and nutrition Cancer Epidemiol Biomarkers Prev 2006 7 2398 2407 10.1158/1055-9965.EPI-06-0595 17164362

[B280] Giovannucci E Ascherio A Rimm EB Colditz GA Stampfer MJ Willett WC Physical activity, obesity, and risk for colon cancer and adenoma in men Ann Intern Med 1995 7 327 334 7847643 10.7326/0003-4819-122-5-199503010-00002

[B281] Isomura K Kono S Moore MA Toyomura K Nagano J Mizoue T Mibu R Tanaka M Kakeji Y Maehara Y Okamura T Ikejiri K Futami K Yasunami Y Maekawa T Takenaka K Ichimiya H Imaizumi N Physical activity and colorectal cancer: the Fukuoka Colorectal Cancer Study Cancer Sci 2006 7 1099 1104 10.1111/j.1349-7006.2006.00282.x 16918995 PMC11158826

[B282] Johnsen NF Christensen J Thomsen BL Olsen A Loft S Overvad K Tjonneland A Physical activity and risk of colon cancer in a cohort of Danish middle-aged men and women Eur J Epidemiol 2006 7 877 884 10.1007/s10654-006-9076-z 17160429

[B283] Larsen IK Grotmol T Almendingen K Hoff G Lifestyle as a predictor for colonic neoplasia in asymptomatic individuals BMC Gastroenterol 2006 7 5 10.1186/1471-230X-6-5 16412216 PMC1374667

[B284] Larsson SC Rutegard J Bergkvist L Wolk A Physical activity, obesity, and risk of colon and rectal cancer in a cohort of Swedish men Eur J Cancer 2006 7 2590 2597 10.1016/j.ejca.2006.04.015 16914307

[B285] Lee IM Paffenbarger RS Jr Physical activity and its relation to cancer risk: a prospective study of college alumni Med Sci Sports Exerc 1994 7 831 837 7934755

[B286] Lee IM Manson JE Ajani U Paffenbarger RS Jr Hennekens CH Buring JE Physical activity and risk of colon cancer: the Physicians' Health Study (United States) Cancer Causes Control 1997 7 568 574 10.1023/A:1018438228410 9242472

[B287] Lee KJ Inoue M Otani T Iwasaki M Sasazuki S Tsugane S Physical activity and risk of colorectal cancer in Japanese men and women: the Japan Public Health Center-based prospective study Cancer Causes Control 2007 7 199 209 10.1007/s10552-006-0098-3 17206529

[B288] Longnecker MP Gerhardsson le Verdier M Frumkin H Carpenter C A case-control study of physical activity in relation to risk of cancer of the right colon and rectum in men Int J Epidemiol 1995 7 42 50 10.1093/ije/24.1.42 7797355

[B289] Mai PL Sullivan-Halley J Ursin G Stram DO Deapen D Villaluna D Horn-Ross PL Clarke CA Reynolds P Ross RK West DW Anton-Culver H Ziogas A Bernstein L Physical activity and colon cancer risk among women in the California Teachers Study Cancer Epidemiol Biomarkers Prev 2007 7 517 525 10.1158/1055-9965.EPI-06-0747 17372247

[B290] Martinez ME Giovannucci E Spiegelman D Hunter DJ Willett WC Colditz GA Leisure-time physical activity, body size, and colon cancer in women. Nurses' Health Study Research Group J Natl Cancer Inst 1997 7 948 955 10.1093/jnci/89.13.948 9214674

[B291] Nilsen TI Romundstad PR Petersen H Gunnell D Vatten LJ Recreational physical activity and cancer risk in subsites of the colon (the Nord-Trondelag Health Study) Cancer Epidemiol Biomarkers Prev 2008 7 183 188 10.1158/1055-9965.EPI-07-0746 18199723

[B292] Schnohr P Gronbaek M Petersen L Hein HO Sorensen TI Physical activity in leisure-time and risk of cancer: 14-year follow-up of 28,000 Danish men and women Scand J Public Health 2005 7 244 249 10.1080/14034940510005752 16087486

[B293] Slattery ML Schumacher MC Smith KR West DW Abd-Elghany N Physical activity, diet, and risk of colon cancer in Utah Am J Epidemiol 1988 7 989 999 3189298 10.1093/oxfordjournals.aje.a115072

[B294] Slattery ML Edwards SL Ma KN Friedman GD Potter JD Physical activity and colon cancer: a public health perspective Ann Epidemiol 1997 7 137 145 10.1016/S1047-2797(96)00129-9 9099401

[B295] Slattery ML Potter J Caan B Edwards S Coates A Ma KN Berry TD Energy balance and colon cancer--beyond physical activity Cancer Res 1997 7 75 80 8988044

[B296] Takahashi H Kuriyama S Tsubono Y Nakaya N Fujita K Nishino Y Shibuya D Tsuji I Time spent walking and risk of colorectal cancer in Japan: the Miyagi Cohort study Eur J Cancer Prev 2007 7 403 408 10.1097/01.cej.0000236249.63489.05 17923810

[B297] Tang R Wang JY Lo SK Hsieh LL Physical activity, water intake and risk of colorectal cancer in Taiwan: a hospital-based case-control study Int J Cancer 1999 7 484 489 10.1002/(SICI)1097-0215(19990812)82:4<484::AID-IJC3>3.0.CO;2-A 10404059

[B298] Tavani A Braga C La Vecchia C Conti E Filiberti R Montella M Amadori D Russo A Franceschi S Physical activity and risk of cancers of the colon and rectum: an Italian case-control study Br J Cancer 1999 7 1912 1916 10.1038/sj.bjc.6690304 10206313 PMC2362771

[B299] Thune I Lund E Physical activity and risk of colorectal cancer in men and women Br J Cancer 1996 7 1134 1140 8624277 10.1038/bjc.1996.218 PMC2074402

[B300] Vena JE Graham S Zielezny M Swanson MK Barnes RE Nolan J Lifetime occupational exercise and colon cancer Am J Epidemiol 1985 7 357 365 4025286 10.1093/oxfordjournals.aje.a114116

[B301] Vetter R Dosemeci M Blair A Wacholder S Unsal M Engin K Fraumeni JF Jr Occupational physical activity and colon cancer risk in Turkey Eur J Epidemiol 1992 7 845 850 10.1007/BF00145330 1294390

[B302] White E Jacobs EJ Daling JR Physical activity in relation to colon cancer in middle-aged men and women Am J Epidemiol 1996 7 42 50 8659484 10.1093/oxfordjournals.aje.a008853

[B303] Wolin KY Lee IM Colditz GA Glynn RJ Fuchs C Giovannucci E Leisure-time physical activity patterns and risk of colon cancer in women Int J Cancer 2007 7 2776 2781 10.1002/ijc.23009 17722094 PMC2291204

[B304] Zhang Y Cantor KP Dosemeci M Lynch CF Zhu Y Zheng T Occupational and leisure-time physical activity and risk of colon cancer by subsite J Occup Environ Med 2006 7 236 243 10.1097/01.jom.0000199521.72764.26 16531827

[B305] Bernstein L Henderson BE Hanisch R Sullivan-Halley J Ross RK Physical exercise and reduced risk of breast cancer in young women J Natl Cancer Inst 1994 7 1403 1408 10.1093/jnci/86.18.1403 8072034

[B306] Bernstein L Patel AV Ursin G Sullivan-Halley J Press MF Deapen D Berlin JA Daling JR McDonald JA Norman SA Malone KE Strom BL Liff J Folger SG Simon MS Burkman RT Marchbanks PA Weiss LK Spirtas R Lifetime recreational exercise activity and breast cancer risk among black women and white women J Natl Cancer Inst 2005 7 1671 1679 16288120 10.1093/jnci/dji374

[B307] Carpenter CL Ross RK Paganini-Hill A Bernstein L Lifetime exercise activity and breast cancer risk among post-menopausal women Br J Cancer 1999 7 1852 1858 10.1038/sj.bjc.6690610 10468309 PMC2374273

[B308] Carpenter CL Ross RK Paganini-Hill A Bernstein L Effect of family history, obesity and exercise on breast cancer risk among postmenopausal women Int J Cancer 2003 7 96 102 10.1002/ijc.11186 12794763

[B309] Chang SC Ziegler RG Dunn B Stolzenberg-Solomon R Lacey JV Jr Huang WY Schatzkin A Reding D Hoover RN Hartge P Leitzmann MF Association of energy intake and energy balance with postmenopausal breast cancer in the prostate, lung, colorectal, and ovarian cancer screening trial Cancer Epidemiol Biomarkers Prev 2006 7 334 341 10.1158/1055-9965.EPI-05-0479 16492925

[B310] Colditz GA Feskanich D Chen WY Hunter DJ Willett WC Physical activity and risk of breast cancer in premenopausal women Br J Cancer 2003 7 847 851 10.1038/sj.bjc.6601175 12942116 PMC2394493

[B311] Coogan PF Newcomb PA Clapp RW Trentham-Dietz A Baron JA Longnecker MP Physical activity in usual occupation and risk of breast cancer (United States) Cancer Causes Control 1997 7 626 631 10.1023/A:1018402615206 9242479

[B312] Coogan PF Aschengrau A Occupational physical activity and breast cancer risk in the upper Cape Cod cancer incidence study Am J Ind Med 1999 7 279 285 10.1002/(SICI)1097-0274(199908)36:2<279::AID-AJIM7>3.0.CO;2-7 10398936

[B313] Dallal CM Sullivan-Halley J Ross RK Wang Y Deapen D Horn-Ross PL Reynolds P Stram DO Clarke CA Anton-Culver H Ziogas A Peel D West DW Wright W Bernstein L Long-term recreational physical activity and risk of invasive and in situ breast cancer: the California teachers study Arch Intern Med 2007 7 408 415 10.1001/archinte.167.4.408 17325304

[B314] Dirx MJ Voorrips LE Goldbohm RA Brandt PA van den Baseline recreational physical activity, history of sports participation, and postmenopausal breast carcinoma risk in the Netherlands Cohort Study Cancer 2001 7 1638 1649 10.1002/1097-0142(20010915)92:6<1638::AID-CNCR1490>3.0.CO;2-Q 11745243

[B315] Dorn J Vena J Brasure J Freudenheim J Graham S Lifetime physical activity and breast cancer risk in pre- and postmenopausal women Med Sci Sports Exerc 2003 7 278 285 10.1249/01.MSS.0000048835.59454.8D 12569217

[B316] Drake DA A longitudinal study of physical activity and breast cancer prediction Cancer Nurs 2001 7 371 377 10.1097/00002820-200110000-00008 11605707

[B317] Friedenreich CM Bryant HE Courneya KS Case-control study of lifetime physical activity and breast cancer risk Am J Epidemiol 2001 7 336 347 10.1093/aje/154.4.336 11495857

[B318] Friedenreich CM Courneya KS Bryant HE Relation between intensity of physical activity and breast cancer risk reduction Med Sci Sports Exerc 2001 7 1538 1545 10.1097/00005768-200109000-00018 11528344

[B319] Friedenreich CM Rohan TE Physical activity and risk of breast cancer Eur J Cancer Prev 1995 7 145 151 10.1097/00008469-199504000-00004 7767240

[B320] Gammon MD Schoenberg JB Britton JA Kelsey JL Coates RJ Brogan D Potischman N Swanson CA Daling JR Stanford JL Brinton LA Recreational physical activity and breast cancer risk among women under age 45 years Am J Epidemiol 1998 7 273 280 9482502 10.1093/oxfordjournals.aje.a009447

[B321] Gilliland FD Li YF Baumgartner K Crumley D Samet JM Physical activity and breast cancer risk in hispanic and non-hispanic white women Am J Epidemiol 2001 7 442 450 10.1093/aje/154.5.442 11532786

[B322] Hsing AW McLaughlin JK Cocco P Co Chien HT Fraumeni JF Jr Risk factors for male breast cancer (United States) Cancer Causes Control 1998 7 269 275 10.1023/A:1008869003012 9684707

[B323] Hu YH Nagata C Shimizu H Kaneda N Kashiki Y Association of body mass index, physical activity, and reproductive histories with breast cancer: a case-control study in Gifu, Japan Breast Cancer Res Treat 1997 7 65 72 10.1023/A:1005745824388 9065600

[B324] John EM Horn-Ross PL Koo J Lifetime physical activity and breast cancer risk in a multiethnic population: the San Francisco Bay area breast cancer study Cancer Epidemiol Biomarkers Prev 2003 7 1143 1152 14652273

[B325] Kruk J Lifetime physical activity and the risk of breast cancer: a case-control study Cancer Detect Prev 2007 7 18 28 10.1016/j.cdp.2006.12.003 17296272

[B326] Kruk J Leisure-time physical activity in relation to the risk of breast cancer European Journal of Sports Science 2007 7 81 91 10.1080/17461390701401813

[B327] Lahmann PH Friedenreich C Schuit AJ Salvini S Allen NE Key TJ Khaw KT Bingham S Peeters PH Monninkhof E Bueno-de-Mesquita HB Wirfalt E Manjer J Gonzales CA Ardanaz E Amiano P Quiros JR Navarro C Martinez C Berrino F Palli D Tumino R Panico S Vineis P Trichopoulou A Bamia C Trichopoulos D Boeing H Schulz M Linseisen J Chang-Claude J Chapelon FC Fournier A Boutron-Ruault MC Tjonneland A Fons Johnson N Overvad K Kaaks R Riboli E Physical activity and breast cancer risk: the European Prospective Investigation into Cancer and Nutrition Cancer Epidemiol Biomarkers Prev 2007 7 36 42 10.1158/1055-9965.EPI-06-0582 17179488

[B328] Lee IM Rexrode KM Cook NR Hennekens CH Burin JE Physical activity and breast cancer risk: the Women's Health Study (United States) Cancer Causes Control 2001 7 137 145 10.1023/A:1008948125076 11246842

[B329] Magnusson CM Roddam AW Pike MC Chilvers C Crossley B Hermon C McPherson K Peto J Vessey M Beral V Body fatness and physical activity at young ages and the risk of breast cancer in premenopausal women Br J Cancer 2005 7 817 824 10.1038/sj.bjc.6602758 16160699 PMC2361642

[B330] Malin A Matthews CE Shu XO Cai H Dai Q Jin F Gao YT Zheng W Energy balance and breast cancer risk Cancer Epidemiol Biomarkers Prev 2005 7 1496 1501 10.1158/1055-9965.EPI-04-0880 15941962 PMC1592607

[B331] Margolis KL Mucci L Braaten T Kumle M Trolle Lagerros Y Adami HO Lund E Weiderpass E Physical activity in different periods of life and the risk of breast cancer: the Norwegian-Swedish Women's Lifestyle and Health cohort study Cancer Epidemiol Biomarkers Prev 2005 7 27 32 15668472

[B332] McTiernan A Stanford JL Weiss NS Daling JR Voigt LF Occurrence of breast cancer in relation to recreational exercise in women age 50-64 years Epidemiology 1996 7 598 604 10.1097/00001648-199611000-00006 8899385

[B333] McTiernan A Kooperberg C White E Wilcox S Coates R Adams-Campbell LL Woods N Ockene J Recreational physical activity and the risk of breast cancer in postmenopausal women: the Women's Health Initiative Cohort Study JAMA 2003 7 1331 1336 10.1001/jama.290.10.1331 12966124

[B334] Silvera SA Jain M Howe GR Miller AB Rohan TE Energy balance and breast cancer risk: a prospective cohort study Breast Cancer Res Treat 2006 7 97 106 10.1007/s10549-005-9098-3 16319973

[B335] Patel AV Callel EE Bernstein L Wu AH Thun MJ Recreational physical activity and risk of postmenopausal breast cancer in a large cohort of US women Cancer Causes Control 2003 7 519 529 10.1023/A:1024895613663 12948283

[B336] Patel AV Press MF Meeske K Calle EE Bernstein L Lifetime recreational exercise activity and risk of breast carcinoma in situ Cancer 2003 7 2161 2169 10.1002/cncr.11768 14601085

[B337] Rintala PE Pukkala E Paakkulainen HT Vihko VJ Self-experienced physical workload and risk of breast cancer Scand J Work Environ Health 2002 7 158 162 12109554 10.5271/sjweh.659

[B338] Rockhill B Willett WC Hunter DJ Manson JE Hankinson SE Spiegelman D Colditz GA Physical activity and breast cancer risk in a cohort of young women J Natl Cancer Inst 1998 7 1155 1160 10.1093/jnci/90.15.1155 9701365

[B339] Slattery ML Edwards S Murtaugh MA Sweeney C Herrick J Byers T Giuliano AR Baumgartner KB Physical activity and breast cancer risk among women in the southwestern United States Ann Epidemiol 2007 7 342 353 10.1016/j.annepidem.2006.10.017 17462544 PMC2925501

[B340] Sprague BL Trentham-Dietz A Newcomb PA Titus-Ernstoff L Hampton JM Egan KM Lifetime recreational and occupational physical activity and risk of in situ and invasive breast cancer Cancer Epidemiol Biomarkers Prev 2007 7 236 243 10.1158/1055-9965.EPI-06-0713 17301255

[B341] Steindorf K Schmidt M Kropp S Chang-Claude J Case-control study of physical activity and breast cancer risk among premenopausal women in Germany Am J Epidemiol 2003 7 121 130 10.1093/aje/kwf181 12522019

[B342] Tehard B Friedenreich CM Oppert JM Clavel-Chapelon F Effect of physical activity on women at increased risk of breast cancer: results from the E3N cohort study Cancer Epidemiol Biomarkers Prev 2006 7 57 64 10.1158/1055-9965.EPI-05-0603 16434587

[B343] Thune I Brenn T Lund E Gaard M Physical activity and the risk of breast cancer N Engl J Med 1997 7 1269 1275 10.1056/NEJM199705013361801 9113929

[B344] Zheng W Shu XO McLaughlin JK Chow WH Gao YT Blot WJ Occupational physical activity and the incidence of cancer of the breast, corpus uteri, and ovary in Shanghai Cancer 1993 7 3620 3624 10.1002/1097-0142(19930601)71:11<3620::AID-CNCR2820711125>3.0.CO;2-S 8490910

[B345] Burchfiel CM Sharp DS Curb JD Rodriguez BL Hwang LJ Marcus EB Yano K Physical activity and incidence of diabetes: the Honolulu Heart Program Am J Epidemiol 1995 7 360 368 10.1093/aje/141.4.360 7840114

[B346] Dziura J Kasl SV DiPietro L Physical activity reduces type 2 diabetes risk in aging independent of body weight change J Phys Activity Health 2004 7 19 28

[B347] Hu FB Sigal RJ Rich-Edwards JW Colditz GA Solomon CG Willett WC Speizer FE Manson JE Walking compared with vigorous physical activity and risk of type 2 diabetes in women: a prospective study JAMA 1999 7 1433 1439 10.1001/jama.282.15.1433 10535433

[B348] Hu FB Leitzmann MF Stampfer MJ Colditz GA Willett WC Rimm EB Physical activity and television watching in relation to risk for type 2 diabetes mellitus in men Arch Intern Med 2001 7 1542 1548 10.1001/archinte.161.12.1542 11427103

[B349] Rana JS Li TY Manson JE Hu FB Adiposity compared with physical inactivity and risk of type 2 diabetes in women Diabetes Care 2007 7 53 58 10.2337/dc06-1456 17192333

[B350] Sawada SS Lee IM Muto T Matuszaki K Blair SN Cardiorespiratory fitness and the incidence of type 2 diabetes: prospective study of Japanese men Diabetes Care 2003 7 2918 2922 10.2337/diacare.26.10.2918 14514602

[B351] Weinstein AR Sesso HD Lee IM Cook NR Manson JE Buring JE Gaziano JM Relationship of physical activity vs body mass index with type 2 diabetes in women JAMA 2004 7 1188 1194 10.1001/jama.292.10.1188 15353531

